# Hybrid versus vaccine immunity of mRNA-1273 among people living with HIV in East and Southern Africa: a prospective cohort analysis from the multicentre CoVPN 3008 (Ubuntu) study

**DOI:** 10.1016/j.eclinm.2024.103054

**Published:** 2025-01-20

**Authors:** Nigel Garrett, Asa Tapley, Aaron Hudson, Sufia Dadabhai, Bo Zhang, Nyaradzo M. Mgodi, Jessica Andriesen, Azwidihwi Takalani, Leigh H. Fisher, Jia Jin Kee, Craig A. Magaret, Manuel Villaran, John Hural, Erica Andersen-Nissen, Guido Ferarri, Maurine D. Miner, Bert Le Roux, Eduan Wilkinson, Richard Lessells, Tulio de Oliveira, Jackline Odhiambo, Parth Shah, Laura Polakowski, Margaret Yacovone, Taraz Samandari, Zvavahera Chirenje, Peter James Elyanu, Joseph Makhema, Ethel Kamuti, Harriet Nuwagaba-Biribonwoha, Sharlaa Badal-Faesen, William Brumskine, Soritha Coetzer, Rodney Dawson, Sinead Delany-Moretlwe, Andreas Henri Diacon, Samantha Fry, Katherine Margaret Gill, Zaheer Ahmed Ebrahim Hoosain, Mina C. Hosseinipour, Mubiana Inambao, Craig Innes, Steve Innes, Dishiki Kalonji, Margaret Kasaro, Priya Kassim, Noel Kayange, William Kilembe, Fatima Laher, Moelo Malahleha, Vongane Louisa Maluleke, Grace Mboya, Kirsten McHarry, Essack Mitha, Kathryn Mngadi, Pamela Mda, Tumelo Moloantoa, Cissy Kityo Mutuluuza, Nivashnee Naicker, Vimla Naicker, Anusha Nana, Annet Nanvubya, Maphoshane Nchabeleng, Walter Otieno, Elsje Louise Potgieter, Disebo Potloane, Zelda Punt, Jamil Said, Yashna Singh, Mohammed Siddique Tayob, Yacoob Vahed, Deo Ogema Wabwire, M. Juliana McElrath, James G. Kublin, Linda-Gail Bekker, Peter B. Gilbert, Lawrence Corey, Glenda E. Gray, Yunda Huang, Philip Kotze, CoVPN 3008 Ubuntu Study Teambn, Sharlaa Badal-Faesen, Kagisho Baepanye, Veronique Bailey, Katekani Baloyi-Oseh, Mumtaz Booley, Johannes Louis Botha, Yolande Brown, Valerie Brown, Lisa Bunts, Soritha Coetzer, Myron Cohen, Shirley Collie, Rodney Dawson, Pallabi Deb, Hana El Sahly, Jill El-Khorazaty, Andries Engelbrecht, Marianne Gildea, Dhevium Govender, Jen Hanke, Jayla Harris, Simone Hendricks, Nick Hopkinson, Haley Howell, Nzeera Ketter, Kentse Khuto, Faatima Laher Omar, Leolin Katsidzira, Kim Linton, James Ludwig, Bongile Mabilane, Matshidiso Malefo, Ndiitwani Mamushiana, Daciana Margineantu, Jeanine May, Fatima Mayat, Cindy Molitor, Yeshnee Naidoo, Michelle Nebergall, Alan Nguyen, Sarah Nikles, Bianca Noronha, Melissa Peda, Tamara Phiri, Shanthie Pillay, Sureshnee Pillay, Lori Proulx-Burns, Laurie Rinn, Lisa Sanders, Carrie Sopher, Smitha Sripathy, Michael Stirewalt, Houriiyah Tegally, Sara Thiebaud, Alicia Toledano, Stephanie Van Wyk, Shamaya Whitby, Stephany Wilcox, Eduan Wilkinson, Haven Wilvich, Charles Wiysonge, Nelisiwe Xaba, Ntokozo Xulu

**Affiliations:** a Centre for the AIDS Programme of Research in South Africa, University of KwaZulu–Natal, Durban, South Africa; b Discipline of Public Health Medicine, School of Nursing and Public Health, University of KwaZulu-Natal, Durban, South Africa; c Division of Allergy and Infectious Diseases, Department of Medicine, University of Washington, Seattle, USA; d Vaccine and Infectious Disease Division, Fred Hutchinson Cancer Center, Seattle, USA; e Department of Medicine, University of Cape Town, Cape Town, South Africa; f Johns Hopkins Research Project, Blantyre, Malawi; g Clinical Trials Research Centre, University of Zimbabwe, Harare, Zimbabwe; h Hutchinson Centre Research Institute of South Africa, Chris Hani Baragwanath Academic Hospital, Soweto, South Africa; i Cape Town HVTN Immunology Laboratory, Hutchinson Centre Research Institute of South Africa, Cape Town, South Africa; j Duke University Human Vaccine Institute, Duke University School of Medicine, Durham, NC, USA; k Center for HIV/AIDS Vaccine Immunology, Duke University School of Medicine, Durham, NC, USA; l KwaZulu-Natal Research Innovation & Sequencing Platform, School of Laboratory Medicine and Medical Sciences, University of KwaZulu-Natal, Durban, South Africa; m Centre for Epidemic Response & Innovation, Stellenbosch, South Africa; n Public Health Sciences Division, Fred Hutchinson Cancer Center, Seattle, USA; o National Institute of Allergy and Infectious Diseases, National Institutes of Health, Bethesda, USA; p COVID-19 Prevention Network, Seattle, USA; q Baylor College of Medicine Children's Foundation-Uganda, Kampala, Uganda; r Botswana Harvard AIDS Institute, Gaborone, Botswana; s Centre for Infectious Disease Research in Zambia, Lusaka, Zambia; t ICAP at Columbia University, Eswatini Prevention Center, Mbabane, Eswatini; u Department of Epidemiology, Mailman School of Public Health, Columbia University, New York, USA; v Clinical HIV Research Unit/Helen Joseph Clinical Research Site, Johannesburg, South Africa; w The Aurum Institute, Rustenburg Clinical Research Site, Rustenburg, South Africa; x Synexus Helderberg, Cape Town, South Africa; y University of Cape Town Lung Institute Clinical Research Site, Cape Town, South Africa; z Wits RHI University of the Witwatersrand, Johannesburg, South Africa; aa TASK Central, Cape Town, South Africa; ab FAMCRU Family Clinical Research Unit, Cape Town, South Africa; ac Desmond Tutu HIV Centre, University of Cape Town, Cape Town, South Africa; ad Josha Research Clinical Research Site, Bloemfontein, South Africa; ae Malawi Clinical Research Site, Lilongwe, Malawi; af University of North Carolina at Chapel Hill School of Medicine, Chapel Hill, USA; ag CFHRZ - Ndola Clinical Research Site, Ndola, Zambia; ah The Aurum Institute, Klerksdorp Clinical Research Site, Klerksdorp, South Africa; ai South African Medical Research Council, Isipingo Clinical Research Site, KwaZulu-Natal, South Africa; aj UNC Global Projects/Kamwala District Health Centre, Lusaka, Zambia; ak Soweto - Kliptown Clinical Research Site, Soweto, South Africa; al Blantyre Clinical Research Site, Blantyre, Malawi; am CFHRZ Clinical Research Site, Lusaka, Zambia; an Perinatal HIV Research Unit, Faculty of Health Sciences, University of the Witwatersrand, South Africa; ao Synergy Biomed Research Institute, East London, South Africa; ap MERC Middelburg, Middelburg, South Africa; aq Kisumu Clinical Research Site, Kisumu, Kenya; ar TASK Eden, Western Cape, South Africa; as Newtown Clinical Research, Johannesburg, South Africa; at Tembisa Clinic 4, Gauteng, South Africa; au Nelson Mandela Academic Clinical Research Unit Clinical Research Site, Mthatha, South Africa; av PHRU Matlosana Clinical Research Site, Klerksdorp, South Africa; aw Joint Clinical Research Centre, Lubowa, Uganda; ax Tongaat Clinical Research Site, KwaZulu-Natal, South Africa; ay UVRI-IAVI HIV Vaccine Program Ltd. Clinical Research Site, Entebbe, Uganda; az MeCRU Clinical Research Site, Pretoria, South Africa; ba Kombewa Clinical Research Site, Kisumu, Kenya; bb Maseno University School of Medicine, Kenya; bc Synexus Stanza Clinical Research Centre Clinical Research Site, Pretoria, South Africa; bd PHOENIX Pharma (Pty) Ltd, Port Elizabeth, South Africa; be Moi University Clinical Research Centre, Eldoret, Kenya; bf MERC Kempton, Kempton, South Africa; bg MERC Welkom, Welkom, South Africa; bh MU-JHU Research Collaboration Clinical Research Site, Kampala, Uganda; bi South African Medical Research Council, Pretoria, South Africa; bj Department of Global Health, University of Washington, Seattle, USA; bk Qhakaza Mbokodo Research Clinic, Ladysmith, South Africa

**Keywords:** People living with HIV, SARS-CoV-2, mRNA-1273, Hybrid immunity, Vaccine immunity, COVID-19

## Abstract

**Background:**

With limited access to mRNA COVID-19 vaccines in lower income countries, and people living with HIV (PLWH) largely excluded from clinical trials, Part A of the multicentre CoVPN 3008 (Ubuntu) study aimed to assess the safety of mRNA-1273, the relative effectiveness of hybrid versus vaccine immunity, and SARS-CoV-2 viral persistence among PLWH in East and Southern Africa during the omicron outbreak.

**Methods:**

Previously unvaccinated adults with HIV and/or other comorbidities associated with severe COVID-19 received either one (hybrid immunity) or two (vaccine immunity) 100-mcg doses of ancestral strain mRNA-1273 in the first month, depending on baseline evidence of prior SARS-CoV-2 infection. In a prospective cohort study design, we used covariate-adjusted Cox regression and counterfactual cumulative incidence methods to determine the hazard ratio and relative risk of COVID-19 and severe COVID-19 with hybrid versus vaccine immunity within six months. The ongoing Ubuntu study is registered on ClinicalTrials.gov (NCT05168813) and this work was conducted from December 2021 to March 2023.

**Findings:**

Between December 2021 and September 2022, 14,237 participants enrolled, and 14,002 (83% PLWH, 69% SARS-CoV-2 seropositive) were included in the analyses. Vaccinations were safe and well tolerated. Common adverse events were pain or tenderness at the injection site (26.7%), headache (20.4%), and malaise (20.3%). Severe adverse events were rare (0.8% of participants after the first and 1.1% after the second vaccination), and none were life-threatening or fatal. Among PLWH, the median CD4 count was 635 cells/μl and 18.5% had HIV viraemia. The six-month cumulative incidences in the hybrid immunity and vaccine immunity groups were 2.02% (95% confidence interval [CI] 1.61–2.44) and 3.40% (95% CI 2.30–4.49) for COVID-19, and 0.048% (95% CI 0.00–0.10) and 0.32% (95% CI 0.59–0.63) for severe COVID-19. Among all PLWH the hybrid immunity group had a 42% lower hazard rate of COVID-19 (hazard ratio [HR] 0.58; 95% CI 0.44–0.77; p < 0.001) and a 73% lower hazard rate of severe COVID-19 (HR 0.27; 95% CI 0.07–1.04; p = 0.056) than the vaccine immunity group, but this effect was not seen among PLWH with CD4 counts <350 cells/μl or HIV viraemia. Twenty PLWH had persistent SARS-CoV-2 virus at least 50 days.

**Interpretation:**

Hybrid immunity was associated with superior protection from COVID-19 compared to vaccine immunity with the ancestral mRNA-1273 vaccine. Persistent infections among immunocompromised PLWH may provide reservoirs for emerging variants.

**Funding:**

10.13039/100000060National Institute of Allergy and Infectious Diseases.

**Research in context**

**Evidence before this study**

We searched PubMed and ClinicalTrials.gov using terms related to “HIV”, “SARS-CoV-2”, “COVID-19”, “vaccine”, and “hybrid immunity” with no language or date restrictions. Several observational studies and case reports have previously indicated that individuals with well-managed HIV likely achieve COVID-19 mRNA vaccine immune responses comparable to people living without HIV, while people living with HIV (PLWH) with lower CD4 counts may have an impaired response. Although clinical trials of COVID-19 mRNA vaccines demonstrated high vaccine efficacy and excellent safety in the general population, PLWH were largely excluded from these trials, in particular immunocompromised PLWH, or those not on antiretroviral therapy with HIV viraemia. Moreover, the impact of past infection on immunity in PLWH was uncertain. Evidence from observational studies indicated that a previous infection could prime the immune system and boost the effectiveness of vaccines, leading to ‘hybrid immunity’ which is more robust than ‘vaccine immunity’ derived solely from vaccination. However, there had been few evaluations of the hybrid immunity effect in large cohort studies, especially among PLWH.

**Added value of this study**

The CoVPN 3008 (Ubuntu) study is one of the largest prospective studies of a COVID-19 vaccine conducted exclusively in Africa, and the only such trial to enrol a large and diverse population of PLWH, including pregnant persons and individuals with poorly controlled HIV. Furthermore, it is one of the largest studies investigating COVID-19 mRNA vaccines during the omicron outbreak, and one of the only to evaluate hybrid immunity. We found that overall hybrid immunity was associated with a significant reduction in the risk of COVID-19 and severe COVID-19 compared to vaccine immunity, but this effect did not seem to hold among PLWH with low CD4 counts or HIV viraemia. The vaccines also appeared safe and well tolerated in PLWH. Severe COVID-19 was rare in Ubuntu, and most infections were mild or asymptomatic. Importantly, the study identified a subpopulation of participants with persistent SARS-CoV-2 shedding, more often observed among PLWH with a detectable HIV viral load or low CD4 count.

**Implications of all the available evidence**

Our findings support the superior efficacy of hybrid immunity over vaccination alone against COVID-19, including among individuals with well-controlled HIV. While ethical constraints excluded an unvaccinated comparator, the enhanced protection observed with hybrid immunity in the Per Protocol cohort suggests vaccination significantly contributed to its advantage. The lack of benefit from hybrid immunity among participants with poorly controlled HIV underscores the importance of strengthening HIV care. Additionally, the study confirms the safety and tolerability of COVID-19 mRNA vaccines, including in PLWH, and highlights the risk of persistent SARS-CoV-2 shedding in immunocompromised PLWH, who may serve as reservoirs for emerging variants. These findings emphasise the importance of improved HIV care, the development of next-generation COVID-19 vaccines, and further research into prolonged SARS-CoV-2 infections.

## Introduction

As the coronavirus disease of 2019 (COVID-19) pandemic transitions to endemicity, vaccines remain essential to the public health response. mRNA vaccines have been effective in preventing severe COVID-19,^1^^,^^2^ but global access has been inequitable,^3^ and knowledge gaps in vaccine efficacy remain, particularly for immunocompromised populations.

Although people living with HIV (PLWH) are at increased risk of severe COVID-19,^4^ only a few, with well-controlled HIV infection, were included in clinical trials of the BNT162b2 and mRNA-1273 vaccines, resulting in insufficient endpoints to draw meaningful conclusions.^5^ Observational studies have suggested that individuals with well-controlled HIV mount similar vaccine responses to people living without HIV (PLWoH),^6^ but that those with HIV viraemia and immunosuppression are especially vulnerable to poor COVID-19 outcomes.^7^^,^^8^

The role of previous infection in shaping immunity among PLWH remains unclear. Evidence suggests that prior infection primes the immune system and enhances vaccine benefits (‘hybrid immunity’) compared to immunity from vaccination alone (‘vaccine immunity’),^9^ yet there have been limited evaluations of the hybrid immunity effect in large cohort studies, particularly among PLWH. This issue is especially relevant in Africa, where SARS-CoV-2 seropositivity is high^10^^,^^11^ and most of the world's 39 million PLWH live, many not yet on antiretroviral therapy (ART).^12^

In CoVPN 3008 (Ubuntu), we sought to determine the relative risk of COVID-19 and severe COVID-19 associated with hybrid versus vaccine immunity among PLWH within six months of completing vaccinations with mRNA-1273, and to assess the safety and tolerability of the vaccine among PLWH. Secondary objectives included similar analyses in PLWoH, and characterising SARS-CoV-2 viral persistence within the cohort.

## Methods

### Study design

This multicentre, multi-stage study of COVID-19 mRNA vaccines enrolled participants at 47 clinical research sites in seven East and Southern African countries: Botswana, Eswatini, Kenya, Malawi, South Africa, Uganda, and Zambia. Study participants received the first vaccination between December 2, 2021, and September 9, 2022.

The initial six months of the study (Part A) was a prospective cohort study comparing hybrid versus vaccine immunity involving the administration of one or two doses of mRNA-1273, a monovalent mRNA vaccine encoding the spike protein of SARS-CoV-2 strain WA1, based on baseline serostatus. Part B of the study, which compares the relative efficacy of a randomly assigned month 6 vaccination with mRNA-1273 or mRNA-1273.222 (a bivalent mRNA vaccine encoding the spike proteins of both WA1 and BA.4/5 strains), will not be discussed here (Fig. S1A).

### Ethics

The study was conducted in accordance with the principles of the Declaration of Helsinki, the International Council for Harmonisation of Technical Requirements for Pharmaceuticals for Human Use Good Clinical Practice (ICH-GCP) guidelines, and all applicable local regulations. The study protocol was reviewed and approved by the relevant regulatory authorities and institutional research ethics committees in each participating country. Written informed consent was obtained from all participants prior to enrolment. Study oversight included an independent data and safety monitoring board to ensure participant welfare and the integrity of study outcomes. The study is registered on ClinicalTrials.gov (NCT05168813).

### Participants

The study enrolled adults aged ≥18 years living with HIV and/or with other comorbidities associated with severe COVID-19 (e.g., chronic lung disease, diabetes, obesity) based on U.S. Centers for Disease Control and Prevention (CDC) criteria.^13^ Individuals who previously received COVID-19 vaccines were excluded, but there were no exclusions for pregnancy, HIV viral load, CD4 count, or ART use (see Table S1 for eligibility criteria).

### Procedures

**Fig. 1:**
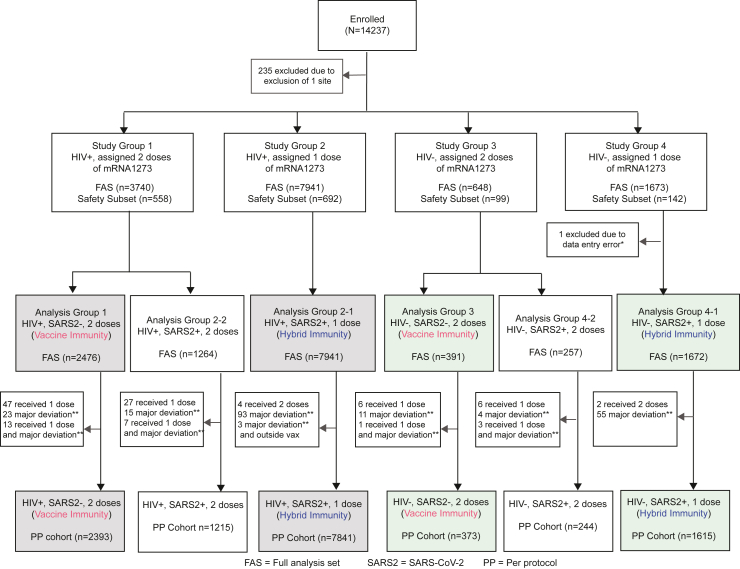
**CONSORT diagram. Analysis populations**. Enrolled participants were assigned to study groups based on HIV status and anti-spike SARS-CoV-2 point-of-care serology (POC anti-S) status. Participants were assigned to receive two doses of mRNA-1273 if their baseline POC anti-S was negative (vaccine immunity) and assigned to receive one dose of mRNA-1273 if their baseline POC anti-S was positive (hybrid immunity). The Full Analysis Set (FAS) consisted of participants who were enrolled and received at least one dose of mRNA-1273. The Safety Subset consisted of a simple random sample of the FAS within each of the four study groups. Six analysis groups (AGs), including the hybrid and vaccine immunity groups, were defined based on study group assignment and the overall SARS-CoV-2 status at baseline. The overall SARS-CoV-2 status was considered positive unless baseline nasal swab NAAT, POC anti-S, and central lab anti-nucleoprotein serology (anti-NP) were all negative. AGs are annotated in terms of HIV status, overall SARS-CoV-2 status, and the number of vaccine doses assigned. Specifically, AG1 represents people living with HIV (PLWH), overall SARS-CoV-2 status negative, and assigned 2 doses (PLWH, vaccine immunity). AG2-1 represents PLWH, overall SARS-CoV-2 status positive (POC anti-S positive), and assigned 1 dose (PLWH, hybrid immunity). AG2-2 represents PLWH, overall SARS-CoV-2 status positive (POC anti-S negative but anti-NP or NAAT positive) and assigned 2 doses. AG3 represents HIV-negative, SARS-CoV-2 status negative, and assigned 2 doses (HIV-negative, vaccine immunity). AG4-1 represents people living without HIV (PLWoH), overall SARS-CoV-2 status positive (POC anti-S positive), and assigned 1 dose (PLWoH, hybrid immunity). AG4-2 represents PLWoH, overall SARS-CoV-2 status positive (POC anti-S negative but anti-NP or NAAT positive) and assigned 2 doses. The Per-Protocol (PP) cohort consisted of participants in the FAS who received the intended number of pre-month 6 vaccination doses: two doses (for POC anti-S negative participants) or one dose (for POC anti-S positive participants), with no major protocol deviations. The data cut-off for the described safety and efficacy analyses occurred on March 31, 2023, 14 days after the last month 6 vaccination visit in Part A of the study. All COVID-19 and severe COVID-19 endpoints were based on data by this date, except one COVID-19 endpoint with symptom data collected through April 10, 2023. SARS2, SARS-CoV-2. ∗Participant anti-Spike SARS-CoV-2 value was negative in the site source data but was entered in the Case Report Form as positive. ∗∗Out of visit-window vaccination was not considered a major deviation.

Participants underwent baseline HIV antibody testing, and among PLWH, a CD4 count and HIV viral load were collected. All participants had a point-of-care SARS-CoV-2 anti-spike serology test (POC anti-S) (Assure Ecotest, Assure Tech, Hangzhou, China), a central laboratory anti-nucleoprotein SARS-CoV-2 serology test (anti-NP) (Abbott SARS-CoV-2 IgG, Abbott, Chicago, IL, USA), and a nasal swab SARS-CoV-2 nucleic acid amplification test (NAAT) taken at baseline. Baseline HIV status and the POC anti-S result were used to assign participants into four study groups (Fig. 1).

All participants received a 100-mcg mRNA-1273 vaccination into the deltoid muscle at enrolment. Baseline POC anti-S-positive participants received this single dose, whereas baseline POC anti-S-negative participants also received a second dose at month 1 (Fig. S1A). Participants were observed for at least 15 min post-vaccination. Vaccine storage, preparation, and administration followed manufacturer's specifications.

Participants provided nasal swabs for SARS-CoV-2 NAAT before each vaccination and at month 6. Participants were trained to self-monitor for prespecified COVID-19 symptoms at home, and sites also contacted participants every two weeks to assess for symptoms. COVID-19 symptoms prompted a clinic visit for further assessment and nasal swab testing. Participants testing positive for SARS-CoV-2 self-monitored symptoms daily, received monitoring calls every other day, and had additional nasal swabs taken fortnightly until testing negative (Fig. S1B). Positive swabs were sent for central laboratory testing, including SARS-CoV-2 whole genome sequencing using the MiSeq or NextSeq platforms (Illumina, Inc., San Diego, USA).

### Outcomes

#### Safety assessments

Solicited local and systemic adverse events (AEs) and unsolicited AEs were collected in a Safety Subset for 7 and 28 days after vaccination, respectively. The Safety Subset constituted participants from nearly all study sites, with contributions proportionate to site enrolment targets, and distributed across study groups. Serious AEs (SAEs) were captured for all participants throughout follow-up. All AEs were graded according to the Division of AIDS Table for Grading the Severity of Adult and Pediatric Adverse Events.^14^ PLWH received counselling, clinical and laboratory monitoring and, if required, were referred for ART and HIV care. Confirmed COVID-19 cases were closely monitored and managed as per standard-of-care. Pregnancy outcomes were followed up even after the participant's study exit.

#### Efficacy endpoint definitions

The primary endpoints were the first occurrence of COVID-19 or severe COVID-19 with onset at least 1 day after the enrolment vaccination until the month 6 visit among PLWH (Table S2). Using CDC criteria, COVID-19 was defined as one NAAT-positive nasal swab within 14 days of the onset of at least one systemic symptom (fever ≥38 °C, chills, myalgia, headache, sore throat, new loss of taste or smell), or at least one respiratory sign/symptom (cough, shortness of breath or difficulty breathing), or clinical or radiographical evidence of pneumonia. For completeness, we also assessed COVID-19 using the COVE trial definition.^1^ Severe COVID-19 cases required a laboratory diagnosis; at least one sign, symptom, or other evidence of severe disease (e.g., respiratory failure, shock, intensive care unit [ICU] admission); and were adjudicated by an independent committee. Considering baseline POC anti-S-negative participants had an additional nasal swab NAAT due to their month 1 vaccination visit compared to baseline POC anti-S-positive participants, different endpoints were derived to include or exclude NAAT testing results at month 1 to ensure comparable assessments of COVID-19 risk between groups.

#### Persistent infection and reinfection definitions

Participants were considered to have persistent SARS-CoV-2 NAAT positivity if they had positive swabs lasting ≥50 days during a single infection. A single infection was assumed unless the participant had a positive NAAT following either a single negative NAAT by ≥90 days or two consecutive negative NAATs over any time interval, in which case it was considered a reinfection.

### Statistical analysis

#### Sample size calculations

The sample size was determined to evaluate the safety objective and compare the risk of COVID-19 between the hybrid and vaccine immunity groups among PLWH. Regarding safety, with the planned enrolment cap of 15,600 participants, if no related safety event occurred, the upper limit of the 95% confidence interval (CI) for the true event rate would be 0.02%. For the 1500 participants in the Safety Subset, if no related AE occurred, the upper limit of the 95% CI for the true event rate would be 0.2%. Regarding the comparison of COVID-19 outcomes, there was >90% power to detect a relative risk of 2.0 between Study Group 1 (PLWH, POC anti-S negative) and Study Group 2 (PLWH, POC anti-S positive) against the null hypothesis H_0_: RR = 1.0 with ≥125 endpoints between enrolment and month 6 (See details in Study Protocol).

#### Baseline SARS-CoV-2 status

In addition to the baseline POC anti-S results used pragmatically in assigning study groups at enrolment, which in turn determined vaccination assignments, a baseline overall SARS-CoV-2 status was used in analyses and was based on results from POC anti-S as well as anti-NP and nasal swab NAAT. If any of these baseline tests were positive, overall SARS-CoV-2 status was positive. All participants had POC anti-S results and imputations were performed for missing data in NAAT and anti-NP results (See Supplementary Materials).

#### Analysis groups

Six analysis groups (AGs) were defined based on the overall baseline SARS-CoV-2 status and the number of vaccine doses received (Fig. 1). AG1 and AG2-1 included PLWH who were either overall baseline SARS-CoV-2 negative and received 2 doses (vaccine immunity) or overall baseline SARS-CoV-2 positive and received 1 dose (hybrid immunity), respectively. AG3 and AG4-1 were similar to AG1 and AG2-1, except were PLWoH with other comorbidities. The primary efficacy analysis compared the risk of COVID-19 and severe COVID-19 among PLWH with hybrid versus vaccine immunity (AG1 versus AG2-1). The secondary efficacy analysis pooled PLWH and PLWoH, and exploratory analyses included assessment of PLWoH alone (AG3 and AG4-1).

The Full Analysis Set (FAS) included participants who received at least one vaccination. The per-protocol (PP) cohort included FAS participants who received all planned vaccinations with no major protocol deviations. Efficacy analyses of COVID-19 were performed with both FAS (counting events starting 1 day after enrolment) and PP cohort (counting events starting 14 days after the last vaccination), using the CDC and COVE case definitions, up to March 31, 2023, 14 days after the last month 6 visit.

Seven additional exploratory comparisons were conducted by SARS-CoV-2 status and/or HIV status (See Supplementary Methods).

#### Analysis methods

Two statistical methods were used to assess the association between baseline status and outcome: calendar-time-scale Cox proportional-hazards regression methods, and analysis of counterfactual cumulative incidence over time.^15^ As the comparison groups were not randomised, covariate-adjusted analyses were performed. For both the Cox regression analysis and the cumulative incidence analysis of COVID-19 (using either the CDC or COVE case definition), two covariate adjustment strategies were considered. In the first strategy for primary analyses, adjustments were performed for variables expected to affect both risk of future COVID-19 and evidence of prior SARS-CoV-2 exposure,^16^, ^17^, ^18^ including region of enrolment (South Africa versus other African countries), period of enrolment (1–3 months, 4–6 months, or >6 months after study launch), age (> vs ≤40 years), sex assigned at birth (female versus male), body mass index (> vs ≤25 kg/m^2^), prior or active tuberculosis (yes versus no), CD4 counts (≤ vs >500 cells/μl), and detectable HIV viral load (yes versus no).

We estimated the counterfactual cumulative incidence using an extension of augmented inverse probability weighting that is equipped for right-censored survival data.^15^ This approach requires estimation of the conditional probabilities of survival and censoring, given prior SARS-CoV-2 status and the covariates, as well as the conditional probability of prior SARS-CoV-2, given the covariates. The conditional probability of survival and the conditional probability of censoring was estimated using Cox proportional-hazards regression, and the probability of prior SARS-CoV-2 infection was estimated using logistic regression. In the second strategy, a sensitivity analysis, continuous variables used in the previous approach were no longer dichotomised, and more flexible covariate adjustment methods, which made fewer assumptions about the data-generating mechanism, were used. In the Cox regression analysis approach, adjustments for continuous variables were made using cubic splines. The Cox proportional assumption was evaluated based on the scaled Schoenfeld residuals.^19^ In the cumulative incidence approach, each of the nuisance parameters was estimated using the SuperLearner, a type of ensemble learning algorithm.^20^ No covariate adjustment was performed for analyses involving the severe COVID-19 outcome or the post-baseline NAAT positive outcome due to a low number of endpoints.

### Role of the funding source

The views expressed in this article are those of the authors and do not necessarily represent the official position of the U.S. National Institute of Allergy and Infectious Diseases.

## Results

### Study population

Of 14,237 participants, 14,002 (83% PLWH) were included in the FAS (Table S3). The median follow-up was 169 days, and was similar across analysis groups, with 96.8% completing the 6-month follow-up (Fig. S2). Among 11,681 PLWH, the median age was 39 years (interquartile range [IQR] 33–46), 77.0% were female sex at birth, and 68.0% were POC anti-S positive. The median CD4 count was 635 cells/μl (IQR 423–866), 6.6% had a CD4 count <200 cells/μl, 18.5% had HIV viraemia (≥50 copies/ml), and 15.6% were not on ART (Table 1). The Safety Subset had similar baseline characteristics to the FAS (Table S4). Among PLWoH, the most common comorbidities determining enrolment were obesity (27.6%), hypertension (23.1%), diabetes mellitus (7.1%), and more than half (65.4%) had a smoking history (Table S5).

**Table 1:** Demographic and clinical characteristics at baseline by the six analysis groups.

Characteristic	Study group 1		Study group 2	Study group 3		Study group 4	Total N = 14,001
	Analysis Group 1 N = 2476	Analysis Group 2–2 N = 1264	Analysis Group 2–1 N = 7941	Analysis Group 3 N = 391	Analysis Group 4–2 N = 257	Analysis Group 4–1 N = 1672	
	HIV+, SARS2−, 2d	HIV+, SARS2+, 2d	HIV+, SARS2+, 1d	HIV−, SARS2−, 2d	HIV−, SARS2+, 2d	HIV−, SARS2+, 1d	
	PLWH Vaccine immunity		PLWH Hybrid immunity	HIV-negative Vaccine immunity		HIV-negative Hybrid immunity	
Point-of-care anti-spike serology test result	Negative	Negative	Positive	Negative	Negative	Positive	
Number of vaccine doses assigned	2	2	1	2	2	1	
Received vaccine doses as assigned	2416 (97.6%)	1230 (97.3%)	7937 (99.9%)	385 (98.5%)	248 (96.5%)	1671 (99.9%)	13,887 (99.2%)
SARS-CoV-2 NAAT result - N (%)							
Negative	2461 (99.4%)	973 (77.0%)	7583 (95.5%)	386 (98.7%)	213 (82.9%)	1592 (95.2%)	13,208 (94.3%)
Positive	0 (0.0%)	283 (22.4%)	340 (4.3%)	0 (0.0%)	44 (17.1%)	76 (4.5%)	743 (5.3%)
Missing	15 (0.6%)	8 (0.6%)	18 (0.2%)	5 (1.3%)	0 (0.0%)	4 (0.2%)	50 (0.4%)
Central lab anti-nucleoprotein test result - N (%)							
Negative	2471 (99.8%)	114 (9.0%)	3304 (41.6%)	389 (99.5%)	17 (6.6%)	600 (35.9%)	6895 (49.2%)
Positive	0 (0.0%)	1150 (91.0%)	4622 (58.2%)	0 (0.0%)	240 (93.4%)	1068 (63.9%)	7080 (50.6%)
Missing	5 (0.2%)	0 (0.0%)	15 (0.2%)	2 (0.5%)	0 (0.0%)	4 (0.2%)	26 (0.2%)
Overall SARS-CoV-2 status	Negative	Positive	Positive	Negative	Positive	Positive	
Sex assigned at birth - N (%)							
Male	800 (32.3%)	353 (27.9%)	1534 (19.3%)	291 (74.4%)	149 (58.0%)	763 (45.6%)	3890 (27.8%)
Female	1676 (67.7%)	911 (72.1%)	6407 (80.7%)	100 (25.6%)	108 (42.0%)	909 (54.4%)	10,111 (72.2%)
Median age (range) - years	39.0 (18.0, 76.0)	40.0 (18.0, 73.0)	39.0 (18.0, 79.0)	34.0 (19.0, 84.0)	39.0 (18.0, 82.0)	34.0 (18.0, 86.0)	39.0 (18.0, 86.0)
Age category - N (%)							
≤40 years	1388 (56.1%)	681 (53.9%)	4484 (56.5%)	260 (66.5%)	136 (52.9%)	1099 (65.7%)	8048 (57.5%)
>40 years	1088 (43.9%)	583 (46.1%)	3457 (43.5%)	131 (33.5%)	121 (47.1%)	573 (34.3%)	5953 (42.5%)
Active tuberculosis							
Yes	32 (1.3%)	12 (0.9%)	77 (1.0%)	3 (0.8%)	2 (0.8%)	16 (1.0%)	142 (1.0%)
No	2386 (96.4%)	1229 (97.2%)	7655 (96.4%)	377 (96.4%)	251 (97.7%)	1623 (97.1%)	13,521 (96.6%)
Missing	58 (2.3%)	23 (1.8%)	209 (2.6%)	11 (2.8%)	4 (1.6%)	33 (2.0%)	338 (2.4%)
History of tuberculosis							
Yes	363 (14.7%)	205 (16.2%)	1073 (13.5%)	15 (3.8%)	12 (4.7%)	75 (4.5%)	1743 (12.4%)
No	2055 (83.0%)	1036 (82.0%)	6659 (83.9%)	365 (93.4%)	241 (93.8%)	1564 (93.5%)	11,920 (85.1%)
Missing	58 (2.3%)	23 (1.8%)	209 (2.6%)	11 (2.8%)	4 (1.6%)	33 (2.0%)	338 (2.4%)
CD4 count^a^ (cells/μl) - N (%)							
<200	285 (11.5%)	87 (6.9%)	397 (5.0%)	–	–	–	769 (6.6%)
200 to <350	340 (13.7%)	158 (12.5%)	720 (9.1%)	–	–	–	1218 (10.4%)
350 to <500	394 (15.9%)	232 (18.4%)	1145 (14.4%)	–	–	–	1771 (15.2%)
≥500	1330 (53.7%)	735 (58.1%)	5341 (67.3%)	–	–	–	7406 (63.4%)
Missing	127 (5.1%)	52 (4.1%)	338 (4.3%)	–	–	–	517 (4.4%)
Median CD4 count^a^ (IQR) - cells/μl	549.0 (336.0, 791.0)	593.0 (393.8, 827.0)	664.0 (458.5, 889.0)	–	–	–	635.0 (423.0, 866.0)
HIV viral load^a^ (copies/mL) - N (%)							
<50	1676 (67.7%)	936 (74.1%)	6175 (77.8%)	–	–	–	8787 (75.2%)
≥50	637 (25.7%)	251 (19.9%)	1269 (16.0%)	–	–	–	2157 (18.5%)
Missing	163 (6.6%)	77 (6.1%)	497 (6.3%)	–	–	–	737 (6.3%)
Median HIV-1 viral load^a^ (IQR) - copies/mL	108 (40, 5192)	60 (40, 1509)	60 (40, 1064)	–	–	–	61 (40, 1741)
ART status^a^							
On ART	2110 (85.2%)	1084 (85.8%)	6670 (84.0%)	–	–	–	9864 (84.4%)
Not on ART	366 (14.8%)	180 (14.2%)	1271 (16.0%)	–	–	–	1817 (15.6%)
Missing	0 (0.0%)	0 (0.0%)	0 (0.0%)	–	–	–	0 (0.0%)

The overall SARS-CoV-2 status was defined as positive unless baseline nasal swab SARS-CoV-2 NAAT, point-of-care anti-spike serology (POC anti-S), and central lab anti-nucleoprotein serology (anti-NP) were all negative. Prior to this assignment, missing data were imputed based on the empirical conditional probabilities observed in the study population (Supplementary Materials). Analysis groups (AG) were based on baseline HIV status, overall SARS-CoV-2 status, and the number of vaccinations assigned. Specifically, AG1 represents people living with HIV (PLWH), overall SARS-CoV-2 status negative and assigned 2 doses. AG2-1 represents PLWH, overall SARS-CoV-2 status positive (POC anti-S positive) and assigned 1 dose. AG2-2 represents PLWH, overall SARS-CoV-2 status positive (POC anti-S negative but anti-NP or NAAT positive) and assigned 2 doses. AG3 represents people living without HIV (PLWoH), SARS-CoV-2 status negative, and assigned 2 doses. AG4-1 represents PLWoH, overall SARS-CoV-2 status positive (POC anti-S positive) and assigned 1 dose. AG4-2 represents PLWoH, overall SARS-CoV-2 status positive (POC anti-S negative but anti-NP or NAAT positive) and assigned 2 doses.

NAAT, nucleic acid amplification test. SARS2, SARS-CoV-2. 1d, one vaccine dose. 2d, two vaccine doses. ART, antiretroviral therapy.

a Only measured among PLWH. The denominator for calculating percentages in the ‘Total’ column is the number of participants in study groups 1 and 2 (N = 11,681).

### Safety

The most common solicited AEs after first or second vaccination among the 1491 Safety Subset participants were pain or tenderness at the injection site (26.7%), headache (20.4%), and malaise (20.3%) (Fig. S3). Severe solicited AEs were rare (0.8% of participants after the first and 1.1% after the second vaccination), and none were life-threatening or fatal (Tables S6 and S7). Unsolicited AEs were observed among 6.1% of Safety Subset participants, with 0.3% deemed vaccination-related (Tables S8 and S9). Among FAS participants, there was 1 related SAE of grade 3 angioedema. The most common unrelated SAEs were pneumonia (0.1%) and pulmonary tuberculosis (0.1%); (Table S10). There were 119 reported pregnancy outcomes with no congenital abnormalities, and there were no statistically significant differences in spontaneous abortions, stillbirths, or premature births between study groups (Table S11).

### Association of hybrid versus vaccine immunity with COVID-19 and severe COVID-19

Overall, 358 COVID-19 cases (309 among PLWH), including 15 severe cases (11 among PLWH), accrued through month 6 among FAS participants (Table S12). There was a single COVID-19-related ICU admission and no deaths.

**Fig. 2:**
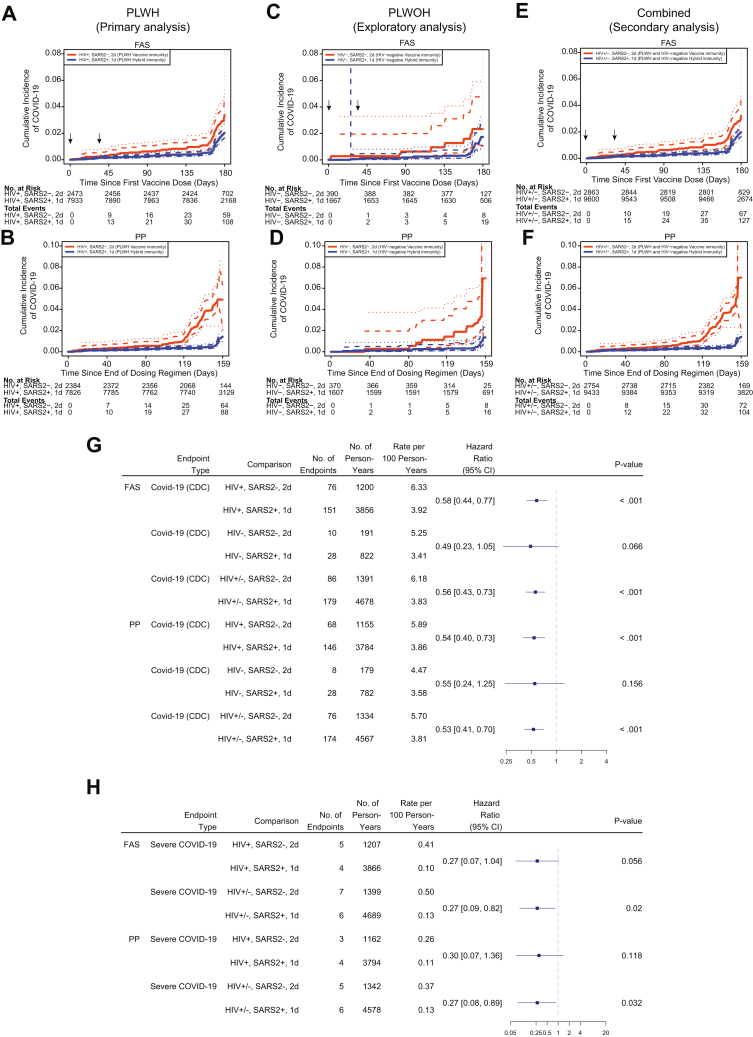
**Association of hybrid versus vaccine immunity against COVID-19 in PLWH, PLWoH, and all participants**. Shown is the cumulative incidence of COVID-19 events based on the CDC case definition among PLWH in the Full Analysis Set (FAS) (**Panel A**), among PLWH in the Per-Protocol (PP) cohort (**Panel B**), among PLWoH in FAS (**Panel C**), among PLWoH in PP cohort (**Panel D**), among all PLWH and PLWoH in FAS (**Panel E**), and among all PLWH and PLWoH in PP cohort (**Panel F**) for the vaccine immunity group (orange) and the hybrid immunity group (blue), counting events starting 1 day after the first vaccination in FAS, and 14 days after the last vaccination in the PP cohort. The number of COVID-19 events shown in this figure contains a subset of the total number of 358 events based on the CDC case definition because only the hybrid immunity and vaccine immunity groups are included, and two more analysis groups AG2-2 and AG4-2 are excluded. Comparisons including other subgroups are in the Supplementary Material. To ensure stability of estimated standard error, pointwise, and simultaneous intervals are reported starting 14 days after the first vaccination in FAS, and 27 days after the last vaccination in the PP cohort. The vaccine immunity group represents participants who were overall SARS-CoV-2 negative and received 2 doses of mRNA-1273 at enrolment and month 1, and the hybrid immunity group represents participants who were overall SARS-CoV-2 positive and received 1 dose of mRNA-1273 at enrolment. Arrows along the x-axis in Panels A, C, and E indicate enrolment and month 1 vaccination visits, and the tick marks in the curves in Panels A–F indicate censored data. Forest-plot shows hazard ratios of COVID-19 (**Panel G**) and of severe COVID-19 (**Panel H**) for the comparison of hybrid immunity versus vaccine immunity in PLWH, PLWoH (only for COVID-19 events), and in all participants in FAS and PP cohort groups. SARS2, SARS-CoV-2. 1d, one vaccine dose. 2d, two vaccine doses.

Among PLWH, the hybrid immunity group had a lower cumulative incidence of both endpoints compared to the vaccine immunity group, 2.02% (95% CI 1.61–2.44) vs 3.40% (95% CI 2.30–4.49) for COVID-19 (Fig. 2A), and 0.048% (95% CI 0.00–0.10) vs 0.32% (95% CI 0.59–0.63) for severe COVID-19 (Fig. S4). Endpoint accumulation trends corresponded with the scheduled NAAT at month 6 (Fig. S5). The hybrid immunity group had a 42% lower adjusted hazard rate of COVID-19 (hazard ratio [HR] 0.58; 95% CI 0.44–0.77; p < 0.001), and a 73% lower hazard rate of severe COVID-19 (HR 0.27; 95% CI 0.07–1.04; p = 0.056) (Fig. 2G and H).

Even more pronounced differences between the hybrid and vaccine immunity groups were observed in the PP analysis (Fig. 2B, D, and G) that included a total of 533 post-enrolment SARS-CoV-2 infections among PLWH. The cumulative incidence in the hybrid immunity group was 3.90% (95% CI 3.30–4.49) vs 7.77% (95% CI 6.20–9.23) in the vaccine immunity group (Fig. S6). Similar results were observed using the more restrictive COVE case definition (Table S13, Figs. S7 and S8), using both the Cox model and the cumulative incidence approaches (Figs. S9–S12), and in sensitivity analyses using alternative covariate adjustment strategies (Figs. S13 and 14).

In secondary analyses of FAS participants pooling PLWH and PLWoH, results mirrored those observed with PLWH alone. The cumulative incidence of COVID-19 was 1.97% (95% CI 1.60–2.34) in the hybrid immunity group versus 3.21% (95% CI 2.25–4.20) in the vaccine immunity group (Fig. 2E, Fig. S15). For severe COVID-19, the cumulative incidence was 0.06% (95% CI 0.01–0.12) vs 0.35% (95% CI 0.06–0.63), respectively (Fig. S16), with slightly more pronounced differences in the PP cohort (Fig. 2F). For the hybrid immunity group, the covariate-adjusted hazard rate of COVID-19 was 44% lower (HR 0.56; 95% CI 0.43–0.73; p < 0.001), and the hazard rate of severe COVID-19 was 73% lower (HR 0.27; 95% CI 0.09–0.82; p = 0.02) (Fig. 2G and H). Similar results were observed in both the FAS and PP cohorts using the COVE case definition (Figs. S17 and S18).

As with PLWH and the pooled populations, among PLWoH a lower risk of COVID-19 in the hybrid immunity group was observed, although with lower statistical power (HR 0.49; 95% CI 0.23–1.05; p = 0.066) (Fig. 2C and G). Moreover, in exploratory analyses of the FAS and PP cohort, we found that the cumulative incidence estimates of COVID-19 were comparable between PLWH and PLWoH (Figs. S19–S33).

**Fig. 3:**
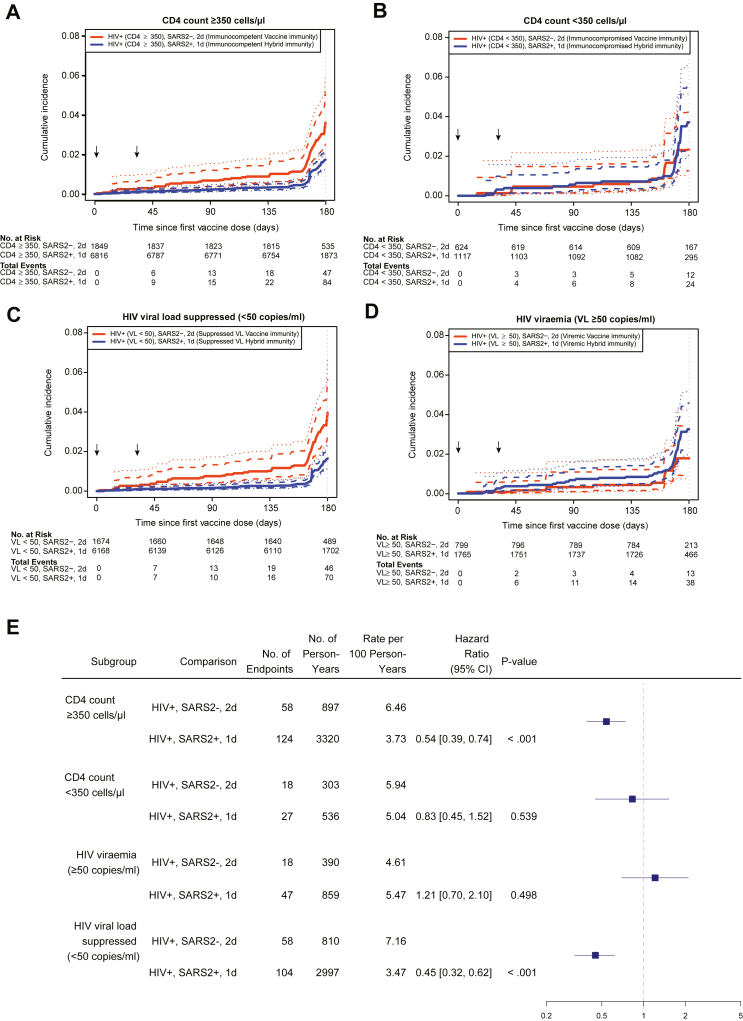
**Association of hybrid versus vaccine immunity against COVID-19 in people living with HIV, within strata defined by HIV immunocompetence and HIV viraemia**. Shown is the cumulative incidence of COVID-19 events based on the CDC case definition among people living with HIV (PLWH) in the Full Analysis Set (FAS) with CD4 count ≥350 cells/μl (**Panel A**), with CD4 count <350 cells/μl (**Panel B**), with HIV viral load <50 copies/mL (**Panel C**), and with HIV viral load ≥50 copies/mL (**Panel D**) for the vaccine immunity group (orange) and the hybrid immunity group (blue), counting events starting 1 day after the first vaccination. To ensure stability of estimated standard error, pointwise and simultaneous intervals are reported starting 14 days after the first vaccination. The vaccine immunity group represents participants who were overall SARS-CoV-2 negative and received 2 doses of mRNA-1273 at enrolment and month 1, and the hybrid immunity group represents participants who were overall SARS-CoV-2 positive and received 1 dose of mRNA-1273 at enrolment. Arrows along the x-axis in Panels A–D indicate enrolment and month 1 vaccination visits. Forest-plot shows hazard ratios of CDC case definition COVID-19 for the comparison of hybrid versus vaccine immunity in PLWH in the FAS within each CD4 count and viral load stratum (**Panel E**). SARS2, SARS-CoV-2. 1d, one vaccine dose. 2d, two vaccine doses.

In a post-hoc analysis, comparisons of COVID-19 incidence among PLWH between hybrid and vaccine immunity groups were made within strata defined by CD4 count (≥ or <350 cells/μl) and viraemia (HIV viral load ≥ or <50 copies/mL). The advantage of hybrid immunity over vaccine immunity was preserved among those with a CD4 count ≥350 cells/μl (HR 0.54; 95% CI 0.39–0.74; p < 0.001), or viral suppression (HR 0.45; 95% 0.32–0.62; p < 0.001). However, when comparing the hybrid and vaccine immunity groups, there was no difference in COVID-19 incidence among those with CD4 counts <350 cells/μl (HR 0.83; 95% CI 0.45–1.52; p = 0.539) or those with HIV viraemia (HR 1.21; 95% CI 0.70–2.1; p = 0.498) (Fig. 3). Of note, in the hybrid immunity group, we found numerically higher, but not statistically significant higher rates of COVID-19 endpoints among PLWH with CD4 counts <350 cells/μl compared to ≥350 cells/μl (HR 1.43; 95% CI 0.93–2.19; p = 0.107), and statistically significant higher rates among PLWH with HIV viraemia compared to those with viral suppression (HR 1.8; 95% CI 1.27–2.56; p = 0.001) (Fig. S34).

### SARS-CoV-2 sequences and persistent infections

**Fig. 4:**
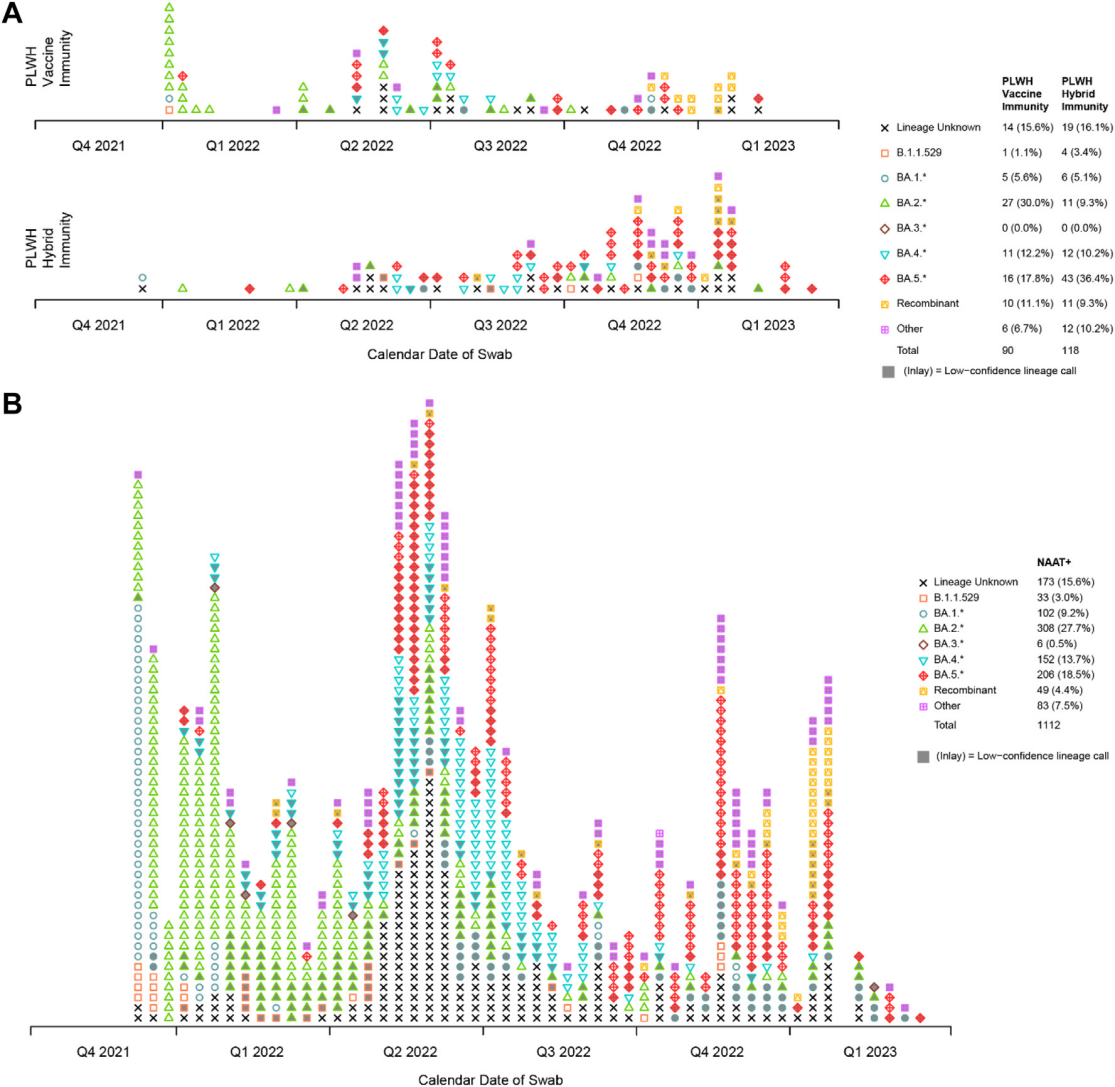
**Lineages and persistence of SARS-CoV-2 infections**. **Panel A** shows the lineages of SARS-CoV-2 associated with the diagnosis of COVID-19 endpoints based on the CDC endpoint definition over calendar time in the two primary comparison groups: People living with HIV (PLWH) vaccine immunity group (HIV+, SARS2−, 2d) and PLWH hybrid immunity group (HIV+, SARS2+, 1d). **Panel B** shows the lineages of SARS-CoV-2 associated with the first occurrence of all COVID-19 infections (positive NAAT tests) regardless of symptoms obtained at either baseline or post-baseline visits based on all sequences available at the time of the analysis. The viral lineage is illustrated by the colour and plot character. Lineage typing was performed with both PANGOLIN and NextClade, and the call with the highest confidence was selected (see Methods). Due to insufficient viral material, many sequences were either (i) unable to be obtained (indicated by “Lineage Unknown”); (ii) exhibited such a degree of missingness that they were incapable of being lineage typed (also indicated by “Lineage Unknown”); or (iii) obtained a lineage from at least one platform, but failed QC on both platforms. These latter sequences are regarded as having low-confidence lineage calls and are indicated in this figure by having their plot character inlaid with grey. Most sequences were lineage-typed within omicron, so these sequences are grouped in the figure by the basal omicron lineage (B.1.1.529), the five major omicron sub-lineages (BA.1 through BA.5; e.g., BE.7 is classified as “BA.5.∗”) or recombinant status (e.g., XBB). Non-omicron lineages, almost exclusively occurring among the low-confidence lineage calls, are classified as “Other”. Q, three-month quarter: Q1 (January–March), Q2 (April–June), Q3 (July–September), and Q4 (October–December). NAAT, nucleic acid amplification test. SARS2, SARS-CoV-2. 1d, one vaccine dose. 2d, two vaccine doses.

Almost all COVID-19 and first occurrences of positive NAAT results in participants, regardless of symptoms, were associated with the omicron lineage, with BA.1 and BA.2 dominating initially, followed by BA.4 and BA.5 (Fig. 4A and B). The geographic distribution is shown in Table S14. The subvariants associated with different endpoints are shown by analysis group in Figs. S35–S37, demonstrating patterns consistent with contemporaneous surveillance data.^21^

**Fig. 5:**
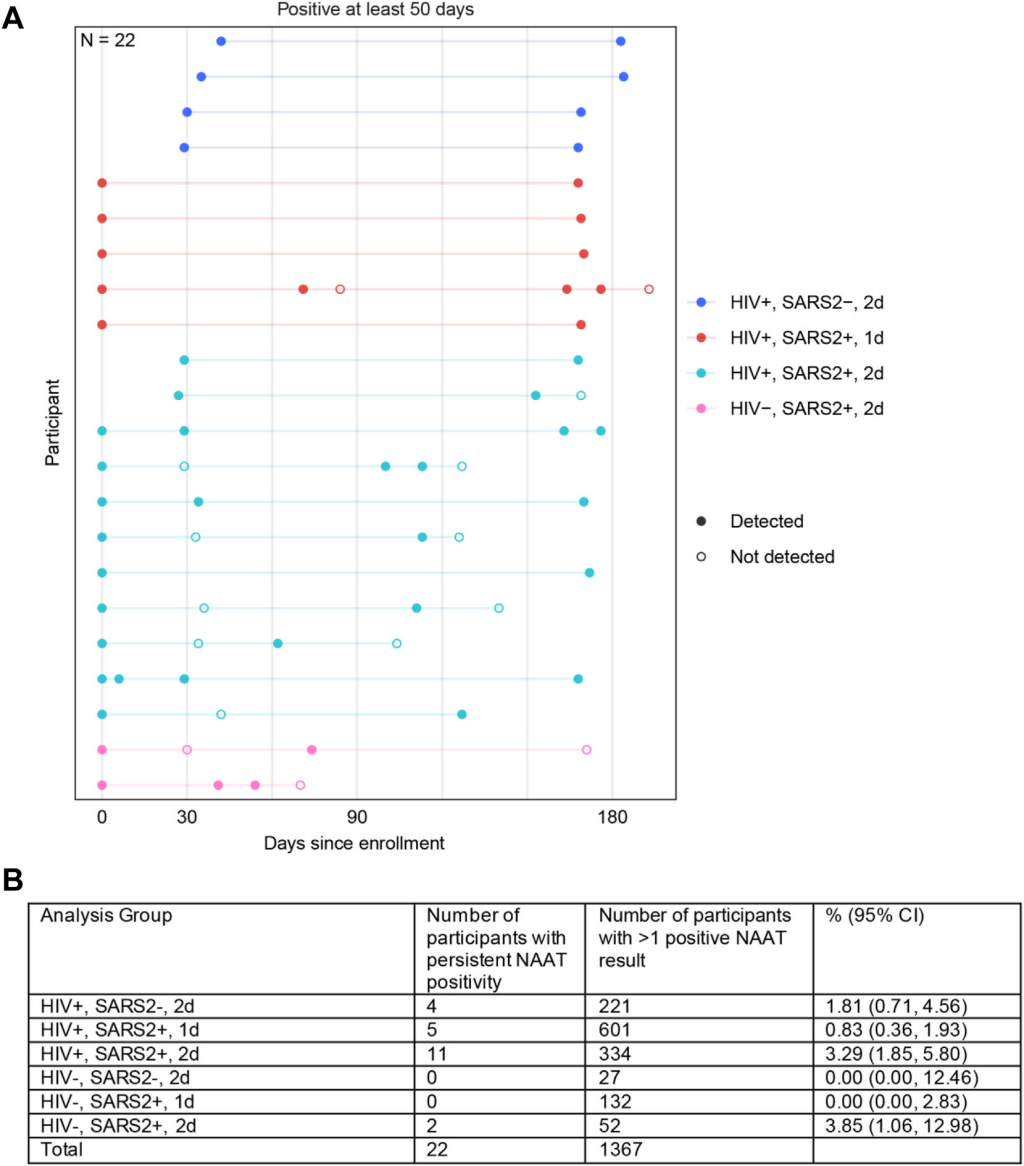
**Persistent SARS-CoV-2 infections. Panel A** shows the 22 participants who experienced persistent NAAT positivity for ≥50 days. **Panel B** shows the number and frequencies of persistent NAAT positivity (≥50 days) and total number of NAAT positive results by analysis group. NAAT, nucleic acid amplification test. SARS2, SARS-CoV-2. 1d, one vaccine dose. 2d, two vaccine doses.

Among FAS participants, 1367 (9.8%) had at least one NAAT positive swab, 743 at baseline and 624 during follow-up (Table S15), of which 1050 (76.8%) were asymptomatic infections. NAAT positivity ≥50 days was observed in 22 participants (including 20 PLWH). Among PLWH, persistent NAAT positivity represented 2.5% of infections compared to 1.4% among PLWoH (Table 2). These proportions were higher among PLWH with a history of tuberculosis at baseline versus those without (4.8% vs 2.2%), a detectable (≥50 copies/ml) versus undetectable HIV viral load (4.7% vs 1.7%), and a baseline CD4 count <200 vs ≥200 cells/μl (3.5% vs 2.4%). NAAT positivity persisting ≥100 days was observed in 19 participants, all PLWH. Fig. 5A and B shows the NAAT results of persistent infections by subgroups.

**Table 2:** The number and frequencies of persistent NAAT positivity (≥50 days) by baseline HIV status, CD4 count, HIV viral load, and tuberculosis status.

	All NAAT positive infections^a^ N (%)	Persistent NAAT positivity^b^ N (%)
**All participants**		
People living without HIV	2/211 (0.9)	2/142 (1.4)
People living with HIV	20/1156 (1.7)	20/802 (2.5)
**Among people living with HIV**		
History of TB status^c^		
No	14/939 (1.5)	14/647 (2.2)
Yes	6/190 (3.2)	6/136 (4.4)
HIV viral load (copies/mL)		
<50	10/861 (1.2)	10/588 (1.7)
≥50	10/295 (3.4)	10/214 (4.7)
CD4 count (cells/μl)		
<200	3/111 (2.7)	3/86 (3.5)
≥200	17/1045 (1.6)	17/716 (2.4)
Immunity status^d^		
Vaccine immunity (AG 1)	0/137 (0)	0/34 (0)
Hybrid immunity with 1 dose (AG 2.1)	5/601 (0.8)	5/379 (1.3)

a Among all baseline and post-baseline infections (i.e., participants with at least one NAAT positive test result regardless of symptomology).

b Among participants with evaluable NAAT test results at ≥2 time points.

c A total of 27 of the 1376 participants were missing baseline tuberculosis status among all infections, and 19 of the 94 participants among those with multiple NAAT results.

d For the vaccine immunity (AG1) group, NAAT test results were excluded if the first positive test was at the Month 1 visit because NAAT tests were not scheduled at this visit for the hybrid immunity (AG2.1) group.

## Discussion

In the CoVPN 3008 (Ubuntu) study, we found that among a diverse population of PLWH, a single vaccination in persons with prior SARS-CoV-2 infection was associated with an over 40% reduction in the risk of COVID-19 and an over 70% reduction in the risk of severe COVID-19 in the first six months after vaccination compared to individuals without prior infection who received two vaccinations. Similar findings were seen among enrolled PLWoH. However, in post-hoc analyses, this effect was not seen among PLWH with poorly controlled HIV. Furthermore, we identified a subgroup of PLWH with SARS-CoV-2 persistence for over three months. Ubuntu also provides additional evidence that COVID-19 mRNA vaccines are safe and well tolerated, including among PLWH and pregnant women. Pregnancy outcomes were similar to available population data^22^^,^^23^ and corroborated prior observational data supporting the safety of mRNA vaccines during pregnancy.^24^^,^^25^

Our findings of the superior efficacy of hybrid immunity to vaccine immunity are consistent with published population-based observational studies, including during omicron, but these studies largely excluded PLWH and mainly involved high-income countries.^26^, ^27^, ^28^ One large test-negative case-control study in Quebec, Canada, for example, found that compared to healthcare workers with no prior vaccination or infection, those without prior infection and two mRNA vaccinations had a 61% adjusted risk reduction of symptomatic COVID-19, compared to 81% for those with prior pre-omicron infection and one vaccination and 98% for those with prior BA.1 infection and one vaccination.^28^ In another test-negative, case-control study, this time in Qatar, which included individuals with prior infection alone, vaccination alone, and hybrid immunity, hybrid immunity from previous infection and a recent booster vaccination provided the strongest protection.^26^ Why we did not see a hybrid immunity benefit among participants with poorly controlled HIV may be partially related to impaired B-cell function and cytotoxic T-cell responses due to HIV-induced immunosuppression, which has been suggested by prior studies.^29^

It is important that our findings be interpreted within the appropriate clinical and public health context. The study does not compare the efficacy of one versus two mRNA vaccine doses. And while ethical considerations precluded including an unvaccinated comparator group, the hybrid immunity group showed a greater advantage over the vaccine immunity group in hazard ratio estimates within the PP cohort compared to the FAS cohort, suggesting that vaccination (not prior infection by itself) contributed to the protective advantage of hybrid immunity, underscoring the importance of vaccination. Moreover, the lack of benefit from hybrid immunity among individuals with poorly controlled HIV highlights the need to strengthen HIV care and suggests more frequent vaccinations may be needed to promote protection in immunosuppressed PLWH, as proposed by international guidelines^30^ and indicated by previous studies.^31^^,^^32^

While prior research including in southern Africa has consistently found less severe disease with omicron compared to prior waves,^33^^,^^34^ the very low rate of severe COVID-19 (0.1%) in Ubuntu is still notable considering enrolment was exclusively among individuals at risk for severe disease. Over 75% of infections in PLWH were asymptomatic and detected via scheduled swabbing, indicating the inadequacy of symptom-driven testing. While the reduced disease from omicron has clear public health benefit, the high proportion of subclinical omicron infections presents a challenge for surveillance and promotes inadvertent transmission.

We found that SARS-CoV-2 persistence was associated with 2.5% of omicron infections among PLWH, and this rose to 3.5–4.7% among those with HIV viraemia, low CD4 counts, or a history of tuberculosis. Whether this persistent NAAT positivity is associated with replication-competent, transmissible virus or mutational changes remains unclear. However, prior studies have suggested that long-term viral shedding may be infectious^35^^,^^36^ and linked to intra-host mutations associated with new variants of concern.^37^^,^^38^ Genomic sequencing of sequential isolates from our study is ongoing.

Our study had several strengths. Ubuntu is one of the largest prospective studies of a COVID-19 vaccine conducted exclusively in Africa. It uniquely enrolled a diverse population of predominantly PLWH, including pregnant individuals and those with low CD4 counts and HIV viraemia. Furthermore, it is among the largest studies investigating COVID-19 mRNA vaccines during the omicron outbreak and one of the few to compare hybrid immunity with vaccine immunity. Our findings on hybrid immunity are robust, supported by multiple analytic approaches, and remained consistent even when more restrictive criteria for COVID-19 endpoints were applied.

The study had some limitations. First, as noted earlier, the timing of the study precluded us from including an unvaccinated comparator group for ethical reasons. Therefore, it is challenging to infer how much vaccination contributed to either hybrid or vaccine immunity. Second, we enrolled a higher proportion of females than males. This is partly explained by the higher prevalence of HIV among women in our study settings. Third, the timing and lineages of prior infections, and the resulting quantitative SARS-CoV-2 antibody levels at enrolment, were unknown, so we were unable to assess if these factors influenced immunity and the differences in efficacy. Fourth, our study investigated the effect of hybrid immunity with mRNA-1273 vaccination; whether other COVID-19 vaccines, e.g., vector-based vaccines, have the same effect is not clear. Lastly, given that participants were designated to study groups rather than randomly assigned, the usual biases inherent in observational research—such as partial confounding and survival bias—may unduly complicate any causal interpretation.

In conclusion, we found that overall hybrid immunity was associated with a significantly lower risk of COVID-19 compared to vaccine immunity in a diverse population of PLWH vaccinated with mRNA-1273, although this benefit was not maintained among those with poorly controlled HIV. The vaccines were safe and well tolerated. Most infections in Ubuntu were subclinical, reflecting the lower disease burden with omicron and reinforcing the need for proactive surveillance strategies. Finally, we identified a subpopulation with persistent SARS-CoV-2 shedding, especially among participants with poorly controlled HIV. Given the significant global population of individuals with uncontrolled HIV who may act as a reservoir for emerging variants, it is imperative to reinforce HIV care, continue developing next-generation COVID-19 vaccines, and further study prolonged SARS-CoV-2 infections.

## Contributors

### Conceptualisation

Nigel Garrett, Asa Tapley, Sufia Dadabhai, Nyaradzo M. Mgodi, Guido Ferarri, Taraz Samandari, Zvavahera Chirenje, Peter James Elyanu, Joseph Makhema, Ethel Kamuti, Harriet Nuwagaba-Biribonwoha, M. Juliana McElrath, James G. Kublin, Linda-Gail Bekker, Peter B. Gilbert, Lawrence Corey, Glenda E. Gray, Yunda Huang, and Philip Kotze.

### Data curation

Nigel Garrett, Sufia Dadabhai, Azwidihwi Takalani, Erica Andersen-Nissen, Eduan Wilkinson, Richard Lessells, Tulio de Oliveira, Taraz Samandari, Zvavahera Chirenje, Peter James Elyanu, Joseph Makhema, Ethel Kamuti, Harriet Nuwagaba-Biribonwoha, Sharlaa Badal-Faesen, William Brumskine, Soritha Coetzer, Rodney Dawson, Sinead Delany-Moretlwe, Andreas Henri Diacon, Samantha Fry, Katherine Margaret Gill, Zaheer Ahmed Ebrahim Hoosain, Mina C. Hosseinipour, Mubiana Inambao, Craig Innes, Steve Innes, Dishiki Kalonji, Margaret Kasaro, Priya Kassim, Noel Kayange, William Kilembe, Fatima Laher, Moelo Malahleha, Vongane Louisa Maluleke, Grace Mboya, Kirsten McHarry, Essack Mitha, Kathryn Mngadi, Pamela Mda, Tumelo Moloantoa, Cissy Kityo Mutuluuza, Nivashnee Naicker, Vimla Naicker, Anusha Nana, Annet Nanvubya, Maphoshane Nchabeleng, Walter Otieno, Elsje Louise Potgieter, Disebo Potloane, Zelda Punt, Jamil Said, Yashna Singh, Mohammed Siddique Tayob Yacoob Vahed, Deo Ogema Wabwire, and Philip Kotze.

### Formal analysis

Aaron Hudson, Bo Zhang, Jessica Andriesen, Leigh H. Fisher, Jia Jin Kee, Craig A. Magaret, Peter B. Gilbert, and Yunda Huang. Jessica Andriesen, Peter B. Gilbert, Aaron Hudson, Bo Zhang, and Yunda Huang accessed and verified the data.

### Funding acquisition

Lawrence Corey, James G. Kublin, and Glenda E. Gray.

### Investigation

Nigel Garrett, Asa Tapley, Aaron Hudson, Sufia Dadabhai, Bo Zhang, Nyaradzo M. Mgodi, Jessica Andriesen, Azwidihwi Takalani, Leigh H. Fisher, Jia Jin Kee, Craig A. Magaret, Guido Ferarri, Eduan Wilkinson, Richard Lessells, Tulio de Oliveira, Laura Polakowski, Margaret Yacovone, Taraz Samandari, Zvavahera Chirenje, Peter James Elyanu, Joseph Makhema, Ethel Kamuti, Harriet Nuwagaba-Biribonwoha, Sharlaa Badal-Faesen, William Brumskine, Soritha Coetzer, Rodney Dawson, Sinead Delany-Moretlwe, Andreas Henri Diacon, Samantha Fry, Katherine Margaret Gill, Zaheer Ahmed Ebrahim Hoosain, Mina C. Hosseinipour, Mubiana Inambao, Craig Innes, Steve Innes, Dishiki Kalonji, Margaret Kasaro, Priya Kassim, Noel Kayange, William Kilembe, Fatima Laher, Moelo Malahleha, Vongane Louisa Maluleke, Grace Mboya, Kirsten McHarry, Essack Mitha, Kathryn Mngadi, Pamela Mda, Tumelo Moloantoa, Cissy Kityo Mutuluuza, Nivashnee Naicker, Vimla Naicker, Anusha Nana, Annet Nanvubya, Maphoshane Nchabeleng, Walter Otieno, Elsje Louise Potgieter, Disebo Potloane, Zelda Punt, Jamil Said, Yashna Singh, Mohammed Siddique Tayob, Yacoob Vahed, Deo Ogema Wabwire, Linda-Gail Bekker, Peter B. Gilbert, Lawrence Corey, Glenda E. Gray, Yunda Huang, and Philip Kotze.

### Methodology

Nigel Garrett, Asa Tapley, Aaron Hudson, Sufia Dadabhai, Bo Zhang, Nyaradzo M. Mgodi, Jessica Andriesen, Azwidihwi Takalani, Leigh H. Fisher, Jia Jin Kee, Craig A. Magaret, Manuel Villaran, John Hural, Erica Andersen-Nissen, Guido Ferarri, Richard Lessells, Jackline Odhiambo, Parth Shah, Laura Polakowski, Margaret Yacovone, Taraz Samandari, Zvavahera Chirenje, Peter James Elyanu, Joseph Makhema, Ethel Kamuti, Harriet Nuwagaba-Biribonwoha, James G. Kublin, Linda-Gail Bekker, Peter B. Gilbert, Lawrence Corey, Glenda E. Gray, Yunda Huang, and Philip Kotze.

### Project administration

Nigel Garrett, Asa Tapley, Sufia Dadabhai, Nyaradzo M. Mgodi, Jessica Andriesen, Azwidihwi Takalani, Manuel Villaran, John Hural, Erica Andersen-Nissen, Bert Le Roux, Richard Lessells, Jackline Odhiambo, Parth Shah, Laura Polakowski, Margaret Yacovone, Taraz Samandari, Zvavahera Chirenje, Peter James Elyanu, Joseph Makhema, Ethel Kamuti, Harriet Nuwagaba-Biribonwoha, Linda-Gail Bekker, Peter B. Gilbert, Lawrence Corey, Glenda E. Gray, Yunda Huang, and Philip Kotze.

### Resources

Nigel Garrett, Sufia Dadabhai, Azwidihwi Takalani, John Hural, Erica Andersen-Nissen, Tulio de Oliveira, Jackline Odhiambo, Parth Shah, Laura Polakowski, Margaret Yacovone, Taraz Samandari, Zvavahera Chirenje, Peter James Elyanu, Joseph Makhema, Ethel Kamuti, Harriet Nuwagaba-Biribonwoha, Sharlaa Badal-Faesen, William Brumskine, Soritha Coetzer, Rodney Dawson, Sinead Delany-Moretlwe, Andreas Henri Diacon, Samantha Fry, Katherine Margaret Gill, Zaheer Ahmed Ebrahim Hoosain, Mina C. Hosseinipour, Mubiana Inambao, Craig Innes, Steve Innes, Dishiki Kalonji, Margaret Kasaro, Priya Kassim, Noel Kayange, William Kilembe, Fatima Laher, Moelo Malahleha, Vongane Louisa Maluleke, Grace Mboya, Kirsten McHarry, Essack Mitha, Kathryn Mngadi, Pamela Mda, Tumelo Moloantoa, Cissy Kityo Mutuluuza, Nivashnee Naicker, Vimla Naicker, Anusha Nana, Annet Nanvubya, Maphoshane Nchabeleng, Walter Otieno, Elsje Louise Potgieter, Disebo Potloane, Zelda Punt, Jamil Said, Yashna Singh, Mohammed Siddique Tayob, Yacoob Vahed, Deo Ogema Wabwire, M. Juliana McElrath, James G. Kublin, Linda-Gail Bekker, Peter B. Gilbert, Lawrence Corey, Glenda E. Gray, Yunda Huang, and Philip Kotze.

### Software

Aaron Hudson, Bo Zhang, Jessica Andriesen, Leigh H. Fisher, Jia Jin Kee, Craig A. Magaret, Peter B. Gilbert, and Yunda Huang.

### Supervision

Nigel Garrett, Asa Tapley, Sufia Dadabhai, Nyaradzo M. Mgodi, Jessica Andriesen, Azwidihwi Takalani, Craig A. Magaret, John Hural, Erica Andersen-Nissen, Guido Ferarri, Bert Le Roux, Richard Lessells, Tulio de Oliveira, Jackline Odhiambo, Parth Shah, Laura Polakowski, Margaret Yacovone, Taraz Samandari, Zvavahera Chirenje, Peter James Elyanu, Joseph Makhema, Ethel Kamuti, Harriet Nuwagaba-Biribonwoha, M. Juliana McElrath, James G. Kublin, Linda-Gail Bekker, Peter B. Gilbert, Lawrence Corey, Glenda E. Gray, Yunda Huang, and Philip Kotze.

### Validation

Nigel Garrett, Asa Tapley, Aaron Hudson, Sufia Dadabhai, Bo Zhang, Nyaradzo M. Mgodi, Jessica Andriesen, Azwidihwi Takalani, Leigh H. Fisher, Jia Jin Kee, Craig A. Magaret, Guido Ferarri, Eduan Wilkinson, Richard Lessells, Tulio de Oliveira, Laura Polakowski, Margaret Yacovone, Taraz Samandari, Zvavahera Chirenje, Peter James Elyanu, Joseph Makhema, Ethel Kamuti, Harriet Nuwagaba-Biribonwoha, Peter B. Gilbert, Lawrence Corey, Glenda E. Gray, Yunda Huang, and Philip Kotze.

### Visualisation

Nigel Garrett, Asa Tapley, Aaron Hudson, Sufia Dadabhai, Bo Zhang, Nyaradzo M. Mgodi, Jessica Andriesen, Azwidihwi Takalani, Leigh H. Fisher, Jia Jin Kee, Craig A. Magaret, Maurine Miner, Eduan Wilkinson, Richard Lessells, Peter B. Gilbert, and Yunda Huang.

### Writing – original draft

Nigel Garrett and Asa Tapley.

### Writing – review & editing

Nigel Garrett, Asa Tapley, Aaron Hudson, Sufia Dadabhai, Bo Zhang, Nyaradzo M. Mgodi, Jessica Andriesen, Azwidihwi Takalani, Leigh H. Fisher, Jia Jin Kee, Craig A. Magaret, Manuel Villaran, John Hural, Erica Andersen-Nissen, Guido Ferarri, Maurine Miner, Bert Le Roux, Eduan Wilkinson, Richard Lessells, Tulio de Oliveira, Jackline Odhiambo, Parth Shah, Laura Polakowski, Margaret Yacovone, Taraz Samandari, Zvavahera Chirenje, Peter James Elyanu, Joseph Makhema, Ethel Kamuti, Harriet Nuwagaba-Biribonwoha, Sharlaa Badal-Faesen, William Brumskine, Soritha Coetzer, Rodney Dawson, Sinead Delany-Moretlwe, Andreas Henri Diacon, Samantha Fry, Katherine Margaret Gill, Zaheer Ahmed Ebrahim Hoosain, Mina C. Hosseinipour, Mubiana Inambao, Craig Innes, Steve Innes, Dishiki Kalonji, Margaret Kasaro, Priya Kassim, Noel Kayange, William Kilembe, Fatima Laher, Moelo Malahleha, Vongane Louisa Maluleke, Grace Mboya, Kirsten McHarry, Essack Mitha, Kathryn Mngadi, Pamela Mda, Tumelo Moloantoa, Cissy Kityo Mutuluuza, Nivashnee Naicker, Vimla Naicker, Anusha Nana, Annet Nanvubya, Maphoshane Nchabeleng, Walter Otieno, Elsje Louise Potgieter, Disebo Potloane, Zelda Punt, Jamil Said, Yashna Singh, Mohammed Siddique Tayob, Yacoob Vahed, Deo Ogema Wabwire, M. Juliana McElrath, James G. Kublin, Linda-Gail Bekker, Peter B. Gilbert, Lawrence Corey, Glenda E. Gray, Yunda Huang, and Philip Kotze.

## Data sharing statement

This study accesses data through the HIV Vaccine Trials Network (HVTN). Permission to access data will have to be requested from HVTN and Statistical Center for HIV/AIDS Research & Prevention (SCHARP).

## Declaration of interests

Peter B Gilbert received funds from NIAID/NIH, consulting fees from Curevo Vaccine Company and MinervaX Vaccine Company, served unpaid on a Moderna Advisory Board for Zika vaccines, AstraZeneca Vaccine SAB, Sanofi SAB, and Vaccine Company SAB. Lawrence Corey, Fatima Laher, Sharlaa Badal-Faesen, Sinead Delany-Moretlwe, Mina C Hosseinipour, Craig A Magaret, Erica Andersen-Nissen, Yunda Huang, M. Juliana McElrath, Cissy Kityo, Grace Mboya, and Jessica Andriesen received funding from NIAID/NIH paid to their institutions. Sufia Dadabhai and Noel Kayange report salary support from Johns Hopkins University. M. Juliana McElrath reports NIAID and Bill & Melinda Gate's Foundation payments to institution, payment from Stanford, CROI, NIH VRC, Ragon Institute. Richard Lessells is a current member of University of KwaZulu- Natal Biomedical Research Ethics Committee, one of the committees that reviewed and approved the CoVPN 3008 study, however he recused himself from decision making because of the conflict of interest. All other authors have nothing to declare.

## Appendix ASupplementary data

Protocol

Statistical Analysis Plan
