# Supplementary material for: Hybrid versus vaccine immunity of mRNA-1273 among people living with HIV in East and Southern Africa: a prospective cohort analysis from the multicentre CoVPN 3008 (Ubuntu) study
Source: eClinicalMedicine. 2025 Jan 20;80:103054. doi: 10.1016/j.eclinm.2024.103054 (PMC11788791; doi:10.1016/j.eclinm.2024.103054)
Supplement: Protocol [file mmc1.pdf]

COVID-19
Prevention Network

PROTOCOL

CoVPN 3008 (Ubuntu)

Multi-Center, Randomized, Efficacy Study of COVID-19 mRNA Vaccine in Regions with SARS-CoV-2 Variants of Concern

DAIDS DOCUMENT ID 38838

CLINICAL TRIAL SPONSORED BY

South African Medical Research Council (SAMRC)
Cape Town, South Africa

February 27, 2023
Final
CoVPN 3008 (Ubuntu)
Version 7.0

Contents

1 Overview 5
1.1 Protocol Team 9
2 Background 12
2.1 Rationale for trial concept 12
2.2 Moderna mRNA-1273 and mRNA-1273.222 vaccines 24
2.3 Population to be studied 32
3 Objectives and endpoints 35
4 Ethical considerations 39
5 IRB/EC review considerations 41
5.1 Minimized risks to participants 41
5.2 Reasonable risk/benefit balance 41
5.3 Equitable participant selection 42
5.4 Appropriate informed consent 42
5.5 Adequate safety monitoring 42
5.6 Protect privacy/confidentiality 43
5.7 Pregnant participants 43
6 Statistical considerations 44
6.1 Summary of statistical design 44
6.2 Definitions of study cohorts for analysis 44
6.3 Accrual and sample size 47
6.4 Statistical analyses 50
6.5 Operational monitoring for quality of trial conduct 54
6.6 Final analyses timing 55
6.7 Roles of the DSMB, OG, and EAC 55
6.8 Randomization implementation 55
7 Selection and withdrawal of participants 56
7.1 Inclusion criteria 56
7.2 Exclusion criteria 57
8 Study products 59
8.1 Vaccine regimen 59
8.2 Storage, handling, preparation and dispensing of the study products 59
8.3 Acquisition of the study products 59
8.4 Administration of study products 60
8.5 Pharmacy records and accountability 60
8.6 Final disposal of the study products 60
9 Clinical procedures 61
9.1 Informed consent 61
9.2 Pre-enrollment procedures 62
9.3 Enrollment and vaccination visits 63
9.4 Follow-up procedures 65
9.5 Active COVID-19 symptomatic surveillance 66

9.6 COVID-19 Symptom Visits and COVID-19 Post-diagnosis Schedule 67
9.7 Visit windows and missed visits 70
9.8 Assessments of reactogenicity for a subset of participants (Safety Subset) 70
9.9 Delaying vaccinations for a participant 71
9.10 Discontinuing vaccination for a participant who receives a COVID-19 vaccine outside the study 72
9.11 Early termination visit 72
9.12 Pregnancy 72

10 Laboratory 74
10.1 CRS laboratory procedures 74
10.2 Total blood volume 74
10.3 Endpoint assays 74
10.4 Lab assay portfolio 76
10.5 Exploratory studies 76
10.6 Specimen storage and other use of specimens 76
10.7 Biohazard containment 77

11 Safety monitoring and safety review 78
11.1 Safety monitoring and oversight 78
11.2 Safety reporting 79
11.3 Safety reviews 82

12 Protocol conduct 84
12.1 Study termination 84
12.2 Emergency communication with study participants 85
12.3 Site monitoring 86

13 Version history 87

14 Document references (other than literature citations) 96

15 Acronyms and abbreviations 98

16 Literature cited 100

Appendix A Sample informed consent form 113

Appendix B Laboratory procedures table for Groups 1 and 3 (SARS-CoV-2 seronegative participants, main study) 131

Appendix C Laboratory procedures table for Groups 2 and 4 (SARS-CoV-2 seropositive participants, main study) 132

Appendix D Laboratory Procedures for PBMC Immunogenicity subset, Groups 1 and 3 133

Appendix E Laboratory Procedures for PBMC Immunogenicity subset, Groups 2 and 4 134

Appendix F Laboratory Procedures for the PBMC Immune Correlates subset (Group 1, HIV positive, SARS-CoV-2 seronegative participants) 135

Appendix G Laboratory Procedures for the PBMC Immune Correlates subset (Group 2, HIV positive, SARS-CoV-2 seropositive participants) ... 137

Appendix H Schedule of clinic procedures for Groups 1 and 3 (SARS-CoV-2 seronegative participants) ... 138

Appendix I Schedule of clinic procedures for Groups 2 and 4 (SARS-CoV-2 seropositive participants) ... 140

Appendix J COVID-19 symptom visits ... 142

Appendix K Visit windows ... 145

Appendix L Addendum to the sample informed consent form ... 147

Appendix M Addendum 2 to the sample informed consent form for additional procedures 152

Appendix N Adverse Events of Special Interest AESIs ... 158

Appendix O Protocol signature page ... 160

1 Overview

Title

Multi-Center, Randomized, Efficacy Study of COVID-19 mRNA Vaccine in Regions with SARS-CoV-2 Variants of Concern.

Primary objectives

Primary objective 1:

PRE–MONTH 6 STAGE: to assess the relative risk of virologically confirmed symptomatic COVID-19 between a 2-dose mRNA-1273 vaccine regimen in adult people living with HIV (PLWH) who are SARS-CoV-2 negative at baseline (Group 1, vaccine-only immunity) vs. a 1-dose mRNA-1273 regimen in adult PLWH who are SARS-CoV-2 positive at baseline (Group 2, hybrid immunity), counting endpoints starting 1 day after Month 0 dose until Month 6 dose.

Primary objective 2:

PRE–MONTH 6 STAGE: to assess the relative risk of severe COVID-19 between a 2-dose mRNA-1273 vaccine regimen in adult PLWH who are SARS-CoV-2 negative at baseline (Group 1, vaccine-only immunity) vs. a 1-dose mRNA-1273 regimen in adult PLWH who are SARS-CoV-2 positive at baseline (Group 2, hybrid immunity), counting endpoints starting 1 day after Month 0 dose until Month 6 dose.

Primary objective 3:

POST–MONTH 6 STAGE: to assess the relative risk of symptomatic COVID-19 after the Month 6 dose among those who received mRNA-1273 at Month 6 vs. those who received mRNA-1273.222 at Month 6, counting endpoints at least 14 days after the Month 6 dose until end of follow up.

Primary objective 4:

POST–MONTH 6 STAGE: to assess the relative risk of severe COVID-19 after the Month 6 dose among those who received mRNA-1273 at Month 6 vs. those who received mRNA-1273.222 at Month 6, counting endpoints at least 14 days after the Month 6 dose until end of follow up.

Primary objective 5:

To assess the safety and tolerability of the mRNA-1273 and mRNA-1273.222 vaccines in adults who are at risk of severe COVID-19 in both pre-Month 6 and post-Month 6 stages.

Study product, route of administration and regimen

Vaccines: The COVID-19 vaccine mRNA-1273, developed by Moderna, Inc. and commercially sold as Spikevax®, is a lipid nanoparticle (LNP) dispersion of a messenger ribonucleic acid (mRNA) encoding the prefusion stabilized spike (S) protein of the ancestral strain of SARS-CoV-2 (Wuhan-Hu-1) formulated in LNPs composed of 4 lipids (1 proprietary and 3 commercially available). The vaccine is full approved in the United States and has been widely used worldwide.

The COVID-19 vaccine mRNA-1273.222 is an updated bivalent version of Moderna's mRNA-1273 vaccine, composed of equal parts of mRNA-1273 and mRNA that encodes the S protein of the Omicron subvariants BA.4/.5 (which have the same S protein). On August 31, 2022, Moderna received Emergency Use Authorization (EUA) from the US Food and Drug Administration (FDA) and endorsement from the US Centers for Disease Control and Prevention (CDC) and the CDC Advisory Committee on Immunization Practices (ACIP) to permit use of mRNA-1273.222 as a COVID-19 booster vaccine in adults. Over 19 million previously vaccinated Americans have already received doses of this new bivalent Moderna vaccine (1-3).

In this study, at Month 0 (enrollment) all participants will receive a 100 mcg dose of mRNA-1273. At Month 1, all participants without evidence of prior SARS-CoV-2 infection at baseline (based on serology) will receive a second dose of mRNA-1273. At Month 6, all participants (regardless of baseline SARS-CoV-2 status) will receive a 100 mcg dose of either mRNA-1273 or mRNA-1273.222, depending on their randomization at enrollment. As a safety precaution, after the first approximately 120 participants have been vaccinated at Month 6 (the "Month 6 sentinel cohort"), Month 6 vaccinations will be paused. Reactogenicity data from this Month 6 sentinel cohort will be reviewed to decide whether it is safe to continue with Month 6 vaccinations. All vaccines in the study will be delivered as intramuscular (IM) injections into the deltoid muscle.

The final phase of the study, which will last until 12 months from the last enrolment, will focus on follow up for participants vaccinated with mRNA-1273 versus mRNA-1273.222, per randomization, and those with certain conditions considered at risk of prolonged SARS-CoV-2 infection (active TB, HIV with low CD4 count, and/or HIV with unsuppressed viral load). Most participants vaccinated at Month 6 prior to implementation of the bivalent vaccine (ie, all received mRNA-1273) will be exited from the trial prior to completing 12 months of follow up. The remaining participants will be administered monthly nasal swabs for nucleic acid amplification tests [NAATs] in addition to swabs initiated in response to symptoms or otherwise scheduled.

Table 1-1 Schema

Group	Max sample size*	HIV-1 sero-status**	SARS-CoV-2 sero-status**	M0	M1	M6
1A	2100	Positive	Negative	mRNA-1273	mRNA-1273	mRNA-1273
1B	2100			mRNA-1273	mRNA-1273	mRNA-1273.222
2A	4200	Positive	Positive	mRNA-1273	-	mRNA-1273
2B	4200			mRNA-1273	-	mRNA-1273.222
3A	500	Negative	Negative	mRNA-1273	mRNA-1273	mRNA-1273
3B	500			mRNA-1273	mRNA-1273	mRNA-1273.222
4A	1000	Negative	Positive	mRNA-1273	-	mRNA-1273
4B	1000			mRNA-1273	-	mRNA-1273.222
Total	15,600					

* These sample sizes are approximate and ceiling numbers.
**HIV infection and SARS-CoV-2 serostatus are evaluated at screening.

Participants

Up to 15,600 participants age ≥ 18 who are living with HIV or another comorbidity known to be associated with severe COVID-19.

Design

Multicenter trial comparing different regimens of the Moderna mRNA-1273 and mRNA-1273.222 vaccines (see Table 1-1 Schema).

Duration per participant

Up to 12 months per participant.

Estimated total study duration

21 months (includes enrollment and follow-up).

Core operations

CoVPN Vaccine Leadership Group/Core Operations Center, Fred Hutchinson Cancer Center (Fred Hutch) (Seattle, Washington, USA)

Statistical and Data Management Center (SDMC)

Biostatistics, Bioinformatics and Epidemiology Program, Fred Hutch (Seattle, Washington, USA)

The Emmes Company, LLC (Rockville, Maryland, USA)

Bioinformatico (Johannesburg, South Africa)

Laboratory Center (LC)

Endpoint assay laboratories

- Bio Analytical Research Corporation South Africa (Pty) Ltd (Johannesburg, South Africa)
- HIV Sero-Molecular Laboratory–National Institute for Communicable Diseases (Johannesburg, South Africa)
- Fred Hutchinson Cancer Center (Seattle, Washington, USA)
- Cape Town Immunology Laboratory (CHIL, Cape Town, South Africa)
- KwaZulu-Natal Research and Innovation Sequencing Platform (KRISP, Durban, South Africa)
- South African Immunology Laboratory-National Institute for Communicable Diseases (SAIL-NICD, Johannesburg, South Africa)
- Duke University Medical Center (Durham, North Carolina, USA)
- Center for Epidemic Response and Innovation (CERI, Durban, South Africa)
- Yerkes National Primate Research Center (Atlanta, Georgia, USA)

Study sites

- Clinical research sites (CRSs) to be specified in the Site Announcement Memo

Study monitoring

CoVPN 3008 (Ubuntu) Protocol Safety Review Team (PSRT); NIAID HIV Data and Safety Monitoring Board (DSMB). The conduct of the study will be monitored by the NIAID Oversight Group (OG). All the aforementioned groups will conduct their reviews based on their respective operation Charters.

1.1 Protocol Team

Protocol leadership

Cochairs	Nigel Garrett<br>eThekwini CRS / Centre for the AIDS Programme of Research in South Africa (CAPRISA)<br>+27 76 330 8300<br>Nigel.Garrett@caprisa.org<br><br>Philip Kotze<br>Ladysmith CRS / Qhakaza Mbokodo Research Clinic<br>+27 82 458 8969<br>plkotze@gmail.com<br><br>Sufia Dadabhai<br>Blantyre CRS / Johns Hopkins Research Project<br>+265 8 8213 0694<br>Sufia@jhu.edu<br><br>Nyaradzo Mgodi<br>University of Zimbabwe Clinical Trials Research Centre<br>+263 24 270 4920; +263 772 264 616<br>nmgodi@uz-ctrc.org	*Protocol Lead Advisors*	Larry Corey<br>CoVPN LOC, Fred Hutch<br>+1-206-667-6770<br>lcorey@fredhutch.org<br><br>Glenda Gray<br>Perinatal HIV Research Unit (PHRU), Chris Hani Baragwanath Hospital, Soweto; President, South African Medical Research Council<br>+27-11-989 9702<br>glenda.gray@mrc.ac.za<br><br>Linda-Gail Bekker<br>The Desmond Tutu HIV Centre, Cape Town, South Africa<br>+27-21-650-6959<br>linda-gail.bekker@hiv-research.org.za<br><br>Julie McElrath<br>CoVPN LOC, Fred Hutch<br>+1-206-667-6704<br>jmcelrath@fhcrc.org<br><br>Myron Cohen<br>University of North Carolina<br>+1-919-966-2537<br>myron_cohen@med.unc.edu<br><br>James Kublin<br>CoVPN LOC, Fred Hutch<br>+1-206-667-1970<br>jkublin@fredhutch.org
Lead Statisticians	Yunda Huang<br>CoVPN SDMC, Fred Hutch<br>+1-206-667-5780<br>yunda@fredhutch.org<br><br>Peter Gilbert<br>CoVPN SDMC, Fred Hutch<br>+1-206-667-7299<br>pgilbert@scharp.org	*Laboratory Leads*	John Hural<br>CoVPN Laboratory Program<br>+1-206-667-1683<br>jhural@fredhutch.org<br><br>Erica Andersen-Nissen<br>CoVPN Laboratory Program<br>+27 82 385 0625<br>eanderse@hcrisa.org.za<br><br>Guido Ferrari<br>CoVPN Laboratory Program<br>+1-919-684-2862<br>guido.ferrari@duke.edu
Protocol Team Leaders	Manuel Villaran<br>CoVPN LOC, Fred Hutch<br>+1-206-349-4938<br>mvillar2@fredhutch.org<br><br>Asa Tapley<br>CoVPN LOC, Fred Hutch<br>+1-260-667-1204<br>atapley@fredhutch.org	*DAIDS Medical Officers*	Margaret Yacovone<br>DAIDS, NIAID<br>+1-240 292 0826<br>margaret.yacovone@nih.gov<br><br>Laura Polakowski<br>DAIDS, NIAID<br>+1-240-627-3040<br>laura.polakowski@nih.gov

In Country Lead Investigators

Botswana	Joe Makhema<br>Botswana Harvard AIDS Institute<br>+267 390 2671<br>jmakhema@bhp.org.bw	*Uganda*	Peter James Elyanu<br>Baylor – Uganda CRS / Baylor College of Medicine Children's Foundation<br>+256 417-119200<br>Pelyanu@baylor-uganda.org
eSwatini	Harriet Nuwagaba-Biribonwoha<br>Eswatini Prevent Center CRS<br>+268 7602 4678<br>hn2158@cumc.columbia.edu	*Zambia*	Ethel Kamuti<br>Matero CRS / Centre for Infectious Disease Research in Zambia<br>+260 972-877-556<br>Ethel.Kamuti@cidrz.org
Kenya	Taraz Samandari<br>Kisumu CRS / KEMRI-CDC<br>+254-703-485-481<br>tts0@cdc.gov		
Malawi	Sufia Dadabhai<br>Blantyre CRS / Johns Hopkins Research Project<br>+26 5882130694<br>Sufia@jhu.edu	*Zimbabwe*	Zvavahera Chirenje<br>University of Zimbabwe Clinical Trials Research Centre<br>+263 24 2704920;<br>+263 772272813<br>zvavahera.chirenje@ucsf.edu<br><br>Nyaradzo Mgodi<br>University of Zimbabwe Clinical Trials Research Centre<br>+263 24 2704920<br>+263 772264616<br>nmgodi@uz-ctrc.org
South Africa	Nigel Garrett<br>eThekwini CRS / Centre for the AIDS Programme of Research in South Africa (CAPRISA)<br>+27 76 330 8300<br>Nigel.Garrett@caprisa.org<br><br>Philip Kotze<br>Ladysmith CRS / Qhakaza MbokodoResearch Clinic<br>+27 824588969<br>plkotze@gmail.com		

Other contributors to the original protocol

Core medical monitors	Nzeera Ketter<br>CoVPN LOC, Fred Hutch<br>Asa Tapley<br>CoVPN LOC, Fred Hutch<br>Manuel Villaran<br>CoVPN LOC, Fred Hutch	*Regional medical liaison*	Azwidhwi Takalani<br>CoVPN LOC, Fred Hutch
Clinical Research Physician	Aida Asmelash<br>CoVPN, FHI360	*Clinical safety specialists*	Maija Anderson<br>Megan Jones<br>CoVPN LOC, Fred Hutch
Clinical Research Project Manager	Marianne Gildea<br>CoVPN, FHI360	*Clinical trials managers*	Michelle Nebergall<br>Carrie Sopher<br>Lori Proulx-Burns<br>Dhevium Govender<br>CoVPN LOC, Fred Hutch
Laboratory center representatives	Nicole Espy<br>Lisa Sanders<br>Jen Hanke<br>CoVPN Laboratory Center, Fred Hutch	*Clinical data managers*	Melissa Peda<br>CoVPN SDMC, Fred Hutch
Regulatory affairs associate	Laurie Rinn<br>CoVPN LOC, Fred Hutch	*SDMC Associate Director of Operations*	Jessica Andriesen<br>CoVPN SDMC, Fred Hutch
Community educator/recruiter	Sam Sandile Mthiya<br>Durban – eThekwini CRS	*SDMC Associate director of lab science*	April Randhawa<br>CoVPN SDMC, Fred Hutch
Community Advisory Board (CAB) member	Nosipho Cynthia Zungu<br>Durban – eThekwini CRS	*Protocol Pharmacist*	Jackline Odhiambo<br>CoVPN LOC, Fred Hutch
Statistical research associate	Jin Jia Kee<br>CoVPN SDMC, Fred Hutch	*Lead Site Liaison manager*	Bert Le Roux<br>CoVPN LOC, Fred Hutch
Community engagement unit representative	Kagisho Baepanye<br>CoVPN LOC, Fred Hutch	*Protocol development manager*	Smitha Sripathy<br>CoVPN LOC, Fred Hutch
Project coordinator	Haven Wilvich<br>CoVPN LOC, Fred Hutch		

2 Background

2.1 Rationale for trial concept

As the global COVID-19 pandemic has persisted, it has become clear not only that there are great risks faced by people who are immunocompromised, but also that there is an urgent need to better understand infection and disease in these populations so we can prevent severe COVID-19 as well as chronic SARS-CoV-2 infections that could promote transmission and the emergence of new, dangerous variants. This is particularly true for the over 37 million people living with HIV (PLWH) who are concentrated in sub-Saharan Africa and have been significantly underrepresented in COVID-19 vaccine trials. This novel study aims to address critical knowledge gaps by evaluating COVID-19 mRNA vaccine efficacy among PLWH, including the influence of prior infection and degree of immunocompromise. The study will be the largest to date to assess one of the original, monovalent mRNA vaccines versus a new bivalent version that better targets new variants. The study will also seek to characterize viral shedding and the evolution of viral mutations among participants who become SARS-CoV-2 infected. By collecting efficacy and safety data in over half-a-dozen countries across sub-Saharan Africa, the study authors hope to inform regulators in the region and help facilitate access to mRNA vaccine technology across Africa (4) .

Evaluating the effectiveness of mRNA vaccines, including bivalent vaccines, against COVID-19 from VOCs among PLWH

Since the emergence of the COVID-19 global pandemic, two highly efficacious mRNA vaccines targeting the S protein of the ancestral strain of SARS-CoV-2 (Wuhan-Hu-1) have been approved and used widely, Pfizer-BioNTech's BNT162b2 vaccine and Moderna's mRNA-1273 vaccine (5, 6). For immunocompetent individuals the recommended primary series consists of two vaccinations followed months later by at least one booster vaccination, depending on age and comorbidities (7). Evidence from clinical trials and the real world have consistently demonstrated the efficacy of mRNA vaccines in preventing at least severe disease in this population (8-13). In addition, after hundreds of millions of doses administered, these COVID-19 vaccines have proven to be remarkably safe and well tolerated in the general population (14).

Although immunocompromised individuals including PLWH are at elevated risk of severe COVID-19 (15, 16), the optimal approach for vaccinating this population with mRNA vaccines is unclear. Current guidelines from the US Centers for Disease Control and Prevention (CDC) for "moderately and severely immunocompromised" individuals recommend three vaccinations as part of the primary series and at least one booster vaccination (17). But these guidelines are largely based on studies involving people with malignancies or people on immunosuppressive medications (eg, solid organ transplant recipients, people with rheumatic disease) (18-21). While observational studies of PLWH suggest good

immunogenicity and safety from mRNA vaccines (22-24), to date, COVID-19 vaccine efficacy trials have generally only included a small number of PLWH, most of whom were required to have well-controlled disease, and very few studies have reported the number of endpoints in this population (8, 13, 25, 26). Neither the Pfizer-BioNTech nor the Moderna phase 3 efficacy trials enrolled adequate numbers of PLWH to define vaccine efficacy in this population (8, 13). This will be the first large scale study evaluating the effectiveness of mRNA vaccines against symptomatic and severe COVID-19 among PLWH.

Table 2-1 Vaccine efficacy and effectiveness for the ancestral strain of SARS-CoV-2 and Variants of Concern

**Manufacturer**	**Vaccine**	**Vaccine efficacy against ancestral strain for symptomatic infection (severe COVID-19)**	**Vaccine Effectiveness for VOC against Symptomatic Infection (Severe COVID-19)** (27)			
			**Alpha (B.1.1.7)**	**Beta / Gamma (B.1.351 / P.1)**	**Delta (B.1.617.2)**	**Omicron (B.1.1.529)**[1]
**Moderna**	mRNA-1273	94 (100)% (8)	91 (94)%	78 (94)%	70 (96)%	75.1 (75-85 against hospitalization)%
**Pfizer**	BNT162b2	95(100)% (13)	90 (95)%	85 (98)%	85 (95)%	65.5 (80 against hospitalization)%
**Johnson and Johnson (JNJ)**	Ad26.COV2.S	66 (85.4)% (28, 29)	N/A (N/A)	52 (65-66 against hospitalization; 91-95 against mortality)%	N/A (71)%	N/A (N/A)
**Novavax**	NVX-CoV2373	89.7 (100)% (26)	86%	60%	60%	N/A (N/A)
**AstraZeneca**	AZD1222 (ChAdOx1)	67 (100)% (30)	80 (95)%	Under 70% (N/A)	70 (95)%	48.9 (70 against hospitalization)%

Note: refer to Tregoning et al., 2021 (31) and Cevik et al, 2021 (27) for a review of the COVID-19 vaccine effort, SARS-CoV-2 variants versus efficacy, effectiveness, and escape.

[1] Effectiveness at week 4 post 2nd dose; effectiveness dropped in the following weeks (32, 33).

While the currently approved mRNA vaccines have demonstrated durable efficacy against severe COVID-19 disease (34, 35), high rates of breakthrough and repeat infections have highlighted their reduced efficacy against infections from new variants of concern (VOCs) (see Table 2-1), particularly Omicron (B.1.1.529 [BA.1]) and its lineages (eg, BA.2, BA.2.12.1, BA.3, BA.4, BA.5). There is therefore a strong interest in developing updated vaccines to provide broader protection against current and future VOCs. To that end, Moderna, along with Pfizer-BioNTech, has developed new bivalent versions of its mRNA vaccine that include equal parts of mRNA encoding the S protein of the ancestral SARS-CoV-2 (eg, mRNA-1273) as well as mRNA encoding for that of a VOC. Preliminary studies of such bivalent vaccines have been highly encouraging. The vaccine candidate mRNA-1273.211 (mRNA-1273 plus mRNA encoding the S protein of Beta) elicited significantly higher neutralizing antibody responses to the ancestral strain as well as variants (eg, Beta, Delta, and Omicron) compared to mRNA-1273 alone or other monovalent formulations evaluated (36). The bivalent vaccine mRNA-1273.214 (mRNA-1273 plus mRNA encoding the S protein of Omicron [BA.1]) similarly demonstrated superior neutralizing antibody responses to prior

variants as well as the Omicron subvariants BA.4/.5 (37). This bivalent vaccine has already been approved for use in the United Kingdom (37). And as of August 31, 2022, the bivalent vaccine mRNA-1273.222 (mRNA-1273 plus mRNA encoding the S protein of BA.4/.5) has received authorization in the United States for use as a booster vaccine in adults. By randomizing participants at Month 6 to either mRNA-1273 or mRNA-1273.222, this trial will provide valuable efficacy and safety/tolerability data for both monovalent and newer bivalent mRNA vaccines in this high-risk population.

Characterizing SARS-CoV-2 viral persistence and evolution among PLWH on ART and starting ART.

Protecting PLWH and other immunocompromised individuals from SARS-CoV-2 is important for their safety as well as the safety of the global community. Many of the VOCs, from Alpha and Beta to Omicron, have been found to carry mutations that can increase transmissibility, viral binding affinity, and/or immunologic escape (38-40). Multiple studies involving people with immunocompromising conditions have demonstrated long-term viral shedding, which may be infectious (41-43). In addition, an increasing number of studies suggest that prolonged infections in immunocompromised individuals, such as PLWH, may be a major source of the multistep mutational changes associated with VOCs (44-46). In one study, researchers in South Africa examined a SARS-CoV-2 infection persisting over 6 months in a person with advanced HIV that started as the ancestral strain but progressively evolved various mutations found in Omicron and other VOCs and developed substantial immunologic escape from vaccine-associated immunity (45). Given the large number of PLWH globally who have uncontrolled disease (47), there is an urgent need for a better understanding of viral shedding and mutational evolution within PLWH of different degrees of immunosuppression. Therefore, this study will monitor participants who acquire SARS-CoV-2 with sequential nasal swabs to track positivity through PCR testing and potential mutational changes via whole genome sequencing. To our knowledge, this will be the first systematic study to do so among a large cohort of persons closely monitored for such events. Given the size of the trial, we anticipate that this monitoring could provide novel insights into the dynamics of viral shedding and accumulation of viral mutations in SARS-CoV-2-infected PLWH of varying stages of HIV disease.

Comparing the effectiveness of a hybrid immunity regimen with vaccine-induced immunity

In addition to immunocompromise, prior SARS-CoV-2 infection has been underexamined in vaccine trials in terms of its impact on vaccine-associated immunity. It is unclear if people who have a history of past SARS-CoV-2 infection require the same vaccination regimen to achieve robust immunity as people who are infection naïve. This is especially relevant in sub-Saharan Africa where many areas likely have SARS-CoV-2 antibody seroprevalence in excess of 85% (48, 49). There is now significant evidence suggesting that natural infection may prime the

immune system, like a vaccination, and synergistically enhance immunity when a vaccine is later administered (50). This has been termed "hybrid immunity" because it is a combination of natural immunity (derived from infection) and vaccine-induced immunity. Numerous observational studies have shown that previously infected individuals who are subsequently vaccinated demonstrate a particularly vigorous immune response, even after just one dose of mRNA vaccine (51-56). In one study, a single dose of mRNA vaccine given to previously infected individuals boosted neutralizing titers against multiple strains of SARS-CoV-2 (and even SARS-CoV-1) by up to 1,000 times above those measured prior to the vaccination (52). Large prospective studies like this trial are needed to assess hybrid immunity compared to vaccine-only immunity to inform our vaccination strategies in a world now where most people have been previously infected. In addition, the post-Month 6 dose follow up in this trial will also provide information on whether individuals with hybrid versus vaccine-only immunity have different responses to the bivalent vaccine, which is of significant importance in the context of the ongoing epidemic of Omicron variants now globally prevalent.

Modifying the final phase of the study based on findings from interim analyses

Interim analyses of the pre-Month 6 study data have provided new information related to some of the study's main research questions and have informed modifications to the final phase of the study. Consistent with observations in HIV-negative populations, mRNA-1273 appears safe and well tolerated in PLWH. Reactions after vaccinations were largely mild, with few severe reactions (3%) and no life-threatening or fatal reactions. To date, adverse events in general have been overwhelmingly mild and only one serious adverse event (a case of angioedema) has been considered potentially related to the vaccine. The pre-Month 6 data also suggest that in our study population, participants with hybrid immunity (ie, immunity from both prior infection and vaccination) experienced a reduced incidence of symptomatic SARS-CoV-2 infections compared to participants with only vaccine-induced immunity. Specifically, PLWH and HIV-negative participants who had evidence of prior SARS-CoV-2 infection at baseline and received one dose of mRNA-1273 had an annual COVID-19 incidence rate of 2.2% and 1.5% respectively, compared to 7.1% and 5.8% among such groups without evidence of prior infection at baseline and received two doses of mRNA-1273. This reduction in SARS-CoV-2 incidence could be due to the combination of natural immunity and mRNA-1273 vaccination or due to natural immunity alone. In addition, serious COVID-19 has been very rare in our study population during the pre-Month 6 period, with only 9 cases identified to date, including 7 in PLWH and 2 in people who were HIV-negative. However, asymptomatic SARS-CoV-2 infections were more common than previously appreciated, representing 86% of pre-Month 6 cases. In addition, the data suggest that there are a significant number of likely persistent infections. Of the 1298 (9.3%) participants with at least one SARS-CoV-2 positive NAAT, 5.6%, 1.8%, and 1.5% were persistently NAAT positive at ≥ 25, 50, 100 days, respectively. Deep genome sequencing of the sequential samples will help determine if these

are persistent infections or reinfections (likely both) and what proportion of infections have acquired mutations. The persistent/recurrent infections appear to be more common among the participants without prior SARS-CoV-2 infection, those with active tuberculosis, and PLWH who have low CD4 T-cell counts and/or HIV viremia.

The interim analyses suggest that PLWH with SARS-CoV-2 infection who receive one dose of mRNA vaccine, are at significantly lower risk of symptomatic COVID-19 compared with those without prior infection who received a two dose regimen. Moreover, the mRNA-1273 vaccine has proven to be safe and well tolerated by PLWH. Given this, and the fact that monovalent mRNA vaccines like mRNA-1273 will likely not be commercially available much longer (to be replaced by bivalent and other next-generation vaccines), the study authors and the Sponsor decided to end routine research follow up for most participants vaccinated with only mRNA-1273. This change in the follow-up schedule was communicated to clinical research sites in January 2023.

For the final phase of the Ubuntu study, the study will focus on participants vaccinated under protocol version 6.0 (ie, mRNA-1273 versus mRNA-1273.222, per randomization), and those with certain conditions that may increase the risk of prolonged SARS-CoV-2 infections (ie, active TB, HIV with low CD4 count, and/or HIV with unsuppressed viral load). To improve surveillance of asymptomatic infections, participants be administered nasal swab NAATs monthly, in addition to those initiated in response to symptoms or otherwise scheduled. And given the potential association between HIV disease and persistent SARS-CoV-2 infections, additional blood samples will be collected periodically among participants who are nasal swab NAAT positive for prolonged periods. Twelve months of follow up after enrolment will be adequate to accomplish the study objectives.

In addition, a second approach for determining symptomatic COVID-19 cases, based on CDC criteria, will be used. To date the Ubuntu study has used the same approach for determining symptomatic COVID-19 cases as used in the COVE study (8, 12), which was developed early in the pandemic when herd immunity to SARS-CoV-2 was low; symptomatic disease was common; and presymptomatic, paucisymptomatic, and asymptomatic infections were under appreciated. Since then, it has become clear that subclinical infections with few symptoms are common with SARS-CoV-2, particularly with the newer variants. Symptom-driven testing, as required by COVE, may be insensitive to detecting mildly symptomatic infections. The CDC criteria are meant to adjust for the changing pandemic and our greater understanding of the disease in order to better capture mild COVID-19 cases, requiring only 1 symptom be present and not requiring the onset of the symptom(s) to follow the NAAT positive result. The CDC criteria also do not require an Endpoint Adjudication Committee (EAC), although an EAC would continue to be used for determination of the COVE criteria-based endpoints. Two sets of endpoints will be calculated in parallel using both the CDC criteria and the more restrictive COVE criteria.

2.1.1 Overall study design

This study will, to our knowledge, involve the largest cohort of PLWH enrolled into a COVID-19 vaccine clinical trial, and the study is constructed to help inform which vaccine regimen, likely in combination with enhanced HIV care, could serve as a public health model for an effective and cost-efficient approach to preventing SARS-CoV-2 disease, prolonged viral shedding, and the emergence of VOCs within this population. Moreover, we will evaluate whether immune responses post-vaccination can be correlated to these clinically important outcomes.

This multicentered trial aims to enroll up to 15,600 participants, with approximately 80 percent of participants being PLWH. Among these, the majority will be confirmed SARS-CoV-2 seropositive at baseline. All participants will receive at least two vaccines during this trial. Participants will be grouped based on their HIV and SARS-CoV-2 serostatus. Regularly planned safety assessments and interim analyses of vaccine safety will be performed. Trial participants are followed for up to 12 months post-enrollment to evaluate the durability of vaccine efficacy, as well as safety.

Because this study is evaluating a potential primary regimen, 100 mcg doses are used for both vaccines being used, mRNA-1273 and mRNA-1273.222. All vaccines will be delivered as an intramuscular (IM) injection into the deltoid muscle and, as with prior versions of the protocol, modest variations in the injection volume are acceptable assuming no change in dose. The mRNA-1273.222 is currently authorized to be administered as a 50 mcg booster dose, whereas mRNA-1273 is approved for both 50- and 100 mcg doses. All available data on the bivalent vaccine formulations suggest that they have a similar rate of reactogenicity as an equivalent dose of the monovalent mRNA-1273 vaccine. Nevertheless, the study will take an extra safety precaution at the beginning of Month 6 vaccinations, when mRNA-1273.222 is introduced, as described below.

The study is designed essentially with two phases. At Month 0 (enrollment) all participants will receive a dose of mRNA-1273. At Month 1, all participants without evidence of prior SARS-CoV-2 infection at baseline (based on serology) will receive a second dose of mRNA-1273. At Month 6 all participants (regardless of baseline SARS-CoV-2 status) will receive a vaccination, either mRNA-1273 or mRNA-1273.222 depending on their randomization at enrollment. As a safety precaution, after the first approximately 120 participants have been vaccinated at Month 6 (the “Month 6 sentinel cohort”), Month 6 vaccinations will be paused. Reactogenicity data from this Month 6 sentinel cohort will be reviewed to decide whether it is safe to continue with Month 6 vaccinations. After all Month 6 doses are administered, most participants who received their final vaccination prior to implementation of the bivalent mRNA1273.222 vaccine (i.e., all received mRNA-1273) will be exited and the trial will focus on follow up for participants vaccinated with mRNA-1273 versus mRNA-1273.222, per randomization, and those with certain conditions (active TB, HIV with low CD4 count, and/or HIV

with unsuppressed viral load) considered at risk of prolonged SARS-CoV-2 infection.

The relative risk of symptomatic and severe COVID-19 will be assessed between the 2-dose vaccine regimen in adult PLWH who were SARS-CoV-2 negative at baseline vs. a 1-dose regimen in adult PLWH who were SARS-CoV-2 positive at baseline, counting new cases of COVID-19 after the Month 0 vaccination and before the Month 6 vaccination. Next, the study will assess the relative risk of symptomatic and severe COVID-19 between the two different Month 6 vaccines (mRNA-1273 versus mRNA-1273.222) until end of follow-up at Month 12. The study will also assess the safety and tolerability of the two vaccines from the time of administration until the end of follow up.

Approximately 1,500 participants who are baseline SARS-CoV-2 negative PLWH (Group 1) and 1,500 who are baseline SARS-CoV-2 positive PLWH (Group 2) will be included in the PBMC Immune Correlates subset with PBMC collected at Months 0, 2, and 7. In addition, about 300 participants with representation from all study groups will be included in the PBMC Immunogenicity subset with PBMC collected at more frequent timepoints to match the schedule for serum samples collected for humoral immune markers (Figure 2-1, Appendix D and Appendix E). The extra blood samples collected from these subsets of participants will allow the characterization of cellular immune responses through the end of study follow-up (Month 12) in all groups and will support the analyses of cellular immune responses as correlates of risk of and protection against COVID-19 in Groups 1 and 2. This trial will be, to our knowledge, the first efficacy trial to evaluate correlates of protection associated with T-cell immune responses.

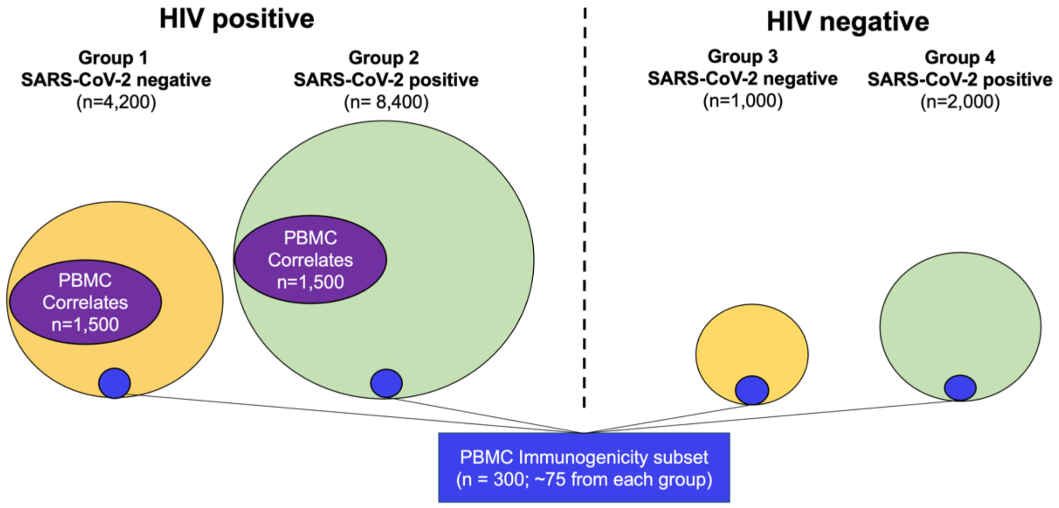

Figure 2-1 Representation of participant inclusion in the PBMC Immune Correlates and PBMC Immunogenicity subsets. (Note: the indicated sample sizes are approximate and ceiling numbers)

With over 10,000 PLWH, the study will have sufficient sample size to provide important outcome information in order to assess the impact of an mRNA vaccine

on the incidence of symptomatic and severe COVID-19. Baseline CD4 T-cell counts and viral loads will be measured to assess the impact of viral control and stage of disease on the immune response where possible. Those with newly diagnosed HIV will be offered counseling and referral for evaluation and care according to local guidelines. Study immunizations will be deferred for 2-4 weeks after the initiation of ART to reduce the difficulty in the interpretation of adverse events. The study team will support sites in monitoring for PLWH participating in the study with unsuppressed viral loads so that they can be referred to local resources in case they need adjustment of their ART regiment or interventions to support adherence.

All participants who acquire new SARS-CoV-2 infection will be monitored with nasal swabs sent for NAAT at 14-day intervals until negative, to characterize the duration of viral shedding. In addition, viral load changes over time will be estimated through cycle threshold values for each NAAT-positive swab. All NAAT-positive swabs will be submitted for gene sequencing to evaluate for the development of new mutations. Participants will also be monitored closely for symptoms. These data will be used to assess potential correlations between these outcomes and degree of immunocompromise, post-vaccination immune response, and other factors. To improve surveillance of asymptomatic infections, participants will be administered nasal swab NAATs monthly after Month 6 in addition to those performed in response to symptoms or otherwise scheduled. Additional blood samples will be collected periodically among participants who are nasal swab NAAT positive for prolonged periods to better assess the potential association between HIV disease and persistent SARS-CoV-2 infections.

Clinical trial sites will be enabled to optimize the detection, management, and follow-up of symptomatic COVID-19 acquired by participants during the course of the trial, including providing access to temperature monitoring, personal pulse oximetry monitoring, oxygen, medications such as steroids, and hospitalization at facilities that are both prepared and experienced with the management of severe cases to minimize potential complications of COVID-19 disease. Study data collected on the duration of viral shedding, PCR cycle thresholds, and viral sequencing may also contribute to optimizing management of SARS-CoV-2 infected participants. The incidence of breakthrough infections will be carefully monitored throughout the study and if there are concerning relative rates of infections among study groups (eg, high incidence of infections in the hybrid immunity group), the DSMB or OG will make recommendations to adjust the course of the study to protect the safety and wellbeing of participants (eg, it could recommend that the study be modified to include an additional vaccine dose, if appropriate). In addition, as previously mentioned, at the start of Month 6 vaccinations (involving mRNA-1273 versus mRNA-1273.222), there will be a vaccination pause after the approximately first 120 participants are vaccinated (the “Month 6 sentinel cohort”) so that reactogenicity data may be reviewed before a decision is made on whether to continue with these vaccinations.

Specific features not present in most study designs of US Government's Department of Health and Human Services [HHS] Coordination Operations and Response Element (HCORE) (previously known as Federal COVID-19 Response, Operation Warp Speed [OWS], and Countermeasures Accelerations Group [CAG]) are included in this study to assess vaccine efficacy against severe COVID-19 as a coprimary efficacy objective in addition to assessing vaccine efficacy against symptomatic infection. Severe COVID-19 is included as a coprimary endpoint because: (1) this endpoint is highly clinically significant, (2) it is feasible to power the study for this endpoint given data showing that a sizable percentage of symptomatic infections are severe in trial countries, and (3) the use of coprimary endpoints can improve statistical power (57).

As noted, the pre-Month 6 vaccine regimens are assigned based on serological evidence of previous SARS-CoV-2 infection. Given demonstrated protection against severe disease and anamnestic immune responses upon re-exposure, we believe that the risk-benefit profile is acceptable for this trial and that the conditions outlined by expert panels are met, namely: 1) the social value of the data is high, 2) the risks to participants are low and justified by the social value of the trial, and 3) the research is responsive to local health needs (58, 59).

2.1.2 Background on the evolution of SARS-CoV-2 variants

Since it was first recognized as a global threat in late 2019, the SARS-CoV-2 virus has adapted to immunologic selection pressures mediated by antibodies that limit, but do not necessarily eliminate, viral replication. Even before the availability of vaccines against SARS-CoV-2, new virus variants had begun to emerge worldwide and are thought to have significantly contributed to new waves of infections and deaths (60, 61). Mutations that lead to increased transmissibility and immunologic escape seem to most determine the success of new variants (38-40) (62-65). Vaccine efficacy against VOCs can be compromised by alterations in the shape of the S protein, which can impair or inactivate binding sites for neutralizing antibodies (66-70).

The first major evidence of SARS-CoV-2 evolutionary change in response to selective pressure was the D614G mutation in the spike protein that emerged in early 2020 and has since become ubiquitous, likely because of its association with increased infectivity (71, 72). By the second half of 2020 the world entered a new stage of the pandemic characterized by the emergence of new SARS-CoV-2 variants with accumulations of mutations facilitating transmission despite population immunity while maintaining or even increasing viral replication (73). In September 2020 the United Kingdom first reported a new variant, B.1.1.7 (Alpha), which had D614G as well as several other important mutations that lent to increased transmission (74-76). By early 2021 Alpha had become the dominant variant in most of Europe, North America, and many parts of Asia and Africa (77). Soon after Alpha was identified, additional highly transmissible variants emerged in populations with significant immunity from prior infection, B.1.351 (Beta) in South Africa and P.1 (Gramma) in Brazil (78, 79). In addition to both strains being

more transmissible, convalescent plasma or vaccine sera showed substantial reductions in neutralizing antibody titers compared to Alpha (80, 81). Both variants became dominant within their immediate region and some other countries, but both were displaced virtually entirely – along with most other variants worldwide – by the emergence of B.1.617.2 (Delta). First detected in India in December 2020, Delta had a number of previously detected and novel mutations which increased transmissibility, immune escape, and severity of disease (67, 82, 83) and led to an increase in cases and deaths in much world (84-86).

The emergence of B.1.1.529 (Omicron), first identified in Botswana and South Africa in November 2021, led to an massive global wave of infections and COVID-19 disease that dwarfed all previous surges (87). Genetically, Omicron had significantly more mutations than had been previously seen in variants, including over 30 in the S gene alone (88). Some of the mutations were ones seen in prior variants that were known to be associated with increased transmissibility, higher viral binding affinity, and immunologic escape (89, 90). But many of the mutations were novel. Phenotypically, the Omicron variant distinguished itself by its remarkable transmissibility, over three-fold greater than Delta, and by its ability to quickly spread in populations with prior immunity. Indeed, studies of the plasma neutralization of the Omicron variant demonstrated an unprecedented degree of neutralizing antibody escape (91, 92).

A leading hypothesis for the origin of Omicron is that it evolved during a chronic infection in someone immunocompromised (93). Indeed, an increasing number of studies suggest that prolonged infections in immunocompromised individuals, including PLWH, may be a major source of the multistep mutational changes associated with VOCs (44-46). In one study, researchers in South Africa examined a SARS-CoV-2 infection persisting over 6 months in a person with advanced HIV that started as ancestral virus but evolved various mutations found in Omicron and other variants and developed substantial immune escape from mRNA-1273 vaccine-associated immunity (45). These findings highlight the urgent need for a better understanding mutational evolution within PLWH of different degrees of immunosuppression to help inform strategies to prevent emergence of further VOCs.

2.1.3 Burden of infections from SARS-CoV-2 variants of concern in Eastern and Southern Africa

SARS-CoV-2 was first identified on the Africa continent in mid-February 2020, and within three months it had been confirmed in every country in Africa (94, 95). From early on, VOCs have been detected in Africa; the Beta variant was first discovered in South Africa in March 2021, and to date, virtually every VOC has been reported on the continent (96, 97).

For the first year of the pandemic, it appeared that Africa had generally avoided the direst predicted consequences of SARS-CoV-2 (although widespread low testing capacity and underreporting obscured assessments). That all changed with

the arrival of the Delta variant in early 2021. By July, the variant was the dominant strain across the continent, and by August death rates had surged by nearly 90% (98, 99). The rising toll was particularly severe in Eastern and Southern Africa. Between June 1 and September 1, 2021, total confirmed deaths from COVID-19 in South Africa rose by 46% to 82,496, and in Kenya, Zambia, and Uganda, deaths rose by 49%, 181%, and 735%, respectively, over the same period (100).

Southern Africa was the first region in the world impacted by Omicron, and it and its subvariants rapidly spread despite significant population immunity from prior infection. Figure 2-2 illustrates the rapid rise of the Omicron variant in 2021 across Africa, and Figure 2-3 demonstrates the associated rise in confirmed SARS-CoV-2 cases. Table 2-2 provides tallies of SARS-CoV-2 cases and associated deaths for a selection of countries across Eastern and Southern Africa.

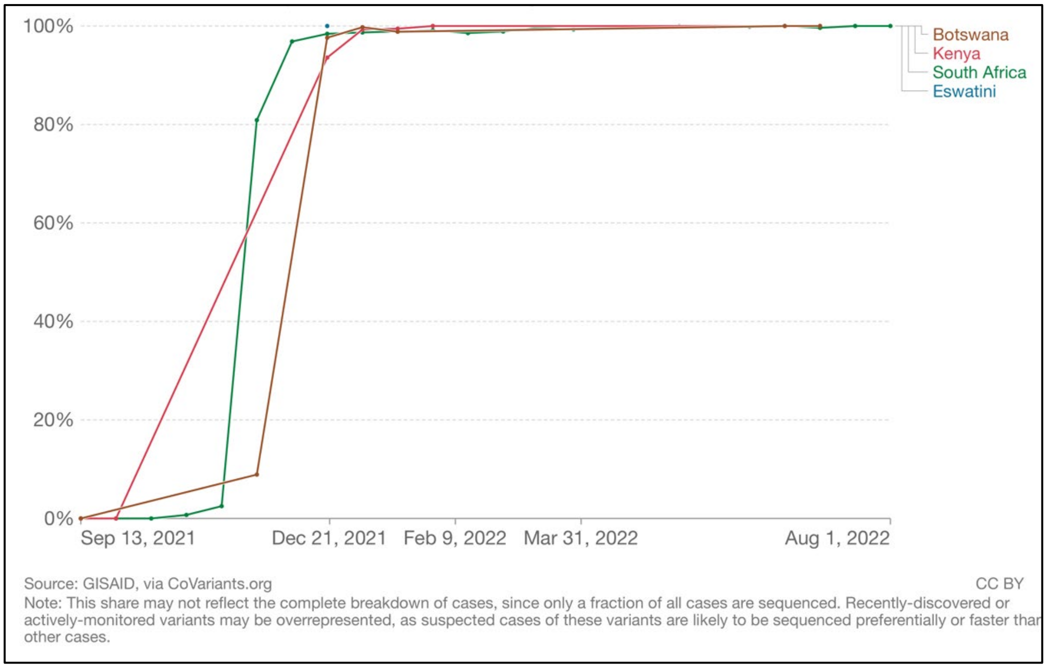

Figure 2-2 Share of SARS-CoV-2 sequences that are the Omicron variant. Shown is the Omicron variant's share of total analyzed sequences in the last two weeks for selection of countries in Sub-Saharan Africa with available data. This share may not reflect the complete breakdown of cases, since only a fraction of all cases are sequenced (Source: Our World in Data (101). Original data from: COVID-19 Data Repository by the Center for Systems Science and Engineering [CSSE] at Johns Hopkins University.)

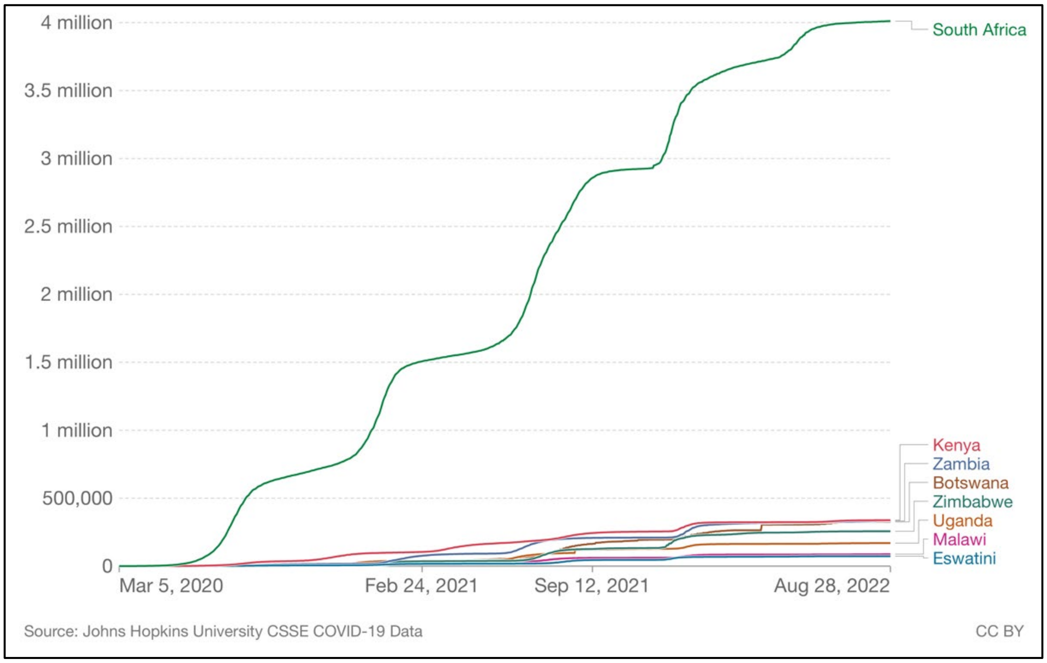

Figure 2-3 Cumulative confirmed COVID-19 cases. The number of confirmed cases is lower than the number of actual cases, mainly due to limited testing. (Source: Our World in Data (101). Original data from: COVID-19 Data Repository by the Center for Systems Science and Engineering [CSSE] at Johns Hopkins University.)

Table 2-2 Cumulative morbidity and mortality due to SARS-CoV-2 in Eastern and Southern Africa. The number of confirmed cases and deaths is lower than the actual numbers, mainly due to limited testing. Data current as of 22 Sep 2022. (Sources: COVID-19 Data Repository by the Center for Systems Science and Engineering [CSSE] at Johns Hopkins University (102) and Worldometer, Countries in the world by population (103).)

Country / Region	Confirmed Cases	Deaths	Population
Worldwide	614.00 M	6.53 M	7.929 M
South Africa	4.02 M	102,169	59.31 M
Botswana	326,127	2,787	2.35 M
Zimbabwe	257,259	5,598	14.86 M
Zambia	333,439	4,017	18.38 M
Malawi	87,997	2,680	19.13 M
Eswatini	73,379	1,422	1.16 M
Uganda	169,396	3,630	45.74 M
Kenya	338,379	5,675	53.77 M

Since the initiation of this study in December of 2021, the Omicron variant epidemic has persisted worldwide including in sub-Saharan Africa. BA.1/BA.2 and then extension of the epidemic by the BA.4/.5 variants has persisted in all regions of the world, with GSAID viral genomic surveillance indicating that, over 98 percent of circulating strains of SARS-CoV-2 are of BA.4/BA.5 subvariants as of September 2022. Many unique subvariants have also been identified, almost all

of which appear to fit phylogenetically within the Omicron subgroup. Thus, the consensus now is that vaccines that target these variants may provide additional protection against Omicron-related disease, and perhaps infections as well. This study will shed light on this issue.

2.2 Moderna mRNA-1273 and mRNA-1273.222 vaccines

This trial will utilize two Moderna vaccines, the monovalent mRNA-1273, which encodes the S protein of the ancestral (Wuhan-Hu-1) SARS-CoV-2 strain, and a bivalent mRNA-1273.222, containing equal parts mRNA-1273 and mRNA encoding the S protein of the Omicron subvariants BA.4/.5. The mRNA-1273 vaccine has been fully approved for adults in the United States and has been widely used in North America and Europe for both the primary vaccine series as well as boosters. The bivalent mRNA-1273.222 is currently authorized in the United States to be used as a booster vaccine, and it is very similar to another bivalent vaccine, mRNA-1273.214, that has been approved by the United Kingdom (37). To date, COVID-19 vaccine access – particularly access to mRNA vaccines – has been limited in Africa. As of September 22, 2022, only 23 percent of Africans had completed a primary vaccination regimen (104). Botswana, Eswatini, Kenya, Malawi, South Africa, Uganda, Zambia, and Zimbabwe have agreed to participate in the COVID-19 Vaccines Global Access (COVAX) initiative, and Moderna has previously committed to provide over 500 million doses to be made available to COVAX (105).

2.2.1 Clinical studies of Moderna mRNA-1273 and bivalent vaccines

The full approval of mRNA-1273 for use in adults in early 2022 was based on a number of large clinical studies in the general population establishing the vaccine's safety, immunogenicity, and efficacy against COVID-19 (Table 2-3). Hundreds of millions of doses of the vaccine have been administered and real-world data have further supported the vaccine's safety and efficacy.

Table 2-3 Summary of clinical studies in adults establishing safety, immunogenicity, and vaccine efficacy of mRNA-1273 in the general population

ClinicalTrials.gov Identifier	Study number	Phase	N	Dose groups	Route	Schedule	Reference
NCT04283461	P101	1	120	25, 50, 100, or 250 mcg	IM	M0, M1	(106, 107)
NCT04405076	P201	2	600	50 or 100 mcg	IM	M0, M1	(108-110)
NCT04470427	P301	3	30,351	100 mcg	IM	M0, M1	(108-110)

While current monovalent mRNA vaccines such as mRNA-1273 do increase antibody responses to VOCs including Omicron when given to previously vaccinated individuals, and real-world data suggests these boosters continue to be effective against severe COVID-19, neutralizing antibody titers are lower against the new VOCs and efficacy against infection appears decreased (111, 112). For this reason, there have been significant efforts to develop bivalent mRNA vaccines that better target VOCs, particularly Omicron.

An ongoing multi-part, open-label, phase 2/3 study (NCT04927065) has been assessing the safety, reactogenicity, and immunogenicity of single doses of Moderna bivalent mRNA candidate vaccines in adults who already received two 100 mcg doses of mRNA-1273 in the phase 3 COVE trial at least 6 months earlier (8, 12, 36, 113). The bivalent vaccines contain equal parts mRNA-1273 and mRNA encoding for the S protein of a VOC. Initial data on these bivalent vaccines have shown significant promise in retaining neutralizing activity against the ancestral SARS-CoV-2 while also providing stronger and more durable immune responses to current VOCs, while being safe and well tolerated.

Table 2-4 Clinical studies with Moderna COVID-19 bivalent vaccines (≥ 18 years of age). All participants previous received a primary series of mRNA-1273 (100 mcg); participants in Parts G & H also previously received a 3rd dose (50 mcg) of mRNA-1273. Part G enrolled Mar 8-23, 2022; Part H enrolled Aug 10-23, 2022. Table adapted from Miller J. Booster Doses of Moderna COVID-19 Vaccines in Adults, Adolescents & Children (114).

Bivalent vaccine	Study (Part)	Dose	N	Median follow-up
Beta (mRNA-1273.211)	205 (A)	3rd	300	245 days
BA.1 Omicron (mRNA-1273.214)	205 (G)	4th	437	43 days
BA.4/.5 Omicron (mRNA-1273.222)	205 (H)	4th	512	Ongoing
		Total	1249	

In study 205 (A) (Table 2-4), 50- and 100 mcg doses of the bivalent vaccine mRNA-1273.211 (containing mRNA-1273 and mRNA encoding for the S protein of the Beta strain) were evaluated. Both doses of the bivalent vaccine elicited significantly higher neutralizing antibody responses against the ancestral, Beta, Delta, and Omicron strains at 1 and 6 months than an extra mRNA-1273 vaccination (36). In addition, both doses were very well tolerated. While the 100 mcg dose of mRNA-1273.211 was associated with slightly more reactogenicity than the 50 mcg dose, it was comparable to what is seen with the FDA-approved 100 mcg dose of mRNA-1273 that is part of the current Moderna primary vaccination regimen and has been given safely to hundreds of millions of people.

And in study 205 (G), the bivalent vaccine mRNA-1273.214 (containing mRNA-1273 and mRNA encoding for the S protein of the Omicron BA.1 strain), demonstrated non-inferior neutralizing antibody titers in response to the ancestral SARS-CoV-2 strain and clearly superior titers to VOCs including Omicron and its subvariants BA.4/.5 compared to the mRNA-1273 booster (113) (Table 2-4 and Figure 2-4). Safety and tolerability were similar to second and third doses of mRNA-1273.

	4th Dose (2nd Booster)	
**Parameter**	**Original (mRNA-1273)** N = 260	**Omicron BA.1 Bivalent (mRNA-1273.214)** (N = 334)
**GMT Pre-booster**	**332**	**298**
95% CI	(282, 391)	(259, 343)
**GMT at Day 29**[1]	**1421**	**2480**
95% CI	(1283, 1574)	(2264, 2716)
**GMT Ratio**[1] **(Bivalent vs Original)**	**1.75**	
97.5% CI	(1.49, 2.04)	
**Seroresponse rate at Day 29**	**99.2%**	**100%**
95% CI	(97.2, 99.9)	(98.9, 100)
**Difference in seroresponse rates**[2]	**1.5**	
97.5% CI	(-1.1, 4.0)	

Superiority of GMTs: Lower 97.5% CI of GMT Ratio > 1.0

Non-inferiority of Seroresponse Rates: Lower 97.5% CI of difference > -10%

Figure 2-4 Omicron BA.1 neutralizing titers were significantly higher following 4th dose (2nd booster) using Omicron BA.1 bivalent (mRNA-1273.214) than with mRNA-1273. Study 205, Per-Protocol Immunogenicity Set with No Prior Infection. 1. Based on ANCOVA model adjusting for age group (<65, ≥65 years) and pre-booster titer. 2. Common risk difference and 97.5% CI were calculated by Miettinen-Nurminen method adjusted for age group (<65, ≥65 years). Table adapted from Miller J. Booster Doses of Moderna COVID-19 Vaccines in Adults, Adolescents & Children (113, 115).

Although study 205 (H), which involves mRNA-1273.222, is ongoing, there is already preclinical (non-human) study data strongly suggesting the efficacy of this bivalent vaccine. In studies with transgenic mice that express human ACE2 on their airway epithelia (to mimic human susceptibility to SARS-CoV-2) and that have previously been given a two-dose primary series of mRNA-1273, an additional dose of mRNA-1273.222 significantly increased the titers of neutralizing antibodies against BA.1 and BA.5 compared to an extra dose of mRNA-1273 (Figure 2-5) (115) . In addition, mice who received the additional dose of mRNA-1273.222 were better protected from infection when challenged with high-dose exposure to BA.5. Similar findings were found with mRNA-1273.214 (Figure 2-6).

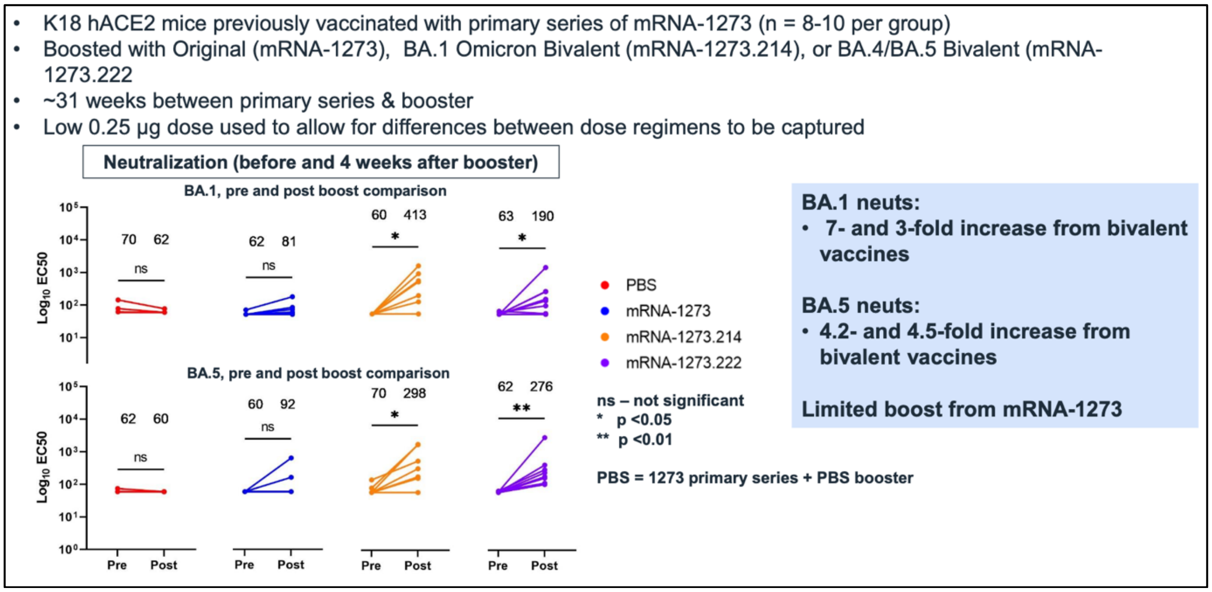

Figure 2-5 Increased immunogenicity after booster dose of the BA.1 & BA.4/.5 Omicron bivalent vaccines (mRNA-1273.214 & mRNA-1273.222) in mice. Figure adapted from Miller J. Booster Doses of Moderna COVID-19 Vaccines in Adults, Adolescents & Children (115)**.**

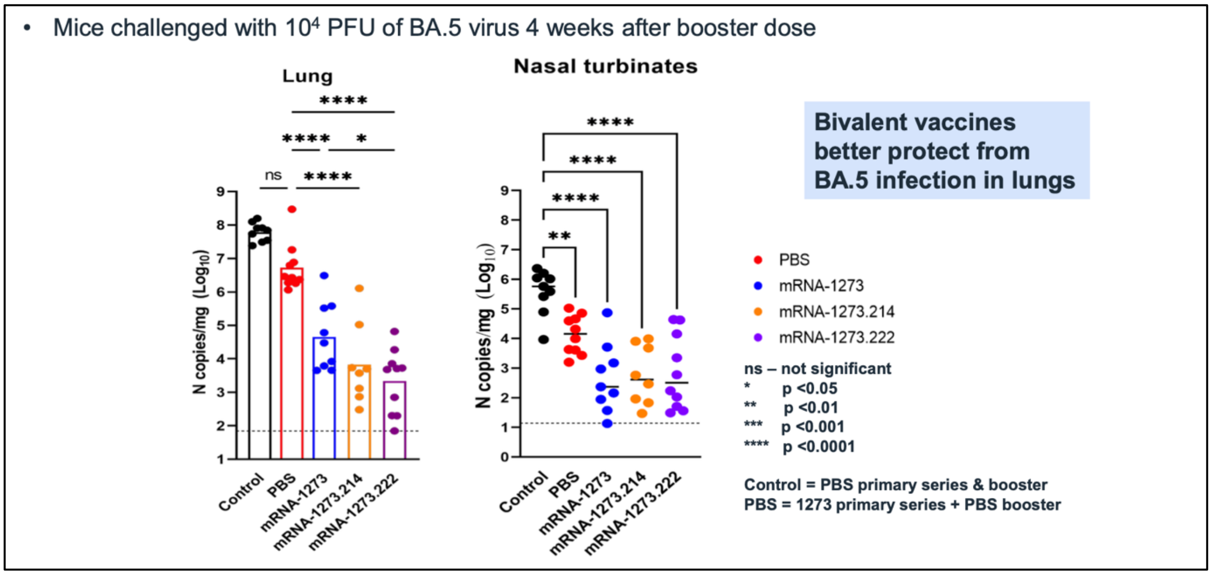

Figure 2-6 Increased protection from BA.5 challenge after booster dose of BA.4/.5 & BA.1 Omicron vaccines (mRNA-1273.214 & mRNA-1273.222) in mice. Figure adapted from Miller J. Booster Doses of Moderna COVID-19 Vaccines in Adults, Adolescents & Children (115)**.**

2.2.2 Rationale for the safety approach in this study

The study takes participant safety very seriously. The COVID-19 mRNA vaccines have excellent safety profiles in clinical trials and based on real-world data. No safety concerns were identified in the initial safety and efficacy trials for the COVID-19 mRNA vaccines other than transient local and systemic reactions (8,

13). On January 31, 2022, after thorough review of the available safety and efficacy data, the FDA approved use of mRNA-1273 for the prevention of COVID-19 in individuals 18 years of age and older. As of August 2022, over 228 million doses of the mRNA-1273 vaccine and 358 million doses of Pfizer-BioNTech's BNT162b2 mRNA vaccine have been administered in the US alone (116). In addition, over 221 million doses of the Moderna vaccine and 797 million doses of the Pfizer-BioNTech vaccine have been administered under the CMA in European countries (103). To date, numerous observational studies have suggested that significant adverse events associated with mRNA vaccines are rare in both the general population as well as specifically immunocompromised populations, including PLWH (7, 14, 22, 117-119).

The safety of mRNA-1273.222 is based on safety data on the nearly identical mRNA-1273.214 from the previously mentioned open-label phase 2/3 study 205 (NCT04927065) (113), safety data from clinical trials involving mRNA-1273 (8, 12), and huge amounts of postmarking safety data with mRNA-1273 (120-122). All of Moderna's bivalent vaccines to date are exceedingly similar to mRNA-1273. In fact, these vaccines include one half portion of mRNA-1273. Study 205 (G) found that the safety and tolerability of mRNA-1273.214 (targeting Omicron as well, albeit BA.1 instead of BA.4/.5) to be comparable to second or third doses of mRNA-1273 (36, 113). After extensive reviews of available safety data, on August 15, 2022, the United Kingdom's Medicines and Healthcare Products Regulatory Agency (MAHPRA) authorized the use of mRNA-1273.214 (37), and on August 31, 2022, the United States FDA authorized the use of mRNA-1273.222 – the bivalent vaccine to be used in this clinical trial (123). To date, over 19 million Americans have received as a booster the same bivalent vaccine being used in the Ubuntu study (3). Recent research examining real-world safety monitoring data for tens of millions of bivalent vaccinations, including millions involving mRNA-1273.222, suggests no difference in safety or tolerability compared to the monovalent mRNA vaccines (124). As of early November 2022, five reports of myocarditis and four reports of pericarditis after bivalent booster vaccination have been received by the US CDC's Vaccine Adverse Event Reporting System (VAERS) following administration of 22.6 million doses among persons aged ≥12 years in the United States (124). In January 2023, an advisory committee to the US FDA endorsed an agency proposal to allow vaccine-naïve Americans to receive the newer bivalent formulations, including the mRNA-1273.222, for their primary regimen (125).

This study will evaluate regimens involving one or two doses of mRNA-1273 in the first month of the trial, and then a final Month 6 dose of either mRNA-1273 or mRNA-1273.222. Because this study is assessing a potential primary regimen, all doses will be 100 mcg to reflect the dosing used in Moderna's standard primary regimen involving mRNA-1273. The administration of three initial 100 mcg doses of mRNA-1273 is already standard for people who are immunocompromised, studies of safety and immunogenicity of a third 100 mcg dose of mRNA-1273 are very reassuring (126, 127), and such dosing of the Moderna vaccine is already part of multiple approved/ongoing clinical trials (128-130). Although mRNA-1273.222

is currently authorized in the United States only as a 50 mcg booster vaccine dose, all available data to date suggests that at the equivalent dose, the bivalent vaccines have similar reactogenicity and safety profiles as mRNA-1273. For example, as previously noted, study 205 (A) evaluated 100 mcg doses of the bivalent vaccine mRNA-1273.211 (containing mRNA-1273 and mRNA encoding for the S protein of the Beta strain), which is highly biosimilar to mRNA-1273.222, and found it was very well tolerated, with reactogenicity and safety event patterns comparable to what is seen with the 100 mcg dose of mRNA-1273.

One of the primary objectives of this study is to collect additional data on the safety and tolerability of the mRNA-1273 and mRNA-1273.222 vaccines in adults who are at risk for severe COVID-19 disease, particularly PLWH. In order to achieve this goal, the study will collect all reports of SAEs and AESIs for all participants throughout the study period, as well as collect solicited local and systemic AEs and unsolicited AEs in a subset of study participants after each vaccination. In addition, after the first approximately 120 participants have received their Month 6 vaccination (a 100 mcg dose of either mRNA-1273 or mRNA-1273.222, depending on their randomization at enrollment), there will be a vaccination pause. Reactogenicity data on all members of this Month 6 sentinel cohort will be collected and reviewed before a decision is made on whether it is safe to continue Month 6 vaccinations.

As previously noted, the vaccine regimens before Month 6 of one versus two doses of mRNA-1273 are assigned based on serological evidence of prior SARS-CoV-2 infection. This is based on significant evidence that prior infection provides protection potentially comparable to a vaccination, and we believe that the risk-benefit profile is acceptable for this trial in terms of the high social value of the data, the low risks to participants that are justified by the social value of the trial, and the responsiveness of the research to local health needs (58, 59).

Clinical trial sites will be enabled to optimize the detection, management, and follow-up of symptomatic COVID-19 acquired by participants during the course of the trial. For example, this includes access to temperature monitoring, personal pulse oximetry monitoring, oxygen, medications such as steroids, and hospitalization at facilities that are both prepared and experienced with the management of severe COVID-19. Study data such as the duration of viral shedding, PCR cycle thresholds, and viral sequencing may contribute to optimizing management of COVID-19 among participants. The study has robust safety and reactogenicity monitoring, with a PSRT and DSMB providing ongoing assessment of this data at the individual and aggregate level, respectively. The rates of breakthrough infections and safety and reactogenicity events will be carefully monitored and if there are concerning relative rates among study groups, the DSMB will be able to make recommendations to adjust the course of the study to protect the safety and wellbeing of participants.

2.2.3 Potential risks of mRNA-1273 and mRNA-1273.222 vaccines

Known risks associated with receipt of the Moderna mRNA-1273 vaccine in the general population are described in the "Fact Sheet For Healthcare Providers Administering Vaccine (Vaccination Providers) Emergency Use Authorization (EUA) of the Moderna COVID-19 Vaccine To Prevent Coronavirus Disease 2019 (COVID-19)," available at https://www.modernatx.com/covid19vaccine-eua/ (revised August 31, 2022). Similarly, known risks associated with receipt of the Moderna mRNA-1273.222 vaccine are described in " Fact sheet for Healthcare Providers Administering Vaccine (Vaccination Providers) Emergency Use Authorization of the Moderna COVID-19 Vaccine, Bivalent (Original and Omicron BA.4/.5)," available at https://www.fda.gov/media/161318/download (revised August 31, 2022). Some of the risks are also described below. Regarding participants who are PLWH, please refer to Section 2.3. Documented adverse reactions include pain, swelling, and erythema at the injection site; axillary swelling/tenderness; fatigue; headache; myalgia; arthralgia; chills; nausea/vomiting; and fever. Figure 2-7 below renders published reactogenicity data for the mRNA-1273 SARS-CoV-2 vaccine in the P301 (COVE) phase 3 efficacy trial NCT04470427, in which over 30,000 participants were enrolled and, aside from transient local and systemic reactions, no safety concerns were identified (8). Solicited local and systemic reactions to mRNA-based COVID-19 vaccines (Moderna and Pfizer-BioNTech) collected by CDC via the v-safe active surveillance system from more than 3 million people (from December 14, 2020 to March 14, 2021) have been published and continue to support an excellent safety profile of this vaccine platform (131). CDC's reports of myocarditis and pericarditis following the two-dose mRNA primary vaccination series among individuals 18-39 years of age show that young men are the group at highest risk (40 cases per million doses administered in the group between 18-24 years of age) and most events occur after the second dose; overall reporting rates exceed the background rates for males and females (132). Nevertheless, the CDC has analyzed the risk-benefit of mRNA vaccination for both Moderna and Pfizer-BioNTech and recommended vaccination in all population groups being enrolled in this trial. SARS-CoV-2 infection has a 5- to 20-fold higher rate of myocardial disease than vaccination with mRNA vaccines (133). The addition of a third dose of the Moderna vaccine, according to an interim analysis of an ongoing clinical trial (NCT04405076) (134), does not introduce further risk of AEs, since the safety profile of a single booster dose was similar to the one observed following the primary vaccination series. In addition, clinical trial and real-world data on the safety of a third dose of the Pfizer-BioNTech vaccine have been very reassuring, indicating reactions comparable to those seen after a second dose (135, 136).

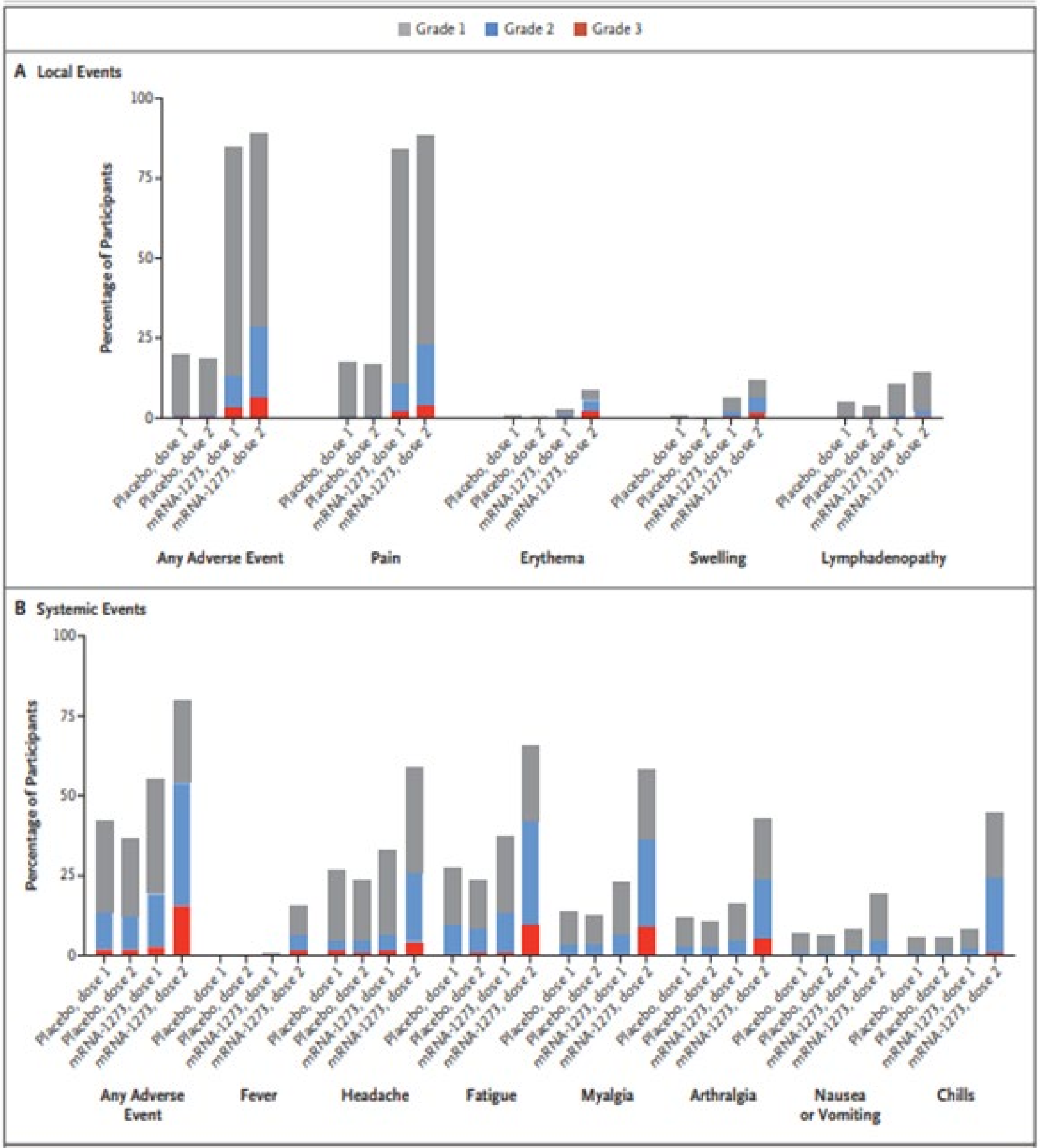

Figure 2. Solicited Local and Systemic Adverse Events.
Shown is the percentage of participants who had a solicited local or systemic adverse event within 7 days after injection 1 or injection 2 of either the placebo or the mRNA-1273 vaccine.

Figure 2-7 Solicited local and systemic adverse events. Shown is the percentage of participants who had a solicited local or systemic adverse event within 7 days after injection 1 or injection 2 of either the placebo or the mRNA-1273 vaccine (from Baden et al, Efficacy and Safety of the mRNA-1273 SARS-CoV-2 Vaccine. N Engl J Med. 2021 (8)).

In terms of potential risks from mRNA-1273.222, as summarized in sections 2.2.1 and 2.2.2, ongoing phase 2/3 studies of bivalent vaccine candidates have found that its safety and tolerability is consistently comparable to second or third doses of mRNA-1273 (36, 113).

Figure 2-8 shows reactogenicity data for mRNA-1273.214, which is highly similar to the mRNA-1273.222, with both targeting a strain of Omicron.

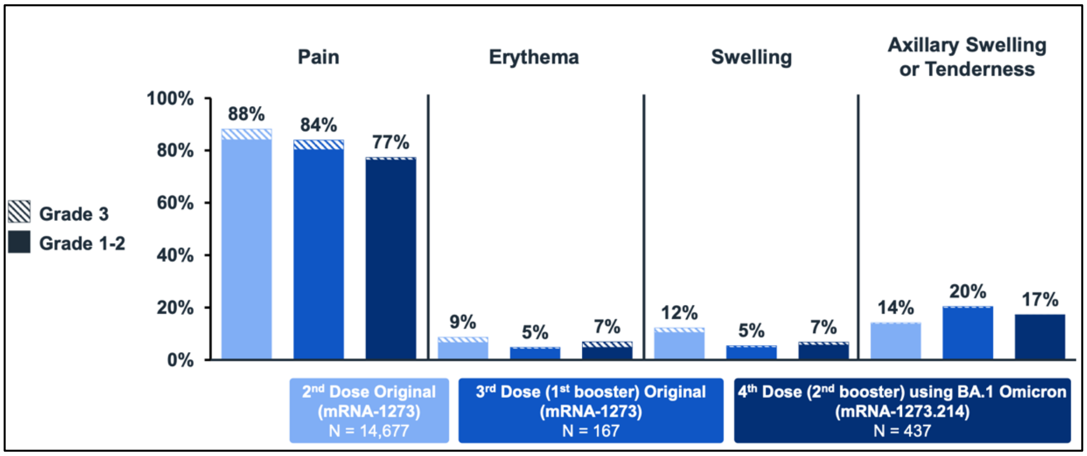

Figure 2-8 Local reactogenicity of BA.1 Omicron bivalent (mRNA-1273.214) as 4th dose similar to 2nd dose of primary series and 3rd dose of original (mRNA-1273) in adults. Study 205 (G), safety subset. Solicited local adverse reactions within 7 days after injection. No Grade 4 events reported. Figure adapted from Miller J. Booster Doses of Moderna COVID-19 Vaccines in Adults, Adolescents & Children (8, 113, 115, 134).

2.3 Population to be studied

This study will enroll up to 15,600 adults at risk of severe COVID-19 based on individual risk factors in order to enrich the study population for SARS-CoV-2 infection and severe COVID-19 disease. The study will enroll participants 18 years and older, approximately 80 percent PLWH and 20 percent HIV-negative people with one or more comorbidities associated with increased risk of severe COVID-19. The proportions of these various risk groups will be operationally managed to ensure that there is adequate representation within this study to draw conclusions about clinical outcomes.

The underlying medical conditions that have compelling evidence for an increased risk of severe illness from COVID-19 have been summarized and regularly updated by the CDC. The conditions for which the strongest and most consistent evidence is available include hypertension, cancer, chronic kidney disease, chronic lung diseases (chronic obstructive pulmonary disease [COPD], interstitial lung disease, pulmonary embolism, bronchiectasis, pulmonary hypertension), heart conditions (eg, heart failure, coronary artery disease, cardiomyopathy,), disabilities (including attention-deficit/hyperactivity disorder [ADHD], cerebral palsy, limitations with self-care or activities of daily living, intellectual and developmental disabilities, learning disabilities, spinal cord injuries), cerebrovascular disease, chronic liver diseases (cirrhosis, non-alcoholic fatty liver disease, alcoholic liver disease, autoimmune hepatitis), cystic fibrosis, dementia, HIV, mental health disorders (mood disorders including depression, schizophrenia spectrum disorder), alpha-1 antitrypsin deficiency, asthma, bronchopulmonary

dysplasia, hepatitis B, hepatitis C, obesity (body mass index [BMI] ≥ 30 kg/m^2), severe obesity (BMI ≥ 40 kg/m^2), sickle cell disease, tuberculosis, smoking, type 1 and type 2 diabetes mellitus, pregnancy, immunocompromised states (eg, medication-induced immunosuppression after solid organ transplant, use of corticosteroids), and physical inactivity. Additional risk factors may be identified in the future. The reason for this weighting is to provide early and strong evidence that the data from the trial can be utilized for population-based efficacy globally.

This study may enroll pregnant persons as part of the study, and study participants who become pregnant during the study may remain enrolled, but the number of pregnant persons enrolled is unlikely to be adequate to provide specific relative vaccine efficacy data for this population. Safety of mRNA vaccines in pregnant women has been adequately demonstrated and the CDC and WHO recommend vaccination against COVID-19 during pregnancy (102, 137-139). A recent study has also shown that COVID-19 vaccination is not associated with a reduction in chances for conception (140). Study sites that enroll pregnant persons will be selected based on well-established and proven experience for the optimal care and management of pregnant persons, including prenatal ultrasound evaluation to assess gestational age and potential fetal abnormalities. Participants who are pregnant at the time of enrollment or become pregnant during the study and are not known to be living with HIV will receive or be referred for HIV testing and counseling, as well as syphilis testing and potential treatment if indicated.

People living with HIV

PLWH are a major focus of this study, given the current significant evidence gap regarding what COVID-19 vaccine regimens would best benefit this group. PLWH will constitute the largest proportion of the study population, and study sites will be selected based on the presence of well-established systems for the diagnosis and care of PLWH.

Studies have consistently suggested that PLWH are at elevated risk of poor outcomes from COVID-19 compared to HIV-uninfected individuals. In a multicenter registry of 286 PLWH who got COVID-19, 94 percent were on HIV antiretroviral therapy and 57 percent were hospitalized, of which 16 percent were in the ICU with a 9 percent mortality rate. This study found that severe COVID-19 outcomes were associated with older age, lung disease, hypertension, and overall comorbidity burden (141). In the UK, a study of 123 PLWH out of 47,000 individuals hospitalized with COVID-19 found that the overall mortality was similar (27 percent for PLWH and 32 percent for people without HIV) but the adjusted hazard ratio was slightly higher for PLWH (1.52 vs 1.92) (142). A study of 50,167 people with COVID-19, of which 404 were PLWH, showed that the latter were more likely to have comorbidities that have risk factors for severe COVID-19, such as hypertension, diabetes, chronic kidney disease, and nicotine dependence (143). The degree of immunosuppression may play an important role in poor outcomes.

In addition to the elevated risk of morbidity and mortality from COVID-19, PLWH also face a risk of prolonged infections and the potential for a longer period of infectiousness and the development of new mutations. A growing number of studies involving people with immunocompromising conditions have demonstrated many instances of long-term viral shedding, sometimes infectious (41-43). In addition, an increasing number of studies suggest that prolonged infections in immunocompromised individuals, including PLWH, may be a major source of the multistep mutational changes associated with the emergence of VOCs (44-46). The evolution of new mutations enabling increased immunologic escape during chronic SARS-CoV-2 infection has been well-documented in PLWH (45).

To date, COVID-19 vaccine efficacy trials have generally only included a small number of PLWH, most of whom were required to have well-controlled disease, and very few studies have reported the number of endpoints in this population (12, 13). None of the primary vaccine efficacy trials enrolled adequate numbers of PLWH to determine vaccine efficacy in this population. Although there continues to be a lack of efficacy and safety data from controlled clinical trials on mRNA vaccines in PLWH, an increasing number of observational studies of PLWH suggest acceptable immunogenicity and safety (22, 24, 118, 119) .

In the case of a new HIV diagnosis, participants will be referred for HIV standard of care according to local guidelines. Participants with newly diagnosed HIV infection will have a CD4 and viral load assessment. If a participant is diagnosed with HIV infection at screening and antiretroviral treatment is initiated, the study vaccines may be administered approximately 2-4 weeks later to avoid overlapping AEs. In addition, the study team will support sites in monitoring for PLWH participating in the study with unsuppressed viral loads so that they can be referred to local resources in case they need adjustment of their ART regiment or interventions to support adherence.

Monitoring of the trial will include safety and efficacy monitoring of symptomatic cases of COVID-19. All confirmed cases of COVID-19 that develop during the study will receive special attention and close follow-up to permit early intervention to prevent complications, morbidity, and mortality. These include temperature monitoring, routine pulse oximetry, provision of oxygen, steroids and other medications, and hospitalization.

3 Objectives and endpoints

All symptomatic COVID-19 endpoints will be evaluated using two case definitions, one used in the COVE study (8, 12) and the other using a case definition based on criteria from the CDC (144, 145). The first two primary objectives count COVID-19 and severe COVID-19 endpoints starting 14 days after the last pre-Month 6 dose until the Month 6 dose in the pre-Month 6 stage, and compare the rate of these endpoints between adult PLWH with vaccine-only immunity vs. adult PLWH with hybrid immunity. The third and fourth primary objectives count COVID-19 and severe COVID-19 endpoints starting 14 days after dose 3 until the end of follow-up in the post-Month 6 stage, and compare the rate of these endpoints in adult PLWH who were randomized and received mRNA-1273 vs. mRNA-1273.222 vaccine. Secondary objective 1 compares rates of COVID-19 and severe COVID-19 in the pre-Month 6 stage during a different period of follow-up: starting 14 days after dose 2 for participants who are SARS-CoV-2 negative at baseline and starting 14 days after dose 1 for participants who are SARS-CoV-2 positive at baseline. Answers to primary objectives 1 and 2 will be available earlier than answers to primary objectives 3 and 4, such that essentially, the trial has two stages answering study questions comparing hybrid vs. vaccine-only immunity, and comparing two different month 6 vaccinations.

Primary Objectives	Primary Endpoints
1. PRE-MONTH 6 STAGE: to assess the relative risk of symptomatic COVID-19 in Group 1 (PLWH who are SARS-CoV-2 negative at baseline) who receive a 2-dose mRNA-1273 vaccine regimen vs. in Group 2 (PLWH who are SARS-CoV-2 positive at baseline) who receive a 1-dose mRNA-1273 regimen, counting endpoints starting 1 day after Month 0 dose until Month 6 dose.	1. COVID-19: Positive result of acute SARS-CoV-2 infection as assessed by nucleic acid amplification testing (NAAT) of a nasal swab and the presence of a prespecified symptom or symptoms (see footnotes 2 and 3)
2. PRE-MONTH 6 STAGE: to assess the relative risk of severe COVID-19 in Group 1 (PLWH who are SARS-CoV-2 negative at baseline) who receive a 2-dose mRNA-1273 vaccine regimen vs. in Group 2 (PLWH who are SARS-CoV-2 positive at baseline) who receive a 1-dose mRNA-1273 regimen, counting endpoints starting 1 day after Month 0 dose until Month 6 dose.	2. Severe COVID-19: COVID-19 that meets the criteria listed in footnote 4
3. POST-MONTH 6 STAGE: to assess the relative risk of symptomatic COVID-19 after the Month 6 dose among those who received mRNA-1273 at Month 6 vs. those who received mRNA-1273.222 at Month 6, counting endpoints at least 14 days after the Month 6 dose until end of follow up.	3. COVID-19: same as above
4. POST-MONTH 6 STAGE: to assess the relative risk of severe COVID-19 after the Month 6 dose among those who received mRNA-1273 at Month 6 vs. those who received mRNA-1273.222 at Month 6, counting endpoints at least 14 days after the Month 6 dose until end of follow up.	4. Severe COVID-19: same as above

Primary Objectives	Primary Endpoints
5. To assess the safety and tolerability of mRNA-1273 and mRNA-1273.222 in adults who are at risk of severe COVID-19 in both the pre-Month 6 and post-Month 6 stages.	5. Adverse events: Solicited and unsolicited AEs collected for a subset of study participants. Serious adverse events (SAEs), adverse events of special interest (AESIs), collected for all participants throughout the study

[1] HIV-infection status and SARS-CoV-2 status are confirmed for each participant based on testing of samples collected during screening (serology) and at enrollment (NAAT). SARS-CoV-2 negative indicates both NAAT negative and serology negative.

[2] To be considered as a case of COVID-19 using the COVE case definition for the evaluation of the Primary Efficacy Endpoint, the following criteria must be met:

1. The participant must have experienced at least TWO of the following systemic symptoms: Fever (≥ 38ºC), chills, myalgia, headache, sore throat, new loss of taste or smell, OR
2. The participant must have experienced at least ONE of the following respiratory signs/symptoms: cough, shortness of breath or difficulty breathing, OR clinical or radiographical evidence of pneumonia; AND
3. The participant must have at least 1 nasal swab (or respiratory sample, if hospitalized) positive for SARS-CoV-2 by NAAT within 14 days following the above systemic or respiratory symptoms.

[3] To be considered as a case of COVID-19 using the CDC-derived case definition for the evaluation of the Primary Efficacy Endpoint, the following criteria must be met:

1. The participant must have experienced at least ONE of the following systemic symptoms: Fever (≥ 38ºC), chills, myalgia, headache, sore throat, new loss of taste or smell, OR
2. The participant must have experienced at least ONE of the following respiratory signs/symptoms: cough, shortness of breath or difficulty breathing, OR clinical or radiographical evidence of pneumonia; AND
3. The participant must have at least 1 nasal swab (or respiratory sample, if hospitalized) positive for SARS-CoV-2 by NAAT within 14 days of any of the above systemic or respiratory symptoms.

[4] To be considered as a case of severe COVID-19 for the evaluation of the Secondary Efficacy Endpoint, the following criteria must be met:

- A confirmed case of COVID-19, per a Primary Efficacy Endpoint case definition, plus any of the following:
  - Clinical signs indicative of severe systemic illness, Respiratory Rate ≥ 30 per minute, Heart Rate ≥ 125 beats per minute, SpO2 ≤ 93% on room air at sea level or PaO2/FIO2 < 300 mm Hg, OR
  - Respiratory failure or Acute Respiratory Distress Syndrome (ARDS), (defined as needing high-flow oxygen, noninvasive or mechanical ventilation, or ECMO), evidence of shock (systolic blood pressure < 90 mmHg, diastolic BP < 60 mmHg or requiring vasopressors), OR
  - Significant acute renal, hepatic, or neurologic dysfunction, OR
  - Admission to an intensive care unit or death.

Secondary Objectives	Secondary Endpoints
1. PRE-MONTH 6 STAGE: to assess the relative risk of symptomatic COVID-19 and of severe COVID-19 in Group 1 (PLWH who are SARS-CoV-2 negative at baseline) vs. in Group 2 (PLWH who are SARS-CoV-2 positive at baseline), counting endpoints at least 14 days after the last pre-Month 6 dose (i.e., dose 2 for Group 1 and dose 1 for Group 2) until the Month 6 dose.	COVID-19 and severe COVID-19 as defined for the primary endpoints
2. PRE-MONTH 6 STAGE: to assess the relative risk of symptomatic COVID-19 and of severe COVID-19 in Groups 1 and 3 together (participants who are SARS-CoV-2 negative at baseline regardless of HIV status) vs. in Groups 2 and 4 together (participants who are SARS-CoV-2 positive at baseline regardless of HIV status), counting	COVID-19 and severe COVID-19 as defined for the primary endpoints

Secondary Objectives	Secondary Endpoints
endpoints starting 1 day after dose 1, and counting endpoints at least 14 days after the last pre-Month 6 dose (ie,, dose 2 for Group 1 and dose 1 for Group 2) until the Month 6 dose.	
3. POST-MONTH 6 STAGE: to assess the relative risk of symptomatic COVID-19 and of severe COVID-19 after the Month 6 dose in Group 1 (PLWH who are SARS-CoV-2 negative at baseline) and Group 2 (PLWH who are SARS-CoV-2 positive at baseline), separately and combined, among those who received mRNA-1273 at Month 6 vs. those who received mRNA-1273.222 at Month 6, counting endpoints at least 14 days after the Month 6 dose until end of follow up.	COVID-19 and severe COVID-19 as defined for the primary endpoints
4. POST-MONTH 6 STAGE: to assess the relative risk of virologically confirmed symptomatic COVID-19 and of severe COVID-19 after the Month 6 dose in Groups 1 and 3 together (participants who are SARS-CoV-2 negative at baseline regardless of HIV status) and in Groups 2 and 4 together (participants who are SARS-CoV-2 positive at baseline regardless of HIV status), respectively, among those who received mRNA-1273 at Month 6 vs. those who received mRNA-1273.222 at Month 6, counting endpoints at least 14 days after Month 6 dose until the end of follow up.	COVID-19 and severe COVID-19 as defined for the primary endpoints
5. PRE-MONTH 6 & POST-MONTH 6 STAGES: to assess the relative risk of symptomatic COVID-19 and of severe COVID-19 after the Month 6 dose by neutralization phenotype and Spike sequence features of acquired SARS-CoV-2 viruses (sieve analysis) in Groups 1 and 3 together (participants who are SARS-CoV-2 negative at baseline regardless of HIV status) and in Groups 2 and 4 together (participants who are SARS-CoV-2 positive at baseline regardless of HIV status), respectively, among those who received mRNA-1273 at Month 6 vs. those who received mRNA-1273.222 at Month 6, counting endpoints starting 1 day after dose 1, 14 days after the last pre-Month6 dose , or 14 days after Month 6 dose until the end of follow up.	COVID-19 and severe COVID-19 as defined for the primary endpoints; neutralization phenotypes and viral genotypic characteristics of SARS-CoV-2 at diagnosis.
6. PRE-MONTH 6 & POST-MONTH 6 STAGES: to assess the relative risk of SARS-CoV-2 infection defined by nucleocapsid protein seroconversion regardless of symptomology after the Month 6 dose in Group 1 (PLWH who are SARS-CoV-2 negative at baseline) and in Group 3 (people HIV-negative who are SARS-CoV-2 negative at baseline) combined, among those who received mRNA-1273 at Month 6 vs. those who received mRNA-1273.222 at Month 6, counting endpoints starting 1 day after dose 1, 14 days after the last pre-Month 6 dose , or 14 days after Month 6 dose until the end of follow up.	SARS-CoV-2 infection diagnosed by seroconversion throughout the study.
7. PRE-MONTH 6 & POST-MONTH 6 STAGES: to assess the relative risk of asymptomatic SARS-CoV-2 infection defined by nucleocapsid protein seroconversion after the Month 6 dose in Group 1 (PLWH who are SARS-CoV-2	SARS-CoV-2 infection diagnosed by seroconversion in the absence of symptoms.

Secondary Objectives	Secondary Endpoints
negative at baseline) and in Group 3 (people HIV-negative who are SARS-CoV-2 negative at baseline) combined, among those who received mRNA-1273 at Month 6 vs. those who received mRNA-1273.222 at Month 6, counting endpoints starting 1 day after dose 1, 14 days after the last dose before Month 6 dose, or 14 days after Month 6 dose until the end of follow up.	
8. PRE-MONTH 6 & POST-MONTH 6 STAGES: to assess the relative risk of symptomatic COVID-19, and severe COVID-19 after the Month 6 dose in Groups 1 and 3 together (participants who are SARS-CoV-2 negative at baseline regardless of HIV status) and in Groups 2 and 4 together (participants who are SARS-CoV-2 positive at baseline regardless of HIV status), respectively, among those who received mRNA-1273 at Month 6 vs. those who received mRNA-1273.222 at Month 6, counting endpoints starting 1 day after dose 1 or 14 days after the last dose before Month 6 dose until the end of follow up.	COVID-19 and severe COVID-19 as defined above
9. To assess post-vaccination immune response markers and immune markers as correlates of risk of COVID-19 and as correlates of protection against COVID-19 in all study groups together.	SARS-CoV-2-specific neutralization antibody, binding antibody and cellular immune responses
10. To assess SARS-CoV-2 viral persistence and evolution among participants virologically diagnosed of SARS-CoV-2 infection in all study groups together.	SARS-CoV-2 viral load (as inferred from RT-PCR cycle threshold values) and viral whole genome sequences

Exploratory Objectives	Exploratory Endpoints
1. To assess the relative risk of COVID-19 and severe COVID-19 by baseline HIV infection status in participants.	COVID-19 and severe COVID-19 as defined for the primary endpoints
2. To estimate VE of COVID-19 and severe COVID-19 using counterfactual placebo	COVID-19 and severe COVID-19 as defined for the primary endpoints
3. Assess post-infection T-cell responses in participants who develop COVID-19	SARS-CoV-2-specific T-cell immune responses; COVID-19 as defined above
**Additional Exploratory Objectives**	
4. To further evaluate immunogenicity of the vaccine, additional assays may be performed	
5. To conduct analyses related to furthering the understanding of SARS-CoV-2, coronavirus, immunology, vaccines, and clinical trial conduct	

4 Ethical considerations

It is critical that universally accepted ethical guidelines are followed at all sites involved in the conduct of clinical trials. The COVID-19 Prevention Network (CoVPN) have addressed ethical concerns in the following ways:

- CoVPN trials are designed and conducted to enhance the scientific knowledge base necessary to find a preventive vaccine, using methods that are scientifically rigorous and valid, and are in accordance with International Council for Harmonisation of Technical Requirements for Pharmaceuticals for Human Use (ICH) and/or other Good Clinical Practice (GCP) guidelines.

- CoVPN scientists and operational staff incorporate the philosophies underlying major codes (146-148), declarations, and other guidance documents relevant to human subjects research into the design and conduct of SARS-CoV-2 vaccine clinical trials.

- CoVPN scientists and operational staff are committed to substantive community input—into the planning, conduct, and follow-up of its research—to help ensure that locally appropriate cultural and linguistic needs of study populations are met.

- The CoVPN provides training so that all participating sites similarly ensure fair participant selection, protect the privacy of research participants, and obtain meaningful informed consent. During the study, participants will have their well-being monitored, and to the fullest extent possible, their privacy protected. Participants may withdraw from the study at any time.

- Prior to implementation, CoVPN trials are rigorously reviewed by scientists who are not involved in the conduct of the trials under consideration.

- CoVPN trials are reviewed by local and national regulatory bodies and are conducted in compliance with all applicable national and local regulations.

- The CoVPN designs its research to minimize risk and maximize benefit to both study participants and their local communities. For example, CoVPN protocols provide enhancement of participants' knowledge of SARS-CoV-2 and SARS-CoV-2 prevention, as well as counseling, guidance, and assistance with any social impacts that may result from research participation. CoVPN protocols also include careful medical review of each research participant's health conditions and reactions to study products while in the study.

- CoVPN research aims to benefit local communities by directly addressing the health and SARS-CoV-2 prevention needs of those communities and by strengthening the capacity of the communities through training, support, shared

knowledge, and equipment. Researchers involved in CoVPN trials are able to conduct other critical research in their local research settings.

- The CoVPN values the role of in-country Institutional Review Boards (IRBs), Ethics Committees (ECs), and other Regulatory Entities (REs) as custodians responsible for ensuring the ethical conduct of research in each setting.

- All participants will receive vaccine during this trial.

- If a participant is diagnosed with COVID-19 during the course of the study, country-specific guidelines on administration of vaccination if available will be followed.

- All participants with COVID-19 will receive the optimal local standard of care for prevention and treatment of COVID-19, including access to temperature monitoring, pulse oximetry, oxygen, medications such as steroids, and hospitalization at facilities that are both prepared and experienced at the management of severe cases to minimize mortality.

- All participants who are pregnant at any time during the study will be offered HIV counseling and testing if they are not known to be PLWH, and referral for prenatal and obstetrical care. All PLWH will be referred for HIV care if not already in a program.

5 IRB/EC review considerations

US FDA and other US federal regulations require IRBs/ECs/REs to ensure that certain requirements are satisfied on initial and continuing review of research (Title 45, Code of Federal Regulations (CFR), Part 46.111(a) 1-7; 21 CFR 56.111(a) 1-7). The following section highlights how this protocol addresses each of these research requirements. Each CoVPN Investigator welcomes IRB/EC/RE questions or concerns regarding these research requirements.

HCORE is paying for this study through funding to NIAID and the trial is being conducted in countries outside of the United States. Due to this, the trial is subject to both US and local regulations and guidelines on the protection of human research subjects and ethical research conduct. Where there is a conflict in regulations or guidelines, the CoVPN strives toward maximum protection of human research participants.

In compliance with international and local (as appropriate) ICH and/or other GCP guidelines, each research location has a locally based Principal Investigator (PI) who is qualified to conduct (and supervise the conduct of) the research. The investigators take responsibility for the conduct of the study, including obtaining all appropriate regulatory and ethical reviews of the research.

5.1 Minimized risks to participants

45 CFR 46.111 (a) 1 and 21 CFR 56.111 (a) 1: Risks to subjects are minimized.

This protocol minimizes risks to participants by (a) correctly and promptly informing participants about risks so that they can join in partnership with the researcher in recognizing and reporting harms; (b) respecting local/national blood draw limits; (c) performing direct observation of participants postvaccination; (d) having staff properly trained in administering study procedures that may cause physical harm or psychological distress, such as blood draws, vaccinations, COVID-19 testing and counseling; (e) providing safety monitoring; (f) ensuring any participant with COVID-19–like symptoms have access to optimal local standard of care for prevention of acquisition and treatment of COVID-19; and (g) participants are permitted to get vaccinated outside the study if they have access.

5.2 Reasonable risk/benefit balance

45 CFR 46.111(a) 2 and 21 CFR 56.111(a) 2: Risks to subjects are reasonable in relation to anticipated benefits, if any, to subjects, and

the importance of the knowledge that may reasonably be expected to result.

In all public health research, the risk-benefit ratio may be difficult to assess because the benefits to participants in prevention protocols are not as apparent as they would be in treatment protocols, where a study participant may be ill and may have exhausted all conventional treatment options. However, this protocol is designed to minimize the risks to participants while maximizing the potential value of the knowledge it is designed to generate.

5.3 Equitable participant selection

45 CFR 46.111 (a) 3 and 21 CFR 56.111 (a) 3: Subject selection is equitable

This protocol has specific inclusion and exclusion criteria for investigators to follow in admitting participants into the protocol. Participants are selected because of these criteria and not because of positions of vulnerability or privilege. Investigators are required to maintain screening and enrollment logs to document participants who screened into and out of the protocol and for what reasons.

5.4 Appropriate informed consent

45 CFR 46.111 (a) 4 and 5 and 21 CFR 56.111 (a) 4 and 5: Informed consent is sought from each prospective subject or the subject's legally authorized representative as required by 45 CFR 46.116 and 21 CFR Part 50; informed consent is appropriately documented as required by 45 CFR 46.117 and 21 CFR 50.27

The protocol specifies that informed consent must be obtained before any study procedures are initiated and assessed throughout the trial (see Section 9.1). Each site is provided training in informed consent by the CoVPN and is required to have a Standard Operating Procedure (SOP) on the informed consent process. The CoVPN requires a signed consent document for documentation, in addition to chart notes or a consent checklist.

5.5 Adequate safety monitoring

45 CFR 46.111 (a) 6 and 21 CFR 56.111 (a) 6: There is adequate provision for monitoring the data collected to ensure the safety of subjects.

This protocol has extensive safety monitoring in place (see Section 11). Safety is monitored daily by CoVPN clinical staff and routinely by the CoVPN 3008

(Ubuntu) PSRT. In addition, an independent DSMB periodically reviews study data, including unblinded study data if/when needed.

5.6 Protect privacy/confidentiality

45 CFR 46.111 (a) 7 and 21 CFR 56.111 (a) 7: There are adequate provisions to protect the privacy of subjects and maintain the confidentiality of data.

Privacy refers to an individual's right to be free from unauthorized or unreasonable intrusion into his/her private life and the right to control access to individually identifiable information about him/her. The term "privacy" concerns research participants or potential research participants as individuals whereas the term "confidentiality" is used to refer to the treatment of information about those individuals. This protocol respects the privacy of participants by informing them about who will have access to their personal information and study data (see Appendix A). The privacy of participants is protected by assigning unique identifiers in place of the participant's name on study data and specimens. In addition, each staff member at each study site in this protocol signs an Agreement on Confidentiality and Use of Data and Specimens with the CoVPN. In some cases, a comparable confidentiality agreement process may be acceptable. Each study site participating in the protocol is required to have a standard operating procedure on how the staff members will protect the confidentiality of study participants.

5.7 Pregnant participants

45 CFR 46.203 and 45 CFR 46.204: There are adequate protections for pregnant women, human fetuses, and neonates involved in research.

The NIH is mandated by law to ensure that pregnant women and children be included in clinical research when appropriate. This study responds to that mandate and will provide valuable research data on the clinical and immunologic outcomes in pregnant women. Per the US Code of Federal Regulations (CFR), site IRBs/ECs must consider the potential risks and benefits to maternal and fetal participants as described in 45 CFR 46 Subpart B (for pregnant women, fetuses, and neonates). In addition to the US regulations cited above, sites not in the US must also comply with all applicable local and national guidelines and regulations.

6 Statistical considerations

6.1 Summary of statistical design

The statistical design primarily aims to evaluate 1) in the pre-Month 6 stage, the relative risk of COVID-19 and severe COVID-19 between the PLWH vaccine-only immunity group who are SARS-CoV-2 negative at enrollment, and receive 2 doses of pre-Month 6 vaccinations, and the PLWH hybrid immunity group who are SARS-CoV-2 positive at enrollment and receive 1 dose of pre-Month 6 vaccination and 2) in the post-Month 6 stage the relative risk of COVID-19 and severe COVID-19 between the mRNA-1273 and mRNA-1273.222 arms. In the pre-Month 6 stage, the evaluation considers follow-up after enrollment until the Month 6 dose; in the post-Month 6 stage, the evaluation considers follow-up after the Month 6 dose until the end of study.

Severe COVID-19 is included as a coprimary endpoint, which recognizes the importance of evaluating severe COVID-19 in PLWH. Given uncertainty in the rate of severe COVID-19 at the time of the primary analysis, an adaptive decision based on the treatment-blinded number of severe COVID-19 endpoints is made of whether to retain severe COVID-19 as a primary endpoint or to change it to a secondary endpoint. In addition, as secondary objectives of this trial, the relative risk of COVID-19 and severe COVID-19 will be evaluated in Groups 1 and 3 combined vs. Groups 2 and 4 combined, accounting for baseline HIV-1 serostatus.

For the pre-Month 6 stage, given that the analysis groups defined by HIV and SARS-CoV-2 statuses, and the number of vaccinations received are not randomized, and the assignment of individuals to study groups is known to participants and study staff, the study objectives are assessed using methods that adjust for measured potential confounder variables (see Section 6.4.3). For the post-Month 6 stage, because participants were randomized at baseline to the assignment of the two different Month 6 vaccinations within each of Groups 1-4, adjustment for potential confounder variables is only considered for the assessment of secondary objectives, not the primary objectives.

6.2 Definitions of study cohorts for analysis

Throughout the protocol, unless otherwise noted, baseline SARS-CoV-2 negative indicates both seronegative and virus detection negative, and SARS-CoV-2 positive indicates either seropositive or virus detection positive of participants' samples collected prior to the first vaccination. Of note, baseline SARS-CoV-2 sero-status that does not account for virus detection results is used to determine treatment group assignment, not analyses cohorts. The definitions of study cohorts that will be analyzed for addressing various study objectives are provided in Table 6-1 below. The Full Analysis Set (FAS) and the Safety Subset (SS) will be the cohorts for the primary safety analyses according to the treatment that participants

actually received. The subgroups of FAS participants will be the cohort for the pre-Month 6 stage primary efficacy analyses. The subgroups of Per-Protocol (PP) participants will be the cohort for the post-Month 6 stage primary efficacy analyses. The subgroups of PP participants that are sampled for cellular and humoral immunogenicity and immune correlates analyses comprise the Immunogenicity Subset (IS). In addition to those measured at Month 7 (one month after the Month 6 dose) across all groups in the IS, immune responses at Month 1 (one month after dose 1) among baseline SARS-CoV-2 seropositive participants in Groups 2 and 4 and immune responses at Month 2 (one month after the second dose) among baseline SARS-CoV-2 seronegative participants in Groups 1 and 3 will be used to characterize vaccine-induced immunogenicity proximal to vaccination. Immunogenicity at baseline (Month 0) and at the Month 6 visit immediately prior to the Month 6 dose, and durability of immunogenicity at Month 12 will also be assessed across all groups in the IS.

Table 6-1 Analysis Cohorts and Corresponding Primary, Secondary or Exploratory Objectives

Cohort	Definition	Objectives
**Full Analysis Set (FAS)**	All randomized participants with ≥ 1 administration of study product	Primary objective 5
• Subgroup FAS.Grp1	• PLWH who are SARS-CoV-2 negative[1] at baseline (Group 1) in the FAS	Primary objectives 1 & 2<br>Secondary objectives 1-2, 10<br>Exploratory objectives 1-5
• Subgroup FAS.Grp2	• PLWH who are SARS-CoV-2 positive[2] at baseline (Group 2) in the FAS, who received either 1 or 2 pre-Month 6 vaccination doses	Primary objectives 1 & 2<br>Secondary objectives 1-2, 10<br>Exploratory objectives 1-5
• Subgroup FAS.Grp3	• People who are HIV-1 seronegative and SARS-CoV-2 negative[1] at baseline (Group 3) in the FAS	Secondary objectives 2, 10<br>Exploratory objectives 1-5
• Subgroup FAS.Grp4	• People who are HIV-1 seronegative and SARS-CoV-2 positive[2] at baseline (Group 4) in the FAS, who received either 1 or 2 pre-Month 6 vaccination doses	Secondary objectives 2, 10<br>Exploratory objectives 1-5
**Safety Subset (SS)**	Participants in the FAS who are sampled for solicited and unsolicited AE assessment	Primary objective 5
. Subgroups SS.Grp1–4	1. Groups 1-4 participants in the SS	Primary objective 5
**Per-Protocol (PP)**	Participants in the FAS who received all planned immunizations according to the current study schema and have no major protocol violations[3]	Primary objectives 3 & 4
• Subgroup PP.Grp1	• Group 1 PP participants who are SARS-CoV-2 negative[1] at baseline	Secondary objectives 3-8<br>Exploratory objectives 1-5
• Subgroup PP.Grp2	• Group 2 PP participants who are SARS-CoV-2 positive[2] at baseline, receiving 1 or 2 pre-Month 6 vaccination doses	Secondary objectives 3-5, 8<br>Exploratory objectives 1-5
• Subgroup PP.Grp3	• Group 3 PP participants who are SARS-CoV-2 negative[1] at baseline	Secondary objectives 4-8<br>Exploratory objectives 1-5
• Subgroup PP.Grp4	• Group 4 PP participants who are SARS-CoV-2 positive[2] at baseline, receiving 1 or 2 pre-Month 6 vaccination doses	Secondary objectives 4-5, 8<br>Exploratory objectives 1-5
**Immunogenicity Subset (IS)**	Participants who are sampled into the subset for measuring humoral immunologic markers[4] at Months 0, 1 (Groups 2 and 4 only), 2 (Groups 1 and 3 only), 6, 7 and 12.	Secondary objective 9<br>Exploratory objectives 4-5
• Subgroups IS.Grp1–4	• Groups 1-4 participants in the IS	
• Subgroup IS.PBMC.Imm.Grp1–4	• Groups 1-4 participants in the IS who are also sampled into the PBMC Immunogenicity subset for measuring cellular immunologic markers at Months 0, 1 (Groups 2 and 4 only), 2 (Groups 1 and 3 only), 6, 7 and 12.	
• Subgroup IS.PBMC.Cor.Grp1-2	• Groups 1 and 2 participants in the IS who are also sampled into the PBMC Immune Correlates subset for measuring cellular immunogenicity markers at Months 0, 1 (Group 2 only), 2 (Group 1 only), and 7.	

[1] SARS-CoV-2 negative is defined by both seronegative and virus detection negative or by seronegative alone if missing virus detection test results.
[2] SARS-CoV-2 positive is defined by seropositive and/or virus detection positive.
[3] Major protocol violations are protocol deviations that compromise study integrity; for example, protocol deviations related to collection or reporting of data necessary to interpret the primary efficacy endpoint.
[4] Immunologic markers measured will include binding antibody and neutralizing antibody markers.

6.3 Accrual and sample size

This study will enroll up to 15,600 participants into four groups defined by baseline HIV-1 and SARS-CoV-2 sero-statuses and randomized to receive the same vaccination series of mRNA-1273 at month 0 and month 1 (if applicable), but two different vaccines (mRNA-1273 or mRNA-1273.222) at month 6 at a 1:1 randomization ratio within each group as shown in the study schema. The sample sizes of Groups 1 and 2 are driven by the total number of COVID-19 cases that are needed to achieve sufficient statistical power to evaluate the relative risk of COVID-19 between Groups 1 and 2 in the pre-Month 6 stage, as well as between randomized interventions at Month 6 within each group in the post-Month 6 stage. The sample sizes of Groups 3 and 4 are primarily driven by the precision to characterize safety and immunogenicity of the regimens in each group of participants while keeping the same 1:2 ratio between the SARS-CoV-2 negative and positive groups as for Groups 1 and 2.

6.3.1 Power calculations for coprimary objectives 1-4 (Efficacy)

Let VE(COVID) and VE(Severe COVID) denote vaccine efficacy of the vaccine regimen vs. counterfactual placebo among baseline SARS-CoV-2 negative participants against COVID-19 and severe COVID-19, respectively. The relative risk objectives are assessed in terms of cumulative incidence ratios between the active vaccine regimens without analysis of counterfactual placebo arms; the notations VE(COVID) and VE(Severe COVID) are mainly used for purposes of communicating effect sizes.

The following calculations focus on the COVID-19 endpoint because the severe COVID-19 endpoint could potentially be changed to be a secondary endpoint if there are insufficient severe COVID endpoints (Section 6.4.3). For the assessment of pre-Month 6 stage objectives, sample sizes of Groups 1 and 2 in the FAS are considered. For the assessment of post-Month 6 stage objectives, the following calculations assume that approximately 4200 participants in Groups 1-4 will receive their Month 6 vaccination under the current protocol version. This assumption is consistent with study data as of January 2023. As shown in the upper portion (pre-Month 6 stage) of Table 6-2, there is ample power (> 90%) to detect a relative risk of 2.0 between Groups 1 and 2 against the null hypothesis H0: RR = 1.0 during Month 0 and 6 in the pre-Month 6 stage. In reality, the sample sizes of the PLWH hybrid immunity group who receive 1 pre-Month 6 dose and the PLWH pure vaccine group who receive 2 pre-Month 6 doses are likely smaller than the sample sizes of Groups 1 and 2, respectively. As shown in the lower portion (post-Month 6 stage) of Table 6-2, there is approximately 90% power to detect a relative risk of 2.5 – 3.0 between mRNA-1273 vs. mRNA-1273.222 vaccination during Month 6.5 and 12 in the post-Month 6 stage.

These calculations are carried out assuming 5% annual dropout, 5% probability of missing any of the 3 doses, VE(COVID) of 63% between Month 0 and Month 6 in Group 1 (SARs-CoV-2 negative) and in Group 2, and 5% six-month COVID-19

incidence rate among unvaccinated PLWH SARs-CoV-2 negative individuals in the community. The assumption on VE(COVID) of 63% between Month 0 and Month 6 accounts for a possible reduced protection of the vaccine regimen in PLWH against the SARS-CoV-2 variant at the time of the trial conduct. The 5% six-month background incidence rate assumption was based on the observed 4.7%, 5.25%, and 10.38% placebo arm six-month incidence rates among baseline SARS-CoV-2 negative participants in, respectively, the AstraZeneca phase 3 (149), Novavax phase 2 (107), and Janssen ENSEMBLE phase 3 (28) trials restricted to South African study sites, and building in conservative margin given the availability of vaccines to potentially reduce transmission and infection in the region. The background SARS-CoV-2 (re-infection) incidence rate among unvaccinated PLWH SARS-CoV-2 positive individuals in the community is assumed to be half of that of unvaccinated PLWH SARS-CoV-2 negative individuals based on the reported protection of approximately 55% against the Omicron variant from previous SARS-CoV-2 infection in South Africa (150-152) and in other countries (153). The statistical power is calculated using an exact binomial test with a (two-sided) false positive error rate of 0.05.

Table 6-2 Pre-Month 6 stage: power to detect relative risk (Months 0-6) being different from 1.0 between Group 1 (n=4200) and Group 2 (n=8400).

True VE (0-6) of Group 1 (n=4200)	Relative Risk (Groups 1 vs. 2)	Total expected number of events M0-6	Power
**60%**	1.3	189	45%
	1.6	176	85%
	**2.0**	**167**	**99%**
**70%**	1.2	149	17%
	1.5	135	59%
	**2.0**	**125**	**96%**
70%	1/1.3	207	45%
75%	1/1.6	203	85%
**80%**	**1/2.0**	**200**	**99%**
75%	1/1.2	159	17%
80%	1/1.5	153	59%
**85%**	**1/2.0**	**150**	**96%**

Post-Month 6 stage: power to detect relative risk (Months 6.5-12) being different from 1.0 between mRNA-1273 vs. mRNA-1273.222 Month 6 vaccination (n=4200, 2100 vs. 2100), assuming the rest of participants either dropped out of the study early or received their Month 6 vaccination under the previous protocol version(s).

Annual incidence for mRNA-1273 Month 6 vaccination arm	Relative Risk (mRNA-1273 vs. mRNA-1273.222)	Total expected number of events M6.5-12	Power
3.5%	2.5	54	89%
	3.0	55	96%
3.0%	2.5	47	84%
	3.0	47	94%
**2.5%**	**2.5**	**39**	69%
	3.0	**39**	85%

In addition, Table 6-3 provides the precision of the relative risk estimates with different numbers of endpoints accrued in the post-Month 6 stage.

Table 6-3 Post-Month 6 Stage: precision in relative risk (RR) estimates with 50, 100 or 150 total endpoints accrued between Month 6.5 and Month 12.

Estimate RR of COVID-19 (mRNA-1273 vs. mRNA-1273.222)	95% CI of RR		
	50 endpoints	100 endpoints	150 endpoints
1.0	(0.57, 1.74)	(0.68, 1.48)	(0.73, 1.38)
1.25	(0.72, 2.18)	(0.84, 1.85)	(0.91, 1.72)
1.5	(0.85, 2.64)	(1.00, 2.24)	(1.08, 2.08)
2.0	(1.11, 3.60)	(1.32, 3.03)	(1.42, 2.81)
2.5	(1.36, 4.62)	(1.62, 3.86)	(1.75, 3.56)
3.0	(1.58, 5.69)	(1.91, 4.72)	(2.07, 4.34)

6.3.2 Power calculations for coprimary objective 5 (Safety)

For pre-Month 6 stage, we consider power for evaluating the safety objective using a total of N = 15,600 participants in the Full Analysis Set (FAS) and N = 1,500 in the Safety Subset (SS). If no SAE or AESI that is considered related to the study vaccine is observed in the FAS, or no AE in the SS, the upper limit of the 95% confidence interval (CI) for the true event rate will be 0.02% or 0.2%, respectively. Hence, observing no SAE/AESI in the FAS or no AE in the SS would provide us with 95% confidence that the true event rate is no more than 0.02% or 0.2%, respectively. For post-Month 6 stage, we consider power for evaluating the safety objective of the mRNA-1273 or mRNA-1273.222 vaccination at Month 6 using half of the cohorts assessed for pre-Month 6 stage and additionally assuming 80% of these cohorts receiving the Month 6 vaccination under the current protocol version. Table 6-4 shows the 95% CIs for event rates ranging from 0% to 5% in the full or half of the FAS, FAS.Grp1-4 and SS, demonstrating the degree of precision conferred by the trial size for evaluating safety events in these cohorts. CIs were calculated using the Clopper-Pearson method (154).

Table 6-4 Pre-Month 6 stage: Confidence intervals (CIs) for observed safety event rates in the FAS (N = 15,600), FAS.Grp1-4 (N = 4,200, 8,400, 1,000, 2,000) and the SS (N = 1500) cohorts concerning the mRNA-1273 regimen. Confidence intervals were calculated using the Clopper-Pearson method (154).

Safety Event Rate (%)	FAS (N = 15,600)	FAS.Grp1 (N = 4,200)	FAS.Grp2 (N = 8,400)	FAS.Grp3 (N = 1,000)	FAS.Grp4 (N = 2,000)	SS (N = 1,500)
	CI (%)	CI (%)	CI (%)	CI (%)	CI (%)	CI (%)
0.0	(0.0, 0.0)	(0.0, 0.1)	(0.0, 0.0)	(0.0, 0.4)	(0.0, 0.2)	(0.0, 0.2)
0.5	(0.4, 0.6)	(0.3, 0.8)	(0.4, 0.7)	(0.2, 1.2)	(0.2, 0.9)	(0.2, 1.0)
1.0	(0.8, 1.2)	(0.7, 1.3)	(0.8, 1.2)	(0.5, 1.8)	(0.6, 1.5)	(0.6, 1.6)
2.0	(1.8, 2.2)	(1.6, 2.5)	(1.7, 2.3)	(1.2, 3.1)	(1.4, 2.7)	(1.4, 2.8)
3.0	(2.7, 3.3)	(2.5, 3.6)	(2.6, 3.4)	(2.0, 4.3)	(2.2, 3.8)	(2.2, 4.0)
4.0	(3.7, 4.3)	(3.4, 4.6)	(3.6, 4.4)	(2.9, 5.4)	(3.2, 5.0)	(3.1, 5.1)
5.0	(4.7, 5.4)	(4.4, 5.7)	(4.5, 5.5)	(3.7, 6.5)	(4.1, 6.0)	(4.0, 6.2)

Post-Month 6 stage: Confidence intervals (CIs) for observed safety event rates in half of the FAS (N = 0.8*15,600/2), FAS.Grp1-4 (N = 0.8*4,200/2, 0.8*8,400/2, 0.8*1,000/2, 0.8*2,000/2) and the SS (N = 0.8*1500/2) cohorts concerning each type of Month 6 vaccination (mRNA-1273 or mRNA-1273.222), assuming 80% of these cohorts receiving the Month 6 vaccination under the current protocol version. Confidence intervals were calculated using the Clopper-Pearson method (154).

Safety Event Rate (%)	N = 6,240	N = 1,680	N = 3, 360	N = 400	N = 800	N = 600
0.0	(0.0, 0.1)	(0.0, 0.2)	(0.0, 0.1)	(0.0, 0.9)	(0.0, 0.4)	(0.0, 0.6)
0.5	(0.3, 0.7)	(0.2, 1.0)	(0.3, 0.8)	(0.1, 1.8)	(0.1, 1.3)	(0.1, 1.5)
1.0	(0.8, 1.3)	(0.6, 1.6)	(0.7, 1.4)	(0.3, 2.5)	(0.4, 2.0)	(0.4, 2.2)
2.0	(1.7, 2.4)	(1.4, 2.8)	(1.6, 2.5)	(0.9, 3.9)	(1.1, 3.2)	(1.0, 3.5)
3.0	(2.6, 3.5)	(2.2, 3.9)	(2.5, 3.6)	(1.6, 5.2)	(1.9, 4.4)	(1.8, 4.7)
4.0	(3.5, 4.5)	(.1, 5.1)	(3.4, 4.7)	(2.3, 6.4)	(2.8, 5.6)	(2.6, 5.9)
5.0	(4.5, 5.6)	(4.0, 6.2)	(4.4, 5.8)	(3.1, 7.6)	(3.6, 6.7)	(3.4, 7.1)

6.4 Statistical analyses

This section describes the primary study analysis. Unless otherwise noted, data from enrolled participants will be analyzed according to the initial randomization assignment regardless of how many vaccinations they received.

Analyses for primary endpoints will be performed using SAS and R. All other descriptive and inferential statistical analyses will be performed using SAS, StatXact, or R statistical software.

No formal multiple-comparison adjustments will be employed for multiple safety endpoints or multiple efficacy endpoints. Multiple comparisons adjustment may be performed for secondary endpoints and objectives, which will be specified in the Statistical Analysis Plan (SAP).

6.4.1 Analysis variables

The analysis variables consist of baseline participant characteristics, safety, efficacy, immunogenicity, and COVID-19 disease, severe COVID-19 and SARS-CoV-2 infection diagnosis variables for the primary and secondary objective analyses.

6.4.2 Baseline comparability

Treatment arms will be compared for baseline participant characteristics using descriptive statistics.

6.4.3 Analysis of relative risk between treatment arms

For the assessment of the coprimary efficacy objectives, this study uses two approaches for determining symptomatic COVID-19 cases, as previously defined in Section 3. For survival data analyses, the time of all participant-level failure and

censoring events are reported relative to the time of enrollment (ie, first vaccination) for the pre-Month 6 stage objectives, and relative to the time of Month 6 dose for post-Month 6 stage. For the COVE-based endpoint definition, the time of diagnosis of COVID-19 and severe COVID-19 will be determined by the EAC adjudicators. For the CDC criteria-based definition, the time of diagnosis of lab-confirmed clinical COVID-19 is defined as the minimum date of reported qualifying COVID-19 symptoms and collection date of SARS-CoV-2 positive result (both must occur), and the time of diagnosis of severe COVID-19 to be the minimum date of reported qualifying severe COVID-19 symptoms and collection date of SARS-CoV-2 positive result (both must occur).

The time of diagnosis of the SARS-CoV-2 infection endpoint is the draw time of the first sample with a positive test result by either antibody seroconversion assays or by virus-detection via NAAT. The serology assays are used to ascertain infections occurred anytime during the study among baseline SARS-CoV-2 negative (NAAT and serology negative) participants to address Secondary objectives 1-8. The NAAT are used to ascertain recent or ongoing infections among both baseline SARS-CoV-2 negative and positive participants to address exploratory objectives. The time of diagnosis of the asymptomatic endpoint is the draw time of the first sample with a positive test result by virus detection assays or seroconversion with no reported COVID-19–like illness episodes between enrollment and 14 days after the timepoint at which SARS-CoV-2 infection is ascertained. In case of reinfection of SARS-CoV-2, the first occurrence of a given endpoint is considered.

In the primary and most secondary efficacy analyses, the failure times of participants never observed to be diagnosed with a given clinical endpoint will be right censored at the date of last contact at which endpoint status was assessed or study termination. Participants who report outside COVID-19 vaccines will be censored at the time of receiving such vaccination. Except where otherwise specified, efficacy analyses of a given endpoint that is counted starting at some specified number of days postvaccination will exclude all participants from the analysis who experienced the particular endpoint diagnosed before the counting period.

The statistical analyses described below use the COVID-19 and severe COVID-19 case definition. Exploratory efficacy analyses may also be considered for a new definition of the endpoints that include suspected but unconfirmed COVID-19 cases.

The primary efficacy analyses focusing on the pre-Month 6 stage will be carried out between the FAS.Grp1 who are SARS-CoV-2 negative at baseline and receive 2 doses of pre-Month 6 vaccination, and FAS.Grp 2 who are SARS-CoV-2 positive at baseline and receive 1 dose of pre-Month 6 vaccination, counting endpoints >= 1 day post–dose 1. The primary efficacy analyses focusing on the post-Month 6 stage will be carried out between the two different Month 6 arms across all groups, counting endpoints ≥ 14 days post the month 6 vaccination.

Secondary efficacy analyses will also entail counting endpoints ≥ 14 days post the primary vaccination series and endpoints ≥ 1 day post the first dose at month 0 in the pre-Month 6 stage, as well as ≥ 14 days post the month 6 dose in Groups 1 and 3 combined vs. Groups 2 and 4 combined.

The relative risk (of COVID-19, severe COVID-19, SARS-CoV-2 infection, or SARS-CoV-2 asymptomatic infection) is defined as the incidence rate ratio between the 2 treatment groups in the pre-Month 6 stage or between the 2 treatment arms within a group in the post-Month 6 stage. When there are at least 7 severe COVID-19 endpoints at the primary analysis for a given relative risk assessment, both COVID-19 and severe COVID-19 are used as coprimary endpoints. Otherwise, severe COVID-19 is considered as a secondary endpoint. For the final analyses of the pre-Month 6 objectives, the incidence will be compared across groups by estimating the marginalized cumulative incidence of the endpoint overtime for each group, using a causal inference method that adjusts for potential confounders and accounts for right-censoring of failure times. Under causal assumptions, the marginalized cumulative incidence equals the causal cumulative incidence, interpreted as cumulative incidence if, hypothetically, the entire study population were assigned to the given group under consideration. Sensitivity analyses may also be conducted to account for unobserved confounding factors. For the final analyses of the post-Month 6 objectives, the RR between treatment arms within each group will also be estimated. The causal inference approach used for the pre-Month 6 stage analysis may also be applied for estimating the relative risk of each endpoint for the post-Month 6 dose objectives. More details will be described in the SAP. For analyses before the final analyses, the RR may be estimated from a Poisson regression analysis for both pre- and post-Month 6 stages. To assess potential time effects of vaccine efficacy, the Nelson-Aalen method will be used to plot the estimated cumulative incidence rates over time for the comparison groups. Additional analysis of RR by specified viral strain categories may also be performed using competing risks failure time methods, as detailed in the SAP.

6.4.4 Safety/Tolerability analysis

Safety endpoints will be assessed in the FAS cohort. Since enrollment is concurrent with receiving the first injection, all participants will have received at least 1 vaccination and therefore will provide some safety data.

During the course of the trial, analyses of safety data will be prepared for review by the DSMB at a frequency determined by the DSMB for as long as it is involved in the trial. Ad hoc safety reports may also be prepared for DSMB review at the request of the PSRT. The OG must approve any other requests for safety data prior to the end of the scheduled follow-up visits.

6.4.4.1 AEs and SAEs

AEs will be summarized using MedDRA System Organ Class and preferred terms. Tables will show by treatment group the number and percentage of participants experiencing an AE within a System Organ Class or within preferred-term category by severity and by relationship to study product. For the calculations in these tables, a participant with multiple AEs within a category will be counted once under the maximum severity and by causal relationship to study product. Formal statistical testing comparing arms is not planned for all AEs since interpretation of differences must rely heavily upon clinical judgment. Barnard's tests will be used to compare rates of SAEs deemed related to study products between vaccine arms. The false discovery rate procedure of Mehrotra and Heyse (155) may be applied to compare grouped systemic SAEs divided by body systems between treatment arms.

A listing of SAEs will provide details of the events including severity, relationship to study product, time between onset and last vaccination, and number of vaccinations received. A separate listing will do the same for AESIs. AESIs for this protocol include but are not limited to potential immune-mediated disorders.

6.4.4.2 Reasons for Vaccination Discontinuation and Early Study Termination

The number and percentage of participants who discontinue vaccination and who terminate the study early will be tabulated by reason and treatment arm.

6.4.5 Immunogenicity and immune correlates analyses

Data from quantitative immunogenicity assays will be summarized for each treatment group using positive response rates and geometric means with 95% CIs, for each timepoint for which an assessment is performed. Data from qualitative (ie., yielding a positive or negative result) assays will be summarized by tabulating the frequency of positive responses for each assay by group at each timepoint that an assessment is performed. Analyses focus on the key immunogenicity timepoints postvaccination (M1, M2, or M7), the M0 prevaccination timepoint, and the change in marker value between the 2 timepoints. The analyses will be conducted in the Immunogenicity Subset, evaluating subgroups of participants defined by baseline HIV-1 and SARS-CoV-2 statuses. A separate Marker SAP will describe the complete set of immunogenicity analyses.

The separate Marker SAP will describe the statistical methods and data analysis implementations for assessing immune response markers as correlates of the COVID-19, severe COVID-19, and infection study endpoints, as well as various types of correlates/surrogates of protection. Objectives include characterizing the distribution of immunologic markers at the approximate peak immunogenicity timepoint and over time (at Months 0, 1 or 2, 6, 7 and 12); assessing these markers at the peak immunogenicity timepoint and over time as correlates of risk of COVID-19, severe COVID-19, and of infection using data from all vaccine

recipients; and assessing these markers at the peak immunogenicity timepoint and over time as various types of correlates of protection, using data from both vaccine arms. The biomarkers are measured in the Immunogenicity Subset (IS) and in infection cases outside the IS. These cases include all primary endpoint cases and asymptomatic infection endpoint cases. Sampling of participants into the IS is stratified by treatment arm and the randomization strata (except study site, although geographic region is accounted for by including South Africa and non-South Africa as sampling strata factors). In addition, PBMC may be stored at Months 0, 1 or 2, 6, 7 and 12 from a subset of participants at selected sites to enable study of B- and T-cell markers as correlates of risk and protection (see Laboratory Procedures for PBMC Immunogenicity subset (Appendix D and Appendix E) and PBMC Immune Correlates subset (Appendix F and Appendix G).

6.4.6 Sieve analysis

SARS-CoV-2 Spike sequences will be determined for all COVID-19 primary endpoints, based on the first available NAAT positive sample starting at COVID-19 diagnosis. SARS-CoV-2 Spike sequences will also be determined for NAAT positive samples for asymptomatic infection cases. Statistical analyses will assess whether and how vaccine efficacy depends on Spike amino acid sequence features, with features of interest including recognized genotypes/variants of interest/concern/high consequence, Hamming distances from RBD, NTD, all of S1 subunit, S2 subunit, and all of Spike to the vaccine insert sequence, whether residues at amino acid positions are mismatched to the corresponding positions in the vaccine insert sequence, and whether haplotypes, such as 9-mers or antibody-relevant constellations of AA positions, are mismatched to the vaccine insert sequence. In addition, based on the Spike sequence from each COVID-19 primary endpoint, a pseudovirus may be made and tested in 1 or more selected neutralization assays to characterize the neutralization sensitivity of the infecting virus to vaccinee sera. The neutralization sensitivity phenotype will be defined using a set of immunogenicity timepoint (eg, Months 2 and 7) serum samples from a sample of vaccine recipients in the Immunogenicity Subset. Neutralization sieve analysis will assess whether and how vaccine efficacy depends on virus neutralization sensitivity measures, such as geometric mean serum ID50. Sieve analysis will be done for each of the coprimary endpoints, with the severe COVID-19 endpoint only analyzed if there are sufficient numbers of cases. Sieve analysis may be done for the infection endpoint and the asymptomatic infection endpoint. Details of the sieve analysis are described in the Marker SAP.

6.5 Operational monitoring for quality of trial conduct

The study OG, protocol team and study investigators will monitor the quality of trial conduct related to achievement of targeted levels of various operational metrics, such as retention, and adherence to study interventions. Detailed operational metrics will be specified in the OG charter.

6.6 Final analyses timing

The final analyses of the study objectives in the pre-Month 6 will be performed when all participants who remain in the study have had the Month 6 dose in the pre-Month 6 stage. The final analyses of the study objectives in the post-Month 6 stages and in the entire study period will be performed when all participants who remain in the study reach their end of follow-up in the post-Month 6 stage.

6.7 Roles of the DSMB, OG, and EAC

OG oversight of the trial will continue, and adaptations in study design and modifications will be made under its recommendations. As the main objectives of the study have been achieved, the frequency of DSMB meetings is under discussion.

An Endpoint Adjudication Committee (EAC) will be assembled for the purpose of reviewing potential cases per COVE criteria to determine if these criteria for the primary and secondary endpoints have been met. More details on the composition, role of and the data provided to the EAC will be described in the EAC charter.

6.8 Randomization implementation

The randomization sequence will be computer generated and provided by the data monitoring center (DMC) to each CRS, ideally through a Web-based randomization system. For statistical efficiency and to avoid chance imbalance in exposure or prognostic factors between treatment groups, the randomization will be stratified by study site, HIV-1 infection status, and SARS-CoV-2 serostatus at enrollment. The randomization will be done in blocks to ensure balance between the vaccine arms within levels jointly determined by these factors. The block size ensures balance among treatment arms over different epidemiological stages of the pandemic. At each institution, the pharmacist with primary responsibility for dispensing study products is charged with maintaining security of the treatment assignments.

7 Selection and withdrawal of participants

Participants will be adults who comprehend the purpose of the study and have provided written informed consent in their language of choice. Participants will be recruited and screened; those determined to be eligible, based on the inclusion and exclusion criteria, will be enrolled in the study. Final eligibility determination will depend on information available at the time of enrollment, medical history questionnaire, physical examinations, and answers to self-administered and/or interview questions.

Investigators should always use good clinical judgment in considering a participant's overall fitness for trial participation. Some participants may not be appropriate for enrollment even if they meet all inclusion/exclusion criteria. Medical, psychiatric, occupational, or other conditions may make evaluation of safety and/or immunogenicity difficult, and some participants may be poor candidates for retention.

Determination of eligibility, taking into account all inclusion and exclusion criteria, must be made within 56 days prior to enrollment unless otherwise noted below.

7.1 Inclusion criteria

General and Demographic Criteria

1. Age ≥ 18 years if participant self-reports living with HIV or another comorbidity known to be associated with severe COVID-19, for example (CDC.gov for exhaustive list):

    - Hypertension
    - Type 2 diabetes mellitus
    - Overweight, obese, or severely obese (ie, body mass index [BMI] ≥ 25 kg/m^2)
    - Heart conditions, such as heart failure, coronary artery disease, or cardiomyopathies
    - Chronic kidney disease
    - COPD (chronic obstructive pulmonary disease)
    - Cancer

- Non-HIV immunocompromised state (weakened immune system) or solid organ transplant
- Pregnancy
- Sickle cell disease
- Smoking

2. Willingness to be followed and remain in the catchment area for the planned duration of the study.

3. Ability and willingness to provide **informed consent**.

4. Willingness to discuss HIV infection status, undergo related testing/monitoring labs, and receive counseling and referrals to minimize HIV acquisition/improve HIV care as appropriate based on their infection status.

5. **Assessment of Understanding (AoU):** Participant demonstrates understanding of this study; completes a questionnaire prior to first vaccination with demonstration of understanding of all questionnaire items answered incorrectly.

6. Agrees not to enroll in another interventional study of an investigational research agent until after the study is completed and all the data has been obtained. Enrollment in studies of investigational research agents for the treatment of COVID-19 is allowed for participants who develop COVID-19 disease.

7.2 Exclusion criteria

General

1. **Acutely ill 72 hours prior to or at screening**. Participants meeting this criterion may be rescheduled within the relevant window periods. Participants with minor illnesses can be enrolled at the discretion of the investigator.

2. History of angioedema or anaphylaxis.

Vaccines and other injections

1. Prior receipt of a SARS-CoV-2 vaccine.

2. History of severe allergic reaction to any ingredient of this vaccine (lipids (SM-102, polyethylene glycol [PEG] 2000 dimyristoyl glycerol [DMG], cholesterol, and 1,2-distearoyl-sn-glycero-3-phosphocholine [DSPC]),

tromethamine, tromethamine hydrochloride, acetic acid, sodium acetate trihydrate, and sucrose).

3. **Live attenuated vaccines** received within 30 days before first vaccination (eg, measles, mumps, and rubella [MMR]; oral polio vaccine [OPV]; varicella; yellow fever; live attenuated influenza vaccine, live attenuated zoster vaccine).

4. **Any vaccines that are not live attenuated vaccines** and were received within 14 days prior to first vaccination (eg, tetanus, human papilloma virus (HPV), pneumococcal, Hepatitis A or B).

5. **Blood products,** systemic immunoglobulins, or monoclonal antibodies (including against SARS-CoV-2) received within 90 days before first vaccination.

8 Study products

The protocol schema is shown in Table 1-1.

8.1 Vaccine regimen

Groups 1 and 3 (SARS-CoV-2 negative at baseline)

Treatment 1 (for Groups 1A and 3A): mRNA-1273 vaccine in 100 mcg dose to be administered as an IM injection into the deltoid muscle on Day 1, Day 29 and Day 169 (Months 0, 1, and 6)

Treatment 2 (for Groups 1B and 3B): mRNA-1273 vaccine in 100 mcg dose to be administered as an IM injection into the deltoid muscle on Day 1 and Day 29 (Months 0 and 1)

AND

mRNA-1273.222 vaccine in 100 mcg dose to be administered as an IM injection into the deltoid muscle on Day 169 (Month 6)

Groups 2 and 4 (SARS-CoV-2 positive at baseline)

Treatment 3 (for Groups 2A and 4A): mRNA-1273 vaccine to be administered as an IM injection into the deltoid muscle on Day 1 and Day 169 (Months 0 and 6)

Treatment 4 (for Groups 2B and 4B): mRNA-1273 vaccine to be administered as an IM injection into the deltoid muscle on Day 1 (Month 0)

AND

mRNA-1273.222 vaccine in 100 mcg dose to be administered as an IM injection into the deltoid muscle on Day 169 (Month 6)

8.2 Storage, handling, preparation and dispensing of the study products

Site Pharmacists should consult the CoVPN 3008 (Ubuntu) Pharmacy Manual for instructions on handling, storing, preparing, and dispensing the study products.

8.3 Acquisition of the study products

COVID-19 vaccine (mRNA-1273) and bivalent vaccine (mRNA-1273.222) will be provided by Moderna, Inc. through the US Government HCORE and allocated to the Sponsor for this trial.

Once a study CRS has completed all procedures for study start, study products can be obtained as outlined in the CoVPN 3008 (Ubuntu) Pharmacy Manual.

8.4 Administration of study products

All injections are to be administered intramuscularly into the deltoid muscle by study staff assigned for this procedure.

If an injection cannot be administered in either deltoid muscle due to a medical contraindication, the injection should be administered in an alternate body site (eg, lateral thigh). The appropriate study staff should document this clearly. Under this circumstance, this is NOT a protocol violation.

Vaccination providers will refer to the CoVPN 3008 (Ubuntu) Study-specific Procedures (SSP) for further guidance.

8.5 Pharmacy records and accountability

The CoVPN CRS pharmacist is required to maintain complete records of all study products. The site pharmacist is responsible for maintaining randomization codes and randomization confirmation notices for each participant in a secure manner.

8.6 Final disposal of the study products

All unused study products must be returned to the Sponsor or destroyed per the CoVPN 3008 (Ubuntu) Pharmacy Manual after the study is completed or terminated unless otherwise instructed by the Sponsor.

9 Clinical procedures

Procedures are in place so that study visits may be conducted remotely, such as via phone, text message, email, or other electronic means in lieu of, or in combination with, in-person visits at the CRS. Furthermore, some visit procedures may be conducted outside the CRS.

Direct data entry or direct data capture of study data into the study database is allowed when capturing information from the participant. Study data may also be sourced from electronic or paper source documents prior to being entered into the study database (see the CoVPN 3008 (Ubuntu) SSP).

The schedules of clinical and laboratory procedures are shown in Appendix B, Appendix D, and Appendix H for participants in Groups 1 and 3; Appendix F for participants in Group 1 only; Appendix G for participants in Group 2 only and Appendix C, Appendix E, and Appendix I for participants in Groups 2 and 4.

9.1 Informed consent

Informed consent is the process of working with participants so that they fully understand what will and may happen to them while participating in a research study. The CoVPN informed consent form documents that a participant (1) has been informed about the potential risks, benefits, and alternatives to participation, and (2) is willing to participate in a CoVPN study. Informed consent encompasses all written, verbal, and electronic study information CRS staff provide to the participant before and during the trial. CRS staff will obtain informed consent of participants according to the CRS's SOP on the informed consent process.

If any new information is learned that might affect the participants' decisions to stay in the trial, that information will be shared with trial participants. If necessary, participants will be asked to provide revised informed consent forms.

9.1.1 Protocol-specific consent forms

The protocol-specific consent forms describe the study products to be used and all aspects of protocol participation, including screening and enrollment procedures. A sample protocol-specific consent form is located in Appendix A and an addendum to the informed consent form is located in Appendix L

Each CRS is responsible for developing a protocol-specific consent form(s) for local use based on the sample protocol-specific consent forms. The consent form(s) must be developed in accordance with requirements of the following:

- CRS's IRB/EC and any applicable REs
- CRS's institution

- Elements of informed consent as described in Title 45, CFR Part 46 and Title 21 CFR, Part 50 and in ICH E6(R2), Good Clinical Practice: Consolidated Guidance 4.8

The sample informed consent form includes instructions for developing specific content.

9.1.2 Assessment of Understanding

Study staff are responsible for ensuring that participants fully understand the study before enrolling them. This process involves reviewing the informed consent form with the participant, allowing time for the participant to reflect on the procedures and issues presented, and answering all questions completely.

An Assessment of Understanding (AoU) is used to document the participant's understanding of key concepts in this study. Participants must demonstrate understanding of all questions answered incorrectly. This process and the participant's understanding of the key concepts is to be recorded in source documentation at the site.

9.2 Pre-enrollment procedures

Screening may occur at the same time as enrollment visit. All inclusion and exclusion criteria must be assessed before enrollment.

After the appropriate informed consent has been obtained and before enrollment, the following procedures are performed:

- Targeted medical history documented in the case history record
- Abbreviated physical examination
- Targeted concomitant medications will be assessed for high-risk conditions
- Obtaining of participant demographics in compliance with the NIH Policy on Reporting Race and Ethnicity Data: Subjects in Clinical Research, Aug. 8, 2001 (available at http://grants.nih.gov/grants/guide/notice-files/NOT-OD-01-053.html)
- Urine pregnancy test for all participants who were assigned female sex at birth and are of reproductive potential
- For participants who are confirmed to be pregnant and plan to continue the pregnancy, the following will be performed: (1) Fetal ultrasound to confirm gestational age and developmental status of the pregnancy; (2) Syphilis test result and history of treatment will be collected from medical records if

previously done and documented; otherwise, the participant will be referred for testing and potential treatment per local standard of care and the results will be collected from the subsequent medical records.

- HIV diagnostic test for all participants will be conducted regardless of self-reported status, HIV counseling will be offered, and participants with a diagnosis of HIV will be referred for treatment per local guidelines if not already engaged in care.

- For participants with a new HIV diagnosis, blood will be collected for HIV viral load and CD4 count. If antiretroviral treatment needs to be initiated, enrollment into the study may be deferred until approximately 2 to 4 weeks after treatment is started.

- SARS-CoV-2 antibody point of care serological test will be used to manage the proportions of participants with prior exposure in the study (see Section 6.3).

9.3 Enrollment and vaccination visits

Once a participant has consented to trial participation and is found to meet all eligibility criteria (see Sections 7.1 and 6), the CRS requests the randomization assignment via a Web-based randomization system. Enrollment is simultaneous with first vaccination.

Enrollment visit only:

- Participants will be administered the baseline risk assessment questionnaire.

- Before leaving the clinic, the participant is given the Memory Aid 1 for surveillance of any symptoms of COVID-19 and is instructed on how to use it. See Section 9.5 and CoVPN 3008 (Ubuntu) SSP for details.

- Participants will be provided with a thermometer and will be instructed to take their temperature if they have symptoms.

- Sites will instruct the participant to contact study staff if they develop symptoms of COVID-19 or are hospitalized. The site will make arrangements to be in contact with the participant should they have any AEs or develop symptoms of COVID-19 (as described in Sections 9.4 and 9.5).

- For participants who self-report living with HIV and/or are confirmed to have HIV infection: blood will be collected for HIV viral load and CD4 count.

- Participants who are confirmed to be pregnant at screening will not be included in the PBMC Immunogenicity and PBMC Immune correlates subsets. If a participant becomes pregnant during the study and is already included in these

subsets, blood for cellular assays will not be drawn while the participant is pregnant.

At vaccination visits, the following procedures are performed before vaccination:

- Symptom-directed physical examination if needed; if the participant is clinically ill or has COVID-19 symptoms, vaccination will be delayed until clinically recovered (see Sections 9.5, 9.6, and 9.9 and CoVPN 3008 (Ubuntu) SSP for more information).

- Assessment of targeted concomitant medications (as described in Section 9.2).

- Nasal swabs collection (as described in Appendix B, Appendix C, Appendix D, Appendix E, Appendix F, Appendix G, Appendix H, Appendix I, and Appendix J).

- Blood collection (as described in Appendix B, Appendix C, Appendix D, Appendix E, Appendix F, Appendix G, Appendix H, Appendix I, and Appendix J).

- Participants will be instructed to contact the clinic if they have any AEs to report.

- Urine pregnancy test for a participant who reports a new pregnancy and/or as clinically indicated.

- If a participant is confirmed to be pregnant and plans to continue the pregnancy, the following will be performed: (1) Fetal ultrasound to confirm gestational age and developmental status of the pregnancy; (2) Syphilis test result and history of treatment will be collected from medical records if previously done and documented; otherwise, the participant will be referred for testing and potential treatment per local standard of care and the results will be collected from the subsequent medical records. Participants will also receive HIV testing and counseling as clinically indicated.

- Any participant with a new HIV diagnosis will be offered counseling, as clinically indicated. They will also be referred for care and treatment according to local guidelines. Viral load and CD4+ T cell count must be obtained on study.

- In case of a change in antiretroviral (ARV) medication (for PLWH participants), blood will be collected for viral load and CD4+ T cell count approximately 4 to 6 weeks since the change in medication.

Following completion of all procedures in the preceding list, and if results indicate that vaccination may proceed, vaccination dose is prepared and administered (see Section 8.4, Appendix H and Appendix I).

Immediately following vaccination, the participant remains in the clinic for observation for 15 minutes or more in accordance with current applicable guidance (eg, local guidance). After the observation period, participants will have a postvaccination assessment prior to clinic discharge, including assessment of:

- The injection site, (see Section 9.8.2 and
- Systemic symptoms (see Section 9.8.1).

See CoVPN 3008 (Ubuntu) SSP for details.

9.4 Follow-up procedures

The following procedures are performed during follow-up per the schedules in Appendix H and Appendix I:

- At all follow-up visits: symptom-directed physical examination if needed
- At the last clinic visit: abbreviated physical examination
- Assessment of targeted concomitant medications
- Blood collection and nasal swabs (as described in Appendix B, Appendix C, Appendix D, Appendix E, Appendix F and Appendix G)
- Participants will be instructed to contact the clinic if they have any AEs to report. The clinic will contact the participant every 2 weeks, as described in Section 9.5.
- Urine pregnancy test for a participant who reports a new pregnancy and/or as clinically indicated
- If a participant is confirmed to be pregnant and plans to continue the pregnancy, the following will be performed: (1) Fetal ultrasound to confirm gestational age and developmental status of the pregnancy; (2) Syphilis test result and history of treatment will be collected from medical records if previously done and documented; otherwise, the participant will be referred for testing and potential treatment per local standard of care and the results will be collected from the subsequent medical records. Participants will also receive HIV testing and counseling as clinically indicated.
- Any participant with a new HIV diagnosis will be offered counseling, as clinically indicated. They will also be referred for care and treatment according to local guidelines. Viral load and CD4+ T-cell count must be obtained on study.

- In case of a change in ARV medication (for PLWH participants), blood will be collected for viral load and CD4+ T-cell count approximately 4 to 6 weeks after the change in medication. For participants being exited less than 4 weeks after a change in ART, the site may choose to follow up these individuals after exit.

- All pregnancies should be monitored to record the outcome of the pregnancy (eg viability, congenital malformations at birth) (see Section 9.12 and CoVPN 3008 (Ubuntu) SSP for details).

9.5 Active COVID-19 symptomatic surveillance

Participants will use Memory Aid 1 for surveillance of symptoms and be instructed to contact the clinic if they have COVID-19 like symptoms. Additionally, the site will make arrangements to contact the participant to elicit symptoms of COVID-19 as described below. These symptoms will be used to establish whether a participant meets criteria for COVID-19 disease as defined by Primary Endpoint 1. If a participant has symptoms consistent with COVID-19, they will be instructed to contact the clinic to review their symptoms and if needed to schedule a COVID-19 Symptom visit. Results of testing will be reported through the CRF and participants who have a positive SARS-CoV-2 NAAT test result will follow procedures described in Section 9.6 and Appendix J.

The below prespecified symptoms that meet the criteria for suspicion of COVID-19 will be elicited approximately every 2 weeks from the participant and the presence of any one of these symptoms lasting at least 48 hours (except for fever and/or respiratory symptoms) will result in the site arranging a Symptom Visit to collect a nasal swab within 72 hours. (If the site becomes aware of the symptoms >72 hours after they have resolved, nasal swab/symptom visit is not required.)

- Fever (temperature ≥ 38ºC) or chills (of any duration, including ≤ 48 hours)

- Shortness of breath or difficulty breathing (of any duration, including ≤ 48 hours)

- Cough (of any duration, including ≤ 48 hours)

- Fatigue

- Muscle or body aches

- Headache

- New loss of taste or smell (of any duration, including ≤ 48 hours)

- Sore throat

- Congestion or runny nose
- Nausea or vomiting
- Diarrhea

It is important to note that some of the symptoms of COVID-19 overlap with systemic adverse reactions that are expected after vaccination with mRNA-1273 and mRNA-1273.222 vaccines (eg, myalgia, headache, fever, chills). During the first 7 days after vaccination, when these adverse reactions are common, investigators should use their clinical judgement to decide if a COVID-19 symptom visit should be scheduled. Any study participant reporting respiratory symptoms during the 7-day period after vaccination should be evaluated for COVID-19.

9.6 COVID-19 Symptom Visits and COVID-19 Post-diagnosis Schedule

The following procedures will be performed at the COVID-19 Symptom Visits for participants meeting the criteria for COVID-19 Symptom Visits outlined in Section 9.5. Participants with COVID-19 confirmed by SARS-CoV-2 NAAT test, regardless of symptoms, enter a postdiagnosis schedule (see Appendix J) to assess the presence, types, severity, and duration of potential COVID-related symptoms as well as duration of viral shedding. Participants found to have a positive SARS-CoV-2 NAAT test as part of a regularly scheduled visit outlined in Section 9.3 will also enter the postdiagnosis schedule (see Appendix J).

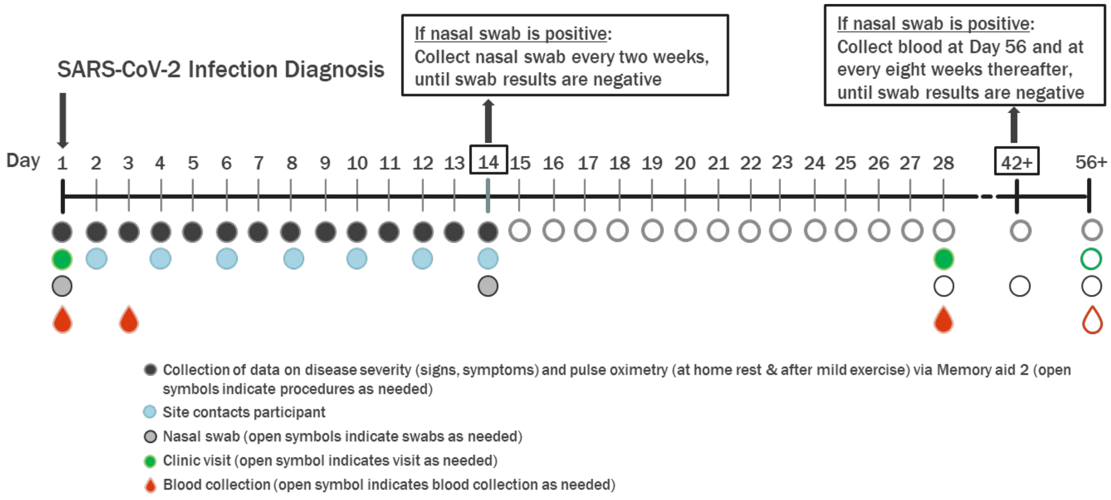

Figure 9-1 Overview of clinical procedures post–SARS-CoV-2 infection diagnosis.

Day 1 (see Appendix J):

- Participant history (participants will be queried regarding COVID-19–like symptoms and any concomitant medications taken for these symptoms)
- Symptom-directed physical examination (to include temperature and O2 saturation)
- Nasal swabs collection per schedule of procedures (see Laboratory Procedures Table for COVID-19 Symptom Visits in Appendix J)
- Blood collection per schedule of procedures (see Laboratory Procedures Table for COVID-19 Symptom Visits in Appendix J)
- Participant will be provided with Memory Aid 2 – for collection of daily symptoms of COVID-19 and recollection of whether clinical care was sought or hospitalization occurred (via self-report and/or request for medical records)
- Participant will be instructed to complete the Memory Aid 2 for a period of 14 days or until a negative SARS-CoV-2 NAAT test is obtained., whichever is longer
- The study sites will provide a portable pulse oximeter for recording O2 saturation (at rest and following mild exercise) daily in the Memory Aid 2 and train the participant on the use of the pulse oximeter under both conditions (rest and mild exercise). The device will be accompanied by careful instructions for use and recording of O2 saturation in the Memory Aid 2 and who to contact for assistance if the device malfunctions.
- The purpose of the monitoring of O2 saturation is to enhance the assessment of severity and progression of COVID-19, as well as to ensure that participants are adequately informed with respect to their medical condition and seek medical care per instructions.
- If results of SARS-CoV-2 NAAT testing indicate infection, the participant is considered to have COVID-19 and will follow the postdiagnosis schedule (see Appendix J) and procedures below. Retesting is permitted if the participant continues to have symptoms and no other diagnosis is apparent.
- If results of SARS-CoV-2 NAAT testing do not indicate infection, the participant will stop recording symptoms on Memory Aid 2 and will return to the regular study schedule.

Hold vaccination until results of SARS-CoV-2 NAAT testing have been received.

Postdiagnosis Schedule for participants with COVID-19

All participants with COVID-19 will receive the optimal local standard of care for prevention and treatment of COVID-19, including, for example, access to

temperature monitoring, pulse oximetry, oxygen, medications such as steroids, and hospitalization at facilities that are both prepared and experienced in the management of severe cases to minimize mortality.

If a participant is diagnosed with COVID-19 between Study Day 1 and Day 28 (ie, first and second study vaccinations), country-specific guidelines on administration of vaccination if available will be followed. If no country-specific guidelines exist, vaccination will be held until participant has recovered adequately to proceed with vaccination in the judgement of the clinician (see Section 9.9).

Postdiagnosis Day 2 – 14:

- Participants will continue to use Memory Aid 2 to record daily symptoms of COVID-19 (if any), and whether clinical care was sought or hospitalization occurred, through Day 14 or until a negative SARS-CoV-2 NAAT test, whichever is longer.
- Participant will measure and record in the Memory Aid 2 their O2 saturation (at rest and following mild exercise, defined as walking around the room for 1 minute) using a pulse oximeter through Day 14 or until a negative SARS-CoV-2 NAAT test, whichever is longer.
- Participant will be contacted by phone (or alternative contact method) by CRS staff every other day, at a minimum, through Day 14 or until symptoms improve (if symptomatic), whichever is longer, and will be instructed to contact the clinic if their symptoms are getting worse.
- Acute illness blood collection (Day 3) per schedule of procedures (see Laboratory Procedures Table for COVID-19 Symptom Visits in Appendix J).
- Participants will be instructed to self-isolate per local guidelines.
- Nasal swabs collection per schedule of procedures (see Laboratory Procedures Table for COVID-19 Symptom Visits in Appendix J).
- Symptoms recorded in the Memory Aid 2 will be entered into the study record if not previously entered.

Postdiagnosis Day 28:

- Nasal swabs collection, if indicated, per schedule of procedures (see Laboratory Procedures Table for COVID-19 Symptom Visits in Appendix J).
- The participant’s clinical course is reviewed in detail.
- Convalescent illness blood collection per schedule of procedures (see Laboratory Procedures Table for COVID-19 Symptom Visits in Appendix J).

- Following resolution of the self-isolation period, participants return to the main study schedule to complete remaining procedures. Follow-up duration for participants diagnosed with severe COVID-19 disease may be adjusted in consultation with the CRS (eg, to avoid interference with participant initiation of SARS-CoV-2 treatment).

Postdiagnosis Day 42:

- If participant had positive SARS-CoV-2 NAAT test on Day 14, nasal swab collection approximately every 2 weeks will continue until a negative test result is obtained. See schedule of procedures for COVID-19 Symptom Visits in Appendix J.

Postdiagnosis Day 56+:

- If participant had positive SARS-CoV-2 NAAT test on Day 42, blood will be collected on Day 56 and approximately every 8 weeks thereafter, along with nasal swab collection approximately every 2 weeks, until a negative SARS-CoV-2 NAAT test result is obtained. See schedule of procedures for COVID-19 Symptom Visits in Appendix J.

9.7 Visit windows and missed visits

Visit windows are included in Appendix K. Guidance for out of window visits is provided in the CoVPN 3008 (Ubuntu) SSP.

9.8 Assessments of reactogenicity for a subset of participants (Safety Subset)

To confirm safety in the local population, reactogenicity events (solicited adverse events) may be collected for a subset of participants at selected sites in each country participating in this trial. (See CoVPN 3008 (Ubuntu) SSP for details).

Reactogenicity assessments are performed after each vaccination. All reactogenicity symptoms are followed until resolution and graded according to the Division of AIDS Table for Grading the Severity of Adult and Pediatric Adverse Events, Corrected Version 2.1, dated July 2017 (see Section 11.2.2).

The reactogenicity assessment period is up to 7 days following vaccination. The maximum severity reached for each symptom during the assessment period is reported on CRFs. Participants will be given a postvaccination memory tool to assist with recall of symptoms. The site staff and the participant will make multiple efforts in good faith to be in contact after the 7-day reactogenicity period, or sooner if indicated. At that time, site staff will discuss reactogenicity events with the participant and will record the relevant information on either the appropriate

CRF(s) or participant chart note. Clinic staff will follow new or unresolved reactogenicity symptoms present at day 7 to resolution. Participants are instructed to contact the clinic for events that arise during the period between vaccination and the next scheduled visit. In general, a participant who self-reports any postvaccination reaction greater than moderate will be advised to contact the clinician within 48 hours after onset, unless the reaction is improving and/or has resolved completely.

Reactogenicity assessments include assessments of systemic and local symptoms and vaccine-related lesions. Events meeting SAE/AEs requiring expedited reporting criteria, are recorded on an AE log form.

9.8.1 Assessment of systemic and local symptoms

Systemic symptoms include increased body temperature, malaise and/or fatigue, generalized myalgia, headache, chills, arthralgia (in several joints), and nausea. Local symptoms include pain and/or tenderness, lymphadenopathy at and/or near the injection site.

Body temperature is measured by oral or infrared thermometry and reported in degrees Celsius. A measurement may be taken if participant is feeling feverish. Body temperature measurement is not required during the in-clinic reactogenicity assessment immediately following vaccination.

9.8.2 Assessment of injection site(s)

Typical injection site reactions are erythema/redness and induration/swelling. The maximum diameter for all injection site reactions is recorded.

All injection site reactions are monitored until resolution. Areas with any diameter greater than 5 cm are followed daily; otherwise, the frequency of follow-up is based on clinician judgment.

9.9 Delaying vaccinations for a participant

Vaccinations will be delayed if the participant is clinically ill, until participant has recovered adequately to proceed with vaccination, in the judgement of the clinician.

A participant's scheduled vaccination will also be delayed if participant is:

Within 90 days prior to any study injection

- Receipt of blood products or immunoglobulin

Within 30 days prior to any study injection

- Receipt of live attenuated vaccines
- Receipt of allergy treatment with antigen injections

Within 14 days prior to any study injection

- Receipt of any vaccines that are not live attenuated vaccines (eg, pneumococcal)

Within 2 to 4 weeks

- Initiating/changing antiretroviral therapy. A delay in the vaccination schedule is not required if a participant only has changes in dosage as opposed to changes in the type of antiretroviral medications.

Participants who plan to receive licensed vaccines or allergy treatments should be counseled to schedule receipt of these substances, when possible, outside the intervals indicated above. Therefore, if circumstances allow, these substances should also be avoided in the interval between a study vaccination and completion of the 2 or 3-week postvaccination follow-up visit.

9.10 Discontinuing vaccination for a participant who receives a COVID-19 vaccine outside the study

In the event a participant receives another available COVID-19 vaccine outside the study, an individual participant’s study vaccinations will be permanently discontinued. Participants should be counseled on the importance of continuing with the study and strongly encouraged to participate in follow-up visits and protocol-related procedures per the protocol for the remainder of the trial, unless medically contraindicated.

9.11 Early termination visit

In the event of early participant termination, site staff should consider if final specimen collection is appropriate and follow operational guidance from the CoVPN protocol team.

9.12 Pregnancy

Pregnancy will be assessed by self-report or confirmed by pregnancy test at screening or enrollment visit. Self-reported pregnancy will be evaluated by urine pregnancy test. If a participant is confirmed to be pregnant and plans to continue the pregnancy, the following will be performed: (1) Fetal ultrasound to confirm gestational age and developmental status of the pregnancy; (2) Syphilis test result and history of treatment will be collected from medical records if previously done

and documented; otherwise, the participant will be referred for testing and potential treatment per local standard of care and the results will be collected from the subsequent medical records. In addition, HIV testing and counseling will be performed if not already indicated for that visit.

If the participant terminates from the study prior to the pregnancy outcome, the site should make efforts to remain in contact with pregnant participants to ascertain and document the pregnancy outcome (maternal and fetal/neonatal) with completion of the relevant CRFs even after exiting the study, per protocol. All pregnancies and pregnancy outcomes should be recorded and reported (see the CoVPN 3008 (Ubuntu) SSP).

10 Laboratory

10.1 CRS laboratory procedures

The CoVPN 3008 (Ubuntu) SSP and other study materials provide further guidelines for operational issues concerning the clinics and laboratories. These documents include special considerations for phlebotomy and guidelines for general specimen collection, specimen labeling and specimen processing.

With the exception of SARS-CoV-2 PCR, all assays described below are performed as research assays to evaluate the ability of the SARS-CoV-2 virus to induce immune responses in the context of the participants' genetic background and are not approved for use in medical care. Results from these research assays are not made available to participants or medical professionals to guide treatment decisions.

10.2 Total blood volume

Required blood collections per visit are shown in Appendix B, Appendix C, Appendix D, Appendix E, Appendix F, Appendix G, Appendix H, Appendix I, and Appendix J. The total blood volume drawn for each participant will not exceed 500 mL in any 56-day (8-week) period. Blood collection for PBMCs for pregnant women will be limited to those who are documented to have COVID-19 unless they are severely anemic. (See CoVPN 3008 (Ubuntu) SSP for details.)

10.3 Endpoint assays

10.3.1 SARS-CoV-2 PCR

Qualitative and/or quantitative, real-time reverse transcription polymerase chain reaction (RT-PCR) assays to detect SARS-CoV-2 will be run on nasal samples collected from study participants.

10.3.2 Anti-SARS-CoV-2 antibodies

Chemiluminescent microparticle immunoassays (CMIA) to detect SARS-CoV-2 antibodies will be run on serum samples collected from study participants.

10.3.3 SARS-CoV-2 viral sequencing

Genome sequencing of SARS-CoV-2 virus may be performed on nasal samples from study participants using a metagenomic approach. RNA from positive specimens is converted to a cDNA library by reverse transcription and the library

is sequenced. Resulting consensus sequences are assembled against a SARS-CoV-2 reference genome.

10.3.4 Neutralizing antibody assay (nAb)

SARS-CoV-2–specific nAb assays may be performed on serum samples from study participants. The assay will test neutralization of S-pseudotyped and/or full-genome recombinant viruses as measured by a reduction in luciferase (Luc) reported gene expression after infection in ACE-2 positive cells.

10.3.5 Binding antibody multiplex assay (BAMA)

SARS-CoV-2 specific total IgG and IgM binding antibodies may be assessed in serum samples. In addition, SARS-CoV-2–specific serum IgA and IgG subclass (IgG1, IgG2, IgG3, and IgG4) antibodies may also be assessed.

10.3.6 T-cell assays

Flow cytometry may be used to examine SARS-CoV-2-specific CD4+ and CD8+ T-cell responses following stimulation of PBMCs with synthetic peptides that span the proteins encoded by the SARS-CoV-2 virus. Data will be reported as percentages of CD4+ or CD8+ T cells responding to a specific peptide pool. Additional cell surface markers, cytokines, or functional markers may also be analyzed. Additional T-cell assays may include evaluation of additional cytokines using a multiplexed secreted cytokine assay, mapping of individual peptide-level T-cell responses, and characterization of viral inhibition.

10.3.7 SARS-CoV-2 -specific B cell phenotyping and monoclonal antibody isolation

SARS-CoV-2 -specific memory B cells and plasmablasts induced by infection may be identified and characterized using fluorescently labeled recombinant proteins in combination with a flow cytometry phenotyping panel. In particular, SARS-CoV-2 spike and receptor binding domain-specific B cells and plasmablasts will be enumerated and may be further characterized for expression of memory, activation, inhibitory or other markers of interest. From a subset of individuals, SARS-CoV-2-specific memory B cells or plasmablasts will be isolated for B-cell receptor sequencing and generation of recombinant monoclonal antibodies for in-depth characterization (eg, function, binding-specificity).

10.3.8 Gene expression

RNA may be isolated from PBMC or whole blood and used for gene expression analysis by RNA sequencing, real-time PCR, single-cell proteogenomic profiling or other methods. Gene expression profiles will be correlated with other immune responses and clinical outcomes.

10.4 Lab assay portfolio

Additional assays may be performed per the Laboratory Center assay portfolio, which includes immune assessments such as those for cellular, humoral, and innate immune responses, and host genetics. The assay portfolio will be updated periodically to include new assays and adjust qualification levels of existing assays.

10.5 Exploratory studies

Samples may be used for other testing and research related to furthering the understanding of SARS-CoV-2 immunology, virology, or vaccines. In addition, cryopreserved samples may be used to perform additional assays to support standardization and validation of existing or newly developed methods.

10.6 Specimen storage and other use of specimens

The CoVPN stores specimens from all study participants indefinitely, unless a participant requests that specimens be destroyed or if required by IRB/EC or RE.

Other use of specimens is defined as studies not covered by the protocol or the informed consent form for the study (see Section 10.5 and Appendix A).

This research may relate to SARS-CoV-2, vaccines, coronaviruses, the immune system, and other diseases. This could include genetic testing and, potentially, genome-wide studies. This research is done only to the extent authorized in each study site's informed consent form, or as otherwise authorized under applicable law. Other research on specimens ("other use") will occur only after review and approval by the CoVPN, the IRB/EC of the researcher requesting the specimens, and the IRBs/ECs/REs of the CRSs if required.

As part of on-going consenting for the study, participants document their initial decision to allow or not allow their specimens to be used in other research, and they may change their decision at any time. The participant's initial decision about other use of their specimens, and any later change to that decision, is recorded by their CRS in a Web-based tool that documents their current decisions for other use of their specimens. The CoVPN will only allow other research to be done on specimens from participants who allow such use.

CRSs must notify CoVPN Regulatory Affairs if institutional or local governmental requirements pose a conflict with or impose restrictions on specimen storage or other use of specimens.

10.7 Biohazard containment

The transmission of blood-borne pathogens can occur through contact with contaminated needles, blood, and blood products, and the transmission of SARS-CoV-2 and other respiratory pathogens may occur through contact with contaminated respiratory droplets, aerosols, and other biological materials. Appropriate precautions will be employed by all personnel in the collection, shipping, and handling of all specimens for this study, as currently recommended by local health authorities, the CDC, the NIH, or other applicable agencies.

All dangerous goods materials, including Biological Substances, Category A or Category B, must be transported according to instructions detailed in the International Air Transport Association Dangerous Goods Regulations.

11 Safety monitoring and safety review

11.1 Safety monitoring and oversight

11.1.1 CoVPN 3008 (Ubuntu) PSRT

The CoVPN 3008 (Ubuntu) PSRT is composed of the following members:

- DAIDS Medical Officer
- Protocol co-chairs
- In Country Lead Investigators
- Protocol Team leaders
- Clinical Research Physician
- Core Medical Monitors
- Regional Medical Liaisons
- Clinical Safety Specialists

The clinician members of CoVPN 3008 (Ubuntu) PSRT are responsible for decisions related to participant safety. A quorum and further details for the PSRT will be defined in the PSRT Charter.

The Protocol Team clinical data manager, clinical trial manager, and others may also be included in CoVPN 3008 (Ubuntu) PSRT meetings.

11.1.2 NIAID Data Safety Monitoring Board (DSMB)

The NIAID HIV DSMB, for as long as it is involved in the trial, and the OG are responsible for safeguarding the interests of clinical trial participants and for enhancing the integrity of the trial. The NIAID HIV DSMB assesses the effects of the study vaccines during the trial and may give advice to the CoVPN 3008 (Ubuntu) OG.

More details on the role of and the data provided to the DSMB will be described in a DSMB charter and SAP.

11.1.3 SDMC or designee roles and responsibilities in safety monitoring

The roles and responsibilities of the SDMC in relation to safety monitoring include:

- Maintaining a central database management system for CoVPN 3008 clinical data;
- Providing reports of clinical data to appropriate groups such as the CoVPN 3008 (Ubuntu) PSRT, DSMB, and to support safety reports for submission to national regulatory authorities as needed (see Section 11.1.1, 11.1.2 and 11.2.5).

11.1.4 CoVPN LOC or designee roles and responsibilities in safety monitoring

- Daily monitoring of clinical data for events that meet the CoVPN 3008 (Ubuntu) PSRT AE review criteria (see Section 11.3.1);
- Notifying CoVPN CRSs and other groups when safety pauses are instituted and lifted (see Section 11.3.1);
- Querying CoVPN CRSs for additional information regarding reported clinical data; and
- Providing support to the CoVPN 3008 (Ubuntu) PSRT.

11.2 Safety reporting

11.2.1 Submission of safety forms to SDMC

Site staff must submit all safety forms (eg, reactogenicity, adverse event) within 3 business days of the site being notified, or sooner per Section 11.3.1 below. The forms should not be held in anticipation of additional information at a later date. If additional information is received at a later date, the forms should be updated and resubmitted before the end of the next business day after receiving the new information. For the case of a longer site holiday closure, site staff must submit the data by the end of the 5th day (local time) after receiving the information even if this day is a holiday.

For example: If the site becomes aware of an AE on Thursday (Day 1), the site must submit the data by the end of the next business day, on Friday. If there is a longer site holiday closure, then this AE must be reported no later than the end of the fifth day, Monday (Day 5). If Monday is a holiday as well, all safety forms still need to be submitted by the end of Monday (Day 5).

11.2.2 AE reporting

Sites are expected to notify CoVPN clinical safety staff or designee of any serious safety concern requiring their attention (see Section 11.3.1). Telephone numbers and email addresses are found on the CoVPN 3008 (Ubuntu) protocol home page

on the CoVPN Members' site (https://fredhutch.sharepoint.com/sites/CoVPN/covpn3008/SitePages/Home.aspx).

In the case of email notification, clinical safety staff or designee will reply within 1 business day. If email service is not available, the CRS should notify clinical safety staff of the event by telephone, and then submit CRFs.

In addition, site investigators are required to submit AE information in accordance with IRB/EC and any applicable RE requirements.

An AE is any untoward medical occurrence in a clinical investigation participant administered a study product/procedure(s) and which does not necessarily have a causal relationship with this treatment. An AE can therefore be any unfavorable and unintended sign (including an abnormal laboratory finding), symptom, or disease temporally associated with the use of an investigational study product/procedure(s), whether or not related to the investigational study product/procedure(s). All AEs are graded according to the Division of AIDS (DAIDS) Table for Grading the Severity of Adult and Pediatric Adverse Events, Corrected Version 2.1, July 2017, available on the RSC website at https://rsc.niaid.nih.gov/clinical-research-sites/daids-adverse-event-grading-tables, <u>except</u>:

Injection Site Erythema or Redness and Injection Site Induration or Swelling activities such that:

- Grade 1 is: 2.5 to < 5 cm in diameter;
- Grade 2 is: ≥ 5 to < 10 cm in diameter;
- Grade 3 is: ≥ 10 cm in diameter OR Ulceration OR Secondary infection OR Phlebitis OR Sterile abscess OR Drainage;
- Grade 4 is: Potentially life-threatening consequences (eg, abscess, exfoliative dermatitis, necrosis involving dermis or deeper tissue);

Clinic staff should evaluate every AE to determine if (1) if the AE meets the criteria for prompt PSRT AE review (see Section 11.3.1), (2) the AE meets the requirements for expedited reporting (see Section 11.2.4), and (3) if the AE is a potential immune-mediated disease that may be listed as an AE of special interest (AESI) (see Appendix M).

For a subset of participants, the following data will be collected and reported:

- Unsolicited AEs for 28 days following each vaccination
- Solicited AEs following each vaccination (see Section 9.8)

For all participants, the following AEs will be collected and reported throughout the entire study:

- SAEs/EAEs,
- AESIs (a list is provided in Appendix M).

The AEs collected in this study are reported to the SDMC on the appropriate CRF.

See CoVPN 3008 (Ubuntu) Study-specific Procedures for detail on AE reporting.

11.2.3 Serious adverse event

The term "Serious Adverse Event" (SAE) is defined as follows: An adverse event or suspected adverse reaction is considered serious if, in the view of either the investigator or the Sponsor, it results in any of the following outcomes:

- death,
- a life-threatening adverse event,
- inpatient hospitalization or prolongation of existing hospitalization,
- a persistent or significant incapacity or substantial disruption of the ability to conduct normal life functions,
- congenital anomaly/birth defect.
- Is Medically Important*

* Important medical events that may not result in death, be life-threatening, or require hospitalization may be considered serious when, based upon appropriate medical judgment, they may jeopardize the patient or subject and may require medical or surgical intervention to prevent one of the outcomes listed in this definition.

"Life-threatening" refers to an adverse event that, at occurrence, represents an immediate risk of death to the subject. Similarly, a hospital admission for an elective procedure is not considered an SAE.

11.2.4 Expedited reporting of adverse events

CRS staff must report all related SAEs within 3 business days of their knowledge of the event to the clinical database, so that applicable safety data can be reviewed, see CoVPN 3008 (Ubuntu) SSP for details.

The study products for which expedited reporting are required are:

- mRNA-1273 vaccine
- mRNA-1273.222 vaccine

11.2.5 Reporting of AEs to pertinent national regulatory authorities

Reports are prepared and filed to appropriate regulatory authorities within the timelines required by pertinent national regulatory authorities.

For details refer to the CoVPN 3008 Safety Data Process document.

11.3 Safety reviews

11.3.1 Prompt PSRT AE review

If the trial is placed on safety pause, all enrollments and vaccinations with the product related to the event that triggered the pause will be held until further notice.

Vaccinations may be suspended for safety concerns, if, in the judgment of the CoVPN 3008 (Ubuntu) PSRT, participant safety may be threatened. Criteria for an individual participant's departure from the schedule of vaccinations are listed in Section 9.9.

The following will result in a prompt PSRT AE review to consider a safety pause:

- Any SAE determined to be related to study product
- Any Grade 4 objective local reactogenicity sign or systemic reactogenicity sign or symptom

These require notification by CRS staff within 24 hours of becoming aware of the event to the clinical safety staff for a prompt PSRT review; see CoVPN 3008 (Ubuntu) SSP for details.

If a prompt PSRT AE review is triggered, CoVPN safety staff or designee notifies the PSRT as soon as possible during working hours (local time) or, if the information was received during off hours, by the morning of the next workday.

Once the trial is paused, the CoVPN clinical safety team notifies the CoVPN 3008 (Ubuntu) PSRT, SAMRC Regulatory Affairs, CoVPN Regulatory Affairs, participating CoVPN CRSs and the OG.

If the trial is paused, the CoVPN 3008 (Ubuntu) PSRT reviews safety data and decides whether the pause can be lifted or permanent discontinuation of vaccination is appropriate, consulting the OG if necessary. CoVPN Core notifies

the participating CoVPN CRSs, SAMRC Regulatory Affairs, and CoVPN Regulatory Affairs of the decision regarding resumption or discontinuation of study vaccinations.

The CoVPN requires that each CRS submit to its IRB/EC and any applicable RE protocol-related safety information (such as unanticipated problems involving risks to participants or others). CRSs must also follow all applicable RE reporting requirements.

Based on the CoVPN 3008 (Ubuntu) PSRT assessment, SAMRC notifies SAHPRA. Other pertinent national and regional authorities will also be notified as appropriate (see Section 11.2.5).

In addition, all other reportable AEs are reviewed routinely by the CoVPN 3008 (Ubuntu) PSRT (see Sections 11.3.2 and 11.3.3).

11.3.2 Review of cumulative safety data

Routine safety review occurs at the start of enrollment and then throughout the study.

Reviews proceed from a standardized set of protocol-specific safety data reports. These reports are produced by the SDMC or designee and include queries to the CoVPN CRSs. Events are tracked by internal reports until resolution.

11.3.3 Weekly review

The CoVPN 3008 (Ubuntu) PSRT reviews clinical safety reports weekly and conducts calls to review the data as appropriate, until 1 month post the initial set of injections (second injection) visits have been completed (Visit 7) are completed. Thereafter, less frequent reporting and safety reviews may be conducted at the discretion of the CoVPN 3008 (Ubuntu) PSRT. CoVPN LOC or designee reviews reports of clinical AEs. Events identified during the review that are considered questionable, inconsistent, or unexplained are referred to the CoVPN CRS clinic staff for verification.

12 Protocol conduct

This protocol and all actions and activities connected with it will be conducted in compliance with the principles of GCP (ICH E6 (R2)), and according to NIAID and CoVPN policies and procedures as specified in the CoVPN 3008 SSP, NIAID Clinical Research Policies and Standard Procedures Documents including procedures for the following:

- Protocol registration, activation, and implementation;
- Informed consent, screening, and enrollment;
- Study participant reimbursement;
- Clinical and safety assessments;
- Safety monitoring and reporting;
- Data collection, documentation, transfer, and storage;
- Participant confidentiality;
- Study follow-up and close-out;
- Unblinding of staff and participants;
- Quality control;
- Protocol monitoring and compliance;
- Specimen collection, processing, and analysis;
- Exploratory and ancillary studies and substudies, and
- Destruction of specimens.

Any policies or procedures that vary from CoVPN and/or NIAID standards or require additional instructions (eg, instructions for randomization specific to this study) will be described in the CoVPN 3008 (Ubuntu) SSP.

12.1 Study termination

NIAID and the Study Sponsor reserve the right to terminate or curtail a clinical study for any reason, including but not limited to the following:

- risk to subject safety
- the scientific question is no longer relevant or the objectives will not be met (ie, slow accrual)
- failure to comply with GCP, US. Federal regulations, or Terms and Conditions of Award
- occurrence of unforeseen drug safety issues or data from preclinical studies indicate a presence of unanticipated toxicity
- risks that cannot be adequately quantified
- ethical concerns raised by the local community or local medical care/health care authorities
- failure to remedy deficiencies identified through site monitoring
- substandard data
- reaching a major study endpoint substantially before schedule with persuasive statistical significance.

This study may also be terminated early by the determination of the FDA, the United States Department of Health and Human Services Office for Human Research Protections (OHRP). In addition, the conduct of this study at an individual CRS may be terminated by the determination of the IRB/EC and any applicable RE.

12.2 Emergency communication with study participants

As in all clinical research, this study may generate a need to reach participants quickly to avoid imminent harm, or to report study findings that may otherwise concern their health or welfare and their willingness to remain on study.

When such communication is needed, the CRS will request that its IRB/EC and any applicable RE expedite review of the message. If this review cannot be completed in a timeframe consistent with the urgency of the required communication, the site can contact the participant without IRB/EC approval if such communication is necessary to avoid imminent harm to the study participant. The CRS must notify the IRB/EC and any applicable RE of the matter as soon as possible.

12.3 Site monitoring

Site monitoring is conducted by Hutchinson Centre Research Institute of South Africa (HCRISA) to ensure that the rights and well-being of trial participants are protected, that the reported trial data are accurate, complete, and verifiable, and that the conduct of the trial is in compliance with the currently approved protocol/amendment(s), Declaration of Helsinki, Guidelines for Good Practice in the Conduct of Clinical Trials in Human Participants, ICH, FDA regulations and with applicable regulatory requirement(s). Refer to CoVPN 3008 (Ubuntu) Clinical Trial Monitoring Plan for more information about monitoring schedule, reports, and virtual and onsite support.

13 Version history

The Protocol Team may modify the original version of the protocol. Modifications are made to CoVPN protocols via clarification memos, letters of amendment, or full protocol amendments.

The version history of, and modifications to, Protocol CoVPN 3008 (Ubuntu) are described below.

Protocol history and modifications

Date: February 27, 2023

Protocol version:7.0
Protocol modification: Full protocol amendment 6

Item 1 Revised in Section 1, *Overview*; Section 2, *Background*; Section ; Section 3, *Objectives and Endpoints*; Section 6, S*tatistical considerations*; Section 9, *Clinical procedures*; Appendix B, *Laboratory procedures table for Groups 1 and 3 (SARS-CoV-2 seronegative participants, main study)*, Appendix C, *Laboratory procedures table for Groups 2 and 4 (SARS-CoV-2 seropositive participants, main study)*, Appendix D, *Laboratory procedures for PBMC immunogenicity subset, Groups 1 and 3*; Appendix E, *Laboratory procedures for PBMC immunogenicity subset, Groups 2 and 4*; Appendix F, *Laboratory procedures for the PBMC immunogenicity correlates (Group 1, HIV positive, SARS-CoV-2 seronegative participants)*; Appendix G, *Laboratory procedures for the PBMC immunogenicity correlates (Group 2, HIV positive, SARS-CoV-2 seropositive participants)*; Appendix H, *Schedule of clinic procedures for Groups 1 and 3 (SARS-CoV-2 seronegative participants)*; Appendix I, *Schedule of clinic procedures for Groups 2 and 4 (SARS-CoV-2 seropositive participants)*, Appendix J, *COVID-19 symptom visits*; Appendix K, *Visit windows* and Appendix M, *Addendum 2 to the sample informed consent form (SICF)*: harmonizing protocol language with the changed study focus

Item 2 Revised in in Section 1, *Overview*; Section 3, *Objectives and endpoints*; Section 6.4.3, *Analysis of relative risk between treatment arms*: definitions to be considered for COVID-19

Item 3 Revised in Section 2, *Background*; Section 3, *Objectives and endpoints* and Section 6, *Statistical considerations*: roles of the DSMB, OG and the EAC; and Section 11, Safety monitoring and safety review

Item 4 Clarified in Section 9.4, *Follow-up procedures*: change in antiretroviral medication

Item 5 Clarified in Section 9.11, *Early termination visit*: operational guidance for early study termination

Item 6 Clarified in Section 9.12, *Pregnancy*: documentation of pregnancy outcome

Item 7 Updated in Section 11.2, 2, *AE reporting*: weblink for the CoVPN Member's site

Item 8 Clarified in Section 11.2.5, *Reporting of AEs to pertinent national regulatory authorities* and Section 12, *Protocol conduct*: reference documents mentioned in these sections refer to CoVPN3008 reference documents

Item 9 Updated in Title page; Section 13, *Version history* and Section 16, *Literature cited*: Contents of this amendment

Item 10 Corrected throughout the Protocol: Minor errors in grammar, typography, formatting

Item 11 Updated throughout the Protocol: Section numbering and cross-references

Item 12 Updated per Protocol Version 6.0, Clarification Memo 1, dated October 05, 2022

Date: October 05, 2022

Protocol version: Version 6.0
Protocol modification: Clarification Memo 1

Item 1 Revised in Section 2.1.1, *Overall study design* and Section 8.1, *Vaccine regimen*: language related to injection volumes of the study vaccines

Item 2 Corrected in Full protocol amendment 5 document: version number

Date: September 27, 2022

Protocol version:6.0
Protocol modification: Full protocol amendment 5

Item 1 Revised in Section 1, *Overview*; Section 2, *Background*; Section 3, *Objectives and E*ndpoints; Section 6, Statistical considerations; Section 8, *Study Products*; Section 9, *Clinical procedures*; Section 14, *Document references (other than literature citations)*; Appendix K, *Visit windows;* Appendix L, *Addendum to the sample informed consent form (SICF)*: updated information/language related to the addition of the mRNA-1273.222 vaccine at Month 6 and the current schema.

Item 2 Clarified in Section 6.2, *Definition of study cohorts for analysis*: group assignment based on SARS-CoV-2 sero-status

Item 3 Added in Section 6.3.1, *Power calculations for coprimary objectives 1-4 (Efficacy)*: SARS-CoV-2 (re-infection) incidence rate among unvaccinated PLWH SARS-CoV-2 positive individua1.

Item 4 Clarified in Section 6.4, *Statistical analyses*: analysis of coprimary efficacy endpoints

Item 5 Clarified in Section 8.4, *Administration of study products*: administration of intramuscular injections

Item 6	Added in Section 9.1.1, *Protocol-specific consent form*: addendum to informed consent form
Item 7	Updated in Appendix J, *COVID-19 Symptom Visits*: Laboratory Procedures Table: added footnote indicators for clarity and updated footnote #5 to correct an error
Item 8	Updated Section 1.1, *Protocol Team*: to reflect current team composition...
Item 9	Updated in Title page; Section 13, *Version history* and Section 16, *Literature cited*: Contents of this amendment
Item 10	Corrected throughout the Protocol: Minor errors in grammar, typography, formatting
Item 11	Updated throughout the Protocol: Section numbering and cross-references
Item 12	Updated per Protocol Version 5.0, Claification Memo 1, dated May 19, 2022
Item 13	Updated per Protocol Version 5.0, Clarification Memo 2, dated June 29, 2022

Date: June 29, 2022

Protocol version: Version 5.0
Protocol modification: Clarification Memo 2

Item 1	Clarified in Appendix D, *Laboratory Procedures for PBMC Immunogenicity subset, Groups 1 and 3*; Appendix E, *Laboratory Procedures for PBMC Immunogenicity subset, Groups 2 and 4*; Appendix F, *Laboratory Procedures for the PBMC Immune Correlates subset (Group 1, HIV positive, SARS-CoV-2 seronegative participants)*; Appendix G, *Laboratory Procedures for the PBMC Immune Correlates subset (Group 2, HIV positive, SARS-CoV-2 seropositive participants)* and Appendix J, *Symptom visits: Laboratory procedures table*: Alternative to ACD tubes for sample collection
Item 2	Corrected in Appendix E, *Laboratory Procedures for PBMC Immunogenicity subset, Groups 2 and 4*: missing ‘Cellular Assays’ row

Date: May 19, 2022

Protocol version: Version 5.0
Protocol modification: Clarification Memo 1

Item 1	Clarified in Appendix J, *COVID-19 Symptom visits: Clinic procedures* and in Section 9.6*, COVID-19 symptom visits and*

	COVID-19 postdiagnosis schedule: symptom monitoring and SpO2 measurement by participants (if symptomatic)
Item 2	Clarified in Section 9.9, *Delaying vaccinations for a participant;* Appendix B, *Laboratory procedures table for Groups 1 and 3 (SARS-CoV-2 seronegative participants, main study)*; Appendix C, *Laboratory procedures table for Groups 2 and 4 (SARS-CoV-2 seropositive participants, main study);* Appendix D, *Laboratory procedures for PBMC immunogenicity subset, Groups 1 and 3*; Appendix E, *Laboratory procedures for PBMC immunogenicity subset, Groups 2 and 4*; Appendix F, *Laboratory procedures for the PBMC immune correlates subset (Group 1, HIV positive, SARS-CoV-2 seronegative participants)* and Appendix G, *Laboratory procedures for the PBMC immune Correlates subset (Group 2, HIV positive, SARS-CoV-2 seropositive participants)*: change in the dosage of antiretroviral therapy.
Item 3	Revised in Section 2.1, *Rational for trial concept* and Appendix J, *COVID-19 Symptom visits: Clinic procedures:* clarified that post diagnosis follow up is until symptoms improve.
Item 4	Clarified in Section 9.5, *Active COVID-19 symptomatic surveillance*: symptom visit after symptom resolution
Item 5	Clarified in Section 2.1, *Rational for trial concept*; Section 2.1.1, *Overall study design*; Section 2.4, *Study schema and overview of trial design*; Section 3, *Objectives and endpoints;* Section 6.4.3, *Analyses of relative risk between treatment arms*; Section 9.5, *Active COVID-19 symptomatic surveillance*; Section 9.6, *COVID-19 symptom visits and COVID-19 postdiagnosis schedule* and Appendix J*, COVID-19 symptom visits*: harmonized SARS-CoV-2 testing assay description for consistency
Item 6	Clarified in Section 3, *Objectives and endpoints*: randomized assignment to treatment arms
Item 7	Clarified in Appendix K, *Adverse Events of Special Interest (AESI)* and Section 14, *Document references (other than literature citations)*: comprehensive list of AESIs
Item 8	Clarified in Section 9.2, Pre-enrollment procedures: enrollment may be deferred if antiretroviral therapy needs to be initiated
Item 9	Corrected in Appendix H, Schedule of clinic procedures for Groups 1 and 3 (SARS-CoV-2 seronegative participants) and Appendix I, Schedule of clinic procedures for Groups 2 and 4 (SARS-CoV-2 seropositive participants) : removed typographical error

Date: March 6, 2022

Protocol version:5.0
Protocol modification: Full Protocol Amendment 4

Item 1	Revisions to Section 1, *Overview*: study schema, primary objectives, study duration, endpoint assay laboratories, protocol team
Item 2	Revised in Section 2, *Background*: study rationale, SARS-CoV-2 epidemiology, study design, added Appendix G
Item 3	Revised in Section 3, *Objectives and endpoints*: primary, secondary, and exploratory objectives
Item 4	Updated Section 5, *IRB/EC review considerations*: Countermeasures Acceleration Group
Item 5	Revised Section 6, *Statistical considerations*: study groups, analysis cohorts, power calculations for vaccine efficacy analysis
Item 6	Revised in Section 7.1, *Inclusion criteria*: criteria 1 and 6
Item 7	Revised in Section 7.2, *Exclusion criteria:* previously, Criterion #3
Item 8	Revised in Section 8.1, *Vaccine regimen*; Section 8.3, *Acquisition of study products* and Section 8.4, *Administration of study products* : updated to align with current study design
Item 9	Revised in Section 9, *Clinical procedures*: procedures for pregnant participants, deleted randomization at enrollment, added blood collection for PLWH participants if there is a change in antiretroviral (ARV) medication, updated COVID-19 postdiagnosis schedule to monitor persistent shedding, updated Appendices B to J
Item 10	Clarified in Section 10, *Laboratory Procedures*: qualitative and quantitative reverse transcriptase PCRs
Item 11	Revisions to Appendix A, *Sample Informed Consent Form*: Key information, participants groups and procedures tables, blood volumes
Item 12	Updated in Title Page; Section 13, *Version history*; Section 16, *Literature cited*: Updates related to contents of this amendment
Item 13	Consistency in references to the Study, study product and participants
Item 14	Updated or corrected throughout the protocol: Section numbering, cross-references, acronym list, and minor errors
Item 15	Updated per protocol version 4.0, Clarification Memo 2, dated February 9, 2022
Item 16	Updated per protocol version 4.0, Clarification Memo 1, dated December 5, 2021

Date: February 9, 2022

Protocol version:4.0
Protocol modification: Clarification Memo 2

Item 1	Clarified in Section 9.6, *COVID-19 Symptom Visits and COVID-19 post-diagnosis schedule*: participants testing positive for SARS-CoV-2 will follow procedures outlined in Appendix I
Item 2	Updated in Appendix I, *COVID-19 symptom visits* (Clinic Procedures and Laboratory procedures tables): avoid duplication of procedures and sample collections
Item 3	Clarified in Section 9.5, *Active COVID-19 Symptomatic Surveillance*, Appendix G, *Schedule of clinic procedures for Groups 1 and 3* (SARS-CoV-2 seronegative participants) and in Appendix H, S*chedule of clinic procedures for Groups 2 and 4* (SARS-CoV-2 seropositive participants): elicit participant feedback approximately every two weeks.

Date: December 5, 2021

Protocol version:4.0
Protocol modification: Clarification Memo 1

Item 1	Updated in Appendix B, *Laboratory procedures table for Groups 1 and 3* (SARS-CoV-2 seronegative participants, main study): nasal swab collection

Date: October 1, 2021

Protocol version:4.0
Protocol modification: Full Protocol Amendment 3

Item 1	Revisions to Section 1, *Overview*: study schema, primary objectives, study duration, protocol team
Item 2	Updated in Section 2, *Background*: study rationale, SARS-CoV-2 epidemiology, study design
Item 3	Revised in Section 3, *Objectives and endpoints*: primary, secondary, and exploratory objectives
Item 4	Revised in Section 4, *Ethical considerations*: updated language
Item 5	Revised Section 6, *Statistical considerations*: study groups, analysis cohorts, power calculations for vaccine efficacy analysis
Item 6	Revised in Section 7.1, *Inclusion criteria*: criteria 1 and 4
Item 7	Revised in Section 8.1, *Vaccine regimen*: updated vaccine regimen
Item 8	Revised in Section 9, *Clinical procedures*: schedule of procedures, added HIV diagnostic test at screening for all participants, revised vaccination and follow-up procedures

Item 9 Added in Section 10, *Laboratory Procedures*: new sections to describe endpoint assays

Item 10 Reconciled in Section 11, *Expedited reporting of adverse events*: study product name

Item 11 Revisions to Appendix A, *Sample Informed Consent Form*: study questions, study duration, participants groups and procedures tables, blood volumes

Item 12 Revised Appendix B, added new Appendix C, *Laboratory Procedures tables*: reorganized, updated visit schedule, and sample collections

Item 13 Added Appendices D and E, *Laboratory Procedures* tables for PBMC Immunogenicity subset

Item 14 Added new Appendix F, *Laboratory Procedures for the Cellular PBMC Immune Correlates subset* (Group 1, HIV positive, SARS-CoV-2 seronegative participants)

Item 15 Revised Appendix G and H, *Schedule of Clinic Procedures*: added Visit 8, study group-specific schedule, procedures

Item 16 Revised Appendix I, *COVID-19 symptom visits, Laboratory Procedures* Table: blood volume, footnotes

Item 17 Revised Appendix J, *Visit Windows*: added Visit 8 and table for Groups 2 and 4, revised visit windows for Visit 7

Item 18 Clarification Memo 1 to Version 3.0, dated August 25, 2021

Item 19 Updated, Section 13, *Version history*

Item 20 Cross-references, acronym list, and minor errors corrected

Date: August 25, 2021

Protocol version: Version 3.0
Protocol modification: Clarification Memo 1

Item 1 Clarified in Section 9.2, *Pre-enrollment procedures* and Appendix B, *Laboratory procedures table* (main study and PBMC subset): SARS-CoV-2 serology testing at screening

Item 2 Corrected in Appendix E, *Visit windows*: Vaccination 3 target window

Date: August 11, 2021

Protocol version:3.0
Protocol modification: Full Protocol Amendment 2

Item 1 Revisions to Section 1, *Overview,* and *Section 1.1, Protocol Team*: vaccine administration and Protocol Team members

Item 2 Updated in Section 2, *Background*: clarified study design

Item 3 Revised in Section 3, *Objectives and endpoints*: Primary Endpoints 3, secondary endpoints 5, exploratory objectives

Item 4 Revised in Section 4, *Ethical considerations*: vaccine regimen

Item 5 Revised Section 6, *Statistical considerations*: statistical design, definitions, power calculations for vaccine efficacy, analysis

Item 6 Revised in Section 7.1, *Inclusion criteria*: criteria 1 and 6

Item 7 Clarified in Section 8.1, *Vaccine regimen*: updated vaccine regimen

Item 8 Clarified in Section 9.8, *Assessments of reactogenicity for a subset of participants*: timepoints for reactogenicity assessments

Item 9 Clarified in Section 11.2.2, *AE reporting*: timepoints for AE collection

Item 10 Revisions to Appendix A, *SICF*: revised language and reconciled clinic procedures and blood collection volumes

Item 11 Revised Appendix B, *Laboratory Procedures tables*: relabeled visits, revised visit schedule and sample collections

Item 12 Revised Appendix C, Schedule of Clinic Procedures

Item 13 Revised Appendix E, *Visit Windows*: visit target day and visit type

Item 14 Updated, Section 13, *Version history*

Item 15 Cross-references, acronym list, and minor errors corrected

Date: July 15, 2021

Protocol version:2.0
Protocol modification: Full Protocol Amendment 1

Item 1 Revised the CoVPN 3008 protocol name to CoVPN 3008 (Ubuntu)

Item 2 Revisions to Section 1, Overview, and Section 1.1, Protocol Team: Protocol Team members and SDMC

Item 3 Updated in Section 2, Background: name of B.1.351 variant and clarified study design

Item 4 Revised in Section 3, Objectives and endpoints: Primary Endpoints 1, secondary endpoints 5, exploratory objectives and endpoints

Item 5 Revised in Section 4, Ethical considerations: optional unblinding for participants

Item 6 Removed in Section 5.7, Pregnant participants: reference to pregnant women as vulnerable population

Item 7 Added in Section 6.2, Definitions of Study Cohorts for Analysis, Section 6.5.1, Immunogenicity and Immune Correlates Analyses: timepoints for blood collection for cellular assays

Item 8	Clarified in Section 6.4.3, Vaccine Efficacy Analyses: use of statistical methods
Item 9	Added in Section 6.9, Roles of DSMB, OG and EAC: information about EAC
Item 10	Updated in Section 7.1, Inclusion criteria: added reference to CDC
Item 11	Revised in Section 7.2, Exclusion criteria: added criterion # 5, revised criteria # 4, # 6, and #7
Item 12	Clarified in Section 8, Study products: placebo and procedures for administration
Item 13	Revised Section 9, Clinical procedures: data entry into the study database, clarified study procedures
Item 14	Added to Section 10, Laboratory: blood collection for PBMC for pregnant women
Item 15	Revisions to Section 11, Safety monitoring and safety review:
Item 16	Added new section 12.3, Site monitoring
Item 17	Revisions to Appendix A, SICF: reconciled blood volumes collected from participants and revised language
Item 18	Revised Appendix B, Laboratory Procedures tables: blood volume for SARS-CoV-2 Serology (main study and PBMC subset) and cellular assays (PBMC subset)
Item 19	Revised Appendix C, Schedule of Clinic Procedures
Item 20	Revised Appendix D, COVID-19 Symptom Visits, to reflect updates to Section 9.6 and Laboratory procedures
Item 21	Revised Appendix E, Visit Windows: visit target day
Item 22	Updated, Section 13, Version history
Item 23	Cross-references, acronym list, and minor errors corrected

Date: May 16, 2021

Protocol version:1.0
Protocol modification: Not applicable

Original protocol

14 Document references (other than literature citations)

Other documents referred to in this protocol and containing information relevant to the conduct of this study include:

- Assessment of Understanding. Accessible through the CoVPN protocol-specific website.
- CDC COVID-19 Vaccination Program Provider Requirements and Support. Available at https://www.cdc.gov/vaccines/covid-19/vaccination-provider-support.html
- CoVPN 3008 (Ubuntu) Study Specific Procedures. Accessible through the CoVPN protocol-specific website.
- Dangerous Goods Regulations (updated annually), International Air Transport Association. Available for purchase at https://www.iata.org/publications/dgr/Pages/index.aspx
- International Council for Harmonisation of Technical Requirements for Pharmaceuticals for Human Use (ICH) E6 (R2), Guideline for Good Clinical Practice: Section 4.8, Informed consent of trial subjects. Available at http://www.ich.org/page/efficacy-guidelines
- Laboratory Center assay portfolio
- Moderna COVID-19 Vaccine EUA Fact Sheet and Full PI for Vaccination Providers. Available at https://www.modernatx.com/covid19vaccine-eua/.Participants' Bill of Rights and Responsibilities. Accessible through the CoVPN protocol-specific website.
- Moderna COVID-19 Vaccine Bivalent (Original and Omicron BA.4/.5) EUA Fact Sheet for Healthcare Providers Administering Vaccine. Available at https://www.fda.gov/media/161318/download.
- NIH Policy on Reporting Race and Ethnicity Data: Subjects in Clinical Research. Available at https://grants.nih.gov/grants/guide/notice-files/NOT-OD-01-053.html
- Title 21, Code of Federal Regulations, Part 50. Available at http://www.accessdata.fda.gov/scripts/cdrh/cfdocs/cfcfr/CFRSearch.cfm?CFRPart=50
- Title 45, Code of Federal Regulations, Part 46 (2018 requirements). Current requirements available at https://www.hhs.gov/ohrp/regulations-and-policy/regulations/45-cfr-46/index.html

- The Protection of Personal Information Act (POPIA, South Africa, 2013). Available at https://www.gov.za/sites/default/files/gcis_document/201409/3706726-11act4of2013protectionofpersonalinforcorrect.pdf

- Brighton Collaboration, COVID-19 AESI list. Available at https://brightoncollaboration.us/covid-19/.

See Section 16 for literature cited in the background and statistics sections of this protocol.

15 Acronyms and abbreviations

Ab	antibody
ACIP	Advisory Committee on Immunization Practices
AE	adverse event
AESI	Adverse Events of Special Interest
ART	antiretroviral therapy
BAMA	binding antibody multiplex assay
CAB	Community Advisory Board
CDC	US Centers for Disease Control and Prevention
CFR	Code of Federal Regulations
CI	confidence interval
COVID-19	Coronavirus Disease 2019
CoVPN	COVID-19 Prevention Network
CRF	case report form
CRS	clinical research site
DAIDS	Division of AIDS (US NIH)
DHHS	US Department of Health and Human Services
DSMB	Data and Safety Monitoring Board
EAC	Endpoint Adjudication Committee
EAE	adverse event requiring expedited reporting
EC	Ethics Committee
ELISA	enzyme-linked immunosorbent assay
EUA	Emergency use authorization
EUL	Emergency use listing
FAS	Full analysis set
FDA	US Food and Drug Administration
Fred Hutch	Fred Hutchinson Cancer Center
GCP	Good Clinical Practice
GMT	geometric mean titer
HCORE	HHS Coordination Operations and Response Element
HHS	US Department of Health and Human Services
HIV	human immunodeficiency virus
ICH	International Council for Harmonisation of Technical Requirements for Pharmaceuticals for Human Use
IQR	Interquartile range
IS	Immunogenicity subset
IM	Intramuscular
IRB	Institutional Review Board

JNJ	Johnson and Johnson
LC	Laboratory Center
LNP	Lipid nanoparticle
LOC	Leadership and Operations Center
mRNA	Messenger ribonucleic acid
nAb	neutralizing antibody
NAAT	nucleic acid amplification testing
NIAID	National Institute of Allergy and Infectious Diseases (US NIH)
NIH	US National Institutes of Health
OHRP	US Office for Human Research Protections
OG	Oversight Group
OWS	Operation Warp Speed
PCR	polymerase chain reaction
PI	Principal Investigator
PLWH	People living with HIV
PP	per-protocol
RE	regulatory entity
RT-PCR	reverse transcription-polymerase chain reaction
RVE	relative vaccine efficacy
SAE	serious adverse event
SAMRC	South African Medical Research Council
SAP	Statistical Analysis Plan
SCHARP	Statistical Center for HIV/AIDS Research and Prevention
SDMC	statistical and data management center
SS	safety subset
SSP	Study specific procedures
SOP	Standard operating procedure
UW-VL	University of Washington Virology Laboratory
VE	Vaccine efficacy
VL	Viral load
VOC	Variants of Concern

16 Literature cited

1. NBC C. Illinois Reports Highest Vaccine Numbers in Months Following Strong Demand for New COVID Booster Shots. 2022 September 16, 2022.

2. Browning K. Covid Booster Rollout Begins in California With Little Fanfare. The NewYork Times. 2022 September 21, 2022.

3. COVID data tracker [Internet]. US Department of Health and Human Services. 2003 [cited February 13, 2023]. Available from: https://covid.cdc.gov/covid-data-tracker/#vaccinations_vacc-%20people-booster-percent-pop5.

4. Prevention CfDCa. Stay Up to Date with Your Vaccines 2022 [cited 2022 01/16/2022]. Available from: https://www.cdc.gov/coronavirus/2019-ncov/vaccines/stay-up-to-date.html.

5. FDA. January 31, 2022 Approval Letter - SPIKEVAX 2022 [cited 2022 01/31/2022]. Available from: https://www.fda.gov/media/155815/download.

6. FDA. August 23, 2021 Approval Letter - Comirnaty 2021 [cited 2021 08/23/2021]. Available from: https://www.fda.gov/media/151710/download.

7. SeyedAlinaghi S, Karimi A, Pashaei Z, Afzalian A, Mirzapour P, Ghorbanzadeh K, et al. Safety and Adverse Events Related to COVID-19 mRNA Vaccines; a Systematic Review. Arch Acad Emerg Med. 2022;10(1):e41.

8. Baden LR, El Sahly HM, Essink B, Kotloff K, Frey S, Novak R, et al. Efficacy and Safety of the mRNA-1273 SARS-CoV-2 Vaccine. N Engl J Med. 2021;384(5):403-16.

9. Lopez Bernal J, Andrews N, Gower C, Gallagher E, Simmons R, Thelwall S, et al. Effectiveness of Covid-19 Vaccines against the B.1.617.2 (Delta) Variant. New England Journal of Medicine. 2021;385(7):585-94.

10. Hall VJ, Foulkes S, Saei A, Andrews N, Oguti B, Charlett A, et al. COVID-19 vaccine coverage in health-care workers in England and effectiveness of BNT162b2 mRNA vaccine against infection (SIREN): a prospective, multicentre, cohort study. 2021(1474-547X (Electronic)).

11. Self WH, Tenforde MW, Rhoads JP, Gaglani M, Ginde AA, Douin DJ, et al. Comparative Effectiveness of Moderna, Pfizer-BioNTech, and Janssen (Johnson & Johnson) Vaccines in Preventing COVID-19 Hospitalizations Among Adults Without Immunocompromising Conditions — United States, March–August 2021. Morbidity and Mortality Weekly Report. 2021.

12. El Sahly HM, Baden LR, Essink B, Doblecki-Lewis S, Martin JM, Anderson EJ, et al. Efficacy of the mRNA-1273 SARS-CoV-2 Vaccine at Completion of Blinded Phase. New England Journal of Medicine. 2021.

13. Polack FP, Thomas SJ, Kitchin N, Absalon J, Gurtman A, Lockhart S, et al. Safety and Efficacy of the BNT162b2 mRNA Covid-19 Vaccine. New England Journal of Medicine. 2020;383(27):2603-15.

14. Rosenblum HG, Gee J, Liu R, Marquez PL, Zhang B, Strid P, et al. Safety of mRNA vaccines administered during the initial 6 months of the US COVID-19 vaccination programme: an observational study of reports to the Vaccine Adverse Event Reporting System and v-safe. Lancet Infect Dis. 2022;22(6):802-12.

15. Mellor MM, Bast AC, Jones NR, Roberts NW, Ordonez-Mena JM, Reith AJM, et al. Risk of adverse coronavirus disease 2019 outcomes for people living with HIV. AIDS. 2021;35(4):F1-F10.

16. Ssentongo P, Heilbrunn ES, Ssentongo AE, Advani S, Chinchilli VM, Nunez JJ, et al. Epidemiology and outcomes of COVID-19 in HIV-infected individuals: a systematic review and meta-analysis. Sci Rep. 2021;11(1):6283.

17. CDC. COVID-19 Vaccines for People who are Moderately or Severely Immunocompromised 2022 [updated July 20, 2022. Available from: https://www.cdc.gov/coronavirus/2019-ncov/vaccines/recommendations/immuno.html.

18. Kamar N, Abravanel F, Marion O, Couat C, Izopet J, Del Bello A. Three Doses of an mRNA Covid-19 Vaccine in Solid-Organ Transplant Recipients. N Engl J Med. 2021;385(7):661-2.

19. Boyarsky BJ, Werbel WA, Avery RK, Tobian AAR, Massie AB, Segev DL, et al. Antibody Response to 2-Dose SARS-CoV-2 mRNA Vaccine Series in Solid Organ Transplant Recipients. JAMA. 2021;325(21):2204-6.

20. Furer V, Eviatar T, Zisman D, Peleg H, Paran D, Levartovsky D, et al. Immunogenicity and safety of the BNT162b2 mRNA COVID-19 vaccine in adult patients with autoimmune inflammatory rheumatic diseases and in the general population: a multicentre study. Ann Rheum Dis. 2021;80(10):1330-8.

21. Roeker LE, Knorr DA, Thompson MC, Nivar M, Lebowitz S, Peters N, et al. COVID-19 vaccine efficacy in patients with chronic lymphocytic leukemia. Leukemia. 2021;35(9):2703-5.

22. Levy I, Wieder-Finesod A, Litchevsky V, Biber A, Indenbaum V, Olmer L, et al. Immunogenicity and safety of the BNT162b2 mRNA COVID-19 vaccine in people living with HIV-1. Clinical microbiology and infection : the official publication of the European Society of Clinical Microbiology and Infectious Diseases. 2021;27(12):1851-5.

23. Lombardi A, Butta GM, Donnici L, Bozzi G, Oggioni M, Bono P, et al. Anti-spike antibodies and neutralising antibody activity in people living with HIV vaccinated with COVID-19 mRNA-1273 vaccine: a prospective single-centre cohort study. Lancet Reg Health Eur. 2022;13:100287.

24. Aledo MG, Canizares A, Vazquez-Rodriguez P, Castro A, Moldes L, Lopez S, et al. Safety and immunogenicity of SARS-CoV-2 mRNA-1273 and BNT162b2 vaccines in people living with HIV. AIDS. 2022.

25. Frater J, Ewer KJ, Ogbe A, Pace M, Adele S, Adland E, et al. Safety and immunogenicity of the ChAdOx1 nCoV-19 (AZD1222) vaccine against SARS-CoV-2 in HIV infection: a single-arm substudy of a phase 2/3 clinical trial. Lancet HIV. 2021;8(8):e474-e85.

26. Heath PT, Galiza EP, Baxter DN, Boffito M, Browne D, Burns F, et al. Safety and Efficacy of NVX-CoV2373 Covid-19 Vaccine. New England Journal of Medicine. 2021;385(13):1172-83.

27. Cevik M, Grubaugh ND, Iwasaki A, Openshaw P. COVID-19 vaccines: Keeping pace with SARS-CoV-2 variants. Cell. 2021.

28. Vaccines and Related Biological Products Advisory Committee Meeting. FDA Briefing Document, Janssen Ad26.COV2.S Vaccine 2021 [Available from: https://www.fda.gov/media/146217/download.

29. Sadoff J, Gray G, Vandebosch A, Cardenas V, Shukarev G, Grinsztejn B, et al. Safety and Efficacy of Single-Dose Ad26.COV2.S Vaccine against Covid-19. N Engl J Med. 2021;384(23):2187-201.

30. Voysey M, Clemens SAC, Madhi SA, Weckx LY, Folegatti PM, Aley PK, et al. Safety and efficacy of the ChAdOx1 nCoV-19 vaccine (AZD1222) against SARS-CoV-2: an interim analysis of four randomised controlled trials in Brazil, South Africa, and the UK. Lancet. 2021;397(10269):99-111.

31. Tregoning JS, Flight KE, Higham SL, Wang Z, Pierce BF. Progress of the COVID-19 vaccine effort: viruses, vaccines and variants versus efficacy, effectiveness and escape. Nature Reviews Immunology. 2021;21(10):626-36.

32. Andrews N, Stowe J, Kirsebom F, Toffa S, Rickeard T, Gallagher E, et al. Covid-19 Vaccine Effectiveness against the Omicron (B.1.1.529) Variant. N Engl J Med. 2022;386(16):1532-46.

33. Agency UHaS. COVID-19 vaccine surveillance report week 24 2022 [updated June 16, 2022July 29, 2022]. Available from: https://assets.publishing.service.gov.uk/government/uploads/system/uploads/attachment_data/file/1083443/Vaccine-surveillance-report-week-24.pdf.

34. Tseng HF, Ackerson BK, Luo Y, Sy LS, Talarico CA, Tian Y, et al. Effectiveness of mRNA-1273 against SARS-CoV-2 Omicron and Delta variants. Nat Med. 2022;28(5):1063-71.

35. Collie S, Champion J, Moultrie H, Bekker LG, Gray G. Effectiveness of BNT162b2 Vaccine against Omicron Variant in South Africa. N Engl J Med. 2022;386(5):494-6.

36. Chalkias S, Essink, B., Khetan, S., Nestorova, B. et al. Safety, Immunogenicity and Antibody Persistence of

a Bivalent Beta-Containing Booster Vaccine. 2022.

37. Mahase E. Covid-19: UK will roll out Moderna's omicron BA.1 vaccine as part of autumn booster programme. Bmj. 2022;378:o2038.

38. Korber B FW, Gnanakaran S, Yoon H, Theiler J, et al. Spike mutation pipeline reveals the emergence of a more transmissible form of SARS CoV-2. 2020.

39. Salleh MZ, Derrick JP, Deris ZZ. Structural Evaluation of the Spike Glycoprotein Variants on SARS-CoV-2 Transmission and Immune Evasion. Int J Mol Sci. 2021;22(14).

40. Cai Y, Zhang J, Xiao T, Lavine CL, Rawson S, Peng H, et al. Structural basis for enhanced infectivity and immune evasion of SARS-CoV-2 variants. Science. 2021;373(6555):642-8.

41. Tarhini H, Recoing A, Bridier-Nahmias A, Rahi M, Lambert C, Martres P, et al. Long-Term Severe Acute Respiratory Syndrome Coronavirus 2 (SARS-CoV-2) Infectiousness Among Three Immunocompromised Patients: From Prolonged Viral Shedding to SARS-CoV-2 Superinfection. J Infect Dis. 2021;223(9):1522-7.

42. Aydillo T, Gonzalez-Reiche AS, Aslam S, van de Guchte A, Khan Z, Obla A, et al. Shedding of Viable SARS-CoV-2 after Immunosuppressive Therapy for Cancer. N Engl J Med. 2020;383(26):2586-8.

43. Meiring S, Tempia S, Bhiman JN, Buys A, Kleynhans J, Makhasi M, et al. Prolonged shedding of SARS-CoV-2 at high viral loads amongst hospitalised immunocompromised persons living with HIV, South Africa. Clin Infect Dis. 2022.

44. Leung WF, Chorlton S, Tyson J, Al-Rawahi GN, Jassem AN, Prystajecky N, et al. COVID-19 in an immunocompromised host: persistent shedding of viable SARS-CoV-2 and emergence of multiple mutations: a case report. Int J Infect Dis. 2022;114:178-82.

45. Cele S, Karim F, Lustig G, San JE, Hermanus T, Tegally H, et al. SARS-CoV-2 prolonged infection during advanced HIV disease evolves extensive immune escape. Cell Host Microbe. 2022;30(2):154-62 e5.

46. Weigang S, Fuchs J, Zimmer G, Schnepf D, Kern L, Beer J, et al. Within-host evolution of SARS-CoV-2 in an immunosuppressed COVID-19 patient as a source of immune escape variants. Nat Commun. 2021;12(1):6405.

47. UNAIDS. UNAIDS Global AIDS Update 2022 2022 [Available from: https://www.unaids.org/sites/default/files/media_asset/2022-global-aids-update_en.pdf.

48. Madhi SA, Kwatra G., Myers, J.E., Jassat, W., Dhar, N., Mukendi, C.K., Nana, A.J., Blumberg, L., Welch, R., Ngorima-Mabhena, N., and Mutevedzi, P.C. South African Population Immunity and Severe Covid-19 with Omicron Variant. 2022.

49. Bingham J, Cable R, Coleman C, Glatt TN, Grebe E, Mhlanga L, et al. Estimates of prevalence of anti-SARS-CoV-2 antibodies among blood donors in South Africa in March 2022. Res Sq. 2022.

50. Crotty S. Hybrid immunity. COVID-19 vaccine responses provide insights

into how the immune system perceives threats. Science. 2022;372(6549).

51. Crotty S. Hybrid immunity. Science. 2021;372(6549):1392-3.

52. Stamatatos L, Czartoski J, Wan Y-H, Homad LJ, Rubin V, Glantz H, et al. mRNA vaccination boosts cross-variant neutralizing antibodies elicited by SARS-CoV-2 infection. Science. 2021;372(6549):1413-8.

53. Reynolds CJ, Pade C, Gibbons JM, Butler DK, Otter AD, Menacho K, et al. Prior SARS-CoV-2 infection rescues B and T cell responses to variants after first vaccine dose. Science. 2021;372(6549):1418-23.

54. Saadat S, Rikhtegaran Tehrani Z, Logue J, Newman M, Frieman MB, Harris AD, et al. Binding and Neutralization Antibody Titers After a Single Vaccine Dose in Health Care Workers Previously Infected With SARS-CoV-2. JAMA. 2021;325(14):1467-9.

55. Blain H, Tuaillon E, Gamon L, Pisoni A, Miot S, Picot M-C, et al. Spike Antibody Levels of Nursing Home Residents With or Without Prior COVID-19 3 Weeks After a Single BNT162b2 Vaccine Dose. JAMA. 2021;325(18):1898-9.

56. Ebinger JE, Fert-Bober J, Printsev I, Wu M, Sun N, Prostko JC, et al. Antibody responses to the BNT162b2 mRNA vaccine in individuals previously infected with SARS-CoV-2. Nature Medicine. 2021;27(6):981-4.

57. Lin DY, Zeng D, Mehrotra DV, Corey L, Gilbert PB. Evaluating the Efficacy of COVID-19 Vaccines. Clin Infect Dis. 2020.

58. Rid A, Saxena A, Baqui AH, Bhan A, Bines J, Bouesseau MC, et al. Placebo use in vaccine trials: recommendations of a WHO expert panel. Vaccine. 2014;32(37):4708-12.

59. Wendler D, Ochoa J, Millum J, Grady C, Taylor HA. COVID-19 vaccine trial ethics once we have efficacious vaccines. Science. 2020;370(6522):1277-9.

60. Rouchka EC, Chariker JH, Chung D. Variant analysis of 1,040 SARS-CoV-2 genomes. PLoS One. 2020;15(11):e0241535.

61. Alam I, Radovanovic A, Incitti R, Kamau AA, Alarawi M, Azhar EI, et al. CovMT: an interactive SARS-CoV-2 mutation tracker, with a focus on critical variants. The Lancet Infectious Diseases. 2021;21(5):602.

62. Hoffmann M, Arora P, Groß R, Seidel A, Hörnich BF, Hahn AS, et al. SARS-CoV-2 variants B.1.351 and P.1 escape from neutralizing antibodies. (1097-4172 (Electronic)).

63. McCallum M Fau - Bassi J, Bassi J Fau - Marco AD, Marco A Fau - Chen A, Chen A Fau - Walls AC, Walls Ac Fau - Iulio JD, Iulio Jd Fau - Tortorici MA, et al. SARS-CoV-2 immune evasion by variant B.1.427/B.1.429. LID - 2021.03.31.437925 [pii] LID - 10.1101/2021.03.31.437925 [doi].

64. Planas D, Veyer D, Baidaliuk AA-O, Staropoli I, Guivel-Benhassine F, Rajah MA-O, et al. Reduced sensitivity of SARS-CoV-2 variant Delta to antibody neutralization. (1476-4687 (Electronic)).

65. Wang P, Nair MA-O, Liu L, Iketani SA-O, Luo Y, Guo YA-OX, et al. Antibody resistance of SARS-CoV-2 variants B.1.351 and B.1.1.7. (1476-4687 (Electronic)).

66. Wang P, Nair MS, Liu L, Iketani S, Luo Y, Guo Y, et al. Antibody resistance of SARS-CoV-2 variants B.1.351 and B.1.1.7. Nature. 2021;593(7857):130-5.

67. Planas D, Veyer D, Baidaliuk A, Staropoli I, Guivel-Benhassine F, Rajah MM, et al. Reduced sensitivity of SARS-CoV-2 variant Delta to antibody neutralization. Nature. 2021;596(7871):276-80.

68. McCallum M, Bassi J, De Marco A, Chen A, Walls AC, Di Iulio J, et al. SARS-CoV-2 immune evasion by the B.1.427/B.1.429 variant of concern. Science. 2021;373(6555):648-54.

69. Hoffmann M, Arora P, Gross R, Seidel A, Hornich BF, Hahn AS, et al. SARS-CoV-2 variants B.1.351 and P.1 escape from neutralizing antibodies. Cell. 2021;184(9):2384-93 e12.

70. Wu K, Werner AP, Koch M, Choi A, Narayanan E, Stewart-Jones GBE, et al. Serum Neutralizing Activity Elicited by mRNA-1273 Vaccine. N Engl J Med. 2021;384(15):1468-70.

71. Boehm E, Kronig I, Neher RA, Eckerle I, Vetter P, Kaiser L. Novel SARS-CoV-2 variants: the pandemics within the pandemic. Clin Microbiol Infect. 2021;27(8):1109-17.

72. Korber B, Fischer WM, Gnanakaran S, Yoon H, Theiler J, Abfalterer W, et al. Tracking Changes in SARS-CoV-2 Spike: Evidence that D614G Increases Infectivity of the COVID-19 Virus. Cell. 2020;182(4):812-27 e19.

73. Tao K, Tzou PL, Nouhin J, Gupta RK, de Oliveira T, Kosakovsky Pond SL, et al. The biological and clinical significance of emerging SARS-CoV-2 variants. Nat Rev Genet. 2021;22(12):757-73.

74. Davies NG, Abbott S, Barnard RC, Jarvis CI, Kucharski AJ, Munday JD, et al. Estimated transmissibility and impact of SARS-CoV-2 lineage B.1.1.7 in England. Science. 2021;372(6538).

75. Davies NG, Jarvis CI, van Zandvoort K, Clifford S, Sun FY, Funk S, et al. Increased mortality in community-tested cases of SARS-CoV-2 lineage B.1.1.7. Nature. 2021;593(7858):270-4.

76. Graham MS, Sudre CH, May A, Antonelli M, Murray B, Varsavsky T, et al. Changes in symptomatology, reinfection, and transmissibility associated with the SARS-CoV-2 variant B.1.1.7: an ecological study. Lancet Public Health. 2021;6(5):e335-e45.

77. GSAID. Overview of Variants in Countries 2022 [updated August 25, 2022. Available from: https://covariants.org/per-country.

78. Wilton T, Bujaki E, Klapsa D, Majumdar M, Zambon M, Fritzsche M, et al. Rapid Increase of SARS-CoV-2 Variant B.1.1.7 Detected in Sewage Samples from England between October 2020 and January 2021. mSystems. 2021;6(3):e0035321.

79. Faria NR, Mellan TA, Whittaker C, Claro IM, Candido DDS, Mishra S, et al. Genomics and epidemiology of the P.1 SARS-CoV-2 lineage in Manaus, Brazil. Science. 2021;372(6544):815-21.

80. Moyo-Gwete T, Madzivhandila M, Makhado Z, Ayres F, Mhlanga D, Oosthuysen B, et al. Cross-Reactive Neutralizing Antibody Responses Elicited by SARS-CoV-2 501Y.V2 (B.1.351). N Engl J Med. 2021;384(22):2161-3.

81. Souza WM, Amorim MR, Sesti-Costa R, Coimbra LD, Brunetti NS, Toledo-Teixeira DA, et al. Neutralisation of SARS-CoV-2 lineage P.1 by antibodies elicited through natural SARS-CoV-2 infection or vaccination with an inactivated SARS-CoV-2 vaccine: an immunological study. Lancet Microbe. 2021;2(10):e527-e35.

82. Liu Y, Liu J, Johnson BA, Xia H, Ku Z, Schindewolf C, et al. Delta spike P681R mutation enhances SARS-CoV-2 fitness over Alpha variant. Cell Rep. 2022;39(7):110829.

83. Saito A, Irie T, Suzuki R, Maemura T, Nasser H, Uriu K, et al. Enhanced fusogenicity and pathogenicity of SARS-CoV-2 Delta P681R mutation. Nature. 2022;602(7896):300-6.

84. Bolze A, Luo S, White S, Cirulli ET, Wyman D, Dei Rossi A, et al. SARS-CoV-2 variant Delta rapidly displaced variant Alpha in the United States and led to higher viral loads. Cell Rep Med. 2022;3(3):100564.

85. Dhar MS, Marwal R, Vs R, Ponnusamy K, Jolly B, Bhoyar RC, et al. Genomic characterization and epidemiology of an emerging SARS-CoV-2 variant in Delhi, India. Science. 2021;374(6570):995-9.

86. Sheikh A, McMenamin J, Taylor B, Robertson C. SARS-CoV-2 Delta VOC in Scotland: demographics, risk of hospital admission, and vaccine effectiveness. Lancet. 2021;397(10293):2461-2.

87. Callaway E. Heavily mutated Omicron variant puts scientists on alert. Nature. 2021;600(7887):21.

88. Martin DP, Lytras S, Lucaci AG, Maier W, Gruning B, Shank SD, et al. Selection analysis identifies unusual clustered mutational changes in Omicron lineage BA.1 that likely impact Spike function. bioRxiv. 2022.

89. Harvey WT, Carabelli AM, Jackson B, Gupta RK, Thomson EC, Harrison EM, et al. SARS-CoV-2 variants, spike mutations and immune escape. Nat Rev Microbiol. 2021;19(7):409-24.

90. Cui Z, Liu P, Wang N, Wang L, Fan K, Zhu Q, et al. Structural and functional characterizations of infectivity and immune evasion of SARS-CoV-2 Omicron. Cell. 2022;185(5):860-71.e13.

91. Schmidt F, Muecksch F, Weisblum Y, Da Silva J, Bednarski E, Cho A, et al. Plasma Neutralization of the SARS-CoV-2 Omicron Variant. N Engl J Med. 2022;386(6):599-601.

92. Dejnirattisai W, Huo J, Zhou D, Zahradnik J, Supasa P, Liu C, et al. SARS-CoV-2 Omicron-B.1.1.529 leads to widespread escape from neutralizing antibody responses. Cell. 2022;185(3):467-84 e15.

93. Mallapaty S. Where did Omicron come from? Three key theories. Nature. 2022;602(7895):26-8.

94. Today E. Egypt announces first Coronavirus infection. Egypt Today. 2020 February 14, 2020.

95. Reuters. Remote Lesotho becomes last country in Africa to record COVID-19 case. Reuters; 2020 May 13, 2020.

96. Tegally H, Wilkinson E, Giovanetti M, Iranzadeh A, Fonseca V, Giandhari J, et al. Detection of a SARS-CoV-2 variant of concern in South Africa. Nature. 2021;592(7854):438-43.

97. Tessema SK, Nkengasong JN. Understanding COVID-19 in Africa. Nat Rev Immunol. 2021;21(8):469-70.

98. News U. Delta variant drives Africa COVID threat to ‘whole new level′: WHO warns; ‘dominant’ in Europe by August. United Nations; 2021 July 01, 2021.

99. Princewill N. Delta variant has spiked Covid-19 deaths in Africa by 80% in one month, WHO says. CNN2021.

100. Mamoon N, Rasskin, G. Covidvisualizer. 2021.

101. Lab GCD. Our World in Data. 2021.

102. VAERS. Vaccine Adverse Event Reporting System 2021 [Available from: https://vaers.hhs.gov/.

103. Control ECfDPa. COVID-19 Vaccine Tracker 2021 [Available from: https://vaccinetracker.ecdc.europa.eu/public/extensions/COVID-19/vaccine-tracker.html#distribution-tab.

104. Mathieu E, Ritchie H, Ortiz-Ospina E, Roser M, Hasell J, Appel C, et al. A global database of COVID-19 vaccinations. Nat Hum Behav. 2021;5(7):947-53.

105. Alliance GTV. Gavi and Moderna reach agreement for additional supply to COVAX Gavi2021 [updated December 10, 2021. Available from: https://www.gavi.org/news/media-room/gavi-and-moderna-reach-agreement-additional-supply-covax.

106. Jackson LA, Anderson EJ, Rouphael NG, Roberts PC, Makhene M, Coler RN, et al. An mRNA Vaccine against SARS-CoV-2 - Preliminary Report. N Engl J Med. 2020.

107. Anderson EJ, Rouphael NG, Widge AT, Jackson LA, Roberts PC, Makhene M, et al. Safety and Immunogenicity of SARS-CoV-2 mRNA-1273 Vaccine in Older Adults. N Engl J Med. 2020;383(25):2427-38.

108. Vaccines and Related Biological Products Advisory Committee Meeting. FDA Briefing Document, Moderna COVID-19 Vaccine 2020 [Available from: https://www.fda.gov/media/144434/download.

109. Vaccines and Related Biological Products Advisory Committee Meeting. Sponsor Briefing Document, Moderna COVID-19 Vaccine 2020 [Available from: https://www.fda.gov/media/144452/download.

110. Products VaRB, Committee A. mRNA-1273 Sponsor Briefing Document Addendum: Moderna Tx; 2020 [updated 12/17/2020. Available from: https://www.fda.gov/media/144453/download.

111. Ai J, Zhang H, Zhang Y, Lin K, Zhang Y, Wu J, et al. Omicron variant showed lower neutralizing sensitivity than other SARS-CoV-2 variants to immune sera elicited by vaccines after boost. Emerg Microbes Infect. 2022;11(1):337-43.

112. Abu-Raddad LJ, Chemaitelly H, Ayoub HH, AlMukdad S, Yassine HM, Al-Khatib HA, et al. Effect of mRNA Vaccine Boosters against SARS-CoV-2 Omicron Infection in Qatar. N Engl J Med. 2022;386(19):1804-16.

113. Chalkias S, Harper C, Vrbicky K, Walsh SR, Essink B, Brosz A, et al. A Bivalent Omicron-containing Booster Vaccine Against Covid-19. medRxiv. 2022:2022.06.24.22276703.

114. CDC. Booster Doses of Moderna COVID-19 Vaccines in Adults,

Adolescents & Children ACIP2022 [updated September 01, 2022. Available from: https://www.cdc.gov/vaccines/acip/meetings/downloads/slides-2022-09-01/06-COVID-Miller-508.pdf.

115. Miller JM, editor Booster doses of Moderna COVID-19 vaccines in adults, adolescents & children2022; Atlanta, GA.

116. Statista. Number of COVID-19 vaccine doses administered in the United States as of August 24, 2022, by vaccine manufacturer 2022 [updated August 24, 2022. Available from: https://www.statista.com/statistics/1198516/covid-19-vaccinations-administered-us-by-company/ Accessed 21 August 2022.

117. Hause AM, Baggs J, Marquez P, Abara WE, Baumblatt JG, Thompson D, et al. Safety Monitoring of COVID-19 mRNA Vaccine First Booster Doses Among Persons Aged ≥12 Years with Presumed Immunocompromise Status - United States, January 12, 2022-March 28, 2022. MMWR Morb Mortal Wkly Rep. 2022;71(28):899-903.

118. González de Aledo M, Cañizares A, Vázquez-Rodríguez P, Castro Á, Moldes L, López S, et al. Safety and Immunogenicity of SARS-CoV-2 vaccines in people with HIV. Aids. 2022;36(5):691-5.

119. Vergori A, Cozzi Lepri A, Cicalini S, Matusali G, Bordoni V, Lanini S, et al. Immunogenicity to COVID-19 mRNA vaccine third dose in people living with HIV. Nat Commun. 2022;13(1):4922.

120. Klein NP, Lewis N, Goddard K, Fireman B, Zerbo O, Hanson KE, et al. Surveillance for Adverse Events After COVID-19 mRNA Vaccination. JAMA. 2021;326(14):1390-9.

121. Niesen MJM, Pawlowski C, O'Horo JC, Challener DW, Silvert E, Donadio G, et al. Surveillance of Safety of 3 Doses of COVID-19 mRNA Vaccination Using Electronic Health Records. JAMA Netw Open. 2022;5(4):e227038.

122. Hause AM, Baggs J, Marquez P, Myers TR, Su JR, Blanc PG, et al. Safety Monitoring of COVID-19 Vaccine Booster Doses Among Adults - United States, September 22, 2021-February 6, 2022. MMWR Morb Mortal Wkly Rep. 2022;71(7):249-54.

123. FDA. Coronavirus (COVID-19) Update: FDA Authorizes Moderna, Pfizer-BioNTech Bivalent COVID-19 Vaccines for Use as a Booster Dose FDA News Release2022 [updated August 31, 2022. Available from: https://www.fda.gov/news-events/press-announcements/coronavirus-covid-19-update-fda-authorizes-moderna-pfizer-biontech-bivalent-covid-19-vaccines-use.

124. Hause AM, Marquez P, Zhang B, Myers TR, Gee J, Su JR, et al. Safety Monitoring of Bivalent COVID-19 mRNA Vaccine Booster Doses Among Persons Aged >/=12 Years - United States, August 31-October 23, 2022. MMWR Morb Mortal Wkly Rep. 2022;71(44):1401-6.

125. Mueller BaL, S. Covid Vaccines Targeting Omicron Should Be Standard, Panel Says. The New York Times. 2023 January 26, 2023.

126. Werbel WA, Boyarsky BJ, Ou MT, Massie AB, Tobian AAR, Garonzik-Wang JM, et al. Safety and Immunogenicity of a Third Dose of SARS-CoV-2 Vaccine in Solid Organ Transplant Recipients: A Case Series. Annals of Internal Medicine. 2021;174(9):1330-2.

127. Westhoff TH, Seibert FS, Anft M, Blazquez-Navarro A, Skrzypczyk S, Zgoura P, et al. A third vaccine dose substantially improves humoral and cellular SARS-CoV-2 immunity in renal transplant recipients with primary humoral nonresponse. Kidney international. 2021:S0085-2538(21)00850-4.

128. A Trial of the Safety and Immunogenicity of the COVID-19 Vaccine (mRNA-1273) in Participants With Hematologic Malignancies and Various Regimens of Immunosuppression, and in Participants With Solid Tumors on PD1/PDL1 Inhibitor Therapy; NCT04847050 [Internet]. 2021. Available from: https://clinicaltrials.gov/ct2/show/NCT04847050.

129. A Study to Evaluate Safety and Immunogenicity of mRNA-1273 Vaccine to Prevent COVID-19 in Adult Organ Transplant Recipients and in Healthy Adult Participants; NCT04860297 [Internet]. 2021. Available from: https://clinicaltrials.gov/ct2/show/NCT04860297.

130. Third Dose of Moderna COVID-19 Vaccine in Transplant Recipients; NCT04885907 [Internet]. 2021. Available from: https://www.clinicaltrials.gov/ct2/show/NCT04885907.

131. Chapin-Bardales J, Myers T, Gee J, Shay DK, Marquez P, Baggs J, et al. Reactogenicity within 2 weeks after mRNA COVID-19 vaccines: Findings from the CDC v-safe surveillance system. Vaccine. 2021;39(48):7066-73.

132. Prevention CfDCa. Updates on myocarditis and pericarditis following Moderna COVID-19 vaccination 2022 [updated 04 February, 2022. Available from: https://www.cdc.gov/vaccines/acip/meetings/downloads/slides-2022-02-04/03-COVID-Shimabukuro-508.pdf.

133. Singer ME, Taub IB, Kaelber DC. Risk of Myocarditis from COVID-19 Infection in People Under Age 20: A Population-Based Analysis. medRxiv. 2021:2021.07.23.21260998.

134. Choi A, Koch M, Wu K, Chu L, Ma L, Hill A, et al. Safety and immunogenicity of SARS-CoV-2 variant mRNA vaccine boosters in healthy adults: an interim analysis. Nat Med. 2021;27(11):2025-31.

135. Chapin-Bardales J, Gee J, Myers T. Reactogenicity Following Receipt of mRNA-Based COVID-19 Vaccines. JAMA. 2021.

136. Hause AM BJ, Gee J, et al. Safety Monitoring of an Additional Dose of COVID-19 Vaccine — United States, August 12–September 19, 2021.; 2021.

137. Gray KJ, Bordt EA, Atyeo C, Deriso E, Akinwunmi B, Young N, et al. COVID-19 vaccine response in pregnant and lactating women: a cohort study. Am J Obstet Gynecol. 2021.

138. Prevention CfDCa. v-safe COVID-19 Vaccine Pregnancy Registry 2021 [updated 2021, March 15. Available from: https://www.cdc.gov/coronavirus/2019-ncov/vaccines/safety/vsafepregnancyregistry.html.

139. Shimabukuro TT, Kim SY, Myers TR, Moro PL, Oduyebo T, Panagiotakopoulos L, et al. Preliminary Findings of mRNA Covid-19 Vaccine Safety in Pregnant Persons. N Engl J Med. 2021;384(24):2273-82.

140. NIH. COVID-19 vaccination does not reduce chances of conception, study suggests 2022 [updated January 20, 2022. Available from: https://www.nih.gov/news-events/news-releases/covid-19-vaccination-does-not-reduce-chances-conception-study-suggests.

141. Dandachi D, Geiger G, Montgomery MW, Karmen-Tuohy S, Golzy M, Antar AAR, et al. Characteristics, Comorbidities, and Outcomes in a Multicenter Registry of Patients with HIV and Coronavirus Disease-19. Clin Infect Dis. 2020.

142. Geretti AM, Stockdale AJ, Kelly SH, Cevik M, Collins S, Waters L, et al. Outcomes of COVID-19 related hospitalization among people with HIV in the ISARIC WHO Clinical Characterization Protocol (UK): a prospective observational study. Clin Infect Dis. 2020.

143. Hadi YB, Naqvi SFZ, Kupec JT, Sarwari AR. Characteristics and outcomes of COVID-19 in patients with HIV: a multicentre research network study. AIDS. 2020;34(13):F3-F8.

144. (CDC) CfDCaP. Symptoms of COVID-19 2022 [updated October 26, 2022. Available from: https://www.cdc.gov/coronavirus/2019-ncov/symptoms-testing/symptoms.html.

145. NIAID-RML. Coronavirus Disease 2019 (COVID-19) Treatment Guidelines: National Institutes of Health (NIH); 2023 [updated January 30, 2023. Available from: https://www.covid19treatmentguidelines.nih.gov/.

146. UNAIDS. Ethical considerations in biomedical HIV prevention trials. 2007 7/2007.

147. The National Commission for the Protection of Human Subjects of Biomedical and Behavioral Research. The Belmont Report: Ethical Principles and Guidelines for the Protection of Human Subjects of Research. 1979 4/18/1979.

148. Council for International Organizations of Medical Sciences (CIOMS). International ethical guidelines for biomedical research involving human subjects. Bull Med Ethics. 2002(182):17-23.

149. Madhi SA, Baillie V, Cutland CL, Voysey M, Koen AL, Fairlie L, et al. Efficacy of the ChAdOx1 nCoV-19 Covid-19 Vaccine against the B.1.351 Variant. N Engl J Med. 2021.

150. Pulliam JRC, van Schalkwyk C, Govender N, von Gottberg A, Cohen C, Groome MJ, et al. Increased risk of SARS-CoV-2 reinfection associated with emergence of Omicron in South Africa. Science. 2022;376(6593):eabn4947.

151. Nunes MC, Mbotwe-Sibanda S, Baillie VL, Kwatra G, Aguas R, Madhi SA, et al. SARS-CoV-2 Omicron Symptomatic Infections in Previously Infected or Vaccinated South African Healthcare Workers. Vaccines (Basel). 2022;10(3).

152. Mallapaty S. COVID reinfections surge during Omicron onslaught. Nature. 2022.

153. Altarawneh HN, Chemaitelly H, Hasan MR, Ayoub HH, Qassim S, AlMukdad S, et al. Protection against the Omicron Variant from Previous SARS-CoV-2 Infection. N Engl J Med. 2022;386(13):1288-90.

154. Datta S, Halloran ME, Longini IM, Jr. Efficiency of estimating vaccine efficacy for susceptibility and infectiousness: randomization by individual versus household. Biometrics. 1999;55(3):792-8.

155. Mehrotra DV, Heyse JF. Use of the false discovery rate for evaluating clinical safety data. Stat Methods Med Res. 2004;13(3):227-38.

Appendix A Sample informed consent form

Title: Multi-Center, Randomized, Efficacy Study of COVID-19 mRNA Vaccine in Regions with SARS-CoV-2 Variants of Concern

Study number: CoVPN 3008

Site: [Insert site name]

Thank you for your interest in our research study. It is called CoVPN 3008 (Ubuntu) or the Ubuntu Study. This informed consent form will tell you more about the study. Please read it carefully as you decide if you want to join. If you have questions, please ask us. At the end, we will ask you to answer a few questions to make sure we have explained everything clearly.

If you decide to join this study, we will ask you to sign this form. We will offer you a copy of this form to keep.

CoVPN 3008 (Ubuntu) is a research study testing the Moderna COVID-19 vaccine. We already know that this vaccine reduces the symptoms and severity of COVID-19. It is widely used in the United States and many other countries. We want to know how many doses of vaccine are needed for protection against COVID-19 for adults living with HIV and adults with existing health conditions that may put them at risk for severe COVID-19. We also want to know if people who have already had COVID-19 (and likely have some immunity) need as many vaccine doses as other people to obtain strong protection from COVID-19.

Research is not the same as medical care. The purpose of a research study is to answer scientific questions. We hope that what we learn will help people in the future. If you have access to a COVID-19 vaccine you should consider the risks and benefits of getting a COVID-19 vaccine as part of your government's vaccine rollout or by joining a clinical trial.

Key information

- Joining this research study is voluntary. It is your choice. There may be other options for you, like joining your government national rollout.

- Our scientific questions are: How many doses of vaccine are needed for protection against COVID-19 for adults living with HIV and adults with existing health conditions that may put them at risk of severe COVID-19? And do people who previously had COVID-19 need as many vaccine doses as other people to obtain good protection against COVID-19?

- The vaccine we are using in this study has been approved by the United States Food and Drug Administration (FDA) and has an Emergency Use Listing

(EUL) by the World Health Organization. More than 206 million doses of vaccine has been given to people in the United States and has been shown to work against the original strain of SARS-CoV-2. We are testing this vaccine in our countries to see how well it works against the variants that circulate in our region. These variants were not present in the US when the vaccine was first tested.

- If you join, your participation in this study will last for about 18 months.
- If you get a positive COVID-19 test result while in this study, we will monitor your health and help you get the care you need. You will need to self-isolate at home away from others. You may also need additional medical care including hospitalization. The clinic staff may come to your home to meet with you and collect the samples for this study as needed.
- If you join, we will ask you to have injections, blood draws, and swabs of your nose.
- Here are the risks of taking part:
    - The most common risk of vaccination are symptoms such as muscle aches, fevers or headaches after getting the study vaccine.
    - There are other risks. We will tell you more about them later in this consent form.
- We know that some vaccines do not work as well in people living with HIV. We want to know if this vaccine will work well in people living with HIV.
- We know that sometimes people who have been infected with a virus are protected against that virus if they get it again. We want to know if people with past COVID-19 infection need as many vaccine doses as other people to have good protection against COVID-19.
- While you are in this study, you should still follow local COVID-19 prevention guidelines.

About the study

The COVID-19 Prevention Network (CoVPN) is doing a study to test a vaccine against SARS-CoV-2, the virus that causes COVID-19. This is the virus that causes the disease called COVID-19. Up to 15,600 people will take part in this study. We will recruit people from many clinics in Eastern and Southern Africa. The researcher in charge of this study at this clinic is [Insert name of site PI]. The US Government is paying for this study through funding from the National Institute of Allergy and Infectious Diseases (NIAID).

The vaccine we will use in this study is the Moderna mRNA-1273 vaccine. From here on we will call it the study vaccine.

The study vaccine used in this trial has been given to over 15,000 people in other research studies and over 206 million doses have been used to vaccinate people in the United States. More than 141 million doses have been given to people in Europe under Conditional Marketing Authorization (CMA). The study vaccine is on the Emergency Use Listing by the World Health Organization which recommends its use to prevent COVID-19 disease. More than 906 million doses of mRNA-1273 vaccine made by Pfizer/BioNTech, which is very similar to our study vaccine, has been given to more than 400 million people in the US and Europe. The person giving you the study vaccine can answer any questions you might have about it.

We already know the study vaccine can prevent serious COVID-19 disease in people from the Unites States. It was found to be over 90% effective in preventing symptomatic Covid-19 illness caused by the initial strains of the virus in the US and Europe. We want to test the study vaccine in people who may have a higher chance of getting severe COVID-19 illness. For that reason, we are inviting people who are 18 years or older to join the study, and have HIV, or at least 1 existing health issue that puts them at risk for severe COVID-19 (for example diabetes, obesity, heart or kidney problems, cancer, etc.).

The study vaccine was developed by ModernaTX, Inc. Typical vaccines for viruses are made from a weakened or killed virus, but the study vaccine is not made from the SARS-CoV-2 virus. The study vaccine includes a short segment of messenger ribonucleic acid (mRNA). The mRNA is a genetic code that tells cells how to make a protein. This mRNA is made in a laboratory. When injected into the body, the mRNA vaccine causes some cells to make that viral protein, which can trigger the immune system. Your immune system protects you from disease. If a person is later infected, their immune system remembers the protein from the vaccine which may help it to fight the virus. It is impossible for the vaccine to give you SARS-CoV-2 or COVID-19 infection.

People living with HIV have higher risk of severe COVID-19. Data from earlier studies have shown that mRNA vaccines appear to be safe for people living with HIV. Current advice in the US is that people living with HIV should get the vaccine. We do not know if people living with HIV need more vaccine doses that other people to have good protection from COVID-19. And we do not know if people who previously had COVID-19 need as many vaccine doses as other people to obtain good protection from COVID-19.

The safety of the mRNA vaccines in pregnant women and their babies is being looked at by health authorities in many countries. No safety problems have been reported so far in many thousands of pregnant women, and health authorities recommend vaccination of pregnant women to prevent COVID-19. Breastfeeding

after vaccination has also been shown to be safe and may protect babies from COVID-19.

Risks of the Moderna COVID-19 Vaccine:

To date, millions of people have already received the study vaccine. In clinical trials, most people who got the study vaccine had some reaction after their injections, especially after the second injection. In most people the reaction to the study vaccine didn't affect their daily lives and it went away after 2 or 3 days. Most of these people said they had pain in the arm where they got the injection. These people also felt tired, had headaches, muscle and joint pain, and chills. A much smaller number of these people said they had redness or swelling where the needle went in their arm. Some people also said they had nausea, fever, and swelling or pain in their arm pit.

A small number of people who got the study vaccine had myocarditis (inflammation of the heart muscle) and pericarditis (inflammation of the lining outside the heart). The chance of having this occur is very low. It was seen more often in people less than 20 years of age. Symptoms began within a few days after getting the second dose. It did not last long and most people got better. You should tell us right away if you have chest pain, shortness of breath, or the feeling of a fast-beating, fluttering, or pounding heart, as you may need medical care.

The side effects of the study vaccine seen so far are the same as what have been seen with most vaccines. Generally, vaccines can cause fever, chills, rash, aches and pains, nausea, headache, dizziness, and feeling tired. Vaccines can also cause pain, redness, swelling, or itching where you got the injection. Most people can still do their planned activities after getting a vaccine. Rarely, people have side effects that limit their normal activities or make them go to the doctor.

Rarely, a vaccine can cause you to have an allergic reaction. You might have a rash, hives, or trouble breathing. Allergic reactions can be life-threatening. Tell us if you have ever had a bad reaction to any injection or vaccine.

There may be other risks that we don't yet know about, even serious ones. We will tell you if we learn about any new risks.

Joining the study

1. **You will receive either 2 or 3 doses of the study vaccine.**

   Everyone who joins the study will get the study vaccine. There are 4 groups in this study. The groups differ in the number of doses of study vaccine you will get. The groups are organized by whether or not people are living with HIV and whether or not people have evidence of prior SARS-CoV-2 infection in their blood.

Group 1 includes people living with HIV and Group 3 includes people who are not living with HIV. All people in Groups 1 and 3 will have no evidence of prior SARS-CoV-2 infection in their blood. If you are in these groups, you will get 3 doses of the study vaccine.

Group 2 includes people living with HIV and Group 4 includes people who are not living with HIV. All people in Groups 2 and 4 will have evidence of prior SARS-CoV-2 infection in their blood. If you are in these groups, you will get 2 doses of the study vaccine.

2. **It is completely up to you whether or not to join this study.**

We know you may have access to other COVID-19 vaccines outside of this study (for example through a national rollout). If you do, we urge you to carefully think about whether joining this study is right for you. Take your time in deciding. If it helps, talk to people you trust, such as your doctor, friends or family. If you decide not to join this study, or if you leave it after you have joined, it will not change your healthcare. If you do join, you do not give up your legal rights.

You cannot be in this study while you are in another study where you get a study product. *Site: Modify or delete the following sentences as appropriate.* We check to make sure that you are not in more than 1 study by taking your fingerprint on an electronic system. This information is only accessed by a few members of the clinic staff using a secure password. We might also check the government COVID-19 vaccine rollout system to see if you have been vaccinated before. We may also be able to update the system with the vaccines you get in this study.

If you choose not to join this study, you may be able to join another study.

3. **If you want to join this study, we will screen you to see if you are eligible.**

We will give you a physical exam. We will give you a coronavirus and an HIV test. And we will ask you about your health history, including any medications you are taking.

If we find that you have HIV, we will refer you for treatment and care. We will check the level of HIV in your blood and the number of immune cells you have.

If you are a person who could become pregnant, we will do a urine pregnancy test. If we find that you are pregnant or you become pregnant during the study, we will do an ultrasound to measure the age and status of your developing pregnancy. We will also contact you after your due date to ask some questions about outcome of your pregnancy, delivery, and the health of your baby.

If you are pregnant, we will help you get advice on how to avoid getting HIV. If we find that you have HIV, we will refer you for treatment and care. We will check the level of HIV in your blood and the number of immune cells you have. We will

also ask if you now have or ever had syphilis and any treatment for it. This is for your safety and could also affect the timing of your injections.

Being in the study

If you want to join and you are eligible, here is what will happen:

4. **You will come to the clinic for scheduled visits about 7 or 8 times over 18 months depending on which group you are in.**

   Site: Insert number of visits and range of visit lengths. (There is site-specific variation in screening protocols and in the number of possible follow-up visits between protocol-mandated visits.)

   Visits can be done in-person or remotely depending on the type of procedures that need to be done. Visits where you get injections must be done in person. Remote visits will be done whenever possible to reduce potential SARS-CoV-2 exposure.

   Visits can last from [#] to [#] hours.

   You may have to come for more visits if you have a lab or health issue.

   We will also ask you for the name of someone who can tell us how you are doing if we can't reach you or if you are unable to talk. We may contact you after the main study ends (for example, to tell you about the study results).

5. **We will give you [Site: Insert compensation]for each study visit you complete.**

   This amount is to cover the costs of [Site: Insert text]

   Site: Insert any covered costs to participants.

   You do not have to pay anything to be in this study.

6. **We will give you the study products on a schedule.**

   You will be in 1 of 4 groups.

   All people in Group 1 and Group 3 will get 3 doses of the study vaccine. You will receive 1 injection into your upper arm at 3 separate visits during the study. The injections are given with a needle and syringe.

   All people in Group 2 and Group 4 will get 2 doses of the study vaccine. You will get 1 injection into your upper arm at 2 separate visits during the study. The injections are given with a needle and syringe.

At the visits where we give you the study products, we will need to make sure you do not have a bad reaction to it. For this reason, we will ask you to stay in the clinic for at least 15 minutes after your injection. You may have to wait longer if the rules in your local area require it.

Site: If your site has been selected for the reactogenicity assessment then include this paragraph below.

Then for that night and up to 7 more days, you will need to keep track of how you are feeling and if you have any symptoms. To help you do this, we can give you tools and show you how to use them. Contact the clinic staff if you have any issues or concerns after receiving an injection. If you have a problem, we will continue to check on you until it goes away.

7. **In addition to giving you the study products, we will do the procedures described and shown in the tables below:**

   a. Do physical exams

   b. Do swabs of your nose

   c. Ask questions about your health, including medications you may be taking

   d. We will ask you to keep track of any symptoms of COVID-19 or any other problems you might develop. We will give you a thermometer and ask you to take your temperature if you have symptoms. We will ask you to record any symptoms you are having on a paper form. We will contact you about every 2 weeks to ask about this. We will ask you to contact us if you develop symptoms or are hospitalized.

   e. Take blood samples.

When we take blood, the amount will depend on the lab tests we need to do. It will be some amount between 19 ½ mL and 114.5 mL (about 4 teaspoons to 7 ½ tablespoons). If you have HIV, some of this blood will be used to check the level of HIV and the number of immune cells you have. This is for your safety and could also affect the timing of your injections if you are started on HIV medications or have recently changed your HIV medications

We will be looking for side effects. We will also be looking for any symptoms of COVID-19. If any of the results are important to your health, we will tell you.

Procedures for Group 1 and Group 3

**Procedures**	Screening visit	First injection visit	1 month	2 months	6 months	7 months	12 months	18 months
Injection		√	√		√			
Physical exam	√							√
Medical history	√	√						
Interview /Questionnaire	√	√	√	√	√	√	√	√
Pregnancy test*	√							
Ultrasound*	√							
HIV test	√							
Blood drawn	√	√		√	√	√	√	√
Nasal swabs		√	√		√			

* May be performed for pregnant participants at screening and as needed during the study.

Procedures for Group 2 and Group 4

**Procedures**	Screening visit	First injection visit	1 month	6 months	7 months	12 months	18 months
Injection		√		√			
Physical exam	√						√
Medical history	√	√					
Interview/ Questionnaire	√	√	√	√	√	√	√
Pregnancy test*	√						
Ultrasound*	√						
HIV test	√						
Blood drawn	√	√	√	√	√	√	√
Nasal swabs		√		√			

* May be performed for pregnant participants at screening and as needed during the study.

8. **The CoVPN will test your samples to see how your body, including your immune system, responds to the study products.**

   We will send your samples to labs approved by the CoVPN. Your samples will not be labeled with your name or other identifying information. Researchers may also do genetic testing related to this study on your samples. Your genes are passed to you from your birth parents. Differences in people's genes can help explain why

some people get a disease while others do not. The genetic testing will only involve some of your genes, not all of your genes (your genome). The researchers will study only the genes related to the immune system and coronavirus, and genes that may affect how people get coronavirus.

If you get SARS-CoV-2, the researchers may look at all of the virus' genes that are in your samples. The researchers will use this information to learn more about SARS-CoV-2 and how the virus is impacted by the study vaccine.

In some cases, researchers may take cells from your samples and grow more of them over time, so that they can continue to contribute to this study.

The tests the researchers do on your samples are for research purposes, not to check your health. The labs will not give the results to you or this clinic because their tests are not approved for use in making health care decisions. These labs are only approved to do research tests.

When your samples are no longer needed for this study, the CoVPN will continue to store them.

9. **We will do our best to protect your private information.**

Your study records and samples will be kept in a secure location. Clinic staff will label all of your samples and most of your records with a code number, not your name or other personal information. We will not share your name with anyone who does not need to know it.

Your records may also be reviewed by groups who watch over this study. These groups include:

- The Sponsor and its study monitors
- The COVID-19 Prevention Network (CoVPN) and people who work for them,
- Some government agencies:
  - a) The US National Institutes of Health
  - b) The US Food and Drug Administration
  - c) The US Office for Human Research Protections
  - d) [Insert name of local and/or national regulatory authority as appropriate]
  - e) Any regulatory agency that reviews research studies
- Some committees that make sure we protect your rights and keep you safe:

a. The Data and Safety Monitoring Board

b. *Sites must include:* [Insert name of local IRB/EC]

All reviewers will take steps to keep your records private.

We cannot guarantee absolute privacy. If you are found to have a medical condition that we are required to report by law, then some of your information may be shared. At this clinic, we have to report the following information:

Site: Include any public health or legal reporting requirements. Bulleted examples should include all appropriate cases (reportable communicable disease, risk of harm to self or others, etc.)

- SARS-CoV-2
- Pneumonia
- [Item 3]
- [Item 4]

We will not share your name or information that can identify you with the CoVPN. The CoVPN may share information from this study with other researchers. Researchers may publish the results of this study.

Site: The text below may not be deleted or changed per FDA requirement.

A description of this clinical trial will be available on http://www.ClinicalTrials.gov, as required by US. Law. This website will not include information that can identify you. At most, the website will include a summary of the results. You can search this website at any time.

The study will also be listed in the Pan-African Clinical Trials registry.

10. **There are several reasons why we may stop your injections.**

We may stop your injections for a time if you get COVID-19. You can still take part in the other parts of the study. We will discuss your study options with you.

We will stop your injections if you enroll in a different study where you get another study product.

11. **We may take you out of the study at any time.**

We may take you out of the study if:

- you do not follow instructions,
- we think that staying in the study might harm you,
- the study is stopped for any reason.

12. **If you get COVID-19, we will monitor your health and help you get the care you need.**

If you have COVID-19 like symptoms or find out that you have COVID-19, you will be directed to contact the clinic, or we may visit you in your home. We will ask you to self-isolate per local guidelines.

We will not give you any study injections until you no longer have symptoms of COVID-19. We will continue your study injections when your symptoms go away, and the study staff thinks you are well enough.

Over a 4-week period, we will also:

- Contact you every other day to find out how you are feeling
- Ask you to keep track of your COVID-19 symptoms daily or until you test negative for COVID-19
- Do physical exams
- Ask questions about your health
- Ask you to measure the oxygen level in your blood daily. We will give you a small device to do this (pulse oximeter). The clinic will teach you about the device and when to contact the clinic if you develop low oxygen levels. If at any time you need additional care, we will help you get the care you need, including if you need to be hospitalized.
- Do swabs of your nose
- Take blood
- Do an HIV test
- At the end of the 4-week period, if you had a test that confirmed you had COVID-19, we will ask you to come to the clinic for a visit

When we take blood, the amount will depend on the lab tests we need to do. It will be some amount between 5 to 114.5 mL (about 1 teaspoon to 7 ½ tablespoons) each time. We will be looking for how your body reacts to infection and the study product. If any of the results are important to your health, we will tell you.

After the 4-week period, if you still have a test that confirms COVID-19, we will do swabs of your nose about every 2 weeks until you test negative for COVID-19.

Some people with COVID-19 develop severe symptoms and must be hospitalized. This can include being put on a ventilator to help you breathe. In the event you are not able to communicate and give consent for the study procedures to continue, we will pause your participation. It is unlikely that you will be hospitalized, but to prepare for this, we will ask you to sign and date a medical release of information so we can get records from your doctor or view your hospital records. When you are released from the hospital, please let us know. We will ask if you would like to continue participating in the study. We will only resume the study procedures if you give us permission to do so.

13. **If you plan to get another available COVID-19 vaccine outside the study, we will stop your in-study vaccinations but you may continue in the study.**

If you have plans to get another available COVID-19 vaccine outside the study, please let us know. We will ask you to tell us what vaccine you plan to get and when you will get it. You may continue to participate in follow-up visits. Your continued participation is important to help us meet our study goals.

Other Risks

14. **There are other risks to being in this study.**

This section describes the other risks and restrictions we know about. There may also be unknown risks, even serious ones. We will tell you if we learn anything new that may affect your willingness to stay in the study.

Risks of routine medical procedures:

In this study, we will do some routine medical procedures. These are taking blood and giving injections. These procedures can cause bruising, pain, fainting, soreness, redness, swelling, itching, a sore, bleeding, and (rarely) muscle damage or infection where you got the injection. Taking blood can cause a low blood cell count (anemia), making you feel tired.

Risks of swabs of your nose:

You may feel some discomfort from the swabs we put into your nose. Rarely, swabs can cause nosebleeds.

Risks to your personal information:

We will take several steps to protect your personal information. Although the risk is very low, it is possible that your personal information could be given to someone who should not have it. If that happened, you could have stress or anxiety.

Risks of genetic testing:

It is unlikely, but the genetic tests done on your samples could show you may be at risk for certain diseases. If others found out, it could lead to discrimination or other problems.

Risk of anxiety/emotional stress:

You may feel anxiety or emotional stress if you experience any of the risks described here. You may feel worried if tests show that you have SARS-CoV-2. Some of the questions we will ask you may make you feel uncomfortable.

Unknown risks:

The study vaccine has been shown to prevent adults from getting COVID-19 for at least 6 months after their injection, but we do not know if it will protect them for any longer. If you get COVID-19, we know that the study vaccine did not make the disease worse. It is also unknown if the study vaccine will prevent you from giving the virus to someone else if you become infected.

We do not know if getting this study vaccine will affect how you respond to any future SARS-CoV-2 vaccines.

Benefits

15. **This study may not benefit you.**

Although this study vaccine was shown to protect people from COVID-19 disease in other countries, we do not know how much the study vaccine will benefit you. However, being in the study might still help you in some ways. The lab tests and physical exams that you get while in this study might detect health problems you don't yet know about.

This study may help in the search for a vaccine to prevent SARS-CoV-2 that works for all people around the world.

If you decide not to join this study, you may be able to get another COVID-19 vaccine in your community. You should continue to follow local guidelines for SARS-CoV-2 infection.

Your rights and responsibilities

16. **If you join the study, you have rights and responsibilities.**

You have many rights that we will respect. You also have responsibilities. We list these in the Bill of Rights and Responsibilities for Research. We will give you a copy of it.

Leaving the study

17. **Tell us if you decide to leave the study.**

    You are free to leave the study at any time and for any reason. If you want to leave, you will need to tell us. Your care at this clinic and your legal rights will not be affected. We may ask you to come back to the clinic one last time, and we may ask to take some more samples. We believe this is important to protecting your health, but it is up to you whether you do this.

Injuries

Sites: Please do not make any changes to this section (other than those specified). If your institution requires changes, then you must consult CoVPN Regulatory Affairs (at CoVPN.core.reg@fredhutch.org).

18. **If you get sick or injured during the study, contact us immediately.**

    Your health is important to us. We will tell you about the care that we can give here. For the care that we cannot provide, we will explain how we will help you get care elsewhere.

    In this study, our clinic has insurance to cover your medical treatment in the case of a study-related injury. The Medical Research Council of South Africa have not provided for any payment if you are harmed as a result of taking part in this study. (*Sites outside of South Africa: remove the rest of the language in this paragraph. Sites in South Africa only: keep the rest of the language in this paragraph.*) We will follow the Association of the British Pharmaceutical Industry guidelines for payment of study-related injury. We can give you a copy of these guidelines.

    Some injuries are not physical. For example, you might be harmed emotionally by being in this study. Or you might lose wages because you cannot go to work. However, there are no funds to pay for these kinds of injuries, even if they are study related.

    You always have the right to use the court system if you are not satisfied.

    Site: Delete next section if using separate consent for use of samples and information in other studies

19. **When samples are no longer needed for this study, the CoVPN wants to use them in other studies and share them with other researchers.**

    The CoVPN calls these samples "extra samples". The CoVPN will only allow your extra samples to be used in other studies if you agree to this. You will mark your decision at the end of this form.

Do I have to agree? No. You are free to say yes or no, or to change your mind after you sign this form. At your request, the CoVPN will destroy all extra samples that it has. Your decision will not affect your being in this study.

Where are the samples stored? Extra samples are stored in a secure central place called a repository. Your samples will be stored in the CoVPN repository in South Africa.

How long will the samples be stored? There is no limit on how long your extra samples will be stored. [*Site: Revise the previous sentence to insert limits if your regulatory authority imposes them.]*

Will I be paid for the use of my samples? No. Researchers may make scientific discoveries or products using your samples. If this happens, there is no plan to share any money with you.

Will I benefit from allowing my samples to be used in other studies? Probably not. Results from these other studies are not given to you, this clinic, or your doctor. They may help other people in the future.

Will the CoVPN sell my samples and information? No, but the CoVPN may share your samples with other researchers. Once the CoVPN shares your samples and information, it may not be able to get them back.

How do other researchers get my samples and information? When a researcher wants to use your samples and information, their research plan must be approved by the CoVPN. Also, the researcher's institutional review board (IRB) or ethics committee (EC) will review their plan. *[Site: If review by your institution's IRB/EC/RE is also required, insert a sentence stating this.]* IRBs/ECs protect the rights and well-being of people in research. If the research plan is approved, the CoVPN will send your samples to the researcher's location.

What information is shared with researchers? The samples and information will be labeled with a code number which will not be removed. The key to the code will stay at this clinic. However, some information that the CoVPN shares may be personal, such as your race, ethnicity, sex, and health information from the study.

What kind of studies might be done with my extra samples and information? The studies will be related to vaccines, the immune system, coronavirus, HIV, and other diseases.

In some cases, researchers may take cells from your samples and grow more of them over time, so that they can continue to do research with them.

Researchers may also do genetic testing on your samples.

If you agree, researchers may compare all of your genes (your genome) to the genomes of many other people. Researchers look for common patterns of genes to help them understand diseases. The researchers may put the information into a protected database so that other researchers can access it. Your name and other personal information will not be included.

Usually, no one could connect your genome to you as a person. There are rules against this. It's also really difficult to do. But there is a risk that someone could combine information from your genome and other public information about you and identify you. If others found out, it could lead to discrimination or other problems. The risk of this happening is very small.

Who will have access to my information in studies using my extra samples?

People who may see your information are:

- Researchers who use your extra samples and information for other research
- Government agencies that fund or monitor the research using your extra samples and information
- The researcher's Institutional Review Board or Ethics Committee
- Any regulatory agency that reviews research studies
- The people who work with the researcher

All of these people will do their best to protect your information. If they publish their research, they will not use your name or identify you personally.

Questions

20. **If you have questions or problems at any time during your participation in this study, use the following important contacts.**

If you have questions about this study, contact
[name or title and telephone number of the investigator or other study staff].

If you have any symptoms that you think may be related to this study, contact
[name or title and telephone number of the investigator or other study staff].

This study has been reviewed and approved by a committee called the
[name of local IRB/EC]. If you have questions about your rights as a research participant, or problems or concerns about how you are being treated in this study, contact [name or title and telephone number of person on IRB/EC] , at the committee.

Paragraph for South African sites. The study has been structured in accordance with the Declaration of Helsinki (last updated October 2013) which deals with the recommendations guiding doctors in biomedical research involving human participants, the Ethics in Health Research: Principles, Structures and Processes Second Edition 2015, and Guidelines for Good Practice in the Conduct of Clinical Trials in Human Participants in South Africa. We can provide you with copies of these guidelines if you wish to review them. In addition, the recent Protection of Personal Information Act (POPIA) ensures that all South African institutions conduct themselves in a responsible manner when collecting, processing, storing and sharing another entity's personal information by holding them accountable should they abuse or compromise your personal information in any way.

If you want to leave this study, contact
[name or title and telephone number of the investigator or other study staff].

Remainder of section for South African sites only.

You can reach a study staff member 24 hours a day at [telephone number].

If you have questions about this trial you should first discuss them with your doctor or the ethics committee (contact details as provided on this form). After you have consulted your doctor or the ethics committee and if they have not provided you with answers to your satisfaction, you should write to the South African Healthcare Products Regulatory Authority (SAHPRA) at:

The Chief Executive Officer
Dr Boitumelo Semete-Makokotlela
South African Health Products Regulatory Authority Private Bag X828
PRETORIA
0001
Tel: (012) 501 0410/13
e-mail: Boitumelo.Semete@sahpra.org.za

Your permissions and signature

Site: Delete this section if using a separate consent for use of samples and information in other studies

21. **We told you above about possible other uses of your extra samples and information outside this study. Please choose only 1 of the options below and write your initials in the box next to it. Whatever you choose, the CoVPN keeps track of your decision about how your samples and information can be used. You can change your mind after signing this form.**

☐ I allow my extra samples and information to be used for other studies related to vaccines, the immune system, coronavirus, HIV, and other diseases. This may include genetic testing and keeping my cells growing over time.

OR

☐ I agree to the option above *and* also to allow my extra samples and information to be used in studies that look at my whole genome.

OR

☐ I do not allow my extra samples to be used in any other studies. This includes not allowing genetic testing, growing more of my cells, or studies that look at my whole genome.

22. **If you agree to join this study, you will need to sign below. Before you sign, make sure of the following:**

- You have read this consent form, or someone has read it to you.
- You feel that you understand what the study is about and what will happen to you if you join. You understand what the possible risks and benefits are.
- You have had your questions answered and know that you can ask more.
- You agree to join this study.

You will not be giving up any of your rights by signing this consent form.

Participant's name (print)	Participant's signature	Date	Time
Clinic staff conducting consent discussion (print)	Clinic staff signature	Date	Time

For participants who are unable to read or write, a witness should complete the signature block below:

Witness's name (print)	Witness's signature	Date	Time

*Witness is impartial and was present for the entire discussion of this consent form.

Appendix B Laboratory procedures table for Groups 1 and 3 (SARS-CoV-2 seronegative participants, main study)

Procedure	Ship to[1]	Assay Location[2]	Tube[4]	Tube size (vol. capacity)[4]	01	02	03	04	05	106	107	108	109	110	111
				Visit:	01	02	03	04	05	106	107	108	109	110	111
				Day:	-56 to 1	D1	D29	D57	D169	D197	D231	D265	D299	D333	D365
				Week:	*Screening visit*[3]	W0	W4	W8	W24	W28	W33	W38	W43	W48	W52
				Month:		M0	M1	M2	M6	M7	M8	M9	M10	M11	M12
						Vac 1	Vac 2		Vac 3						
BLOOD COLLECTION															
Screening/Diagnostic															
HIV test[5]	Local Lab	Local Lab	EDTA	5mL	5	—	—	—	—	—	—	—	—	—	—
HIV Viral Load[6]	Local Lab	Local Lab	EDTA	5mL	5[6]	5[6]	—	—	5[6]	—	—	—	—	—	—
CD4+ T Cell Count[6]	Local Lab	Local Lab	EDTA	5mL	5[6]	5[6]	—	—	5[6]	—	—	—	—	—	—
SARS-CoV-2 POC Serology	Local Lab	Local Lab	EDTA	5mL	5	—	—	—	—	—	—	—	—	—	—
SARS-CoV-2 Serology	BARC	HVTN Labs	SST	5mL	—	5	—	5	5	5	—	—	—	—	5
Immunogenicity assays															
Humoral assays	BARC	HVTN Labs	SST	8.5mL	—	8.5	—	8.5	8.5	8.5	—	—	—	—	8.5
Gene Expression	BARC	HVTN Labs	Tempus	3mL	—	6	—	6	6	6	—	—	—	—	6
**Visit total**					**20.0**	**29.5**	**0.0**	**19.5**	**29.5**	**19.5**	**0.0**	**0.0**	**0.0**	**0.0**	**19.5**
**56-Day total**					**20.0**	**49.5**	**49.5**	**69.0**	**29.5**	**49.0**	**19.5**	**19.5**	**0.0**	**0.0**	**19.5**
**NASAL SWAB SAMPLE COLLECTION**[4]															
SARS-CoV-2 PCR	Local Lab	Local Lab			—	X	X	—	X	X	X	X	X	X	X
SARS-CoV-2 Viral Sequencing[7]	BARC	HVTN Labs			—	X[7]	X[7]	—	X[7]	X[7]	X[7]	X[7]	X[7]	X[7]	X[7]
**URINE COLLECTION**															
Pregnancy Test[8]	Local Lab	Local Lab			X	—	—	—	—	—	—	—	—	—	—

[1] BARC = Bio Analytical Research Corporation South Africa (Pty) Ltd (Johannesburg, South Africa).

[2] HVTN Laboratories include: Bio Analytical Research Corporation South Africa (Pty) Ltd (Johannesburg, South Africa); Duke University Medical Center (Durham, North Carolina, USA); KwaZulu-Natal Research and Innovation Sequencing Platform (KRISP, Durban, South Africa).

[3] Screening may occur over the course of several contacts/visits up to and including Day 1 prior to study product administration.

[4] Local labs may assign appropriate alternative specimen type, tube and volume for locally performed tests.

[5] HIV diagnostic test will be performed for all participants at screening and as clinically indicated.

[6] HIV Viral Load and CD4+ T Cell Count will be performed at screening or at any visit for participants with newly diagnosed HIV infection, at enrollment for known PLWH, approximately 4-6 weeks after any change in antiretroviral therapy (other than just dosage) for PLWH, at visit 5 for PLWH, and as clinically indicated. (see Sections 9.2, 9.3, 9.4 and 9.9). HIV Viral Load and CD4+ T Cell Count do not need to be recollected if they were collected and the results available within the past 14 days.

[7] Nasal swab sample will only be sent to site processing lab for processing and storage if local testing results at the same visit return as SARS-CoV-2 infected.

[8] Pregnancy test will be performed at screening for all participants who were assigned female sex at birth and are of reproductive potential. A pregnancy test will be performed at other visits if the participant reports a new pregnancy and / or as clinically indicated.

Appendix C Laboratory procedures table for Groups 2 and 4 (SARS-CoV-2 seropositive participants, main study)

				Visit:	**01**	**02**	**03**	**04**	**05**	**106**	**107**	**108**	**109**	**110**	**111**
				Day:	-56 to 1	D1	D29	D57	D169	D197	D231	D265	D299	D333	D365
				Week:		W0	W4	W8	W24	W28	W33	W38	W43	W48	W52
				Month:	*Screening visit*[3]	M0	M1	M2	M6	M7	M8	M9	M10	M11	M12
**Procedure**	**Ship to**[1]	**Assay Location**[2]	**Tube**[4]	**Tube size (vol. capacity)**[4]		**Vac 1**			**Vac 2**						
BLOOD COLLECTION															
Screening/Diagnostic															
HIV test[5]	Local Lab	Local Lab	EDTA	5mL	5	—	—	—	—	—	—	—	—	—	—
HIV Viral Load[6]	Local Lab	Local Lab	EDTA	5mL	5[6]	5[6]	—	—	5[6]	—	—	—	—	—	—
CD4+ T Cell Count[6]	Local Lab	Local Lab	EDTA	5mL	5[6]	5[6]	—	—	5[6]	—	—	—	—	—	—
SARS-CoV-2 POC Serology	Local Lab	Local Lab	EDTA	5mL	5	—	—	—	—	—	—	—	—	—	—
SARS-CoV-2 Serology	BARC	HVTN Labs	SST	5mL	—	5	5	—	5	5	—	—	—	—	5
Immunogenicity assays															
Humoral assays	BARC	HVTN Labs	SST	8.5mL	—	8.5	8.5	—	8.5	8.5	—	—	—	—	8.5
Gene Expression	BARC	HVTN Labs	Tempus	3mL	—	6	6	—	6	6	—	—	—	—	6
**Visit total**					**20.0**	**29.5**	**19.5**	**0.0**	**29.5**	**19.5**	**0.0**	**0.0**	**0.0**	**0.0**	**19.5**
**56-Day total**					**20.0**	**49.5**	**69.0**		**29.5**	**49.0**	**19.5**	**19.5**	**0.0**	**0.0**	**19.5**
**NASAL SWAB SAMPLE COLLECTION**[4]															
SARS-CoV-2 PCR	Local Lab	Local Lab			—	X	—	—	X	X	X	X	X	X	X
SARS-CoV-2 Viral Sequencing[7]	BARC	HVTN Labs			—	X[7]	—	—	X[7]	X[7]	X[7]	X[7]	X[7]	X[7]	X[7]
**URINE COLLECTION**															
Pregnancy Test[8]	Local Lab	Local Lab			X	—	—	—	—	—	—	—	—	—	—

Greyed out visit (4) does not apply to this groups.

[1] BARC = Bio Analytical Research Corporation South Africa (Pty) Ltd (Johannesburg, South Africa)

[2] HVTN Laboratories include: Bio Analytical Research Corporation South Africa (Pty) Ltd (BARC) (Johannesburg, South Africa); Duke University Medical Center (Durham, North Carolina, USA); KwaZulu-Natal Research and Innovation Sequencing Platform (KRISP, Durban, South Africa)

[3] Screening may occur over the course of several contacts/visits up to and including Day 1 prior to study product administration.

[4] Local labs may assign appropriate alternative specimen type, tube and volume for locally performed tests.

[5] HIV diagnostic test will be performed for all participants at screening and as clinically indicated.

[6] HIV Viral Load and CD4+ T Cell Count will be performed at screening or at any visit for participants with newly diagnosed HIV infection, at enrollment for known PLWH, approximately 4-6 weeks after any change in antiretroviral therapy (other than just dosage) for PLWH, at visit 5 for PLWH, and as clinically indicated (see Sections 9.2, 9.3, 9.4 and 9.9). HIV Viral Load and CD4+ T Cell Count do not need to be recollected if they were collected and the results available within the past 14 days.

[7] Nasal swab sample will only be sent to site processing lab for processing and storage if local testing results at the same visit return as SARS-CoV-2 infected.

[8] Pregnancy test will be performed at screening for all participants who were assigned female sex at birth and are of reproductive potential. A pregnancy test will be performed at other visits if the participant reports a new pregnancy and / or as clinically indicated.

Appendix D Laboratory Procedures for PBMC Immunogenicity subset, Groups 1 and 3

				Visit:	01	02	03	04	05	106	107	108	109	110	111
				Day:	-56 to 1	D1	D29	D57	D169	D197	D231	D265	D299	D333	D365
				Week:		W0	W4	W8	W24	W28	W33	W38	W43	W48	W52
				Month:	*Screeining visit[3]*	M0	M1	M2	M6	M7	M8	M9	M10	M11	M12
**Procedure**	**Ship to[1]**	**Assay Location[2]**	**Tube[4]**	**Tube size (vol. capacity)[4]**		**Vac 1**	**Vac 2**		**Vac 3**						
BLOOD COLLECTION															
Screening/Diagnostic															
HIV test[5]	Local Lab	Local Lab	EDTA	5mL	5	—	—	—	—	—	—	—	—	—	—
HIV Viral Load[6]	Local Lab	Local Lab	EDTA	5mL	5[6]	5[6]	—	—	5[6]	—	—	—	—	—	—
CD4+ T Cell Count[6]	Local Lab	Local Lab	EDTA	5mL	5[6]	5[6]	—	—	5[6]	—	—	—	—	—	—
SARS-CoV-2 POC Serology	Local Lab	Local Lab	EDTA	5mL	5	—	—	—	—	—	—	—	—	—	—
SARS-CoV-2 Serology	BARC	HVTN Labs	SST	5mL	—	5	—	5	5	—	—	—	—	—	5
Immunogenicity assays															
Cellular assays[9]	BARC	HVTN Labs	ACD	8.5mL	—	85	—	85	85	85	—	—	—	—	85
Humoral assays	BARC	HVTN Labs	SST	8.5mL	—	8.5	—	8.5	8.5	8.5	—	—	—	—	8.5
Gene Expression	BARC	HVTN Labs	Tempus	3mL	—	6	—	6	6	6	—	—	—	—	6
**Visit total**					**20.0**	**114.5**	**0.0**	**104.5**	**114.5**	**99.5**	**0.0**	**0.0**	**0.0**	**0.0**	**104.5**
**56-Day total**					**20.0**	**134.5**	**134.5**	**239.0**	**114.5**	**99.5**	**99.5**	**99.5**	**0.0**	**0.0**	**104.5**
**NASAL SWAB SAMPLE COLLECTION[4]**															
SARS-CoV-2 PCR	Local Lab	Local Lab			—	X	X	—	X	X	X	X	X	X	X
SARS-CoV-2 Viral Sequencing[7]	BARC	HVTN Labs			—	X[7]	X[7]	—	X[7]	X[7]	X[7]	X[7]	X[7]	X[7]	X[7]
**URINE COLLECTION**															
Pregnancy Test[8]	Local Lab	Local Lab			X	—	—	—	—	—	—	—	—	—	—

[1]BARC = Bio Analytical Research Corporation South Africa (Pty) Ltd (Johannesburg, South Africa)

[2]HVTN Laboratories include: Bio Analytical Research Corporation South Africa (Pty) Ltd (BARC) (Johannesburg, South Africa); Fred Hutchinson Cancer Center (Seattle, Washington, USA); Duke University Medical Center (Durham, North Carolina, USA); Cape Town Immunology Laboratory (CHIL, Cape Town, South Africa); KwaZulu-Natal Research and Innovation Sequencing Platform (KRISP, Durban, South Africa).

[3]Screening may occur over the course of several contacts/visits up to and including Day 1 prior to study product administration.

[4]Local labs may assign appropriate alternative specimen type, tube and volume for locally performed tests.

[5]HIV diagnostic test will be performed for all participants at screening and as clinically indicated.

[6] HIV Viral Load and CD4+ T Cell Count will be performed at screening or at any visit for participants with newly diagnosed HIV infection, at enrollment for known PLWH, approximately 4-6 weeks after any change in antiretroviral therapy (other than just dosage) for PLWH, at visit 5 for PLWH, and as clinically indicated (see Sections 9.2, 9.3, 9.4 and 9.9). HIV Viral Load and CD4+ T Cell Count do not need to be recollected if they were collected and the results available within the past 14 days.

[7] Nasal swab sample will only be sent to site processing lab for processing and storage if local testing results at the same visit return as SARS-CoV-2 infected.

[8] Pregnancy test will be performed at screening for all participants who were assigned female sex at birth and are of reproductive potential. A pregnancy test will be performed at other visits if the participant reports a new pregnancy and / or as clinically indicated.

[9]8x10mL NaHep tubes may be used if ACD tubes are not available

Appendix E Laboratory Procedures for PBMC Immunogenicity subset, Groups 2 and 4

				Visit:	01	02	03	04	05	106	107	108	109	110	111
				Day:	-56 to 1	D1	D29	D57	D169	D197	D231	D265	D299	D333	D365
				Week:		W0	W4	W8	W24	W28	W33	W38	W43	W48	W52
				Month:	Screening visit[3]	M0	M1	M2	M6	M7	M8	M9	M10	M11	M12
**Procedure**	**Ship to[1]**	**Assay Location[2]**	**Tube[4]**	**Tube size (vol. capacity)[4]**		**Vac 1**			**Vac 2**						
BLOOD COLLECTION															
Screening/Diagnostic															
HIV test[5]	Local Lab	Local Lab	EDTA	5mL	5	—	—	—	—	—	—	—	—	—	—
HIV Viral Load[6]	Local Lab	Local Lab	EDTA	5mL	5[6]	5[6]	—	—	5[6]	—	—	—	—	—	—
CD4+ T Cell Count[6]	Local Lab	Local Lab	EDTA	5mL	5[6]	5[6]	—	—	5[6]	—	—	—	—	—	—
SARS-CoV-2 POC Serology	Local Lab	Local Lab	EDTA	5mL	5	—	—	—	—	—	—	—	—	—	—
SARS-CoV-2 Serology	BARC	HVTN Labs	SST	5mL	—	5	5	—	5	—	—	—	—	—	5
Immunogenicity assays															
Cellular assays[9]	BARC	HVTN Labs	ACD	8.5mL	—	85	85	—	85	85	—	—	—	—	85
Humoral assays	BARC	HVTN Labs	SST	8.5mL	—	8.5	8.5	—	8.5	8.5	—	—	—	—	8.5
Gene Expression	BARC	HVTN Labs	Tempus	3mL	—	6	6	—	6	6	—	—	—	—	6
**Visit total**					**20.0**	**114.5**	**104.5**	**0.0**	**114.5**	**99.5**	**0.0**	**0.0**	**0.0**	**0.0**	**104.5**
**56-Day total**					**20.0**	**134.5**	**239.0**		**114.5**	**214.0**	**214.0**	**99.5**	**0.0**	**0.0**	**104.5**
**NASAL SWAB SAMPLE COLLECTION[4]**															
SARS-CoV-2 PCR	Local Lab	Local Lab			—	X	—	—	X	X	X	X	X	X	X
SARS-CoV-2 Viral Sequencing[7]	BARC	HVTN Labs			—	X[7]	—	—	X[7]	X[7]	X[7]	X[7]	X[7]	X[7]	X[7]
**URINE COLLECTION**															
Pregnancy Test[8]	Local Lab	Local Lab			X	—	—	—	—	—	—	—	—	—	—

Greyed out Visit 4 does not apply to this group.

[1]BARC = Bio Analytical Research Corporation South Africa (Pty) Ltd (Johannesburg, South Africa)

[2]HVTN Laboratories include: Bio Analytical Research Corporation South Africa (Pty) Ltd (BARC) (Johannesburg, South Africa); Fred Hutchinson Cancer Center (Seattle, Washington, USA); Duke University Medical Center (Durham, North Carolina, USA); Cape Town Immunology Laboratory (CHIL, Cape Town, South Africa); KwaZulu-Natal Research and Innovation Sequencing Platform (KRISP, Durban, South Africa)

[3]Screening may occur over the course of several contacts/visits up to and including Day 1 prior to study product administration.

[4]Local labs may assign appropriate alternative specimen type, tube and volume for locally performed tests.

[5]HIV diagnostic test will be performed for all participants at screening and as clinically indicated.

[6] HIV Viral Load and CD4+ T Cell Count will be performed at screening or at any visit for participants with newly diagnosed HIV infection, at enrollment for known PLWH, approximately 4-6 weeks after any change in antiretroviral therapy (other than just dosage) for PLWH, at visit 5 for PLWH, and as clinically indicated (see Sections 9.2, 9.3,9.4 and 9.9). HIV Viral Load and CD4+ T Cell Count do not need to be recollected if they were collected and the results available within the past 14 days.

[7] Nasal swab sample will only be sent to site processing lab for processing and storage if local testing results at the same visit return as SARS-CoV-2 infected.

[8] Pregnancy test will be performed at screening for all participants who were assigned female sex at birth and are of reproductive potential. A pregnancy test will be performed at other visits if the participant reports a new pregnancy and / or as clinically indicated.

[9]8x10mL NaHep tubes may be used if ACD tubes are not available

Appendix F Laboratory Procedures for the PBMC Immune Correlates subset (Group 1, HIV positive, SARS-CoV-2 seronegative participants)

				Visit:	**01**	**02**	**03**	**04**	**05**	**106**	**107**	**108**	**109**	**110**	**111**
				Day:	-56 to 1	D1	D29	D57	D169	D197	D231	D265	D299	D333	D365
				Week:		W0	W4	W8	W24	W28	W33	W38	W43	W48	W52
				Month:	*Screening visit*[3]	M0	M1	M2	M6	M7	M8	M9	M10	M11	M12
**Procedure**	**Ship to**[1]	**Assay Location**[2]	**Tube**[4]	**Tube size (vol. capacity)**[4]		**Vac 1**	**Vac 2**		**Vac 3**						
BLOOD COLLECTION															
Screening/Diagnostic															
HIV test[5]	Local Lab	Local Lab	EDTA	5mL	5	—	—	—	—	—	—	—	—	—	—
HIV Viral Load[6]	Local Lab	Local Lab	EDTA	5mL	5[6]	5[6]	—	—	5[6]	—	—	—	—	—	—
CD4+ T Cell Count[6]	Local Lab	Local Lab	EDTA	5mL	5[6]	5[6]	—	—	5[6]	—	—	—	—	—	—
SARS-CoV-2 POC Serology	Local Lab	Local Lab	EDTA	5mL	5	—	—	—	—	—	—	—	—	—	—
SARS-CoV-2 Serology	BARC	HVTN Labs	SST	5mL	—	5	—	5	5	—	—	—	—	—	5
Immunogenicity assays															
Cellular assays[9]	BARC	HVTN Labs	ACD	8.5mL	—	85	—	85	—	85	—	—	—	—	—
Humoral assays	BARC	HVTN Labs	SST	8.5mL	—	8.5	—	8.5	8.5	8.5	—	—	—	—	8.5
Gene Expression	BARC	HVTN Labs	Tempu	3mL	—	6	—	6	6	6	—	—	—	—	6
**Visit total**					**20.0**	**114.5**	**0.0**	**104.5**	**29.5**	**99.5**	**0.0**	**0.0**	**0.0**	**0.0**	**19.5**
**56-Day total**					**20.0**	**134.5**	**134.5**	**239.0**	**29.5**	**99.5**	**99.5**	**99.5**	**0.0**	**0.0**	**19.5**
**NASAL SWAB SAMPLE COLLECTION**[4]															
SARS-CoV-2 PCR	Local Lab	Local Lab			—	X	X	—	X	X	X	X	X	X	X
SARS-CoV-2 Viral Sequencing[7]	BARC	HVTN Labs			—	X[7]	X[7]	—	X[7]	X[7]	X[7]	X[7]	X[7]	X[7]	X[7]
**URINE COLLECTION**															
Pregnancy Test[8]	Local Lab	Local Lab			X	—	—	—	—	—	—	—	—	—	—

[1]BARC = Bio Analytical Research Corporation South Africa (Pty) Ltd (Johannesburg, South Africa)

[2]HVTN Laboratories include: Bio Analytical Research Corporation South Africa (Pty) Ltd (BARC) (Johannesburg, South Africa); Fred Hutchinson Cancer Center (Seattle, Washington, USA); Duke University Medical Center (Durham, North Carolina, USA); Cape Town Immunology Laboratory (CHIL, Cape Town, South Africa); KwaZulu-Natal Research and Innovation Sequencing Platform (KRISP, Durban, South Africa)

[3]Screening may occur over the course of several contacts/visits up to and including Day 1 prior to study product administration.

[4]Local labs may assign appropriate alternative specimen type, tube and volume for locally performed tests.

[5]HIV diagnostic test will be performed for all participants at screening and as clinically indicated.

[6] HIV Viral Load and CD4+ T Cell Count will be performed screening or at any visit for participants with newly diagnosed HIV infection, at enrollment for known PLWH, approximately 4-6 weeks after any change in antiretroviral therapy (other than just dosage) for PLWH, at visit 5 for PLWH, and as clinically indicated (see Sections 9.2, 9.3, 9.4 and 9.9). HIV Viral Load and CD4+ T Cell Count do not need to be recollected if they were collected and the results available within the past 14 days.

[7] Nasal swab sample will only be sent to site processing lab for processing and storage if local testing results at the same visit return as SARS-CoV-2 infected.

[8] Pregnancy test will be performed at screening for all participants who were assigned female sex at birth and are of reproductive potential. A pregnancy test will be performed at other visits if the participant reports a new pregnancy and / or as clinically indicated.

[9]8x10mL NaHep tubes may be used if ACD tubes are not available

Appendix G Laboratory Procedures for the PBMC Immune Correlates subset (Group 2, HIV positive, SARS-CoV-2 seropositive participants)

Procedure	Ship to[1]	Assay Location[2]	Tube[4]	Tube size (vol. capacity)[4]	01	02	03	04	05	106	107	108	109	110	111
				Visit:	01	02	03	04	05	106	107	108	109	110	111
				Day:	-56 to 1	D1	D29	D57	D169	D197	D231	D265	D299	D333	D365
				Week:		W0	W4	W8	W24	W28	W33	W38	W43	W48	W52
				Month:	*Screening visit*[3]	M0	M1	M2	M6	M7	M8	M9	M10	M11	M12
						**Vac 1**			**Vac 2**						
BLOOD COLLECTION															
Screening/Diagnostic															
HIV test[5]	Local Lab	Local Lab	EDTA	5mL	5	—	—	—	—	—	—	—	—	—	—
HIV Viral Load[6]	Local Lab	Local Lab	EDTA	5mL	5[6]	5[6]	—	—	5[6]	—	—	—	—	—	—
CD4+ T Cell Count[6]	Local Lab	Local Lab	EDTA	5mL	5[6]	5[6]	—	—	5[6]	—	—	—	—	—	—
SARS-CoV-2 POC Serology	Local Lab	Local Lab	EDTA	5mL	5	—	—	—	—	—	—	—	—	—	—
SARS-CoV-2 Serology	BARC	HVTN Labs	SST	5mL	—	5	5	—	5	—	—	—	—	—	5
Immunogenicity assays															
Cellular assays[9]	BARC	HVTN Labs	ACD	8.5mL	—	85	85	—	—	85	—	—	—	—	—
Humoral assays	BARC	HVTN Labs	SST	8.5mL	—	8.5	8.5	—	8.5	8.5	—	—	—	—	8.5
Gene Expression	BARC	HVTN Labs	Tempus	3mL	—	6	6	—	6	6	—	—	—	—	6
**Visit total**					**20.0**	**114.5**	**104.5**	**0.0**	**29.5**	**99.5**	**0.0**	**0.0**	**0.0**	**0.0**	**19.5**
**56-Day total**					**20.0**	**134.5**	**239.0**		**29.5**	**99.5**	**99.5**	**99.5**	**0.0**	**0.0**	**19.5**
**NASAL SWAB SAMPLE COLLECTION**[4]															
SARS-CoV-2 PCR	Local Lab	Local Lab			—	X	—	—	X	X	X	X	X	X	X
SARS-CoV-2 Viral Sequencing[7]	BARC	HVTN Labs			—	X[7]	—	—	X[7]	X[7]	X[7]	X[7]	X[7]	X[7]	X[7]
**URINE COLLECTION**											—	—	—	—	—
Pregnancy Test[8]	Local Lab	Local Lab			X	—	—	—	—	—	—	—	—	—	—

Greyed out Visit 4 does not apply to this group.

[1]BARC = Bio Analytical Research Corporation South Africa (Pty) Ltd (Johannesburg, South Africa)

[2]HVTN Laboratories include: Bio Analytical Research Corporation South Africa (Pty) Ltd (BARC) (Johannesburg, South Africa); Duke University Medical Center (Durham, North Carolina, USA); KwaZulu-Natal Research and Innovation Sequencing Platform (KRISP, Durban, South Africa)

[3]Screening may occur over the course of several contacts/visits up to and including day 1 prior to study product administration.

[4]Local labs may assign appropriate alternative specimen type, tube and volume for locally performed tests.

[5] HIV diagnostic test will be performed for all participants at screening and as clinically indicated.

[6] HIV Viral Load and CD4+ T Cell Count will be performed at screening or any visit for participants with newly diagnosed HIV infection, at enrollment for known PLWH, approximately 4-6 weeks after any change in antiretroviral therapy (other than just dosage) for PLWH, at visit 5 for PLWH, and as clinically indicated (see Sections 9.2, 9.3, 9.4 and 9.9). HIV Viral Load and CD4+ T Cell Count do not need to be recollected if they were collected and the results available within the past 14 days.

[7] Nasal swab sample will be only be sent to site processing lab for processing and storage if local testing results at the same visit return as SARS-CoV-2 infected.

[8] Pregnancy test will be performed at screening for all participants who were assigned female sex at birth and are of reproductive potential. A pregnancy test will be performed at other visits if the participant reports a new pregnancy and / or as clinically indicated.

[9]8x10mL NaHep tubes may be used if ACD tubes are not available

Appendix H Schedule of clinic procedures for Groups 1 and 3 (SARS-CoV-2 seronegative participants)

Visit Number	01	02	03	04	05	106	107	108	109	110	111
Study Week		0	4	8	24	28	33	38	43	48	52
Study Month		0	1	2	6	7	8	9	10	11	12
Study Day	-56 to 1	1	29	57	169	197	231	265	299	333	365
Procedure	Screen	Vac 1	Vac 2	Follow up	Vac 3	Follow up					
**Study procedures**											
Assessment of Understanding	√										
Informed consent	√										
Medical history[1]	√										
Physical exam[2]	√										√
Obtain demographics	√										
Risk assessment questionnaire		√									
Targeted concomitant medications[3]	√	√	√	√	√	√	√	√	√	√	√
Vaccination[4]		√			√						
Ultrasound for gestational age[5]	√										
COVID-19 surveillance[6]		√	√	√	√	√	√	√	√	√	√
SAEs, AESIs[7]		√	√	√	√	√	√	√	√	√	√
**Safety Subset participants** [7]											
Reactogenicity assessment		√	√		√						
AEs for 28 days postvaccination		√	√		√						
**Specimen Collections**											
Pregnancy test[8]	√										
HIV diagnostic test[9]	√										
Blood draw[10]	√[11]	√		√	√	√					√
Nasal swab[12]		√	√		√	√	√	√	√	√	√

[1] Medical history: Targeted medical history is performed during screening. At enrollment and at subsequent visits, an interim medical history may be performed.

[2] Abbreviated physical exam will be performed at screening and at the last visit. At other clinic visits a physical exam will be performed as needed, based on participant self-reported symptoms or indications of illness.

[3] Targeted concomitant medication will be assessed for high-risk conditions.

[4] Vaccination (in clinic assessments): At least 15 minutes after each vaccination and prior to clinic discharge, the injection site will be assessed, and systemic symptoms will be assessed (see Section 9.3 for detail).

[5] Ultrasound to evaluate gestational age and for presence of fetal anomalies will be performed for all participants confirmed to be pregnant at enrollment or any other visit, and as clinically indicated, if the participant intends to continue with the pregnancy.

[6] COVID-19 surveillance: prespecified symptoms that meet the criteria for suspicion of COVID-19 will be elicited approximately every 2 weeks from the participant (see Section 9.5).

[7] Solicited and unsolicited AEs collected for a subset of study participants. Serious adverse events and adverse events of special interest (see Appendix M) collected for all participants throughout the study.

[8] Pregnancy test will be performed at screening for all participants who were assigned female sex at birth and are of reproductive potential. A pregnancy test will be performed at other visits if the participant reports a new pregnancy and / or as clinically indicated.

[9] HIV diagnostic test will be performed for all participants at screening and as clinically indicated. Participants with a positive HIV status will be offered HIV counseling. They will also be referred for care and treatment according to local guidelines.

[10] For details on blood draws see Appendix B, Appendix D and Appendix F.

[11] Blood sample will be drawn to determine SARS-CoV-2 serostatus (see Section 9.2).

[12] Two nasal swabs will be collected at each indicated visit.

Appendix I Schedule of clinic procedures for Groups 2 and 4 (SARS-CoV-2 seropositive participants)

Visit Number	01	02	03	04	05	106	107	108	109	110	111
Study Week		0	4	8	24	28	33	38	43	48	52
Study Month		0	1	2	6	7	8	9	10	11	12
Study Day	-56 to 1	1	29	57	169	197	231	265	299	333	365
Procedure	Screen	Vac 1			Vac 2	Follow up					
**Study procedures**											
Assessment of Understanding	√										
Informed consent	√										
Medical history[1]	√										
Physical exam[2]	√										√
Obtain demographics	√										
Risk assessment questionnaire		√									
Targeted concomitant medications[3]	√	√	√		√	√	√	√	√	√	√
Vaccination[4]		√			√						
Ultrasound for gestational age[5]	√										
COVID-19 surveillance[6]		√	√		√	√	√	√	√	√	√
SAEs, AESIs[7]		√	√		√	√	√	√	√	√	√
**Safety Subset participants**[7]											
Reactogenicity assessment		√			√						
AEs for 28 days postvaccination		√			√						
**Specimen Collections**											
Pregnancy test[8]	√										
HIV diagnostic test[9]	√										
Blood draw[10]	√[11]	√	√		√	√					√
Nasal swab[12]		√			√	√	√	√	√	√	√

Grayed out Visit 4 does not apply for this group.

[1] Medical history: Targeted medical history is performed during screening. At enrollment and at subsequent visits, an interim medical history may be performed.

[2] Abbreviated physical exam will be performed at screening and at the last visit. At other clinic visits a physical exam will be performed as needed, based on participant self-reported symptoms or indications of illness.

[3] Targeted concomitant medication will be assessed for high-risk conditions.

[4] Vaccination (in clinic assessments): At least 15 minutes after each vaccination and prior to clinic discharge, the injection site will be assessed, and systemic symptoms will be assessed (see Section 9.3 for detail).

[5] Ultrasound to evaluate gestational age and for presence of fetal anomalies will be performed for all participants confirmed to be pregnant at enrollment or any other visit, and as clinically indicated, if the participant intends to continue with the pregnancy.

[6] COVID-19 surveillance: prespecified symptoms that meet the criteria for suspicion of COVID-19 will be elicited approximately every 2 weeks from the participant (see Section 9.5).

[7] Solicited and unsolicited AEs collected for a subset of study participants. Serious adverse events and adverse events of special interest (see Appendix M) collected for all participants throughout the study.

[8] Pregnancy test will be performed at screening for all participants who were assigned female sex at birth and are of reproductive potential. A pregnancy test will be performed at other visits if the participant reports a new pregnancy and / or as clinically indicated.

[9] HIV diagnostic test will be performed for all participants at screening and as clinically indicated. Participants with a positive HIV status will be offered HIV counseling. They will also be referred for care and treatment according to local guidelines.

[10] For details on blood draws see Appendix C, Appendix E and Appendix G.

[11] Blood sample will be drawn to confirm SARS-CoV-2 serostatus (see Section 9.2).

[12] Two nasal swabs will be collected at each indicated visit.

Appendix J COVID-19 symptom visits

COVID-19 Symptom Visits: Clinic Procedures

**Procedure[5] [visit allowance]**	**Day 1 (clinic visit)**	**Day 3 (blood collection) [-2, + 2]**	**Day 2-14 (phone call)**	**Day 14 (swab collection) [-3, + 3]**	**Day 28 (clinic visit) [-3, +3]**	**Day 42+ (swab collection if indicated[7,8]) [-3, +3]**	**Day 56+ (swab and blood collection, if indicated)**
History & Physical Exam	X[1]				X		X
Symptom and SpO2 monitoring by participant[2]	Daily through 14 days or until a negative SARS-CoV-2 NAAT test is obtained, whichever is longer						
Symptom monitoring by CRS[3]	Every other day through 14 days or until symptoms improve (if symptomatic), whichever is longer						
**Sample collections[6]**							
Nasal swabs (two)[4]	X			X	X[7]	X[7]	X[7]
Blood collection	X	X			X		X[8]

[1] Participant history and physical exam on Day 1.

[2] On Day 1 participant will receive the Memory Aid 2 and will be instructed to record symptoms (if any) and their maximum daily severity if determined to have a SARS-CoV-2 positive NAAT test. Participant will also receive pulse oximeter and will be instructed on how to use it at rest and after mild exercise and how to record these O2 saturations in the Memory Aid 2. Participants will be instructed to record daily symptoms (if any) and O2 saturations through Day 14 or until a negative SARS-CoV-2 NAAT test result, whichever is longer. They will be instructed to contact the clinic if symptoms worsen. (See Section 9.6.) Recorded symptoms and their severity will be entered into the appropriate CRF by CRS staff.

[3] Participants will be contacted by the CRS staff every other day, at a minimum, by phone (or alternative contact method) through Day 14 or until symptom improvement (if symptomatic), whichever is longer.

[4] Two nasal swabs will be collected at indicated timepoints (1 swab will be sent for local SARS-CoV-2 NAAT testing to confirm diagnosis and the other one will be shipped to the central laboratory). Local tests can be repeated at the investigator's discretion. Symptom history will be collected at this visit and consolidated information will be entered into the appropriate CRF by CRS staff.

[5] Day 1 and Day 3 procedures can be combined in a single visit if participants have been diagnosed with SARS-CoV-2 and all required COVID-19 Visit procedures for Day 1 and Day 3 can be done in 1 visit.

[6] Specimens previously collected during a routine visit do not need to be recollected if it was within the visit window. HIV Viral Load and CD4+ T Cell Count do not need to be recollected if they were collected and the results available within the past 14 days. See CoVPN 3008 (Ubuntu) SSP for further guidance.

[7] If nasal swab at day 14 is positive, nasal swabs will be collected approximately every 2 weeks until a negative result is received (1 swab will be sent for local SARS-CoV-2 NAAT testing to confirm diagnosis and the other one will be shipped to the central laboratory). Local tests can be repeated at the investigator's discretion.

[8] If nasal swab at day 42 is positive, blood samples will be collected on day 56 and every 8 weeks thereafter until a negative swab result is received.

COVID-19 Symptom Visits: Laboratory Procedures Table

				Visit:	**#.X[4]**	**#.Y[5]**	**#.S[5]**	**#.Z[5]**	**#.S[5,10]**	**#.S[5,10]**
				Days relative to Symptom Visit:	D1	D3	D14	D28	D42	D56+[12]
**Procedure**	**Ship to[1]**	**Assay Location[2]**	**Tube[3]**	**Tube size (vol. capacity)[3]**	**Symptom visit**					
BLOOD COLLECTION										
HIV test	Local Lab	Local Lab	EDTA	5mL	5[6]	—	—	—	—	—
HIV Viral Load	Local Lab	Local Lab	EDTA	5mL	—	5[7]	—	—	—	5[7]
CD4+ T Cell Count	Local Lab	Local Lab	EDTA	5mL	—	5[7]				5[7]
SARS-CoV-2 Serology	BARC	HVTN Labs	SST	5mL	—	5		5		
Immunogenicity Assays[9]										
Cellular Assays[11]	BARC	HVTN Labs	ACD[8]	8.5mL	—	85	—	85	—	85
Humoral Assays	BARC	HVTN Labs	SST	8.5mL	—	8.5	—	8.5	—	8.5
Gene Expression	BARC	HVTN Labs	Tempus	3mL	—	6	—	6	—	—
**Visit total**					**5.0**	**114.5**	**0.0**	**104.5**	**0.0**	**103.5**
**56-Day total**					**5.0**	**119.5**	**119.5**	**224.0**	**224.0**	**327.5**
**NASAL SWAB SAMPLE COLLECTION[3]**										
SARS-CoV-2 NAAT	Local Lab	Local Lab			X	—	X	X[10]	X[10]	X[10]
SARS-CoV-2 Viral Sequencing	BARC	HVTN Labs			X	—	X	X[10]	X[10]	X[10]

[1] BARC = Bio Analytical Research Corporation South Africa (Pty) Ltd (Johannesburg, South Africa);
[2] HVTN Laboratories include: Bio Analytical Research Corporation South Africa (Pty) Ltd (BARC) (Johannesburg, South Africa); Fred Hutchinson Cancer Center (Seattle, Washington, USA); Duke University Medical Center (Durham, North Carolina, USA); Cape Town Immunology Laboratory (CHIL, Cape Town, South Africa); KwaZulu-Natal Research and Innovation Sequencing Platform (KRISP, Durban, South Africa).
[3] Local labs may assign appropriate alternative specimen type, tube and volume for locally performed tests.
[4] Visit #.X indicates a Symptom visit for participants where they were confirmed SARS-CoV-2 positive by NAAT, which can be an interim or scheduled visit. See protocol Section 9.5 and 9.6.
[5] Visit #.Y, #.S and #.Z indicates a Symptom follow-up visit for participants with confirmed SARS-CoV-2 infection which can be an interim or scheduled visit. See protocol Section 9.6.
[6] HIV test will not be performed for known PLWH.
[7] HIV Viral Load and CD4+ T Cell Count do not need to be recollected for PLWH if they were collected and the results available within the past 14 days.
[8] CPT tubes may be collected at predetermined sites.
[9] Samples for immunogenicity and gene expression do not need to be recollected if they have already been collected within the visit window.
[10] If nasal swab at day 14 is positive, nasal swabs will be collected approximately every two weeks until a negative result is received.
[11] 8x10mL NaHep tubes may be collected if ACD tubes are not available
[12] If nasal swab on day 42 is positive, blood samples will be collected on day 56 and approximately every 8 weeks thereafter until a negative swab result is received.

Appendix K Visit windows

Groups 1 and 3

Visit Number	Visit Type	Lower Allowable Window	Lower Target Window	Target Day[1]	Upper Target Window	Upper Allowable Window
01	Screening	-56	-		-	0
02	**Vaccination 1**	-	-0	1	+0	0
03	**Vaccination 2**		-3	29	+7	+14
04	Follow-up[2]		-3	57	+7	+14
05	**Vaccination 3**	-28	-7	169	+14	+28
106	Follow-up[2]		-7	197	+7	+16
107	Follow-up[2]	-17	-7	231	+7	+16
108	Follow-up[2]	-17	-7	265	+7	+16
109	Follow-up[2]	-17	-7	299	+7	+16
110	Follow-up[2]	-17	-7	333	+7	+16
111	Follow-up[2]	-15	-7	365	+7	+16

1. Target dates are relative to Visit 2 (Enrollment/Randomization), except for cases described in footnote 2.
2. Target dates are relative to the vaccination visit before.

Groups 2 and 4

Visit Number	Visit Type	Lower Allowable Window	Lower Target Window	Target Day[1]	Upper Target Window	Upper Allowable Window
01	Screening	-56	-		-	0
02	**Vaccination 1**	-	-0	1	+0	0
03	Follow-up		-3	29	+7	+14
04[3]						
05	**Vaccination 2**	-28	-7	169	+14	+28
106	Follow-up[2]		-7	197	+7	+16
107	Follow-up	-17	-7	231	+7	+16
108	Follow-up	-17	-7	265	+7	+16
109	Follow-up	-17	-7	299	+7	+16
110	Follow-up	-17	-7	333	+7	+16
111	Follow-up	-15	-7	365	+7	+16

1. Target dates are relative to Visit 2 (Enrollment/Randomization), except for cases described in footnote 2.
2. Target dates are relative to the vaccination visit before.
3. Grayed out Visit 4 does not apply for these groups.

Appendix L Addendum to the sample informed consent form

Title: Multi-Center, Randomized, Efficacy Study of COVID-19 mRNA Vaccine in Regions with SARS-CoV-2 Variants of Concern<br>Study number: CoVPN 3008 (Ubuntu)<br>Site: [Insert site name]

Key information

- Enrollment in this study is now complete with over 14,000 participants. About half of the participants have completed their injections visits. Injection visits are ongoing for the rest.

- We are adding Moderna's updated bivalent vaccine to this study. Bivalent means it contains components of the original strain of SARS-CoV-2 and the Omicron subvariants BA.4 and BA.5.

- Participants will need to sign this consent form. They will have the same study procedures as were described in the original consent form, but the version of study product given at their last injection may change.

- We do not expect there to be any additional risks from the updated study vaccine, but we do not know for sure.

1. **Review of study information**

You are a participant in CoVPN 3008 (Ubuntu), a research study that tests the Moderna COVID-19 vaccine in adults living with HIV and adults with existing health conditions that may put them at risk of severe COVID-19. The study is fully enrolled with over 14,000 participants. About half of the participants have completed their injections visits. Injection visits are ongoing for the rest. We are making an update to the study that we want to share with you.

Please review this information carefully. We also suggest that you review information in the main study consent form. You are free to ask questions at any time.

To show that you have received and understand this information, we will ask you to sign this form. You will get a copy to keep.

2. **What is changing in this study?**

Until now everyone in this study has gotten the first version of Moderna's vaccine (Moderna mRNA-1273). An updated version of this vaccine is now available (Moderna mRNA-1273.222). This updated version was made to target the newer Omicron subvariants, BA.4 and BA.5, as well as the original strain of SARS-CoV-2. This version was made to give better protection against these newer variants of the virus.

We want to test whether there a difference between the two study vaccines in their ability to protect against COVID-19 and the newer strains of the virus. To do this, at your last dose we will now either give you Moderna mRNA-1273 or the updated vaccine version.

If you have already completed all of your injection visits, you will not get the updated study vaccine.

3. **What do I need to know about the updated study vaccine?**

The updated study vaccine, Moderna mRNA-1273.222, was made by a similar approach to how influenza vaccine is made each year. It uses the Moderna mRNA-1273 vaccine and adds better coverage against current variants. Because the process for making the vaccine did not change, it does not need to be tested in large studies like the original vaccine. There are some smaller studies in progress. As of September 2022, the updated vaccine has been given to over 500 people as part of a study that is still ongoing. So far there have been no safety concerns. Studies done in mice showed that the updated vaccine worked well against both older and newer variants and we expect it will be the same in humans. Earlier versions of the updated study vaccine were given to over 700 people and there have been no safety concerns. We think that the risks of this updated study vaccine will be similar. But we do not know for sure.

In the United States (US), the updated study vaccine Moderna mRNA-1273.222 has been authorized for use in people by the Food and Drug Administration (FDA) and hundreds of thousands of previously vaccinated Americans have already received booster doses of it or a similar bivalent vaccine by Pfizer-BioNTech.

We will be using the new vaccine version authorized in the US. In this study, we will be giving it to you at a higher dose than the dose that is currently authorized in the US. This is because in the US the new version is currently being given as a booster vaccination, meaning an extra vaccination given to people already fully vaccinated to enhance or restore protection against COVID-19, which may decrease over time. In the US the booster vaccination dose for Moderna vaccines is half the dose of the vaccinations normally given at first to people to get them fully vaccinated. Because this study is evaluating potential ways of initially vaccinating people to get them fully vaccinated, this study is using a higher dose than the booster dose. The older version of the Moderna vaccine has already been approved and widely used at the higher dose, while the newer version has not yet.

4. **We will give you the study products on the same schedule, but the version of the study product you get at your last dose may change.**

All people in Group 1 and Group 3 will still get 3 doses of the study vaccines. The first 2 doses will be of the Moderna mRNA-1273 study vaccine. For the last dose, you will get either Moderna mRNA-1273 or the updated study vaccine.

All people in Group 2 and Group 4 will still get 2 doses of the study vaccines. The first dose will be of the Moderna mRNA-1273 study vaccine. For the last dose, you will get either Moderna mRNA-1273 or the updated study vaccine.

For all groups, which version of the study vaccine you get in the last dose is completely random, like flipping a coin. We have no say and will not know which version you get, and neither will you. Only the pharmacist and the person injecting you at this clinic will have this information while the study is going on. The updated study vaccine comes in a different volume. To keep you from knowing which version you are getting, we may ask you to turn you head away when you get your injection.

Because this is also the first time the updated study vaccine is being given to people at this dose, the study has a planned safety pause where safety information will be reviewed to decide if it is safe to continue. The pause will be after about 120 participants get the updated study vaccine and will determine if other participants should get it, too.

5. **You are free to choose to continue or to leave the study.**

   You are still free to leave the study at any time and for any reason. If you want to leave, you will need to tell us. Your care at this clinic and your legal rights will not be affected. We may ask you to come back to the clinic one last time, and we may ask to take some more samples. We believe this is important to protecting your health, but it is up to you whether you do this.

6. **Most things described in the informed consent form you signed previously remain the same.**

   These include:

   - The study procedures we will do;
   - The length of time you will be in the study;
   - The potential risks and benefits of being in the study;
   - Your rights and responsibilities in the study;
   - How your samples will be used;
   - What we will do if we find you have a health problem or you get COVID-19;
   - How we will protect your private information and who can access your study records;
   - Reasons we might take you out of the study;
   - What will happen if you get sick or injured during the study.

   As before, there is no cost to you for being in the study. We will give you [Site: Insert compensation] for each study visit you complete.

   In the consent form you signed when you joined this study, you chose whether the CoVPN could use your extra samples and information in other studies. The CoVPN will continue to

honor the choice you made in that consent form. You can change your mind if you want. Your decision will not affect your being in this study or have any negative consequences here.

7. **If you have questions or problems at any time during this study, use the following important contacts.**

If you have questions about this study, contact [name or title and telephone number of the investigator or other study staff].

If you have any symptoms that you think may be related to this study, contact [name or title and telephone number of the investigator or other study staff].

This study has been reviewed and approved by a committee called the [name of local IRB/EC]. If you have questions about your rights as a research participant, or problems or concerns about how you are being treated in this study, contact [name or title and telephone number of person on IRB/EC], at the committee.

Paragraph for South African sites. The study has been structured in accordance with the Declaration of Helsinki (last updated October 2013) which deals with the recommendations guiding doctors in biomedical research involving human participants, the Ethics in Health Research: Principles, Structures and Processes Second Edition 2015, and Guidelines for Good Practice in the Conduct of Clinical Trials in Human Participants in South Africa. We can provide you with copies of these guidelines if you wish to review them. In addition, the recent Protection of Personal Information Act (POPIA) ensures that all South African institutions conduct themselves in a responsible manner when collecting, processing, storing and sharing another entity's personal information by holding them accountable should they abuse or compromise your personal information in any way.

If you want to leave this study, contact [name or title and telephone number of the investigator or other study staff].

You can reach a study staff member 24-hours a day at [telephone number].

Remainder of section for South African sites only.

If you have questions about this trial you should first discuss them with your doctor or the ethics committee (contact details as provided on this form). After you have consulted your doctor or the ethics committee and if they have not provided you with answers to your satisfaction, you should write to the South African Healthcare Products Regulatory Authority (SAHPRA) at:

The Chief Executive Officer
Dr Boitumelo Semete-Makokotlela
South African Health Products Regulatory Authority Private Bag X828 PRETORIA
0001

Tel: (012) 501 0410/13
e-mail: Boitumelo.Semete@sahpra.org.za

8. **If you agree to continue in this study, you will need to sign or make your mark below. Before you sign or make your mark on this consent form, make sure of the following:**

- You have read this consent form, or someone has read it to you.
- You feel that you understand what the study is about and what will happen to you if you continue. You understand what the possible risks and benefits are.
- You have had your questions answered and know that you can ask more.
- You agree to stay in this study.

You will not be giving up any of your rights by signing this consent form.

Participant's name (print)	Participant's signature or mark	Date	Time
Clinic staff conducting consent discussion (print)	Clinic staff signature	Date	Time

For participants who are unable to read or write, a witness should complete the signature block below:

Witness's name (print)	Witness's signature	Date	Time

*Witness is impartial and was present for the consent process.

Appendix M Addendum 2 to the sample informed consent form for additional procedures

Title: Multi-Center, Randomized, Efficacy Study of COVID-19 mRNA Vaccine in Regions with SARS-CoV-2 Variants of Concern
Study number: CoVPN 3008 (Ubuntu)
Site: [Insert site name]

Key information

- Enrollment in this study is now complete with 14,237 participants.
- Some participants will be ending their participation earlier than others.
- For people staying in the study, we are changing the schedule of procedures to include monthly nasal swabs through your month 12 visit.
- We are decreasing the duration of per participant follow-up from 18 months to 12 months
- Participants will need to sign this addendum consent form.

1. Review of study information

You are a participant in CoVPN 3008 (Ubuntu), a research study that tests the Moderna COVID-19 vaccine in adults living with HIV and adults with existing health conditions that may put them at risk of severe COVID-19. The study is fully enrolled with 14,237 participants.

As you were informed recently, the study sponsors have done an early review of the study data and have made several observations. The mRNA vaccinations appear safe and well tolerated by study participants, including people living with HIV. Participants across all sites have rarely gotten seriously ill from COVID-19, especially if they previously had COVID-19. Testing has shown that quite a few participants across sites have gotten COVID-19 during the study but without symptoms, and some people have had infections for weeks or months, particularly some people living with HIV with low CD4 counts.

These observations, along with the fact that the pandemic and vaccine availability have changed since the Ubuntu study began, have led us to make some changes to the study as described below.

Please review this information carefully. We also suggest that you review information in the main study consent form and the first addendum to the consent form. You are free to ask questions at any time.

To show that you have received and understand this information, we will ask you to sign this form. You will get a copy to keep.

2. What is changing in this study?

We are making the following changes to the study:

- Most participants who received or will receive their final vaccination under version 5 of the study protocol will have a final visit as soon as possible (but at least a month after the final vaccination).

- About 400 participants in this group will be asked to remain in the study for further monitoring until approximately 12 months after enrolment.

- Participants who received their final vaccination under version 6 of the protocol (about 4000) will be asked to remain in the study until approximately 12 months after enrolment.

- All participants who remain in the study until month 12 after enrolment will also be asked to provide some additional nasal samples for COVID-19 testing.

- If you test positive for COVID-19 you will follow the same procedures in the main consent form, except for the following:

  If you are still testing positive for COVID-19 at your Day 42 swab, we will ask you to come to the clinic for a blood draw, and again every 8 weeks thereafter as long as you continue to test positive for COVID-19.

You are being asked to stay in the study to continue to provide valuable information. We will continue to do most of the same procedures that are described in the original consent form you signed that include:

- Doing physical exams

- Doing swabs of your nose

- Asking questions about your health, including medications you may be taking

- Asking you to keep track of any symptoms of COVID-19 or any other problems you might develop.

- Taking blood samples

In addition to the procedures above, we would like you to do monthly nasal swabs from month 6 through month 12. This means you will have additional swabs done, but the amount varies depending on where you are in the study. The updated schedule of procedures is shown below:

Procedures for Group 1 and Group 3

**Procedures**	Screening visit	First injection visit	1 month	2 months	6 months	7 months	8 months	9 months	10 months	11 months	12 months
Injection		√	√		√						
Physical exam	√										√
Medical history	√	√									
Interview /Questionnaire	√	√	√	√	√	√	√*	√*	√*	√*	√
Pregnancy test	√										
Ultrasound	√										
HIV test	√										
Blood drawn	√	√		√	√	√					√
Nasal swabs		√	√		√	√*	√*	√*	√*	√*	√*

* Additional procedure/timepoint

Procedures for Group 2 and Group 4

**Procedures**	Screening visit	First injection visit	1 month	6 months	7 months	8 months	9 months	10 months	11 months	12 months
Injection		√		√						
Physical exam	√									√
Medical history	√	√								
Interview/ Questionnaire	√	√	√	√	√	√*	√*	√*	√*	√
Pregnancy test	√									
Ultrasound*	√									
HIV test	√									
Blood drawn	√	√	√	√	√					√
Nasal swabs		√		√	√*	√*	√*	√*	√*	√*

* Additional procedure/timepoint

If you are testing positive for COVID-19 at Month 12, we will connect you with appropriate follow up as needed to support your care.

3. **You are free to choose to continue or to leave the study.**

   You are still free to leave the study at any time and for any reason. If you want to leave, you will need to tell us. Your care at this clinic and your legal rights will not be affected. We may ask you to come back to the clinic one last time, and we may ask to take some more samples. We believe this is important to protecting your health, but it is up to you whether you do this.

4. **Most things described in the informed consent form and first addendum to the consent form you signed previously remain the same.**

   These include:

   - The potential risks and benefits of being in the study;
   - Your rights and responsibilities in the study;
   - How your samples will be used;
   - How we will protect your private information and who can access your study records;
   - Reasons we might take you out of the study;
   - What will happen if you get sick or injured during the study.

5. **If you have questions or problems at any time during this study, use the following important contacts.**

   If you have questions about this study, contact [name or title and telephone number of the investigator or other study staff].

   If you have any symptoms that you think may be related to this study, contact [name or title and telephone number of the investigator or other study staff].

   This study has been reviewed and approved by a committee called the [name of local IRB/EC]. If you have questions about your rights as a research participant, or problems or concerns about how you are being treated in this study, contact [name or title and telephone number of person on IRB/EC], at the committee.

   Paragraph for South African sites. The study has been structured in accordance with the Declaration of Helsinki (last updated October 2013) which deals with the recommendations guiding doctors in biomedical research involving human participants, the Ethics in Health Research: Principles, Structures and Processes Second Edition 2015, and Guidelines for Good Practice in the Conduct of Clinical Trials in Human Participants in South Africa. We can provide you with copies of these guidelines if you wish to review them. In addition, the recent Protection of Personal Information Act (POPIA) ensures that all South African institutions conduct themselves in a responsible manner when collecting, processing, storing

and sharing another entity's personal information by holding them accountable should they abuse or compromise your personal information in any way.

If you want to leave this study, contact [name or title and telephone number of the investigator or other study staff].

You can reach a study staff member 24-hours a day at [telephone number].

Remainder of section for South African sites only.

If you have questions about this trial you should first discuss them with your doctor or the ethics committee (contact details as provided on this form). After you have consulted your doctor or the ethics committee and if they have not provided you with answers to your satisfaction, you should write to the South African Healthcare Products Regulatory Authority (SAHPRA) at:

The Chief Executive Officer
Dr Boitumelo Semete-Makokotlela
South African Health Products Regulatory Authority Private Bag X828 PRETORIA
0001
Tel: (012) 501 0410/13
e-mail: Boitumelo.Semete@sahpra.org.za

6. If you agree to continue in this study, you will need to sign or make your mark below. Before you sign or make your mark on this consent form, make sure of the following:

- You have read this consent form, or someone has read it to you.
- You feel that you understand what the study is about and what will happen to you if you continue. You understand what the possible risks and benefits are.
- You have had your questions answered and know that you can ask more.
- You agree to stay in this study.

You will not be giving up any of your rights by signing this consent form.

Participant's name (print)	Participant's signature or mark	Date	Time
Clinic staff conducting consent discussion (print)	Clinic staff signature	Date	Time

For participants who are unable to read or write, a witness should complete the signature block below:

Witness's name (print)	Witness's signature	Date	Time

*Witness is impartial and was present for the consent process.

Appendix N Adverse Events of Special Interest AESIs

Nonexhaustive list of examples of AEs of Special Interest (AESI) for this protocol, derived in large part from the Brighton Collaboration COVID-19 Updated AESI list (section 14):

1. Anosmia, Ageusia
2. Subacute thyroiditis
3. Acute pancreatitis
4. Appendicitis
5. Rhabdomyolysis
6. Acute respiratory distress syndrome (ARDS)
7. Coagulation disorders
8. Acute cardiovascular injury
9. Acute kidney injury
10. Acute liver injury
11. Dermatologic findings
12. Multisystem inflammatory disorders
13. Thrombocytopenia (platelet counts $< 125 \times 10^9$ per mm^3)
14. Acute aseptic arthritis
15. New onset of or worsening of neurologic disease
16. Anaphylaxis
17. Fibromyalgia
18. Postural Orthostatic Tachycardia Syndrome
19. Chronic Fatigue Syndrome (includes myalgic encephalomyelitis and postviral fatigue syndrome)
20. Myasthenia gravis

Note: SARS-CoV-2 symptomatic or asymptomatic infections / COVID-19 events are study endpoints and will not be reported as AEs or AESIs unless SAE criteria are met.

Appendix O Protocol signature page

Multi-Center, Randomized, Efficacy Study of COVID-19 mRNA Vaccine in Regions with SARS-CoV-2 Variants of Concern

I will conduct the study in accordance with the provisions of this protocol and all applicable protocol-related documents. I agree to conduct this study in compliance with United States (US.) Health and Human Service regulations (45 CFR 46); applicable US. Food and Drug Administration regulations; standards of the International Council for Harmonisation of Technical Requirements for Pharmaceuticals for Human Use (ICH) Guideline for Good Clinical Practice (E6) (R2); Institutional Review Board/Ethics Committee determinations; all applicable in-country, state, and local laws and regulations; and other applicable requirements (eg, US. National Institutes of Health, Division of AIDS) and institutional policies.

Investigator of Record Name (print)	Investigator of Record Signature	Date

Protocol Number: CoVPN 3008 (Ubuntu)

Protocol Version: Version 7.0

Protocol Date: February 27, 2023
