# Supplementary material for: Hybrid versus vaccine immunity of mRNA-1273 among people living with HIV in East and Southern Africa: a prospective cohort analysis from the multicentre CoVPN 3008 (Ubuntu) study
Source: eClinicalMedicine. 2025 Jan 20;80:103054. doi: 10.1016/j.eclinm.2024.103054 (PMC11788791; doi:10.1016/j.eclinm.2024.103054)
Supplement: Statistical Analysis Plan [file mmc2.pdf]

Statistical Analysis Plan for Comparing COVID-19 Risk in Ubuntu/CoVPN 3008: First Stage (Follow-up Through Month 6 Booster Dose) Final Analysis

(alphabetical order) Peter Gilbert, Yunda Huang, Aaron Hudson, Bo Zhang

Date finalized for signature: June 21, 2023
SAP effective upon last signature

Protocol version 7.0 (February 27, 2023)

SAP Version 2.0

SAP APPROVAL SIGNATURE PAGE

I have read this Statistical Analysis Plan and approve its contents.

See appended email approval	See appended email approval
Peter Gilbert, PhD<br>CoVPN 3008 Blinded Statistician	Date
See appended email approval	See appended email approval
Yunda Huang, PhD<br>CoVPN 3008 Blinded Statistician	Date
See appended email approval	See appended email approval
Aaron Hudson, PhD<br>CoVPN 3008 Blinded Statistician	Date
See appended email approval	See appended email approval
Bo Zhang, PhD<br>CoVPN 3008 Blinded Statistician	Date

SAP Modification History

The version history of, and modifications to, this statistical analysis plan are described below.

Date: April 21, 2023
Protocol version: 7.0
SAP version: 1.0

Date: June 21, 2023
Protocol version: 7.0
SAP version: 2.0
Modifications:

- Modified selection of timepoint t_0 in cumulative incidence analysis.
- In cumulative incidence analysis, participants with endpoints after t_0 are now to be treated as censored outcomes.
- Clarified how enrollment date is to be treated as binary in variable selection strategy (Section 7.3.2).
- Removed statement that for the purposes of variable selection in Section 7.3.2, continuous covariates are dichomizted by being above or below their median value, as there are no continuous covariates in the exploratory analysis.
- Added calculation of E-values in Section 11.
- Clarified that persistent NAAT positivity is considered only for the first infection in the described analysis (Section 12).

Contents

List of Tables **6**

List of Figures **7**

1 Introduction **7**

2 Baseline SARS-CoV-2 status and analysis group definition **7**

3 Primary, secondary and exploratory comparisons of COVID-19 risk **8**

4 Study endpoints **9**
4.1 Primary COVID-19 endpoint 9
4.1.1 COVE-based primary COVID-19 endpoint definition 9
4.1.2 CDC-based primary COVID-19 endpoint definition 10

5 Cohorts and time frames for COVID-19 endpoint evaluation **11**

6 Parameters of interest to estimate **12**
6.1 Cumulative-failure-time-based parameters of interest 12
6.2 Selection of the final time point t_0 13
6.3 Cox-regression-based parameters of interest 13

7 Statistical methods for estimation/inference **14**
7.1 Set of statistical analyses 14
7.2 Hypothesis tests 15
7.3 Cumulative failure time approach 15
7.3.1 Primary and Secondary Comparisons 17
7.3.2 Exploratory Comparisons 18
7.4 Cox modeling approach 20
7.4.1 Primary comparison 20
7.4.2 Secondary comparison 21
7.4.3 Exploratory comparisons 22

8 Primary versus sensitivity analyses **23**

9 Multiplicity adjustment **23**

10 Handling missing data **24**

11 Sensitivity analyses of primary and secondary comparisons to unmeasured confounders **24**

12 Persistent NAAT positivity **24**
12.1 Descriptive analysis including all baseline and post-baseline NAAT positive infections 24
12.2 Subgroup analyses pertaining to symptomatic infections diagnosed post baseline . . 25

12.3 Regression modeling of NAAT persistence 25

List of Tables

1 **Panel A**: Empirical probability that CLS or NAAT test result was positive conditional on different configurations of the other two test results among 13,857/14,040 participants with all three test results measured. **Panel B**: Empirical probability that CLS or NAAT was positive conditional on POCS test result among 13,929/14,040 participants with both CLS and POCS test results and among 13,915/14,040 participants with both NAAT and POCS test results, respectively. 10

1 Introduction

This SAP focuses on comparisons of COVID-19 risk between analysis groups defined by diagnostic testing data on prior SARS-CoV-2 infection and the number of mRNA-1273 vaccination doses, with endpoints counted up to the Month 6 visit when the Month 6 booster dose is received. In addition, analysis of persistent Nucleic Acid Amplification Test (NAAT) positivity as evidence of persistent viral shedding is described for SARS-CoV-2 infections confirmed by NAAT after study enrolment. These analyses address the co-primary objectives and key secondary objectives of the Pre-Month 6 stage trial period as specified in the CoVPN 3008 protocol. Section 2 defines the baseline SARS-CoV-2 status (baseline SARS-CoV-2 naïve and two ways to define baseline SARS-CoV-2 non-naïve) and the 6 derived analysis groups. Section 3 specifies the primary, secondary and exploratory comparisons of COVID-19 risk and overview of analysis approaches.

Section 4 defines primary and secondary study endpoints. Different analyses specify different time origins for the COVID-19 time-to-event T; the endpoint date is always the same: the date of the COVID-19 endpoint up to the date of receiving the booster dose. The failure time T in the Pre-Month 6 stage is right-censored by the first event of receipt of the booster dose, outside vaccination, loss to follow-up, or reaching March 31, 2023, whichever occurs first. Details regarding the time frames, parameters of interest, and statistical methods for estimation can be found in Section 5, 6, and 7, respectively. Section12 specifies the analysis of persistent NAAT positivity.

2 Baseline SARS-CoV-2 status and analysis group definition

This section defines baseline SARS-CoV-2 status and analysis groups. According to the protocol, in addition to the Point-of-Care serology (POCS) testing result, which was used for assigning participants into the 4 study groups at enrolment, the central lab serology (CLS) testing result and the Nucleic Acid Amplification Test (NAAT) result are also used to define the SARS-CoV-2 naïve and non-naïve status in subsequent statistical analyses. Specifically, a participant is said to be baseline SARS-CoV-2 naïve (or negative) if all 3 test are negative. A participant is said to be non-naïve (or positive) if at least 1 of the 3 tests is positive. We will further differentiate between two types of non-naïve participants: (i) those who tested negative for POCS but positive for either CLS or NAAT; and (ii) those who tested positive for POCS.

These definitions will be applied after imputing missing baseline CLS and NAAT testing results according to the following imputation scheme. For participants who missed the CLS result but had both POCS and NAAT results, the missing CLS result was imputed as positive if either POCS or NAAT was positive and negative if both POCS and NAAT were negative. For participants who missed the NAAT result but had both POCS and CLS results, the missing NAAT result was always imputed as negative. For participants who missed both CLS and NAAT test results, their missing CLS test result was imputed as positive if POCS result was positive and negative if the POCS result was negative. Their NAAT result was always imputed as negative. This imputation scheme was motivated by the joint distribution of POCS, CLS and NAAT among those who had complete measurements of test results (more than 98% of the study cohort); see Table 1 for various empirical conditional probabilities. We also considered these conditional probabilities separately in the stratum defined by baseline HIV status; results were qualitatively similar and the derived

imputation scheme was the same.

To summarize, data analysis is conducted based on the following 6 analysis groups, all of which are defined at enrolment:

1. G1 = PLWH; baseline SARS-CoV-2 naïve (negative for POCS, CLS, and NAAT); assigned 2 vaccinations pre-Month 6
2. G2-I = PLWH; baseline POCS positive; assigned 1 vaccination pre-Month 6
3. G2-II = PLWH; baseline POCS negative but CLS or NAAT positive; assigned 2 vaccinations pre-Month 6
4. G3 = HIV-negative; baseline SARS-CoV-2 naïve (negative for POCS, CLS, and NAAT); assigned 2 vaccinations pre-Month 6
5. G4-I = HIV-negative; baseline POCS positive; assigned 1 vaccination pre-Month 6
6. G4-II = HIV-negative; baseline POCS negative but CLS or NAAT positive; assigned 2 vaccinations pre-Month 6

3 Primary, secondary and exploratory comparisons of COVID-19 risk

We will consider the following primary comparison:

- G1 vs. G2-I

to address the following primary objective specified in the protocol:

> To assess the relative risk of virologically confirmed symptomatic COVID-19 between a 2-dose mRNA-1273 vaccine regimen in adult people living with HIV (PLWH) who are SARS-CoV-2 negative at baseline (Group 1, vaccine-only immunity) vs. a 1-dose mRNA-1273 regimen in adult PLWH who are SARS-CoV-2 positive at baseline (Group 2, hybrid immunity), counting endpoints starting 1 day after Month 0 dose until the Month 6 dose.

We will consider the following secondary comparison:

- G1 + G3 vs. G2-I + G4-I; PLWH and HIV-negative pooled; SARS-CoV-2 naïve vs. SARS-CoV-2 non-naïve (POCS positive);

to address the following secondary objective specified in the protocol:

> To assess the relative risk of symptomatic COVID-19 and of severe COVID-19 in Groups 1 and 3 together (participants who are SARS-CoV-2 negative at baseline regardless of HIV status) vs. in Groups 2 and 4 together (participants who are SARS-CoV-2 positive at baseline regardless of HIV status), counting endpoints starting 1 day after dose 1, and counting endpoints at least 14 days after the last pre-Month 6 dose (ie, dose 2 for Group 1 and dose 1 for Group 2) until the Month 6 dose.

We will consider the following exploratory comparisons:

- G1 vs. G3: SARS-CoV-2 naïve; PLWH vs. HIV-negative; assigned 2 vaccinations pre-Month 6;
- G2-I vs. G4-I: SARS-CoV-2 non-naïve (POCS positive); PLWH vs. HIV-negative; assigned 1 vaccination pre-Month 6
- G3 vs. G4-I: HIV-negative; SARS-CoV-2 naïve and assigned 2 vaccinations pre-Month 6 vs. SARS-CoV-2 non-naïve (POCS positive) and assigned 1 vaccination pre-Month 6;
- G2-I vs G2-II: PLWH; SARS-CoV-2 non-naïve (POCS positive) and assigned 1 vaccination pre-Month 6 vs SARS-CoV-2 non-naïve (POCS negative but CLS or NAAT positive) and assigned 2 vaccinations pre-Month 6
- G1 vs G2-II: PLWH; assigned 2 vaccinations pre-Month 6; SARS-CoV-2 naïve vs non-naïve (POCS negative but CLS or NAAT positive)
- G4-I vs G4-II: HIV-negative; SARS-CoV-2 non-naïve (POCS positive) and assigned 1 vaccination pre-Month 6 vs SARS-CoV-2 non-naïve (POCS negative but CLS or NAAT positive) and assigned 2 vaccinations pre-Month 6
- G3 vs G4-II: HIV-negative; assigned 2 vaccinations pre-Month 6; SARS-CoV-2 naïve vs non-naïve (POCS negative but CLS or NAAT positive)

Kaplan-Meier estimates will be provided for all comparisons. All exploratory comparisons will be performed using the Cox regression model. Exploratory comparisons will also be performed inferentially using the covariate-adjusted cumulative incidence approach, but only in the presence of enough failure events (because of the stability issue of the resulting estimator); details can be found in Section 7.3.

4 Study endpoints

This section defines the primary COVID-19 endpoint. In future SAP versions, other secondary endpoints including asymptomatic/subclinical SARS-CoV-2 infection, overall SARS-CoV-2 infection regardless of symptomology, as well as new infection confirmed by genetic sequence data will be described.

4.1 Primary COVID-19 endpoint

According to Version 7 of the protocol, all symptomatic COVID-19 endpoints will be evaluated using two case definitions, one used in the COVE study and the other using a case definition based on criteria from the CDC.

4.1.1 COVE-based primary COVID-19 endpoint definition

The COVE-based primary COVID-19 endpoint is defined as the first occurrence of adjudicated symptom-triggered NAAT-confirmed COVID-19 based on the following criteria (same as in the Moderna mRNA-1273 COVE trial):

Table 1: **Panel A**: Empirical probability that CLS or NAAT test result was positive conditional on different configurations of the other two test results among $13,857/14,040$ participants with all three test results measured. **Panel B**: Empirical probability that CLS or NAAT was positive conditional on POCS test result among $13,929/14,040$ participants with both CLS and POCS test results and among $13,915/14,040$ participants with both NAAT and POCS test results, respectively.

	Value
**Panel A**	
$P(\text{CLS} = 1 \mid \text{POCS} = 0 \ \& \ \text{NAAT} = 0)$	0.29
$P(\text{CLS} = 1 \mid \text{POCS} = 1 \ \& \ \text{NAAT} = 0)$	0.58
$P(\text{CLS} = 1 \mid \text{POCS} = 1 \ \& \ \text{NAAT} = 1)$	0.80
$P(\text{CLS} = 1 \mid \text{POCS} = 0 \ \& \ \text{NAAT} = 1)$	0.59
$P(\text{NAAT} = 1 \mid \text{POCS} = 0 \ \& \ \text{CLS} = 0)$	0
$P(\text{NAAT} = 1 \mid \text{POCS} = 1 \ \& \ \text{CLS} = 1)$	0.07
$P(\text{NAAT} = 1 \mid \text{POCS} = 1 \ \& \ \text{CLS} = 0)$	0
$P(\text{NAAT} = 1 \mid \text{POCS} = 0 \ \& \ \text{CLS} = 1)$	0.23
**Panel B**	
$P(\text{CLS} = 1 \mid \text{POCS} = 0)$	0.32
$P(\text{CLS} = 1 \mid \text{POCS} = 1)$	0.59
$P(\text{NAAT} = 1 \mid \text{POCS} = 0)$	0.07
$P(\text{NAAT} = 1 \mid \text{POCS} = 1)$	0.04

- At least TWO of the following systemic symptoms: Fever $\geq 38°$C), chills, myalgia, headache, sore throat, new loss of taste or smell, OR
- At least ONE of the following respiratory signs/symptoms: cough, shortness of breath or difficulty breathing, OR clinical or radiographical evidence of pneumonia; AND
- At least ONE (post-baseline) nasal swab (or respiratory sample, if hospitalized) positive for SARS-CoV-2 by NAAT.

The date of a documented/adjudicated COVID-19 endpoint is the later date of a symptom and the date of positive NAAT. This (event) date is supplied by the Endpoint Adjudication Committee. This endpoint is coded as **ADJD1** or **ADJLD14** in the Analysis Data Model (ADaM) datasets, counting events, respectively, 1 day after enrolment or 14 days after the last vaccination dose in the pre-Month 6 stage.

4.1.2 CDC-based primary COVID-19 endpoint definition

The CDC-based definition of the COVID-19 endpoint is defined as symptomatic NAAT-confirmed COVID-19 based on CDC criteria, where the nucleic acid test NAAT may or may not be triggered by symptoms. Results based on this alternative definition are included alongside those based on the primary COVID-19 endpoint. No multiplicity adjustments are applied to adjust for analyses of multiple COVID-19 endpoints. The CDC-based COVID-19 endpoint is defined as the first occurrence of symptomatic NAAT-confirmed COVID-19 based on the following criteria:

- At least ONE of the following systemic or respiratory symptoms: Fever ($\geq$ 38°C), chills, cough, shortness of breath and/or difficulty breathing, fatigue, muscle and/or body aches [not related to exercise], headache, new loss of taste/smell, sore throat, congestion, runny nose, nausea, vomiting, or diarrhea; AND
- At least ONE (post-baseline) nasal swab (or respiratory sample, if hospitalized) positive for SARS-CoV-2 by NAAT.
- The symptom date and NAAT positive date must be within 14 days of each other. Specifically, if the onset of qualifying symptom(s) occurred prior to the specimen collection date of a NAAT-positive result, then the date of last reported symptom(s) must be after 14 days prior to the NAAT-positive date. If the onset of qualifying symptoms occurred after a NAAT-positive date, then the onset date of qualifying symptom(s) must be within 14 days of the NAAT-positive date.

The date of a COVID-19 endpoint according to the CDC-based definition is the earlier date of a symptom and the date of positive NAAT that satisfy the criteria stated above.

Because additional NAAT testing was performed at the M1 vaccination visit for Groups 1 and 3 participants, but not for Groups 2-I, 2-II, 4-I and 4-II participants, different endpoints were derived to include (CDC1) or exclude (CDC2) NAAT testing results at M1 for comparisons of COVID risk between different groups. Specifically,

- For comparisons of two groups where only one group was but the other was not assigned the M1 vaccination, including the primary comparison G1 vs. G2-I, secondary comparison G1+G3 vs. G2-I+G4-I, and exploratory comparisons G3 vs. G4-I, G2-I vs. G2-II, G4-I vs. G4-II, the **CDC2D1** or **CDC2LD14** endpoint is used, counting events, respectively, 1 day after enrolment or 14 days after the last vaccination in the pre-Month 6 stage. These two endpoints consider post-baseline NAAT testing triggered by symptoms, by outside testing or other reasons, and testing performed at M6.
- For comparisons of two groups neither of which was assigned the M1 vaccination (i.e., no routine NAAT testing at M1), including the exploratory comparisons G2-I vs. G4-I, the **CDC2D1** or **CDC2LD14** endpoint should also be used, counting events, respectively, 1 day after enrolment or 14 days after the last vaccination in the pre-Month 6 stage. These two endpoints consider post-baseline NAAT testing triggered by symptoms, by outside testing or other reasons, and testing performed at M6.
- For comparisons of two groups where both were assigned the M1 vaccination (i.e., routine NAAT testing at M1), including exploratory comparisons G1 vs. G3, G1 vs. G2-II and G3 vs. G4-II, the **CDC1D1** or **CDC1LD14** endpoint in the ADaM datasets is used, counting events, respectively, 1 day after enrolment or 14 days after the last vaccination in the pre-Month 6 stage. These two endpoints consider post-baseline NAAT testing triggered by symptoms, by outside testing or other reasons, and testing performed at M1 and M6 visits.

5 Cohorts and time frames for COVID-19 endpoint evaluation

We refer to the following four situations as **"Scenarios"** for setting up the data analysis.

1. Time origin date of first vaccine dose; full analysis set (FAS) cohort; SARS-CoV-2 naïve or non-naïve (POCS positive) as specified in Section 2; count all COVID-19 endpoints after dose one

2. Time origin 14 days after last dose; per-protocol cohort (PP); SARS-CoV-2 naïve or non-naïve (POCS positive) defined as in scenario 1 above; count all COVID-19 endpoints starting 14 days after last dose

3. Time origin number of days between the first dose of the first enrolled participant (calendar time scale used only for the calendar-time based Cox modeling); FAS cohort; SARS-CoV-2 naïve or non-naïve (POCS positive) defined as in scenario 1 above; count all COVID-19 endpoints after dose one

4. Time origin number of days between 13 days after the last dose and this date for the first person in the study receiving a last dose (calendar time scale used only for the calendar-time based Cox modeling); PP cohort; SARS-CoV-2 naïve or non-naïve (POCS positive) defined as in scenario 1 above; count all COVID-19 endpoints starting 14 days after last dose

6 Parameters of interest to estimate

6.1 Cumulative-failure-time-based parameters of interest

Let T be the failure time using the study time scale (based on one of the study time origins defined above), t_0 a fixed time point of interest after the given time origin, W baseline covariates, and A the indicator of being SARS-CoV-2 non-naïve for a given way that it is defined in Section 2. Let S_0 be a baseline anti-spike antibody immune marker [we consider $\log_{10}$-scale anti-spike IgG concentration and $\log_{10}$-scale 50% neutralizing antibody titer ("nAb titer")] in the non-naïve subgroup $A = 1$. One challenge posed to causal inference with A considered to be an exposure or treatment is multiple versions of treatment (a violation of SUTVA); e.g. if A is thought of as "previous infection vs. not", then variations in time between previous infection and enrollment can be viewed to constitute multiple versions of treatment. All of the analyses make inference on statistical association parameters involving A that are free from this challenge; it should be borne in mind when pursuing causal inferences on the effect of A.

First, parameters of interest are the marginalized failure time cdfs $E_W[P(T \leq t_0|A = a, W)]$ over a range of fixed time points t_0, and the contrasts $\theta^{\text{diff}}(t_0) = E_W[P(T \leq t_0|A = 1, W)] - E_W[P(T \leq t_0|A = 0, W)]$ and $\theta^{rr}(t_0) = E_W[P(T \leq t_0|A = 1, W)]/E_W[P(T \leq t_0|A = 0, W)]$. Under standard causal assumptions, the statistical parameter $E_W[P(T \leq t_0|A = a, W)]$ equals the causal parameter $E[T(a) \leq t_0]$, the counterfactual failure time cdf under the point treatment defined by SARS-CoV-2 naïve or non-naïve, and $\theta^{\text{diff}}(t_0)$ and $\theta^{rr}(t_0)$ are two versions of an average treatment effect.

Even if the causal assumptions fail, the parameter $E_W[P(T \leq t_0|A = a, W)]$ may still be of greater scientific interest than the unadjusted survival probability. The first reason for this is that this parameter allows for the adjustment of covariates related to both A and T. As a result, this parameter can be interpreted as the average probability that $T \leq t_0$ in a hypothetical population of participants with $A = a$ but with a distribution of the covariate vector W identical to that in the target population. The second reason is that adjusting for W allows the relaxation of the

marginal independent censoring assumption $T(a) \perp C(a)|A$ to a conditional independent censoring assumption $T(a) \perp C(a)|A, W$. This relaxation can be important in contexts where the event and censoring times may be dependent, but the recorded covariate vector W at least partly explains the dependence between them.

Second, restricting to non-naïve individuals, we consider cumulative distribution functions $E_W[P(T \leq t_0|S_0 = s_0, A = 1, W)]$ for S_0 an anti-spike antibody marker as noted above, which is a "continuous treatment." These analyses will be conducted following a future version of the SAP, as the data on S_0 are not yet available.

6.2 Selection of the final time point t_0

For the FAS cohort, and for a given analysis group comparison, the fixed time point t_0 is chosen to be minimum of 180 days and the latest possible time point when stable estimation using follow-up data through the Month 6 booster dose can be reasonably assured. For the PP cohort, and for a given analysis group comparison, the fixed time point t_0 is chosen to be minimum of 165 days and the latest possible time point when stable estimation using follow-up data through the Month 6 booster dose can be reasonably assured. Identifying the minimum t_0 for which stable estimation can be assured is operationalized by selecting the latest point for which the standard error for the log risk ratio satisfies

$$\left|\frac{\log(3)}{\widehat{SE}(t)}\right| \geq z^{1-\alpha/2},$$

where $\widehat{SE}(t)$ denotes the estimated standard error for the log of the cumulative risk ratio at time t and $z^{1-\alpha/2}$ is the $1-\alpha/2$ quantile of the standard normal distribution. The rationale is we would only consider an estimate valuable if we have enough precision to conclude that a large cumulative risk ratio (of size 3.0 or larger) is statistically significant at the level $1-\alpha$. Study participants with an endpoint observed after t_0 are considered to have censored outcomes.

6.3 Cox-regression-based parameters of interest

Third, we consider parameters of interest defined under the assumption of a proportional hazards model. Suppose there are J baseline strata, $j = 1, \cdots, J$. The proportional hazards model can be written as

$$\lambda_j(t|A, W) = \lambda_{0j}(t) \exp\left\{\beta A + \gamma^T W\right\} \tag{1}$$

for binary A, or

$$\lambda_j(t|S_0, A = 1, W) = \lambda_{0j}(t) \exp\left\{g(S_0; \beta) + \gamma^T W\right\} \tag{2}$$

for the continuous antibody marker S_0 for some specified function $g(S_0; \beta)$ such as $g(S_0; \beta) = \beta S_0$. In addition to the β parameters, the same marginalized-mean cdfs considered above are of interest, which can be written in terms of the Cox models, with T on study time.

Fourth, we consider the same Cox models, except now T is the time between the date that a participant enrolls and the date that the first participant enrolls (i.e., calendar time). Consider a given time origin listed in Section 5: for concreteness let this be 14 days after the last dose (D14). Participants enter the risk set at D14 and are censored at enrollment if they acquire the COVID-19 endpoint prior to D14. For example, the Cox model that studies S_0 for participant i is written as

$$\lambda_{ji}(t|S_{i0}, A_i = 1, W_i) = \lambda_{0j}(t) \exp\left\{g(S_{i0}; \beta) + \gamma^T W_i\right\} I(\tau_i^{D14} > t), \tag{3}$$

where τ_i^{D14} is the calendar time of D14 for participant i.

The β coefficients have the same interpretation as the Cox models that model T in study time; the reason to consider this approach is for flexible nonparametric modeling of the baseline hazard as a function of calendar time, acknowledging that the COVID-19 epidemic undergoes unpredictable changes over calendar time. The β coefficients for these calendar-time Cox models are the coefficients for which results are reported; results for the β coefficients for the study time Cox modeling are not reported, to avoid too many results and potential confusion. Therefore, the use of the Cox model on the study/enrolment time scale is solely as an intermediate step in covariate-adjusted cumulative incidence estimation, given that the cumulative incidence results use study/enrolment time.

For cumulative incidence based analyses discussed in the previous section, a final time point t_0 was selected and failure times beyond t_0 were right-censored at t_0. The calendar-time Cox models do not use this fixed value of t_0, rather they consider all follow-up up through the M6 vaccination. Right-censoring is by the first event of receiving M6 vaccination, early termination, or last visit before the data cut.

7 Statistical methods for estimation/inference

7.1 Set of statistical analyses

The data analyses are set up differently depending on the analysis groups that are being compared (due to assignment to 1 vs. 2 vaccinations or due to different baseline covariate vectors W for PLWH and for HIV-neg).

In describing the analyses, first the approach to the primary comparison (G1 vs. G2-I) is described. Then in turn modifications of the analysis for the secondary comparison and each of the exploratory comparisons are described.

The following statistical approaches are employed, with the scenarios defined in Section 5 for application indicated.

1. (Scenarios 1, 2) Estimation of $E_W[P(T \leq t_0|A = a, W)]$, $a = 0, 1$, $\theta^{\text{diff}}(t_0)$, $\theta^{rr}(t_0)$, using nonparametric methods.
2. (Scenarios 1, 2) Estimation of $E_W[P(T \leq t_0|S_0 = s_0, A = 1, W)]$ over the span of s_0 values, using a nonparametric method to be determined in a future version of the SAP.

3. (Scenarios 1, 2) Estimate the same parameters as estimated in 1 and 2 above except using semiparametric Cox modeling with T being the study time. While this approach also provides estimates of β coefficients, they are not used for results so as to avoid complications in interpretations when also reporting results for β coefficients for the calendar-time Cox modeling noted next (this analysis is used for covariate-adjusted cumulative incidence estimation).

4. (Scenario 3, 4) Cox proportional hazards modeling with failure time on the calendar time scale and exposure variable the indicator of being SARS-CoV-2 non-naïve, for estimation of β coefficients (this analysis does not estimate marginalized cumulative failure time parameters).

7.2 Hypothesis tests

Each nonparametric method described above provides 95% confidence intervals about the parameters of interest as well as two-sided p-values for testing the following null hypotheses of interest (with t_0 fixed at the latest time point of interest):

$$\text{(A dichotomous)} \quad H_0: \quad \theta^{\text{diff}}(t_0) = 0, \quad \theta^{\text{rr}}(t_0) = 1 \tag{4}$$

$$\text{(Continuous S_0)} \quad H_0: \quad E_W[P(T \leq t_0 | S_0 = s_0, A = 1, W)] = E_W[P(T \leq t_0 | A = 1, W)]. \tag{5}$$

Each Cox modeling method provides two-sided p-values for testing the following null hypotheses of interest:

$$\text{(A dichotomous)} \quad H_0: \quad \beta = 0 \tag{6}$$

$$\text{(Continuous S_0)} \quad H_0: \quad g(s_0; \beta) = 0 \tag{7}$$

A partial likelihood ratio test will be used for testing each of the first two Cox model null hypotheses. For the continuous S_0 modeling with $g(s_0; \beta) = \beta s_0$, a generalized Wald test will be used for testing the null (7), accounting for the 2-phase sampling of S_0.

7.3 Cumulative failure time approach

Below, we first describe our general approach for estimating the cumulative incidence parameter, and we subsequently provide details describing how this approach is applied for each of the contrasts of interest.

We estimate the marginalized cumulative incidence function $\Gamma^a(t) = E_W[P(T \leq t | A = a, W)]$ using the strategy proposed by Westling et al. (2023). Estimating the marginalized cumulative incidence function requires estimation of the following three nusiance functions:

1. The conditional probability of the outcome by or prior to time t, given analysis group and covariates, $\eta_1(t, a, w) = P(T \leq t | A = a, W = w)$

2. The conditional probability of censoring by or prior to time t, given analysis group and covariates, $\eta_2(t, a, w) = P(C \leq t | A = a, W)$ (where C is the censoring time)

3. The conditional probability of analysis group given the covariate vector, $\pi(a|w) = P(A = a|W = w)$ also referred to as the propensity score

The approach we consider allows for us to supply nonparametric estimators for each of the above nuisances, which could be obtained using a flexible machine learning procedure (e.g., the Super Learner). In this case, our estimator of $\Gamma^a(t)$ is fully nonparametric, and resulting inferences are valid even if one of the the nuisance parameters is not estimated well. However, it can be advantageous to also consider an approach based on parametric modeling of the nuisances in consideration of various practical issues. In particular, nonparametric estimators for the survival function can be computationally intensive and have long run times when the sample size is large; they can also be computationally unstable when the number of endpoints is small, and they can have higher variance than more parametric methods. In view of these considerations, we consider application of both parametric and nonparametric approaches for nuisance estimation.

Parametric modeling approach: We estimate the conditional survival functions η_1 and η_2 using a Cox proportional-hazards model in conjunction with the Breslow estimator for the baseline hazard. Our Cox regression models include linear terms for each covariate, and no interaction or higher order terms (e.g., splines) unless otherwise specified. We estimate π using logistic regression, and similarly, we only include linear terms unless otherwise specified.

Nonparametric modeling approach: Each of the nuisance parameters is estimated using a super learner. For the survival functions η_1 and η_2, the super learner library consists of the following estimators: (1) Kaplan-Meier estimator; (2) Cox proportional hazard estimator; (3) generalized additive models; (4) survival random forests. The super learner library for the propensity score contains: (1) the sample average of A – i.e., an intercept-only model; (2) logistic regression model; (3) generalized additive models; (4) random forests. Cross-fitting is performed in order to avoid assumptions that would disallow the use of flexible estimators that do not satisfy complex conditions.

The following figures and tables are to be produced for an analysis performed under any setting (i.e. for a given starting time, definition of baseline SARS-CoV-2 positive, choice of group comparison, and covariate adjustment strategy):

- A table reporting estimates, standard errors, 95% confidence intervals, and 2-sided p-values for the following quantities (p-values are only reported for contrasts) for each pair of analysis groups that are compared:
  - Marginalized cumulative incidence evaluated at time t_0 in each analysis group
  - Ratio of marginalized cumulative incidences at time t_0 comparing the two analysis groups
  - Difference in marginalized cumulative incidences at time t_0 comparing the two analysis groups
- A multi-panel figure showing the following:
  - Estimates of the marginalized cumulative incidence function for $t \leq t_0$ for each analysis group, with both pointwise and uniform 95% confidence bands
  - Estimates of the ratio of the marginalized cumulative incidence functions, comparing the two analysis groups, for $t \leq t_0$, with both pointwise and uniform 95% confidence bands

– Estimates of the difference between the marginalized cumulative incidence functions, comparing the two analysis groups groups, for $t \leq t_0$, with both pointwise and uniform 95% confidence bands

Each of the quantities above are obtained using the `CFSurvival` package in `R`.

7.3.1 Primary and Secondary Comparisons

The analysis for the primary comparison assesses differences in covariate-adjusted cumulative incidence by baseline SARS-CoV-2 status among people living with HIV (G1 vs. G2-I). The analysis for the secondary comparison assesses differences in covariate-adjusted cumulative incidence by baseline SARS-CoV-2 status among persons living with HIV and persons not living with HIV (G1 + G3 vs G2-I + G4-I). For any given origin date, the failure time is calculated as the number of days between the origin and the date of the study endpoint (by a given definition). Similarly, the censoring time is calculated as the number of days between the origin and the date of censoring.

Each of the the following covariate adjustment strategies are used for both the primary and secondary comparisons in the cumulative incidence approach.

Parametric modeling; binary covariates
We use the following variables to produce the Cox proportional hazards fit of η_1 and η_2: an indicator for baseline SARS-CoV-2 status (for a given definition), a binary indicator of being enrolled in South Africa, a binary indicator of age being less than or equal to 40 years, sex assigned at birth, a binary indicator of BMI being less than or equal to 25, an interaction term between the BMI and sex indicators, a categorical variable indicating the period of enrollment (3 levels are used: Dec-Mar; Apr-Jun; Jul-Sept), a binary indicator of tuberculosis at baseline, a binary indicator of CD4+ cell counts being less than or equal to 500, and an indicator of whether the baseline HIV viral load is detectable. The logistic regression fit of π is produced using the same variables, except excluding baseline SARS-CoV-2 status.

Parametric modeling; continuous covariates
We use the following variables to produce the Cox proportional hazards fit of η_1 and η_2: an indicator for baseline SARS-CoV-2 status (for a given definition), a binary indicator of being enrolled in South Africa, age (treated as continuous), sex assigned at birth, BMI (treated as continuous), an interaction term between the continuous BMI variable and the sex assigned at birth indicator, a categorical variable indicating the period of enrollment (3 levels are used: Dec-Mar; Apr-Jun; Jul-Sept), a binary indicator of tuberculosis at baseline, CD4+ cell count (treated as continuous), and $\log_{10}$-transformed baseline HIV viral load. The logistic regression fit of π is produced using the same variables, except excluding baseline SARS-CoV-2 status.

Nonparametric modeling; binary covariates
The following covariates are supplied to the Super Learner for estimating η_1 and η_2: an indicator for baseline SARS-CoV-2 status (for a given definition), a binary indicator of being enrolled in South Africa, a binary indicator of age being less than or equal to 40 years, sex assigned at birth, a binary indicator of BMI being less than or equal to 25, an interaction term between the BMI and sex indicators, a categorical variable indicating the period of enrollment (3 levels are used:

Dec-Mar; Apr-Jun; Jul-Sept), a binary indicator of tuberculosis at baseline, a binary indicator of CD4+ cell counts being less than or equal to 500, and an indicator of whether the baseline HIV viral load is detectable. The Super Learner estimate for π is produced using the same variables, aside from baseline SARS-CoV-2 status.

Nonparametric modeling; continuous covariates

The following covariates are supplied to the Super Learner for estimating η_1 and η_2: an indicator for baseline SARS-CoV-2 status (for a given definition), a binary indicator of being enrolled in South Africa, age (treated as continuous), sex assigned at birth, BMI (treated as continuous), an interaction term between the continuous BMI variable and the sex assigned at birth indicator, a categorical variable indicating the period of enrollment (3 levels are used: Dec-Mar; Apr-Jun; Jul-Sept), a binary indicator of tuberculosis at baseline, CD4+ cell count (treated as continuous), and $\log_{10}$-transformed baseline HIV viral load. The Super Learner estimate for π is produced using the same variables, aside from baseline SARS-CoV-2 status.

Main analysis vs sensitivity analysis

Of the strategies described above, the parametric modeling approach with binary covariates is treated as the main analysis strategy, and the other three strategies are treated as sensitivity analyses.

Figures and tables to be presented

For both the primary and secondary comparisons, the figures and tables described in the beginning of Section 7.3 are produced under each of the following settings:

1. Two origin time points (1 & 2) described in Section 5
2. Two endpoint definitions described in Section 4
3. Four covariate adjustment strategies – adjusting for either continuous or binary versions of the covariates, as well as parametric or nonparametric estimation of the nuisance parameters.

7.3.2 Exploratory Comparisons

The analyses for the exploratory comparisons are very similar to those used for the primary comparison. The main difference in our approach is that our covariate adjustments will be different for each comparison because the notion of "treatment" varies.

Below, we describe an approach for covariate adjustment in the cumulative incidence analyses. While we considered four different covariate adjustment strategies for the primary and secondary comparisons, for the exploratory comparisons, we only consider the parametric modeling strategy with binary covariates. In what follows, we first describe the set of covariates that can be considered as potential adjustments for any exploratory comparison. For these comparisons, there may be too-few study endpoints to support adjustment for the full set of baseline covariates listed. To address this issue, we also use an approach to determine which subset of covariates to use for adjustment; this method is also described below.

Full set of candidate covariate adjustments for each comparison group

G1 vs G3: We consider using the following variables to produce the Cox proportional hazards fit of η_1 and η_2: an indicator for baseline HIV status, a binary indicator of being enrolled in South Africa, a binary indicator of age being less than or equal to 40 years, sex assigned at birth, a binary indicator of BMI being less than or equal to 25, an interaction term between the BMI and sex indicators, and a categorical variable indicating the enrollment period, and a binary indicator of tuberculosis at baseline. The logistic regression fit of π is produced using the same variables, aside from baseline HIV status.

G2-I vs G4-I: The same adjustments are used as for the comparison of G1 vs G3.

G2-I vs G2-II: The same adjustments are used as for the G1 vs G2-I analysis with parametric modeling and treatment of the covariates as binary variables.

G1 vs G2-II: The same adjustments are used as for the G1 vs G2-I analysis with parametric modeling and treatment of the covariates as binary variables.

G3 vs G4-I: The same adjustments are used as in the primary analysis that compares G1 vs G2-I with parametric modeling and treatment of the covariates as binary variables, excluding CD4 count and HIV viral load.

G4-I vs G4-II: The same adjustments are used as in the primary analysis that compares G1 vs G2-I with parametric modeling and treatment of the covariates as binary variables, excluding CD4 count and HIV viral load.

G3 vs G4-II: The same adjustments are used as in the primary analysis that compares G1 vs G2-I with parametric modeling and treatment of the covariates as binary variables, excluding CD4 count and HIV viral load.

Strategy for selecting the subset of covariates to be used as adjustments

First, we exclude covariates where, among the relevant sub-group of the study population for a given between-group comparison, (1) there are fewer than 5 events per category of the covariate, and (2) under 10% of the participants in some category belongs to one of the comparison groups. Only for the purposes of this screening, enrollment period is treated as a binary indicator enrollment Dec-Apr.

We then rank the remaining covariates by strength of marginal association with the endpoint. To measure strength of marginal association, we fit a set of Cox regression models, where each model treats the endpoint as the time-to-event outcome and a single covariate as a predictor. The covariates are then ranked from highest to lowest by the absolute value of the log-hazard ratio. If there are fewer than 5 events per main comparison group, we perform no covariate adjustment. If there are at least 5 events per main comparison group, we adjust for the covariate that has the strongest marginal association with the endpoint. If there are at least 10 events per main comparison group, we adjust for the the two covariates that have the strongest marginal association with the endpoint, and so on.

Figures and tables to be presented

For each exploratory comparison, the figures and tables described in the beginning of Section 7.3 are produced under each of the following settings:

1. Two origin time points (1 & 2) described in Section 5
2. Two endpoint definitions described in Section 4
3. One covariate adjustment strategy – adjusting for binary versions of the covariates and parametric modeling of the nuisance parameters.

7.4 Cox modeling approach

Advantages of the Cox modeling approach include (1) automatically includes all follow-up without somewhat tricky choices about the last time point t_0 for cumulative failure time analyses; (2) stable modeling approach in the presence of small numbers of events; (3) we have good *a priori* understanding of how this method should behave; in addition for the calendar-time scale analyses (4) it advantageously accommodates scenarios where the baseline hazard depends strongly and unpredictably on calendar time. Its disadvantages are the usual ones with a strong parametric modeling assumption, including that quantifying uncertainty with a 95% confidence interval about the conditional hazard ratio communicates more precision than we actually have. PH assumptions will be assessed using the Schoenfeld residuals (Grambsch & Therneau, 1994).

7.4.1 Primary comparison

The primary comparison compares G1 and G2-I. To achieve this within a calendar-time-scale Cox regression framework, we consider the following two time origins and associated analyses, which correspond to Scenarios 3 and 4 defined in Section 5.

Time origin I. Time origin of participant i equals the number of days between the date of participant i's first dose and the date of the first dose of the first participant. The first vaccination is concurrent with enrollment; hence, the analysis under this time origin framework will be conducted on the full analysis set (FAS) cohort. Adapting the notation in Section 6, for a dichotomized A ($A = 1$ if a participant belongs to G2-I and 0 if in G1), the Cox model in this setting is written as

$$\lambda_{ji}(t \mid A_i, W_i) = \lambda_{0j}(t) \exp\left\{\beta A_i + \gamma^T W_i\right\} I(\tau_i^{\text{enroll}} > t), \tag{8}$$

where τ_i^{enroll} is the enrollment and first vaccination time of a participant i. We will report the parameter $\exp(\beta)$ which measures the hazard ratio conditional on W in the G1 vs. G2-I analysis groups.

We will conduct the Cox regression analysis with the following covariate adjustment strategies:

- Covariates W consist of participant i's age (≤ 40 or not), sex assigned at birth (Female or Male), BMI (≤ 25 or not), interaction of sex assigned at birth and BMI indicator, a binary indicator of tuberculosis at baseline, CD4+ cell counts (≤ 500 or not), and viral load (detected or not). Baseline hazard function $\lambda_{0j}(t)$ will be estimated within geographic regions (RSA or outside RSA).

- Covariates W consist of participant i's age, sex assigned at birth, BMI, interaction of sex assigned at birth and BMI, a binary indicator of tuberculosis at baseline, CD4+ cell counts, and viral load in the $\log_{10}$-scale. Continuous variables age, BMI by sex at birth, CD4+ cell counts, and $\log_{10}$-scale viral load will be modeled flexibly using cubic splines. Baseline hazard function $\lambda_{0j}(t)$ will be estimated within geographic regions (RSA or outside RSA).

The first covariate adjustment strategy is the main approach, and the second covariate adjustment strategy is a sensitivity analysis.

The parameter $\exp(\beta)$ in model (8) measures the association between baseline SARS-CoV-2 status (naïve versus POCS positive) and instantaneous risk of the study endpoint for participants in Group 2-I compared to those in Group 1.

We will repeat analyses outlined above with a continuous anti-Spike antibody marker level S_0 within the $A = 1$ stratum (this analysis will be done following a future version of the SAP).

Time origin II. Let τ_0 denote the calendar time when the first Group 1 participant completed the second dose or the first Group 2-I participant completed the first dose (whichever came first). For a participant in Group 1, the time origin equals the number of days between 13 days after the participant's second dose and τ_0. For a participant in Group 2-I, the time origin equals the number of days between 13 days after the participant's first dose and τ_0. At any calendar time t, the risk set consists of Group 1 participants who are at least 14 days post their second pre-Month-6 vaccination and Group 2-I participants who are at least 14 days post their first (and only) pre-Month-6 vaccination. The analysis will be restricted to the per-protocol cohort, although a participant could be in the per-protocol cohort but not in the risk set at time t if the participant is in Group 1, having received the second vaccination but not reached Day 14 post second vaccination by time t.

Similar to time origin I, we consider the following Cox regression models:

$$\lambda_{ji}(t \mid A_i, W_i) = \lambda_{0j}(t) \exp\left\{\beta A_i + \gamma^T W_i\right\} I(\tau_i^{\mathrm{D14}} > t), \tag{9}$$

where τ_i^{D14} is Day 14 post second vaccination for $A_i = 0$ and Day 14 post first vaccination for $A_i = 1$. When analyzing data under model (9), we will report $\exp(\beta)$ which measures the hazard ratio conditional on baseline covariates W when comparing the Group 2-I participants 14 days after their first vaccination to the Group 1 participants 14 days after their second vaccination.

We will conduct analysis according to time origin II using the same covariate adjustment strategies as in time origin I for a dichotomized A. We will repeat analyses with a continuous anti-Spike antibody marker level S_0 within the $A = 1$ stratum (this analysis will be done following a future version of the SAP).

7.4.2 Secondary comparison

The secondary comparison compares Group 1 and Group 3 combined versus Group 2-I and Group 4-I combined, and investigates the association between instantaneous risk and baseline SARS-CoV-2 status (naïve versus POCS positive). We will consider the same two time origins as described in

Section 7.4.1. Within each time frame, we will conduct the analysis with the following covariate adjustment strategies:

- Covariates W consist of participant i's age (≤ 40 or not), sex assigned at birth (Female or Male), BMI (≤ 25 or not), interaction of sex assigned at birth and BMI indicator, a binary indicator of tuberculosis at baseline, and HIV status. Baseline hazard function $\lambda_{0j}(t)$ will be estimated within geographic regions (RSA or outside RSA).
- Covariates W consist of participant i's age, sex assigned at birth, BMI, interaction of sex assigned at birth and BMI, a binary indicator of tuberculosis at baseline, and HIV status. Continuous variables age and BMI by sex at birth will be modeled flexibly using cubic splines. Baseline hazard function $\lambda_{0j}(t)$ will be estimated within geographic regions (RSA or outside RSA).

The first covariate adjustment is the primary approach and the second is a sensitivity analysis.

7.4.3 Exploratory comparisons

For the exploratory comparison between Group 3 (HIV seronegative and SARS-CoV-2 naïve) and Group 4-I (HIV seronegative and SARS-CoV-2 non-naïve), the same time origin frameworks as described in Section 7.4.1 will be applied to investigating the association between baseline SARS-CoV-2 status (naïve versus POCS positive) and instantaneous risk among HIV seronegative participants. We will not adjust for baseline covariates if the number of endpoints is fewer than 10, and only adjust for binary indicators of age, sex, BMI, and a binary indicator of tuberculosis at baseline as outlined in Section 7.4.1 otherwise.

For the exploratory comparison between Group 1 (PLWH and SARS-CoV-2 naïve) and Group 3 (HIV seronegative and SARS-CoV-2 naïve), the same time origin frameworks will be applied. Let $H_i = 1$ if participant i is HIV seropositive at baseline and 0 otherwise. We will consider the following Cox regression models:

$$\lambda_{ji}(t \mid H_i, W_i) = \lambda_{0j}(t) \exp\left\{\beta H_i + \gamma^T W_i\right\} I(\tau_i^{\text{enroll}} > t), \tag{10}$$

$$\lambda_{ji}(t \mid H_i, W_i) = \lambda_{0j}(t) \exp\left\{\beta H_i + \beta_1 Z_i(t) + \beta_2 H_i Z_i(t) + \gamma^T W_i\right\} I(\tau_i^{\text{enroll}} > t), \tag{11}$$

where $Z_i(t) = 1$ if a participant has received a second vaccination by time t and 0 otherwise, and

$$\lambda_{ji}(t \mid H_i, W_i) = \lambda_{0j}(t) \exp\left\{\beta H_i + \gamma^T W_i\right\} I(\tau_i^{\text{D14}} > t). \tag{12}$$

In model (12), by entering the risk set, a participant has to have two vaccinations. We will not adjust for baseline covariates if the number of endpoints is fewer than 10, and only adjust for binary indicators of age, sex, BMI, and a binary indicator of tuberculosis at baseline as outlined in Section 7.4.1 otherwise.

For the exploratory comparison between Group 2-I (PLWH and POCS positive) and Group 4-I (HIV seronegative and POCS positive), we will consider the following Cox regression models:

$$\lambda_{ji}(t \mid H_i, W_i) = \lambda_{0j}(t) \exp\left\{\beta H_i + \gamma^T W_i\right\} I(\tau_i^{\text{enroll}} > t), \tag{13}$$

and

$$\lambda_{ji}(t \mid H_i, W_i) = \lambda_{0j}(t) \exp\left\{\beta H_i + \gamma^T W_i\right\} I(\tau_i^{\mathrm{D14}} > t). \tag{14}$$

We will not adjust for baseline covariates if the number of endpoints is fewer than 10 and only adjust for binary indicators of age, sex, BMI, and a binary indicator of tuberculosis at baseline as outlined in Section 7.4.1 otherwise.

For the exploratory comparison between Group 2-I (PLWH and POCS positive) and Group 2-II (PLWH and POCS negative but CLS or NAAT positive), we will consider Cox regression models analogous to model (8) and model (9), except that $A_i = 1$ now denotes participant i in Group 2-II and 0 otherwise. We will not adjust for baseline covariates if the number of endpoints is fewer than 10 and adjust for binary indicators of age, sex, BMI, tuberculosis at baseline, CD4 counts, and viral load as outlined in Section 7.4.1 otherwise. The exploratory comparison between Group 4-I (HIV-negative and POCS positive) and Group 4-II (HIV-negative and POCS negative but CLS or NAAT positive) is analogous.

For the exploratory comparison between Group 1 (PLWH and naïve) and Group 2-II (PLWH and POCS negative but CLS or NAAT positive), we will consider Cox regression models analogous to model (8) and model (9), except that $A_i = 1$ now denotes participant i in Group 2-II and 0 otherwise. We will not adjust for baseline covariates if the number of endpoints is fewer than 10 and only adjust for binary indicators of age, sex, BMI, tuberculosis at baseline, CD4 counts, and viral load as outlined in Section 7.4.1 otherwise. The exploratory comparison between Group 3 (HIV-negative and naïve) and Group 4-II (HIV-negative and POCS negative but CLS or NAAT positive) is analogous.

8 Primary versus sensitivity analyses

For pre-M6 data analysis, the FAS cohort analyses are primary. For primary and secondary comparisons defined in Section 3, a single covariate adjustment approach is pre-specified as the primary analysis for these FAS analyses, as well as for the supportive per-protocol analyses. For the primary and secondary comparisons, analyses with the other covariate-adjustment strategies are sensitivity analyses. For the exploratory comparisons, the pre-specified method adjusting for dichotomized versions of covariates will be performed. For the sensitivity analyses, the 95% CIs provide information on the precision of results. Only p-values from the single pre-specified covariate adjustment strategy for the main analysis are to be used for assessing statistical significance. In any analysis, if inferences are unstable, as indicated by exceptionally large standard error estimates, alternative covariate adjustment analysis may be considered. All deviations from the pre-specified analysis will be indicated in resulting statistical reports and manuscripts.

9 Multiplicity adjustment

For exploratory comparisons, point estimates, 95% CIs and nominal p-values will be displayed in forest plots without multiplicity adjustment. No multiple hypothesis testing will be done in any described analyses.

10 Handling missing data

A small number (about 5%) of PLWH participants are missing CD4 cell count and/or HIV-1 viral load at enrollment. In order to adjust for CD4 cell count and HIV VL in analyses and retain these participants, hard-imputation is used to assign a fixed CD4 cell count value and VL value for each of the participants with missing data. Specifically, within each study group (Group 1 or 2), baseline HIV anti-retroviral therapy (ART) status (Yes, No or Missing) and Sex (Female or Male), missing CD4 cell count is imputed to be the median CD4 count of those with observed data. The same imputation is done for missing VL. Missing data of other baseline characteristics, including BMI ($< 1\%$ with missing data) are imputed by the median value of the variable in question of the overall FAS study population with observed data.

11 Sensitivity analyses of primary and secondary comparisons to unmeasured confounders

E-values for the point estimate and the upper 95% confidence limit will be computed for the Cox model analyses Ding and VanderWeele (2016), to quantify robustness of the results to unmeasured confounding after accounting for baseline covariates in the analysis.

12 Persistent NAAT positivity

To monitor SARS-CoV-2 viral persistence and evolution, nasal swabs are collected from all study participants and are tested for SARS-CoV-2 infection via NAAT at baseline (screening or M0 vaccination visit), at each subsequent vaccination visit (M1, if applicable, and M6), and every two weeks after SARS-CoV-2 infection diagnosis until a negative nasal swab test result is observed. For the final analysis of data collected in the Pre-M6 stage, NAAT test results will be included up till the Month 6 vaccination visit date or the pre-Month 6 stage data cutoff date (March 31, 2023), whichever occurred earlier for each participant.

12.1 Descriptive analysis including all baseline and post-baseline NAAT positive infections

In this analysis, NAAT positivity pertaining to the first occurrence of NAAT-confirmed SARS-CoV-2 infection (regardless of symptomology), including those diagnosed at baseline, is considered. The duration of NAAT positivity for the given infection stops at the first negative NAAT visit after the last NAAT positive prior to a new infection. A new infection is implied if, for a given participant, either a) there are two consecutive negative NAAT results between positive swabs, or b) a positive NAAT result is found at least 90 days after a single negative swab. Future analyses incorporating sequence information may also consider multiple occurrences.

The number and percentage of these infections out of FAS participants will be summarized by analysis group. The number and percentage of these infections by the duration of persistent NAAT positivity ≥ 25, 50 or 100 days will also be summarized overall, by analysis group and by other subgroups of interest (e.g., HIV status). The duration of persistent NAAT positivity is defined as

the number of days between the first and last NAAT positive results. The interval of 25 days is to account for the left visit window of the M1 vaccination. The number and percentage of infections with the duration of persistent NAAT positivity $\geq$ 25, 50 or 100 days will also be calculated among those participants with at least 2 NAAT test results as the denominator; these numbers are more relevant because participants with only a single NAAT positive test result do not have available information on their NAAT positivity persistence. In other words, this second approach requires a participant to have some evaluability for NAAT positive persistency.

12.2 Subgroup analyses pertaining to symptomatic infections diagnosed post baseline

In this analysis, NAAT results associated with the (post-baseline) symptomatic infection diagnosis is considered, including pre-symptomatic NAAT positive test results prior to the diagnosis.

The number and percentage of these infections by the duration of persistent NAAT positivity $\geq$ 25, 50 or 100 days will be summarized overall, by analysis group and by other subgroups of interest (e.g., HIV status). The number and percentage of infections with the duration of persistent NAAT positivity $\geq$ 25, 50 or 100 days will also be calculated among those participants with at least 2 NAAT test results as the denominator.

12.3 Regression modeling of NAAT persistence

The following analysis will be performed: 1) among participants with at least one baseline or post-baseline NAAT test positive result regardless of symptomology and 2) among participants with symptomatic infections diagnosed post baseline. Similar analyses may also be performed restricting to participants with at least 2 NAAT test results.

Let T denote time (in days) between the first positive (FP) and the first negative (FN) NAAT dates, assuming hypothetically a daily NAAT testing procedure with no measurement error (i.e., no inter-current blips of negative results followed by a positive test result in the absence of a new infection). Because NAAT testing after a positive NAAT may not be done or available at the time of analysis, the observed data of T in this study contain a mixture of left-, interval- and/or right-censored observations, or so-called partly interval-censored data in the literature. In addition, given the nature of the data, both the time-origin and the endpoint of T are partly interval-censored. However, since we typically do not observe the last negative (LN) NAAT test date prior to the time origin, or the LN date would be at least 1 to 4 months prior to the FP, we consider T as singly-censored at the endpoint. Consequently, the singly-censored T is a lower bound for the failure time of true interest. In future exploratory analyses, we may consider incorporating NAAT test results prior to the FP date, and consider T as doubly partly interval-censored.

Let (L, R] denote the smallest observed interval that brackets T, where L is the time (in days) of the last positive (LP) test result since the time origin, and R is the time (in days) of the first negative (FN) test result since the time origin. In the analysis cohort of participants with at least 1 NAAT positive test result, when the last NAAT test after the time origin is negative, i.e., $0 \leq L < R < \infty$, T is interval-censored with L and R defined above. When the last NAAT test after the time-origin is positive, i.e., $R = \infty$, T is right-censored at $L + 1$ day(s). As a special case, when the first NAAT positive test is the only observed NAAT test, i.e., $L = 0$ and $R = \infty$, T is right-censored at 1

day. In the analysis cohort of participants with at least 2 NAAT test results, T will also be either right-censored or interval censored, but with L always > 0.

Quantile regression models (Frumento (2022)) that account for such partly interval censored data will be used to estimate the conditional distribution function of persistent NAAT positivity, T, among participants with at least 1 positive NAAT test result using the *ctqr()* function in the ctqr R package. The effect of baseline characteristics (e.g., HIV-1 sero-status, concomitant TB status, HIV-1 VL among PLWH ($\geq$ vs. $<$ 50 copies/mL), and CD4 count among PLWH ($\leq$ vs. $<$ 200 cells/mm^3) may also be estimated at different quantiles, especially at the median. For implementation in *ctqr()*, the following describes the specifications of the L and R input arguments of the function: 1) when the FN test result is observed after the FP time-origin, the left interval, or L ($time1$ argument in the function) is defined as the difference between the LP and FP dates, and the right interval or R ($time2$ argument in the function) is defined as the difference between the FN and the FP time-origin (i.e., L); 2) when the FN test result is not observed at the time of analysis, $time1$ is defined as 1 day plus the difference between the LP and FP dates, and $time2$ is set to infinity to indicate T being right-censored at $time1$. As a special case, $time1 = 1$ and $time2 =$*Inf* when the FP test date is the only observed NAAT test date (i.e. FP==LP). The conditional distribution function is computed using the *pchreg* function of the pch R package. Similar to the descriptive analyses, quantile regression models may also be applied to the analysis of participants with at least 2 NAAT test results pending on data availability at the time of the analysis.

References

Ding, P. and VanderWeele, T. (2016), "Sensitivity analysis without assumptions," *Epidemiology*, 27(3), 368.

Frumento, P. (2022), "A quantile regression estimator for interval-censored data," *Int J Biostat*.

Westling, T., Luedtke, A., Gilbert, P. B., and Carone, M. (2023), "Inference for treatment-specific survival curves using machine learning," *Journal of the American Statistical Association*, 1–26.

From: Gilbert PhD, Peter B
To: Kee, Jia Jin
Subject: RE: CoVPN3008 final pre-Month 6 stage efficacy analysis SAP version 2.0 signoff
Date: Monday, July 10, 2023 9:59:08 AM

I, Peter Gilbert, PI of the HVTN SDMC, approve UbuntuP3008primaryefficacySAP_V2.0.pdf, Statistical Analysis Plan for Comparing COVID-19 Risk in Ubuntu/CoVPN 3008: First Stage (Follow-up Through Month 6 Booster Dose) Final Analysis (Version 2.0). This email is a substitute for a handwritten signature.

Best regards,

Peter Gilbert

**From:**	Huang, Yunda
**To:**	Kee, Jia Jin
**Subject:**	RE: CoVPN3008 final pre-Month 6 stage efficacy analysis SAP version 2.0 signoff
**Date:**	Saturday, July 15, 2023 1:54:21 AM

I, Yunda Huang, Principal Staff Scientist, approve UbuntuP3008primaryefficacySAP_V2.0.pdf, Statistical Analysis Plan for Comparing COVID-19 Risk in Ubuntu/CoVPN 3008: First Stage (Follow-up Through Month 6 Booster Dose) Final Analysis (Version 2.0). This email is a substitute for a handwritten signature.

From: Kee, Jia Jin <jkee2@scharp.org>
Sent: Monday, July 10, 2023 9:59 AM
To: Huang, Yunda <yunda@scharp.org>
Subject: CoVPN3008 final pre-Month 6 stage efficacy analysis SAP version 2.0 signoff

Hi Yunda,

If you approve the attached pre-Month 6 stage efficacy analysis SAP version 2.0 of CoVPN 3008, please respond with the following statement:

I, <name and title>, approve UbuntuP3008primaryefficacySAP_V2.0.pdf, Statistical Analysis Plan for Comparing COVID-19 Risk in Ubuntu/CoVPN 3008: First Stage (Follow-up Through Month 6 Booster Dose) Final Analysis (Version 2.0). This email is a substitute for a handwritten signature.

Thanks,
Jin

Jin Kee
Pronouns: She/They
Statistical Research Associate
Statistical Center for HIV/AIDS Research & Prevention (SCHARP)
Fred Hutchinson Cancer Center
jkee2@scharp.org

From: Hudson, Aaron
To: Kee, Jia Jin
Subject: Re: CoVPN3008 final pre-Month 6 stage efficacy analysis SAP version 2.0 signoff
Date: Monday, July 10, 2023 12:40:08 PM

Hi Jin,

I, Aaron Hudson, Assistant Professor, BBE, VIDD, approve UbuntuP3008primaryefficacySAP_V2.0.pdf, Statistical Analysis Plan for Comparing COVID-19 Risk in Ubuntu/CoVPN 3008: First Stage (Follow-up Through Month 6 Booster Dose) Final Analysis (Version 2.0). This email is a substitute for a handwritten signature.

Thanks,
Aaron

Aaron Hudson
(he/him/his)
Assistant Professor
Biostatistics, Bioinformatics, and Epidemiology Program
Vaccine and Infectious Disease Division
Fred Hutchinson Cancer Center

From: Kee, Jia Jin <jkee2@scharp.org>
Sent: Monday, July 10, 2023 9:58 AM
To: Hudson, Aaron <ahudson@fredhutch.org>
Subject: CoVPN3008 final pre-Month 6 stage efficacy analysis SAP version 2.0 signoff

Hi Aaron,

If you approve the attached pre-Month 6 stage efficacy analysis SAP version 2.0 of CoVPN 3008, please respond with the following statement:

I, <name and title>, approve UbuntuP3008primaryefficacySAP_V2.0.pdf, Statistical Analysis Plan for Comparing COVID-19 Risk in Ubuntu/CoVPN 3008: First Stage (Follow-up Through Month 6 Booster Dose) Final Analysis (Version 2.0). This email is a substitute for a handwritten signature.

Thanks,
Jin

Jin Kee
Pronouns: She/They
Statistical Research Associate
Statistical Center for HIV/AIDS Research & Prevention (SCHARP)
Fred Hutchinson Cancer Center

**From:**	Zhang, Bo
**To:**	Kee, Jia Jin
**Subject:**	Re: CoVPN3008 final pre-Month 6 stage efficacy analysis SAP version 2.0 signoff
**Date:**	Monday, July 10, 2023 10:14:15 AM

I, Bo Zhang, Assistant Professor of Biostatistics, approve UbuntuP3008primaryefficacySAP_V2.0.pdf, Statistical Analysis Plan for Comparing COVID-19 Risk in Ubuntu/CoVPN 3008: First Stage (Follow-up Through Month 6 Booster Dose) Final Analysis (Version 2.0). This email is a substitute for a handwritten signature.

From: Kee, Jia Jin <jkee2@scharp.org>
Sent: Monday, July 10, 2023 9:58 AM
To: Zhang, Bo <bzhang3@fredhutch.org>
Subject: CoVPN3008 final pre-Month 6 stage efficacy analysis SAP version 2.0 signoff

Hi Bo,

If you approve the attached pre-Month 6 stage efficacy analysis SAP version 2.0 of CoVPN 3008, please respond with the following statement:

I, <name and title>, approve UbuntuP3008primaryefficacySAP_V2.0.pdf, Statistical Analysis Plan for Comparing COVID-19 Risk in Ubuntu/CoVPN 3008: First Stage (Follow-up Through Month 6 Booster Dose) Final Analysis (Version 2.0). This email is a substitute for a handwritten signature.

Thanks,
Jin

Jin Kee
Pronouns: She/They
Statistical Research Associate
Statistical Center for HIV/AIDS Research & Prevention (SCHARP)
Fred Hutchinson Cancer Center
jkee2@scharp.org
