# Supplementary material for: Hybrid versus vaccine immunity of mRNA-1273 among people living with HIV in East and Southern Africa: a prospective cohort analysis from the multicentre CoVPN 3008 (Ubuntu) study
Source: eClinicalMedicine. 2025 Jan 20;80:103054. doi: 10.1016/j.eclinm.2024.103054 (PMC11788791; doi:10.1016/j.eclinm.2024.103054)
Supplement: Supplemental Materials [file mmc3.pdf]

Supplemental Materials

Table of Contents

Glossary ... 4

Study Team ... 5

Supplemental Methods ... 7

Trial Oversight ... 7

Participants and Procedures ... 7

Trial Vaccine ... 7

Safety Assessments ... 7

Post-SARS-CoV-2 Infection Diagnosis Schedule ... 7

Covid-19 Assessment ... 7

Data Management ... 8

SARS-CoV-2 Serology Assays ... 8

Determination of Viral Lineage ... 8

Statistical Analysis ... 9

Analysis of NAAT-persistence ... 11

Supplemental Tables ... 12

Table S1. Full eligibility criteria. ... 12

Table S2. Covid-19 and severe Covid-19 definitions ... 13

Table S3. Enrolment by country and HIV status, Full Analysis Set ... 14

Table S4. Baseline demographic and clinical characteristics of the Safety Subset by the six analysis groups. ... 15

Table S5. Summary of participant comorbidities, other than HIV, associated with increased risk of severe Covid-19, as reported at screening by ≥ 1% of participants, Full Analysis Set ... 18

Table S6. Solicited adverse events within 7 days after month 0 vaccination by grade in the Safety Subset. ... 20

Table S7. Solicited adverse events within 7 days after month 1 vaccination by grade in the Safety Subset. ... 23

Table S8. Unsolicited adverse events within 28 days after month 0 or month 1 vaccination in the Safety Subset. ... 25

Table S9. Summary of unsolicited adverse events reported by ≥1% of participants within 28 days after month 0 or month 1 vaccination in the Safety Subset. ... 26

Table S10. Serious adverse events up till month 6 visit reported by preferred term in the Full Analysis Set. ... 27

Table S11. Pregnancy outcomes up till month 6 visit in the Full Analysis Set. .. **Error! Bookmark not defined.**

Table S12. Number and incidence rates of Covid-19 endpoints based on the CDC case definition, including or excluding endpoints associated with NAAT results obtained at the month 1 vaccination

visit, and the number and incidence rates of severe Covid-19 endpoints in the Full Analysis Set and the Per-Protocol cohort by analysis group..37

Table S13. Number and incidence rates of Covid-19 endpoints based on the COVE case definition, including or excluding endpoints associated with NAAT results obtained at the month 1 vaccination visit in the Full Analysis Set and the Per-Protocol cohort by analysis group....................................38

Table S14. Geographic distribution of identified subvariants of SARS-CoV-2 infections from nasal swabs collected at baseline and post-baseline visits throughout the study. 39

Table S15. Number of baseline and post-baseline first occurrence of NAAT positive results, including or excluding those obtained at the month 1 vaccination visit, in the Full Analysis Set by the analysis group...40

Figure S1. Ubuntu CoVPN 3008 study flowcharts 41

Figure S2. Time to early termination by analysis group...43

Figure S3. Solicited local and systemic adverse events. 45

Figure S4. Hybrid and vaccine immunity cumulative incidence of severe Covid-19 among people living with HIV in the Full Analysis Set...46

Figure S5. Number of SARS-CoV-2 nucleic acid amplification tests performed over study time........47

Figure S6. Association of hybrid versus vaccine immunity with SARS-CoV-2 nucleic acid amplification test positivity in the Full Analysis Set. 48

Figure S7. Association of hybrid versus vaccine immunity with Covid-19 (COVE) in the Full Analysis Set..49

Figure S8. Association of hybrid versus vaccine immunity with Covid-19 (COVE) in the Per-Protocol cohort. 51

Figure S9. Association of hybrid versus vaccine immunity with Covid-19 (CDC) based on cumulative incidence analysis in the Full Analysis Set and the Per-Protocol cohort..53

Figure S10. Association of hybrid versus vaccine immunity with Covid-19 (COVE) based on cumulative incidence analysis in the Full Analysis Set and the Per-Protocol cohort. 55

Figure S11. Association of hybrid versus vaccine immunity with Covid-19 (CDC) over time based on cumulative incidence analysis in the Full Analysis Set and the Per-Protocol cohort. 57

Figure S12. Association of hybrid versus vaccine immunity with Covid-19 (COVE) over time based on cumulative incidence analysis in the Full Analysis Set and the Per-Protocol cohort. 58

Figure S13. Sensitivity analysis of Cox proportional hazards results with alternative covariate adjustment strategies. 59

Figure S14. Sensitivity analysis of cumulative incidence results with alternative covariate adjustment strategies..60

Figure S15. Association of hybrid versus vaccine immunity with the Covid-19 (CDC) over time based on cumulative incidence analysis and the secondary comparison pooling over people living with HIV and people living without HIV in the Full Analysis Set and the Per-Protocol cohort........................61

Figure S16. Hybrid and vaccine immunity cumulative incidence of severe Covid-19 for the secondary comparison pooling over people living with HIV and people living without HIV in the Full Analysis Set and the Per-Protocol cohort...62

Figure S17. Hybrid and vaccine immunity cumulative incidence of Covid-19 (COVE) for the secondary comparison pooling over people living with HIV and people living without HIV. 64

Figure S18. Association of hybrid versus vaccine immunity with Covid-19 (COVE) over time based on cumulative incidence analysis and the secondary comparison pooling over people living with HIV and people living without HIV in the Full Analysis Set and the Per-Protocol cohort. 66

Figure S19. Seven exploratory comparisons of the risk of Covid-19 (CDC) based on the Cox models in the Full Analysis Set and the Per-Protocol cohort. 67

Figure S20. Comparison of cumulative incidence of Covid-19 (CDC) between exploratory comparison groups HIV-, SARS-CoV-2-, 2 doses versus HIV+, SARS-CoV-2+, 1 dose. 69

Figure S21. Comparison of cumulative incidence of Covid-19 (CDC) between exploratory comparison groups HIV+, SARS-CoV-2-, 2 doses versus HIV-, SARS-CoV-2-, 2 doses. 70

Figure S22. Comparison of cumulative incidence of Covid-19 (CDC) between exploratory comparison groups HIV+, SARS-CoV-2+, 1 dose versus HIV-, SARS-CoV-2+, 1 dose.....................................71

Figure S23. Comparison of cumulative incidence of Covid-19 (CDC) between exploratory comparison groups HIV+, SARS-CoV-2+, 1 dose versus HIV+, SARS-CoV-2+, 2 doses. 72

Figure S24. Comparison of cumulative incidence of Covid-19 (CDC) between exploratory comparison groups HIV-, SARS-CoV-2+, 1 dose versus HIV-, SARS-CoV-2+, 2 doses. 73

Figure S25. Comparison of cumulative incidence of Covid-19 (CDC) between exploratory comparison groups HIV+, SARS-CoV-2-, 2 doses versus HIV+, SARS-CoV-2+, 2 doses.................................74

Figure S26. Comparison of cumulative incidence of Covid-19 (CDC) between exploratory comparison groups HIV-, SARS-CoV-2-, 2 doses versus HIV-, SARS-CoV-2+, 2 doses. 75

Figure S27. Comparison of cumulative incidence of Covid-19 (COVE) between exploratory comparison groups HIV-, SARS-CoV-2-, 2 doses versus HIV-, SARS-CoV-2+, 1 dose. 76

Figure S28. Comparison of cumulative incidence of Covid-19 (COVE) between exploratory comparison groups HIV+, SARS-CoV-2-, 2 doses versus HIV-, SARS-CoV-2-, 2 doses. 77

Figure S29. Comparison of cumulative incidence of Covid-19 (COVE) between exploratory comparison groups HIV+, SARS-CoV-2+, 1 dose versus HIV-, SARS-CoV-2+, 1 dose. 78

Figure S30. Comparison of cumulative incidence of Covid-19 (COVE) between exploratory comparison groups HIV+, SARS-CoV-2+, 1 dose versus HIV+, SARS-CoV-2+, 2 doses. 79

Figure S31. Comparison of cumulative incidence of Covid-19 (COVE) between exploratory comparison groups HIV-, SARS-CoV-2+, 1 dose versus HIV-, SARS-CoV-2+, 2 doses. 80

Figure S32. Comparison of cumulative incidence of Covid-19 (COVE) between exploratory comparison groups HIV+, SARS-CoV-2-, 2 doses versus HIV+, SARS-CoV-2+, 2 doses. 81

Figure S33. Comparison of cumulative incidence of Covid-19 (COVE) between exploratory comparison groups HIV-, SARS-CoV-2-, 2 doses versus HIV-, SARS-CoV-2+, 2 doses. 82

Figure S34. Association of CD4 count (< vs. ≥ 350 cells/µl) and HIV viremia (< vs. ≥ 50 copies/ml) with risk of Covid-19 in people living with HIV, within strata defined by hybrid and vaccine immunity. 83

Figure S35. Lineages of SARS-CoV-2 associated with diagnosis of Covid-19 (CDC) by analysis group in the Full Analysis Set. 84

Figure S36. Lineages of SARS-CoV-2 associated with diagnosis of Covid-19 (COVE) by analysis group in the Full Analysis Set. 86

Figure S37. Lineages of all baseline and post-baseline positive nucleic acid amplification tests by analysis group in the Full Analysis Set. 87

References 89

Glossary

AE	Adverse event
AG	Analysis group
Anti-NP	anti-nucleoprotein serology
AoU	Assessment of Understanding
ARDS	Acute Respiratory Distress Syndrome
ART	Antiretroviral therapy
BARC SA	Bio Analytical Research Corporation South Africa
BARDA	Biomedical Advanced Research and Development Authority
BMI	Body mass index
BP	Blood pressure
CDC	US Center for Disease Control and Prevention
CERI	Center for Epidemic Response and Innovation
Covid-19	Coronavirus disease of 2019
CoVPN	COVID-19 Prevention Network
CRF	Case report form
D	Dose
DAIDS	Division of AIDS
ECMO	Extracorporeal membrane oxygenation
FAS	Full analysis set
FIO_2	Fraction of Inspired Oxygen
HIV	Human immunodeficiency virus
HR	Hazard ratio
ICU	Intensive care unit
KRISP	KwaZulu-Natal Research and Innovation Sequencing Platform
NAAT	Nucleic acid amplification test
NIAID	National Institute of Allergy and Infectious Diseases
NP	Nucleoprotein
PaO_2	Partial pressure of oxygen
PBMC	Peripheral blood mononuclear cells
PLWH	People living with HIV
PLWoH	People living without HIV
POC anti-S	Point-of-care anti-spike serology
PP	Per protocol
QC	Quality control
SAE	Serious adverse event
SAMRC	South African Medical Research Council
SARS-CoV-2	Severe acute respiratory syndrome coronavirus 2
SpO_2	Peripheral capillary oxygen saturation
SS	Safety subset
TB	Tuberculosis
VOC	Variant of concern

Study Team

First name	Surname	Organization
Sharlaa	Badal-Faesen	Clinical HIV Research Unit / Helen Joseph CRS
Kagisho	Baepanye	Hutchinson Centre Research Institute of South Africa
Veronique	Bailey	Hutchinson Centre Research Institute of South Africa
Katekani	Baloyi-Oseh	Hutchinson Centre Research Institute of South Africa
Mumtaz	Booley	Hutchinson Centre Research Institute of South Africa
Johannes Louis	Botha	TASK Eden
Yolande	Brown	Hutchinson Centre Research Institute of South Africa
Valerie	Brown	The Emmes Company, LLC
Lisa	Bunts	Fred Hutchinson Cancer Center
Soritha	Coetzer	Synexus Helderberg
Myron	Cohen	University of North Carolina at Chapel Hill
Shirley	Collie	BioInformatiCo
Rodney	Dawson	University of Cape Town Lung Institute CRS
Pallabi	Deb	Fred Hutchinson Cancer Center
Hana	El Sahly	Baylor College of Medicine
Jill	El-Khorazaty	The Emmes Company, LLC
Andries	Engelbrecht	Hutchinson Centre Research Institute of South Africa
Marianne	Gildea	FHI 360
Dhevium	Govender	Hutchinson Centre Research Institute of South Africa
Jen	Hanke	Fred Hutchinson Cancer Center
Jayla	Harris	Fred Hutchinson Cancer Center
Simone	Hendricks	Hutchinson Centre Research Institute of South Africa
Nick	Hopkinson	Fred Hutchinson Cancer Center
Haley	Howell	The Emmes Company, LLC
Nzeera	Ketter	Fred Hutchinson Cancer Center
Kentse	Khuto	Hutchinson Centre Research Institute of South Africa
Faatima	Laher Omar	Hutchinson Centre Research Institute of South Africa
Leolin	Katsidzira	University of Zimbabwe
Kim	Linton	The Emmes Company, LLC
James	Ludwig	Fred Hutchinson Cancer Center
Bongile	Mabilane	Hutchinson Centre Research Institute of South Africa
Matshidiso	Malefo	Hutchinson Centre Research Institute of South Africa
Ndiitwani	Mamushiana	Ndlovu Research Centre
Daciana	Margineantu	Fred Hutchinson Cancer Center
Jeanine	May	The Emmes Company, LLC
Fatima	Mayat	Perinatal HIV Research Unit
Cindy	Molitor	Fred Hutchinson Cancer Center
Yeshnee	Naidoo	KwaZulu-Natal Research Innovation and Sequencing Platform

Michelle	Nebergall	Fred Hutchinson Cancer Center
Alan	Nguyen	Fred Hutchinson Cancer Center
Sarah	Nikles	The Emmes Company, LLC
Bianca	Noronha	The Emmes Company, LLC
Melissa	Peda	Fred Hutchinson Cancer Center
Tamara	Phiri	Queen Elizabeth Central Hospital, Malawi
Shanthie	Pillay	Hutchinson Centre Research Institute of South Africa
Sureshnee	Pillay	KwaZulu-Natal Research Innovation and Sequencing Platform
Lori	Proulx-Burns	Fred Hutchinson Cancer Center
Laurie	Rinn	Fred Hutchinson Cancer Center
Lisa	Sanders	Fred Hutchinson Cancer Center
Carrie	Sopher	Fred Hutchinson Cancer Center
Smitha	Sripathy	Fred Hutchinson Cancer Center
Michael	Stirewalt	Fred Hutchinson Cancer Center
Houriiyah	Tegally	KwaZulu-Natal Research Innovation and Sequencing Platform
Sara	Thiebaud	Fred Hutchinson Cancer Center
Alicia	Toledano	The Emmes Company, LLC
Stephanie	Van Wyk	Centre for Epidemic Response & Innovation
Shamaya	Whitby	Fred Hutchinson Cancer Center
Stephany	Wilcox	Hutchinson Centre Research Institute of South Africa
Eduan	Wilkinson	Centre for Epidemic Response & Innovation
Haven	Wilvich	Fred Hutchinson Cancer Center
Charles	Wiysonge	South African Medical Research Council
Nelisiwe	Xaba	Hutchinson Centre Research Institute of South Africa
Ntokozo	Xulu	Hutchinson Centre Research Institute of South Africa

Supplemental Methods

Trial Oversight

The trial sponsor, the South African Medical Research Council (SAMRC), provided oversight on the trial design, site selection and monitoring, and data analysis, with input from Biomedical Advanced Research and Development Authority (BARDA), the National Institute of Allergy and Infectious Diseases (NIAID), the Covid-19 Prevention Network (CoVPN), and the protocol team.

Participants and Procedures

Site-level enrolment targets were set at 80% people living with HIV (PLWH) and 70% people with evidence of prior SARS-CoV-2 infection.

Trial Vaccine

The mRNA-1273 vaccines were stored long term -50° and -15° C. Unpunctured vials could be stored at 2° to 8° C at clinical sites before preparation and administration for up to 30 days from removal from freezer. Punctured vials were held between 2° and 25° C for up to 12 hours from time of first vial puncture. The injections prepared from punctured vials had to administered within 12 hours from first vial puncture. No dilution was required and doses could be held in syringes for up to 8 hours at room temperature before administration. Vaccines were administered as an intramuscular injection into the deltoid muscle at a dose of 100 mcg of mRNA-1273.

Safety Assessments

All adverse events were graded according to the Division of AIDS (DAIDS) Table for Grading the Severity of Adult and Pediatric Adverse Events, Corrected Version 2.1, July 2017 [1]. The study team supported sites in monitoring PLWH to ensure referral for adherence counselling or optimize treatment as appropriate, in cases of poorly controlled HIV. Pregnant participants continuing their pregnancy were referred for local prenatal care including fetal ultrasound, syphilis testing/treatment, and HIV testing, if not already known to be living with HIV. All pregnancies were followed up for outcomes. Confirmed Covid-19 cases were closely monitored to permit early interventions, as appropriate, to prevent complications, including temperature monitoring, pulse oximetry, provision of oxygen, steroids and other medications, and hospitalization.

Post-SARS-CoV-2 Infection Diagnosis Schedule

All participants with SARS-CoV-2 infection confirmed by nucleic acid amplification test (NAAT), regardless of symptoms or rationale for being tested, entered the same post-diagnosis schedule. During the post-diagnosis time period, participants used daily diaries to record temperature, heart rate, oxygen saturation, and presence and severity of respiratory and systemic symptoms of Covid-19. Daily signs and symptoms were recorded for 14 days or through symptom resolution, whichever was later. An overall severity assessment was completed 28 days following diagnosis. To further address the persistence of NAAT-detectable SARS-CoV2, an update to the post-diagnosis schedule was made beginning in April 2022 to add additional nasal swabs collected at day 14 and day 28 following the initial NAAT-positive swab, with additional swabs collected every 14 days as needed until a negative result was obtained.

Covid-19 Assessment

For the assessment of severe Covid-19 end points, severe symptoms included any of the following:

- Clinical signs indicative of severe systemic illness, respiratory rate ≥ 30 per minute, heart rate ≥ 125 beats per minute, $SpO_2 \leq 93\%$ on room air at sea level or $PaO_2/FIO_2 < 300$ mm Hg, OR
- respiratory failure or Acute Respiratory Distress Syndrome (ARDS, defined as needing high-flow oxygen, non-invasive or mechanical ventilation, or ECMO [extracorporeal membrane oxygenation]),

evidence of shock (systolic blood pressure < 90 mmHg, diastolic blood pressure < 60 mmHg or requiring vasopressors), OR

- Significant acute renal, hepatic or neurologic dysfunction, OR
- Admission to an intensive care unit or death.

Data Management

Clinical case report form (CRF) data were collected in Advantage eClinical® software managed by The Emmes Company, LLC (Rockville, Maryland, USA). Sequencing data were provided by KwaZulu-Natal Research and Innovation Sequencing Platform (KRISP, Durban, South Africa) and the Center for Epidemic Response and Innovation (CERI, Durban, South Africa).

SARS-CoV-2 Serology Assays

The baseline point-of-care anti-spike serology (POC anti-S) testing used to assign Study Group was performed by site staff using the EcoTest IgG/IgM rapid kit. All site staff performing testing were required to document training on proper conduct of the tests. Central lab anti-nucleoprotein (NP) serology (anti-NP) testing was performed using the Abbott Allinity anti-NP IgG assay at the Bio Analytical Research Corporation South Africa Global Central Laboratory (BARC SA, Johannesburg, South Africa).

Determination of Viral Lineage

Because the obtained SARS-CoV-2 sequences were missing a considerable amount of their genomic content (Q1: 3.1% missing; median: 18.8% missing; Q3: 66.1% missing), we used two methods to best determine their viral lineages: PANGOLIN [2] and NextClade [3]. Specifically, we used the quality control (QC) flags for the two platforms to assign the sequences into one of three groups:

1. *High-confidence*: QC passed for both platforms (found in 22.3% of sequences; median genomic missingness = 0.8%)
2. *Moderate-confidence*: QC passed or was "mediocre" for at least one platform (found in 32.1% of sequences; median genomic missingness = 10.1%)
3. *Low-confidence*: QC failed for both platforms or no lineage was obtainable (found in 45.7% of sequences; median genomic missingness = 74.3%)

Using these three confidence categories, we determined the viral lineage of a given sequencing using the following decision tree:

1. *High-confidence calls* (n = 209):
    a. If the lineage calls with both platforms matched, then we used that determined lineage (n = 196).
    b. If the lineage calls did not match, then we defaulted to PANGOLIN's lineage call, as PANGOLIN is regarded as being slightly more accurate than NextClade [4] (n = 13).

2. *Moderate-confidence calls* (n = 301):
    a. If a lineage call was made with both platforms, and those lineage calls matched, then we used that determined lineage (n = 130).
    b. If a lineage call was made with both platforms, but the lineage calls differed and are on the same branch of the tree (e.g., BA.5 vs. BA.5.11), then we accepted the lineage call from the ancestor of the two calls (e.g., BA.5) (n = 53).
    c. If a lineage call was made with both platforms, but the calls differed and are from different branches of the tree (e.g. BA.5.2 vs. BA.5.11) then we selected the most-recent common ancestor from the two lineages (e.g., BA.5) (n = 1).
    d. If a lineage call was made with both platforms, but the calls differ, and one platform failed QC, then we selected the lineage from the platform that didn't fail QC (n = 99).
    e. If a lineage call was only present from one platform, and that platform passed QC, then we accepted that lineage call (n = 18).

3. *Low-confidence calls* (n = 429):
   f. If a lineage call was available from only one platform, we accepted that call but documented it as low confidence (n = 395). Many of these lineage calls are seemingly erroneous as they are of an extinct distant ancestor (e.g., A.1, B, B.1.1), which is likely the result of the available sequence fragment missing key mutations that characterize its true lineage from the ancestral lineage.
   g. If no lineage calls were available from either platform, then we were unable to estimate the lineage for the sequence (n = 34).

Statistical Analysis

Imputation of missing baseline SARS-CoV-2 status

Participants' point-of-care anti-spike serology (POC anti-S) status based on specimens collected prior to baseline and with results available prior to the receipt of their first vaccination was used to determine the number of doses that a participant will receive during the study, and hence the study group they belong to. As specified in the statistical analysis plan, participants' central lab anti-NP and NAAT results based on specimens collected prior to baseline but with results not available until later, were used to define the six analysis groups (**Figure 1**). To account for the correlation between POC anti-S, anti-NP and NAAT results, the following algorithm was applied to impute missing data in anti-NP and NAAT (POC anti-S results were available on all enrolled participants). Specifically, for participants who missed the anti-NP result but had both the POC anti-S and NAAT results, the missing anti-NP result was imputed as positive if either POC anti-S or NAAT was positive and negative if both POC anti-S and NAAT were negative. For participants who missed the NAAT result but had both POC anti-S and anti-NP results, the missing NAAT result was always imputed as negative. For participants who missed both anti-NP and NAAT results, their missing anti-NP test result was imputed as positive if POC anti-S result was positive and negative if the POC anti-S result was negative. Their NAAT result was always imputed as negative. This imputation scheme was motivated by the joint distribution of POC anti-S, anti-NP, and NAAT among those who had complete measurements of test results (> 98% of the study cohort); see Table below for various empirical conditional probabilities. We also considered these conditional probabilities separately in the stratum defined by baseline HIV status; results were qualitatively similar and the derived imputation scheme was the same.

Panel A: Empirical probability that anti-NP or NAAT result was positive conditional on different configurations of the other two test results among 13,934/14,001 participants with all three test results measured. **Panel B:** Empirical probability that anti-NP or NAAT was positive conditional on POC anti-S test result among 13,975/14,001 participants with both anti-NP and POC anti-S test results and among 13,951/14,001 participants with both NAAT and POC anti-S test results, respectively.

**Panel A**	
P(anti-NP = 1 | POC anti-S = 0 & NAAT = 0)	0.29
P(anti-NP = 1 | POC anti-S = 1 & NAAT = 0)	0.58
P(anti-NP = 1 | POC anti-S = 1 & NAAT = 1)	0.81
P(anti-NP = 1 | POC anti-S = 0 & NAAT = 1)	0.60
P(NAAT = 1 | POC anti-S = 0 & anti-NP = 0)	0.04
P(NAAT = 1 | POC anti-S = 1 & anti-NP = 0)	0.02
P(NAAT = 1 | POC anti-S = 1 & anti-NP = 1)	0.06
P(NAAT = 1 | POC anti-S = 0 & anti-NP = 1)	0.14
**Panel B**	
P(anti-NP = 1 | POC anti-S = 0)	0.32
P(anti-NP = 1 | POC anti-S = 1)	0.59
P(anti-NP = 1 | NAAT = 0)	0.08
P(anti-NP = 1 | NAAT = 1)	0.04

Analysis approaches

Two approaches were used to analyze the association between hybrid immunity vs. vaccine immunity with the study endpoints: a calendar-time-scale Cox regression approach and a counterfactual cumulative incidence approach. Both approaches account for baseline potential confounding variables to address the non-randomized nature of the assessments.

Calendar-time-scale Cox regression approach

In the first approach, we conducted a calendar-time-scale Cox proportional-hazards regression analysis and quantified the association via the hazard ratio. The Cox regression analysis allows flexible nonparametric modeling of the baseline hazard as a function of the calendar time, which helps acknowledge that the Covid-19 epidemic could have undergone unpredictable changes over the study period. Another advantage of the Cox regression approach is that it naturally takes into account all follow-up of study participants. For each comparison between the hybrid immunity and the vaccine immunity, we considered two time-origins, corresponding to the Full Analysis Set (FAS) analysis and the Per-Protocol (PP) analysis, respectively.

In the primary comparison of Analysis Group 2-I and Analysis Group 1, we considered the following time origins:

i. Time origin of each participant equals the number of days between the participant's time of their first dose (i.e., enrolment date) and the time of the first dose of the first enrolled participant in FAS;
ii. Time origin of each participant equals the number of days between 13 days post the last dose in the series (i.e., the first dose for participants in the Analysis Group 2-1 and the second dose for participants in the Analysis Group 1) and the earlier of the following two dates: the earliest date of completion of the second dose in Analysis Group 2-1 and the earliest date of completion of the first dose in Analysis Group 1 in PP.

Time origin 1 was conducted on the FAS cohort and time origin 2 was restricted to the PP cohort. Under time origin 2 restricted to the PP cohort, the risk set at any calendar time consisted of Analysis Group 1 participants who were at least 14 days post their second pre-month-6 vaccination and Analysis Group 2-I participants who were at least 14 days post their first (and only) pre-month-6 vaccination. Time origins of other calendar-time-scale Cox regression analyses were similarly defined and conducted, and details can be found in Section 7.4 of the Statistical Analysis Plan.

Counterfactual cumulative incidence approach

In the second approach, we produced estimates of the counterfactual cumulative incidence over time, and we quantified the association via both the cumulative risk difference and the cumulative risk ratio. Under standard causal assumptions, the counterfactual probability of a Covid-19 event occurring before a given time point was identified from the observed data using a G-computation result. One obtains unbiased estimates of the counterfactual cumulative incidence when estimates of the following nuisance parameters were obtained: (i) the conditional probability of Covid-19-free survival, given prior infection status and covariates (ii) the conditional probability of censoring, given prior infection status and covariates, and (iii) the conditional probability of prior SARS-CoV-2 infection, given the covariates. Two strategies for nuisance estimation were used. Either approach yields unbiased and approximately normal estimates when the models used to estimate the nuisance parameters are specified correctly. First, the conditional probabilities of survival and censoring were estimated using Cox regression, and the conditional probability of prior SARS-CoV-2 infection was estimated using logistic regression. Second, as sensitivity analysis, nuisance parameters were estimated using the SuperLearner, an ensemble machine learning

algorithm. The conditional probability of survival and the conditional probability of censoring were estimated using SuperLearner libraries that included the following estimators: (i) the Kaplan-Meier estimator; (ii) the Cox proportional hazard estimator; (iii) generalized additive models; (iv) survival random forests. The conditional probability of prior SARS-CoV-2 infection was estimated using a SuperLearner library that included the following estimators: (i) the sample average; (ii) a logistic regression model; (iii) generalized additive models; (iv) random forests.

As in the Cox regression analysis, different time origins were defined for the FAS cohort and the PP cohort. The definition of the time origins was the same in both the cumulative incidence and Cox analyses. Estimates of the cumulative incidence curves were produced for all time points between the time origin and a final stopping time. In the FAS cohort, the final stopping time was selected as the minimum of (i) 180 days after time origin 1 and (ii) the latest time point at which the standard error of the cumulative risk ratio estimate fell below a specified threshold. The threshold for the standard error was chosen as the largest value for which an estimated cumulative risk ratio exceeding 3.0 would be deemed statistically significant at the .05 level, using the rationale that an estimate could only be considered valuable if there was enough precision to conclude that a large cumulative risk ratio was greater than one. Similarly, in the PP cohort, the final stopping time was selected as the minimum of (i) 165 days after time origin 2 and (ii) the latest time point at which the standard error of the cumulative risk ratio estimate fell below the same pre-specified threshold. Study participants with an endpoint observed after the stopping time were considered to have censored outcomes.

Analysis of NAAT-persistence

Determining the duration of NAAT positivity was difficult in this study due to the coarse sampling schedule, especially for those identified as NAAT positive at a vaccination visit. To assess duration in this context, NAAT positive swabs were assumed to be from a single infection unless re-infection was implied by the qualitative NAAT trajectory. In particular, re-infection (or a new infection) was assumed if either: 1) a positive NAAT was proceeded by two consecutive NAAT negative swabs, or 2) a single NAAT negative test was observed more than 90 days before the NAAT positive result. Ongoing sequencing analyses may be used in future analyses to distinguish ongoing infection from re-infection.

The duration of NAAT positivity pertaining to the first occurrence of NAAT-confirmed SARS-CoV-2 infection (regardless of symptomology), including those diagnosed at baseline. An individual was considered to have persistent NAAT positivity if they had NAAT positive swabs lasting at least 50 days during a single infection. The number and percentage of persistent infections of FAS infections were summarized by HIV status, and by other baseline characteristics of interest amongst PLWH, including baseline TB status, HIV-1 RNA VL ≥50, and baseline CD4 count <200. The number and percentage of persistent NAAT positive infections was also calculated among those participants with at least 2 NAAT results associated with the infection as the denominator.

Supplemental Tables

Table S1. Full eligibility criteria.

1.	Age ≥ 18 years if participant self-reports living with HIV or another comorbidity known to be associated with severe Covid-19, for example (CDC.gov for exhaustive list):<br>• Hypertension<br>• Type 2 diabetes mellitus<br>• Overweight, obese, or severely obese (i.e., body mass index [BMI] ≥ 25 kg/m2)<br>• Heart conditions, such as heart failure, coronary artery disease, or cardiomyopathies<br>• Chronic kidney disease<br>• COPD (chronic obstructive pulmonary disease)<br>• Cancer<br>• Non-HIV immunocompromised state (weakened immune system) or solid organ transplant<br>• Pregnancy<br>• Sickle cell disease<br>• Smoking
2.	Willingness to be followed and remain in the catchment area for the planned duration of the study.
3.	Ability and willingness to provide informed consent.
4.	Willingness to discuss HIV infection status, undergo related testing/monitoring labs, and receive counseling and referrals to minimize HIV acquisition/improve HIV care as appropriate based on their infection status.
5.	Assessment of Understanding (AoU): Participant demonstrates understanding of this study; completes a questionnaire prior to first vaccination with demonstration of understanding of all questionnaire items answered incorrectly.
6.	Agrees not to enroll in another interventional study of an investigational research agent until after the study is completed and all the data has been obtained. Enrolment in studies of investigational research agents for the treatment of Covid-19 is allowed for participants who develop Covid-19 disease.

Table S2. Covid-19 and severe Covid-19 definitions.

	Covid-19 (CDC)	Covid-19 (COVE)	Severe Covid-19
**Post-baseline NAAT**	Positive	Positive	Positive
**Symptom onset**	Within 14 days before or after +NAAT	Within 14 days before +NAAT	Within 14 days before +NAAT
**Eligible systemic symptoms**	At least ONE of the following systemic symptoms:<br>• fever (≥ 38ºC)<br>• chills<br>• myalgia<br>• headache<br>• sore throat<br>• new loss of taste or smell	At least TWO of the following systemic symptoms:<br>• fever (≥ 38ºC)<br>• chills<br>• myalgia<br>• headache<br>• sore throat<br>• new loss of taste or smell	At least TWO of the following systemic symptoms:<br>• fever (≥ 38ºC)<br>• chills<br>• myalgia<br>• headache<br>• sore throat<br>• new loss of taste or smell
**OR**			
**Eligible respiratory symptom(s)**	At least ONE of the following respiratory signs/symptoms:<br>• cough<br>• shortness of breath or difficulty breathing<br>• clinical or radiographical evidence of pneumonia	At least ONE of the following respiratory signs/symptoms:<br>• cough<br>• shortness of breath or difficulty breathing<br>• clinical or radiographical evidence of pneumonia	At least ONE of the following respiratory signs/symptoms:<br>• cough<br>• shortness of breath or difficulty breathing<br>• clinical or radiographical evidence of pneumonia
**Eligible severe conditions**	n/a	n/a	ANY of the following:<br>• clinical signs indicative of severe systemic illness<br>• respiratory rate ≥ 30 breaths per minute<br>• heart rate ≥ 125 beats per minute<br>• $SpO_2 \leq 93\%$ on room air at sea level or $PaO_2/FIO_2 <$ 300 mm Hg<br>• respiratory failure or ARDS*<br>• evidence of shock**<br>• significant acute renal, hepatic, or neurologic dysfunction<br>• admission to an intensive care unit or death.
**Severe condition timing**	n/a	n/a	Within 14 days before / 28 days after +NAAT
**Date of diagnosis**	Earlier date of earliest eligible symptom and NAAT	Later date of earliest eligible symptom and NAAT	Later date of documented Covid-19 and earliest eligible severe symptom
**Independent adjudication**	Not required	Required	Required

*ARDS (Acute Respiratory Distress Syndrome) is defined as needing high-flow oxygen, noninvasive or mechanical ventilation, or ECMO (extracorporeal membrane oxygenation).

**Evidence of shock includes systolic blood pressure < 90 mmHg, diastolic blood pressue < 60 mmHg, or requiring vasopressors.

Table S3. Enrolment by country and HIV status, Full Analysis Set

Country	People living with HIV N = 11681	People living without HIV N = 2321	Total N = 14002
Botswana	72 (0.6%)	47 (2.0%)	119 (0.8%)
Eswatini	202 (1.7%)	7 (0.3%)	209 (1.5%)
Kenya	891 (7.6%)	53 (2.3%)	944 (6.7%)
Malawi	640 (5.5%)	2 (0.1%)	642 (4.6%)
South Africa	7776 (66.6%)	2013 (86.7%)	9789 (69.9%)
Uganda	1080 (9.2%)	104 (4.5%)	1184 (8.5%)
Zambia	1020 (8.7%)	95 (4.1%)	1115 (8.0%)

Table S4. Baseline demographic and clinical characteristics of the Safety Subset by the six analysis groups.

Characteristics	Study Group 1		Study Group 2	Study Group 3		Study Group 4	Total N = 1491
	AG1 (N = 327)	AG2-2 (N = 231)	AG2-1 (N = 692)	AG3 (N = 44)	AG4-2 (N = 55)	AG4-1 (N = 142)	
Point-of-care anti-spike test result	Neg	Neg	Pos	Neg	Neg	Pos	
Number of primary series vaccine doses assigned	2	2	1	2	2	1	
Received vaccine doses as assigned	318 (97.2%)	225 (97.4%)	692 (100%)	44 (100%)	54 (98.2%)	142 (100%)	1475 (98.9%)
SARS-CoV-2 NAAT result - N (%)							
Negative	326 (99.7%)	163 (70.6%)	644 (93.1%)	43 (97.7%)	47 (85.5%)	133 (93.7%)	1356 (90.9%)
Positive	0 (0.0%)	68 (29.4%)	47 (6.8%)	0 (0.0%)	8 (14.5%)	8 (5.6%)	131 (8.8%)
Missing	1 (0.3%)	0 (0.0%)	1 (0.1%)	1 (2.3%)	0 (0.0%)	1 (0.7%)	4 (0.3%)
Central lab anti-NP test result - N (%)							
Negative	327 (100.0%)	33 (14.3%)	270 (39.0%)	43 (97.7%)	0 (0.0%)	53 (37.3%)	726 (48.7%)
Positive	0 (0.0%)	198 (85.7%)	420 (60.7%)	0 (0.0%)	55 (100.0%)	88 (62.0%)	761 (51.0%)
Missing	0 (0.0%)	0 (0.0%)	2 (0.3%)	1 (2.3%)	0 (0.0%)	1 (0.7%)	4 (0.3%)
Overall SARS-CoV-2 status*	Neg	Pos	Pos	Neg	Pos	Pos	
Sex assigned at birth - N (%)							

Male	115 (35.2%)	72 (31.2%)	145 (21.0%)	26 (59.1%)	31 (56.4%)	50 (35.2%)	439 (29.4%)
Female	212 (64.8%)	159 (68.8%)	547 (79.0%)	18 (40.9%)	24 (43.6%)	92 (64.8%)	1052 (70.6%)
Age (years) – median (range)	39.0 (18.0, 70.0)	39.0 (18.0, 71.0)	39.0 (19.0, 65.0)	34.0 (19.0, 74.0)	44.0 (19.0, 82.0)	36.0 (18.0, 67.0)	39.0 (18.0, 82.0)
Age category - N (%)							
≤40 years	179 (54.7%)	135 (58.4%)	399 (57.7%)	26 (59.1%)	21 (38.2%)	82 (57.7%)	842 (56.5%)
>40 years	148 (45.3%)	96 (41.6%)	293 (42.3%)	18 (40.9%)	34 (61.8%)	60 (42.3%)	649 (43.5%)
Active tuberculosis							
Yes	4 (1.2%)	1 (0.4%)	3 (0.4%)	0 (0.0%)	0 (0.0%)	2 (1.4%)	10 (0.7%)
No	308 (94.2%)	225 (97.4%)	657 (94.9%)	42 (95.5%)	53 (96.4%)	133 (93.7%)	1418 (95.1%)
Missing	15 (4.6%)	5 (2.2%)	32 (4.6%)	2 (4.5%)	2 (3.6%)	7 (4.9%)	63 (4.2%)
History of tuberculosis							
Yes	46 (14.1%)	44 (19.0%)	99 (14.3%)	1 (2.3%)	3 (5.5%)	5 (3.5%)	198 (13.3%)
No	266 (81.3%)	182 (78.8%)	561 (81.1%)	41 (93.2%)	50 (90.9%)	130 (91.5%)	1230 (82.5%)
Missing	15 (4.6%)	5 (2.2%)	32 (4.6%)	2 (4.5%)	2 (3.6%)	7 (4.9%)	63 (4.2%)
CD4 count** (cells/µ) - N (%)							
<200	35 (10.7%)	16 (6.9%)	40 (5.8%)	-	-	-	91 (7.3%)
200 - <350	48 (14.7%)	27 (11.7%)	50 (7.2%)	-	-	-	125 (10.0%)
350 - <500	46 (14.1%)	38 (16.5%)	105 (15.2%)	-	-	-	189 (15.1%)
≥500	167 (51.1%)	137 (59.3%)	439 (63.4%)	-	-	-	743 (59.4%)
Missing	31 (9.5%)	13 (5.6%)	58 (8.4%)	-	-	-	102 (8.2%)

CD4 count** (cells/µ) – median (IQR)	547.5 (328.8, 799.3)	612.0 (396.5, 853.3)	655.5 (457.0, 898.5)	-	-	-	614.5 (412.8, 860.3)
HIV viral load** (copies/mL) - N (%)							
<50	213 (65.1%)	170 (73.6%)	519 (75.0%)	-	-	-	902 (72.2%)
≥50	80 (24.5%)	44 (19.0%)	109 (15.8%)	-	-	-	233 (18.6%)
Missing	34 (10.4%)	17 (7.4%)	64 (9.2%)	-	-	-	115 (9.2%)
HIV-1 viral load** (copies/mL) – median (IQR)	312 (40, 20074)	60 (40, 3623)	124.0 (40, 2514)	-	-	-	145 (40, 4354)
ART status**							
On ART	275 (84.1%)	189 (81.8%)	581 (84.0%)	-	-	-	1045 (83.6%)
Not on ART	52 (15.9%)	42 (18.2%)	111 (16.0%)	-	-	-	205 (16.4%)
Missing	0 (0.0%)	0 (0.0%)	0 (0.0%)	-	-	-	0 (0.0%)

The overall SARS-CoV-2 status was defined as positive unless baseline nasal swab SARS-CoV-2 NAAT, point-of-care anti-spike serology (POC anti-S), and central lab anti-nucleoprotein serology (anti-NP) were all negative. Prior to this assignment, missing data were imputed based on the empirical conditional probabilities observed in the study population (Supplementary Materials). Analysis groups (AG) were based on baseline HIV status, overall SARS-CoV-2 status and the number of vaccinations assigned. Specifically, AG1 represents people living with HIV (PLWH), overall SARS-CoV-2 status negative, and assigned 2 doses. AG2-1 represents PLWH, overall SARS-CoV-2 status positive (POC anti-S positive), and assigned 1 dose. AG2-2 represents PLWH, overall SARS-CoV-2 status positive (POC anti-S negative but anti-NP or NAAT positive), and assigned 2 doses. AG3 represents people living without HIV (PLWOH), SARS-CoV-2 status negative, and assigned 2 doses. AG4-1 represents PLWoH, overall SARS-CoV-2 status positive (POC anti-S positive), and assigned 1 dose. AG4-2 represents PLWoH, overall SARS-CoV-2 status positive (POC anti-S negative but anti-NP or NAAT positive), and assigned 2 doses.
NAAT, nucleic acid amplification test. ART, antiretroviral therapy.

Table S5. Summary of participant comorbidities, other than HIV, associated with increased risk of severe Covid-19, as reported at screening by ≥ 1% of participants, Full Analysis Set

Comorbidity	People living without HIV<br>N = 2321<br>n (%)	People living with HIV<br>N = 11681<br>n (%)	Total<br>N = 14002<br>n (%)
Overweight ($25 \leq$ BMI < 30)			
Yes	352 (15.2)	2727 (23.3)	3079 (22.0)
No	1969 (84.8)	8953 (76.6)	10922 (78.0)
Unknown	0 (0.0)	1 (<0.1)	1 (<0.1)
Obesity (BMI $\geq$ 30)			
Yes	641 (27.6)	2997 (25.7)	3638 (26.0)
No	1680 (72.4)	8683 (74.3)	10363 (74.0)
Unknown	0 (0.0)	1 (<0.1)	1 (<0.1)
Smoking			
Yes	1519 (65.4)	2732 (23.4)	4251 (30.4)
No	802 (34.6)	8946 (76.6)	9748 (69.6)
Unknown	0 (0.0)	3 (<0.1)	3 (<0.1)
Tuberculosis			
Yes	123 (5.3)	1762 (15.1)	1885 (13.5)
No	2150 (92.6)	9629 (82.4)	11779 (84.1)
Unknown	48 (2.1)	290 (2.5)	338 (2.4)
High Blood Pressure			
Yes	537 (23.1)	1199 (10.3)	1736 (12.4)
No	1782 (76.8)	10432 (89.3)	12214 (87.2)
Unknown	2 (0.1)	50 (0.4)	52 (0.4)
Diabetes Mellitus			
Yes	164 (7.1)	151 (1.3)	315 (2.2)
No	2156 (92.9)	11489 (98.4)	13645 (97.5)
Unknown	1 (<0.1)	41 (0.4)	42 (0.3)
Asthma			
Yes	79 (3.4)	210 (1.8)	289 (2.1)
No	2242 (96.6)	11463 (98.1)	13705 (97.9)
Unknown	0 (0.0)	8 (0.1)	8 (0.1)

Comorbidities reported at screening for <1% of participants included autoimmune disease, blood disorder, cancer, chronic bronchitis, chronic kidney disease, chronic liver disease, chronic obstructive pulmonary disease, current pregnancy, cystic fibrosis, emphysema, heart condition, immunodeficiency, neurologic condition, non-HIV immunocompromised state, solid organ transplant, pulmonary fibrosis.
Among 1117, 8994, and 10111 female-sex-assigned-at-birth participants living without HIV, living with HIV, and overall, 1.3%, 1.1%, and 1.1% were pregnant,

respectively.

Table S6. Solicited adverse events within 7 days after month 0 vaccination by grade in the Safety Subset.

Solicited Adverse Event After Month 0 Vaccination n (%)	People living with HIV			People living without HIV			Total
	Study Group 1 (HIV+, Anti-S SARS2-)	Study Group 2 (HIV+, Anti-S SARS2+)	Total HIV+	Study Group 3 (HIV-, Anti-S SARS2-)	Study Group 4 (HIV-, Anti-S SARS2+)	Total HIV-	
	(N=558)	(N=692)	(N=1250)	(N=99)	(N=142)	(N=241)	(N=1491)
Any Reactogenicity	238 (42.7)	263 (38.0)	501 (40.1)		31 (31.3)	60 (42.3)	91 (37.8)
Grade 1	158 (28.3)	167 (24.1)	325 (26.0)	19 (19.2)	37 (26.1)	56 (23.2)	381 (25.6)
Grade 2	79 (14.2)	87 (12.6)	166 (13.3)	11 (11.1)	22 (15.5)	33 (13.7)	199 (13.3)
Grade 3	1 (0.2)	9 (1.3)	10 (0.8)	1 (1.0)	1 (0.7)	2 (0.8)	12 (0.8)
Grade 4	0	0	0	0	0	0	0
Any Local Reactogenicity	183 (32.8)	187 (27.0)	370 (29.6)		23 (23.2)	49 (34.5)	72 (29.9)
Grade 1	135 (24.2)	133 (19.2)	268 (21.4)	17 (17.2)	29 (20.4)	46 (19.1)	314 (21.1)
Grade 2	47 (8.4)	52 (7.5)	99 (7.9)	5 (5.1)	20 (14.1)	25 (10.4)	124 (8.3)
Grade 3	1 (0.2)	2 (0.3)	3 (0.2)	1 (1.0)	0	1 (0.4)	4 (0.3)
Grade 4	0	0	0	0	0	0	0
Local Reactogenicity							
Erythema/Redness	25 (4.5)	20 (2.9)	45 (3.6)	4 (4.0)	12 (8.5)	16 (6.6)	61 (4.1)
Grade 1	18 (3.2)	14 (2.0)	32 (2.6)	3 (3.0)	9 (6.3)	12 (5.0)	44 (3.0)
Grade 2	7 (1.3)	6 (0.9)	13 (1.0)	1 (1.0)	3 (2.1)	4 (1.7)	17 (1.1)
Grade 3	0	0	0	0	0	0	0
Grade 4	0	0	0	0	0	0	0
Induration/Swelling	50 (9.0)	55 (7.9)	105 (8.4)	3 (3.0)	17 (12.0)	20 (8.3)	125 (8.4)
Grade 1	27 (4.8)	37 (5.3)	64 (5.1)	1 (1.0)	9 (6.3)	10 (4.1)	74 (5.0)
Grade 2	23 (4.1)	18 (2.6)	41 (3.3)	2 (2.0)	8 (5.6)	10 (4.1)	51 (3.4)
Grade 3	0	0	0	0	0	0	0
Grade 4	0	0	0	0	0	0	0
Lymphadenopathy	41 (7.3)	34 (4.9)	75 (6.0)	6 (6.1)	10 (7.0)	16 (6.6)	91 (6.1)
Grade 1	33 (5.9)	23 (3.3)	56 (4.5)	4 (4.0)	6 (4.2)	10 (4.1)	66 (4.4)
Grade 2	7 (1.3)	11 (1.6)	18 (1.4)	1 (1.0)	4 (2.8)	5 (2.1)	23 (1.5)
Grade 3	1 (0.2)	0	1 (0.1)	1 (1.0)	0	1 (0.4)	2 (0.1)
Grade 4	0	0	0	0	0	0	0
Pain/Tenderness	169 (30.3)	173 (25.0)	342 (27.4)	20 (20.2)	46 (32.4)	66 (27.4)	408 (27.4)
Grade 1	144 (25.8)	135 (19.5)	279 (22.3)	15 (15.2)	32 (22.5)	47 (19.5)	326 (21.9)
Grade 2	24 (4.3)	36 (5.2)	60 (4.8)	4 (4.0)	14 (9.9)	18 (7.5)	78 (5.2)
Grade 3	1 (0.2)	2 (0.3)	3 (0.2)	1 (1.0)	0	1 (0.4)	4 (0.3)
Grade 4	0	0	0	0	0	0	0

Any Systemic Reactogenicity	183 (32.8)	212 (30.6)	395 (31.6)	24 (24.2)	51 (35.9)	75 (31.1)	470 (31.5)
Grade 1	126 (22.6)	132 (19.1)	258 (20.6)	13 (13.1)	38 (26.8)	51 (21.2)	309 (20.7)
Grade 2	56 (10.0)	72 (10.4)	128 (10.2)	10 (10.1)	12 (8.5)	22 (9.1)	150 (10.1)
Grade 3	1 (0.2)	8 (1.2)	9 (0.7)	1 (1.0)	1 (0.7)	2 (0.8)	11 (0.7)
Grade 4	0	0	0	0	0	0	0
Systemic Reactogenicity							
Arthralgia	70 (12.5)	70 (10.1)	140 (11.2)	11 (11.1)	15 (10.6)	26 (10.8)	166 (11.1)
Grade 1	48 (8.6)	45 (6.5)	93 (7.4)	7 (7.1)	8 (5.6)	15 (6.2)	108 (7.2)
Grade 2	22 (3.9)	23 (3.3)	45 (3.6)	3 (3.0)	7 (4.9)	10 (4.1)	55 (3.7)
Grade 3	0	2 (0.3)	2 (0.2)	1 (1.0)	0	1 (0.4)	3 (0.2)
Grade 4	0	0	0	0	0	0	0
Chills	47 (8.4)	52 (7.5)	99 (7.9)	9 (9.1)	8 (5.6)	17 (7.1)	116 (7.8)
Grade 1	37 (6.6)	36 (5.2)	73 (5.8)	5 (5.1)	7 (4.9)	12 (5.0)	85 (5.7)
Grade 2	10 (1.8)	15 (2.2)	25 (2.0)	4 (4.0)	1 (0.7)	5 (2.1)	30 (2.0)
Grade 3	0	1 (0.1)	1 (0.1)	0	0	0	1 (0.1)
Fever	18 (3.2)	31 (4.5)	49 (3.9)	2 (2.0)	2 (1.4)	4 (1.7)	53 (3.6)
Grade 1	11 (2.0)	19 (2.7)	30 (2.4)	1 (1.0)	2 (1.4)	3 (1.2)	33 (2.2)
Grade 2	7 (1.3)	10 (1.4)	17 (1.4)	1 (1.0)	0	1 (0.4)	18 (1.2)
Grade 3	0	2 (0.3)	2 (0.2)	0	0	0	2 (0.1)
Grade 4	0	0	0	0	0	0	0
Headache	123 (22.0)	134 (19.4)	257 (20.6)	14 (14.1)	36 (25.4)	50 (20.7)	307 (20.6)
Grade 1	96 (17.2)	104 (15.0)	200 (16.0)	11 (11.1)	29 (20.4)	40 (16.6)	240 (16.1)
Grade 2	26 (4.7)	28 (4.0)	54 (4.3)	3 (3.0)	7 (4.9)	10 (4.1)	64 (4.3)
Grade 3	1 (0.2)	2 (0.3)	3 (0.2)	0	0	0	3 (0.2)
Grade 4	0	0	0	0	0	0	0
Malaise/Fatigue	115 (20.6)	133 (19.2)	248 (19.8)	15 (15.2)	33 (23.2)	48 (19.9)	296 (19.9)
Grade 1	86 (15.4)	91 (13.2)	177 (14.2)	9 (9.1)	27 (19.0)	36 (14.9)	213 (14.3)
Grade 2	29 (5.2)	39 (5.6)	68 (5.4)	5 (5.1)	6 (4.2)	11 (4.6)	79 (5.3)
Grade 3	0	3 (0.4)	3 (0.2)	1 (1.0)	0	1 (0.4)	4 (0.3)
Grade 4	0	0	0	0	0	0	0
Myalgia	89 (15.9)	85 (12.3)	174 (13.9)	13 (13.1)	24 (16.9)	37 (15.4)	211 (14.2)
Grade 1	64 (11.5)	60 (8.7)	124 (9.9)	7 (7.1)	18 (12.7)	25 (10.4)	149 (10.0)
Grade 2	25 (4.5)	22 (3.2)	47 (3.8)	5 (5.1)	5 (3.5)	10 (4.1)	57 (3.8)
Grade 3	0	3 (0.4)	3 (0.2)	1 (1.0)	1 (0.7)	2 (0.8)	5 (0.3)
Grade 4	0	0	0	0	0	0	0
Nausea	51 (9.1)	58 (8.4)	109 (8.7)	7 (7.1)	11 (7.7)	18 (7.5)	127 (8.5)
Grade 1	36 (6.5)	38 (5.5)	74 (5.9)	6 (6.1)	9 (6.3)	15 (6.2)	89 (6.0)
Grade 2	15 (2.7)	19 (2.7)	34 (2.7)	1 (1.0)	2 (1.4)	3 (1.2)	37 (2.5)

Grade 3	0	1 (0.1)	1 (0.1)	0	0	0	1 (0.1)
Grade 4	0	0	0	0	0	0	0

Denominators for percentages are based on number of participants who received month 0 vaccination.
SARS2, SARS-CoV-2.

Table S7. Solicited adverse events within 7 days after month 1 vaccination by grade in the Safety Subset.

Solicited Adverse Events After Month 1 Vaccination n (%)	People living with HIV Study Group 1 (HIV+, Anti-S SARS2-)	People living without HIV Study Group 3 (HIV-, Anti-S SARS2-)	Total
	(N=543)	(N=98)	(N=641)
Any Reactogenicity	190 (35.0)	32 (32.7)	222 (34.6)
Grade 1	126 (23.2)	21 (21.4)	147 (22.9)
Grade 2	59 (10.9)	9 (9.2)	68 (10.6)
Grade 3	5 (0.9)	2 (2.0)	7 (1.1)
Grade 4	0	0	0
Any Local Reactogenicity	146 (26.9)	25 (25.5)	171 (26.7)
Grade 1	114 (21.0)	18 (18.4)	132 (20.6)
Grade 2	32 (5.9)	7 (7.1)	39 (6.1)
Grade 3	0	0	0
Grade 4	0	0	0
Local Reactogenicity			
Erythema/Redness	10 (1.8)	1 (1.0)	11 (1.7)
Grade 1	3 (0.6)	1 (1.0)	4 (0.6)
Grade 2	7 (1.3)	0	7 (1.1)
Grade 3	0	0	0
Grade 4	0	0	0
Induration/Swelling	33 (6.1)	3 (3.1)	36 (5.6)
Grade 1	22 (4.1)	1 (1.0)	23 (3.6)
Grade 2	11 (2.0)	2 (2.0)	13 (2.0)
Grade 3	0	0	0
Grade 4	0	0	0
Lymphadenopathy	29 (5.3)	7 (7.1)	36 (5.6)
Grade 1	24 (4.4)	5 (5.1)	29 (4.5)
Grade 2	5 (0.9)	2 (2.0)	7 (1.1)
Grade 3	0	0	0
Grade 4	0	0	0
Pain/Tenderness	139 (25.6)	23 (23.5)	162 (25.3)
Grade 1	115 (21.2)	19 (19.4)	134 (20.9)
Grade 2	24 (4.4)	4 (4.1)	28 (4.4)
Grade 3	0	0	0
Grade 4	0	0	0
Any Systemic Reactogenicity	149 (27.4)	26 (26.5)	175 (27.3)
Grade 1	102 (18.8)	16 (16.3)	118 (18.4)
Grade 2	42 (7.7)	8 (8.2)	50 (7.8)
Grade 3	5 (0.9)	2 (2.0)	7 (1.1)
Grade 4	0	0	0
Systemic Reactogenicity			
Arthralgia	60 (11.0)	12 (12.2)	72 (11.2)
Grade 1	42 (7.7)	11 (11.2)	53 (8.3)
Grade 2	17 (3.1)	1 (1.0)	18 (2.8)
Grade 3	1 (0.2)	0	1 (0.2)
Grade 4	0	0	0
Chills	48 (8.8)	8 (8.2)	56 (8.7)
Grade 1	35 (6.4)	6 (6.1)	41 (6.4)
Grade 2	11 (2.0)	2 (2.0)	13 (2.0)
Grade 3	2 (0.4)	0	2 (0.3)
Fever	15 (2.8)	4 (4.1)	19 (3.0)

Grade 1	10 (1.8)	2 (2.0)	12 (1.9)
Grade 2	4 (0.7)	2 (2.0)	6 (0.9)
Grade 3	1 (0.2)	0	1 (0.2)
Grade 4	0	0	0
Headache	109 (20.1)	18 (18.4)	127 (19.8)
Grade 1	85 (15.7)	15 (15.3)	100 (15.6)
Grade 2	21 (3.9)	2 (2.0)	23 (3.6)
Grade 3	3 (0.6)	1 (1.0)	4 (0.6)
Grade 4	0	0	0
Malaise/Fatigue	114 (21.0)	22 (22.4)	136 (21.2)
Grade 1	80 (14.7)	15 (15.3)	95 (14.8)
Grade 2	30 (5.5)	5 (5.1)	35 (5.5)
Grade 3	4 (0.7)	2 (2.0)	6 (0.9)
Grade 4	0	0	0
Myalgia	78 (14.4)	13 (13.3)	91 (14.2)
Grade 1	62 (11.4)	11 (11.2)	73 (11.4)
Grade 2	14 (2.6)	1 (1.0)	15 (2.3)
Grade 3	2 (0.4)	1 (1.0)	3 (0.5)
Grade 4	0	0	0
Nausea	41 (7.6)	10 (10.2)	51 (8.0)
Grade 1	33 (6.1)	9 (9.2)	42 (6.6)
Grade 2	7 (1.3)	1 (1.0)	8 (1.2)
Grade 3	1 (0.2)	0	1 (0.2)
Grade 4	0	0	0

Denominators for percentages are based on number of participants who received month 1 vaccination.
SARS2, SARS-CoV-2.

Table S8. Unsolicited adverse events within 28 days after month 0 or month 1 vaccination in the Safety Subset.

Participants Reporting Adverse Event n (%)	People living with HIV			People living without HIV			Total
	Study Group 1 (HIV+, Anti-S SARS2-)	Study Group 2 (HIV+, Anti-S SARS2+)	Total HIV+	Study Group 3 (HIV-, Anti-S SARS2-)	Study Group 4 (HIV-, Anti-S SARS2+)	Total HIV-	
	(N=558)	(N=692)	(N=1250)	(N=99)	(N=142)	(N=241)	(N=1491)
Regardless of relationship to study vaccination							
All	51 (9.1)	24 (3.5)	75 (6.0)	8 (8.1)	8 (5.6)	16 (6.6)	91 (6.1)
Mild	16 (2.9)	7 (1.0)	23 (1.8)	1 (1.0)	2 (1.4)	3 (1.2)	26 (1.7)
Moderate	29 (5.2)	17 (2.5)	46 (3.7)	7 (7.1)	5 (3.5)	12 (5.0)	58 (3.9)
Severe	6 (1.1)	0	6 (0.5)	0	1 (0.7)	1 (0.4)	7 (0.5)
Potentially life-threatening	0	0	0	0	0	0	0
Fatal	0	0	0	0	0	0	0
Related to study vaccination							
All	4 (0.7)	0	4 (0.3)	1 (1.0)	0	1 (0.4)	5 (0.3)
Mild	1 (0.2)	0	1 (0.1)	0	0	0	1 (0.1)
Moderate	3 (0.5)	0	3 (0.2)	1 (1.0)	0	1 (0.4)	4 (0.3)
Severe	0	0	0	0	0	0	0
Potentially life-threatening	0	0	0	0	0	0	0
Fatal	0	0	0	0	0	0	0

All AEs were graded according to the Division of AIDS Table for Grading the Severity of Adult and Pediatric Adverse Events (https://rsc.niaid.nih.gov/clinical-research-sites/daids-adverse-event-grading-tables).
SARS2, SARS-CoV-2.

Table S9. Summary of unsolicited adverse events reported by ≥1% of participants within 28 days after month 0 or month 1 vaccination in the Safety Subset.

n (%)	People living with HIV			People living without HIV			Total
	Study Group 1 (HIV+, Anti-S SARS2-)	Study Group 2 (HIV+, Anti-S SARS2+)	Total HIV+	Study Group 3 (HIV-, Anti-S SARS2-)	Study Group 4 (HIV-, Anti-S SARS2+)	Total HIV-	
	(N=558)	(N=692)	(N=1250)	(N=99)	(N=142)	(N=241)	(N=1491)
Totals							
Number of participants reporting unsolicited AEs	50 (9.0)	24 (3.5)	74 (5.9)	8 (8.1)	8 (5.6)	16 (6.6)	90 (6.0)
Number of unsolicited AEs	54	28	82	10	10	20	102
Events by System Organ Class / Preferred Term							
Infections and infestations	19 (3.4)	13 (1.9)	32 (2.6)	3 (3.0)	2 (1.4)	5 (2.1)	37 (2.5)
Abscess limb	0	1 (0.1)	1 (0.1)	0	0	0	1 (0.1)
Dysentery	0	1 (0.1)	1 (0.1)	0	0	0	1 (0.1)
Gastroenteritis	3 (0.5)	1 (0.1)	4 (0.3)	0	0	0	4 (0.3)
Gingivitis	1 (0.2)	0	1 (0.1)	0	0	0	1 (0.1)
Lower respiratory tract infection	0	1 (0.1)	1 (0.1)	0	0	0	1 (0.1)
Malaria	1 (0.2)	0	1 (0.1)	0	0	0	1 (0.1)
Otitis media acute	0	1 (0.1)	1 (0.1)	0	0	0	1 (0.1)
Pharyngitis	1 (0.2)	1 (0.1)	2 (0.2)	1 (1.0)	0	1 (0.4)	3 (0.2)
Pneumonia	0	1 (0.1)	1 (0.1)	0	0	0	1 (0.1)
Pulmonary tuberculosis	1 (0.2)	0	1 (0.1)	0	0	0	1 (0.1)
Rash pustular	0	0	0	1 (1.0)	0	1 (0.4)	1 (0.1)
Respiratory tract infection	0	1 (0.1)	1 (0.1)	0	0	0	1 (0.1)
Sexually transmitted disease	0	1 (0.1)	1 (0.1)	0	0	0	1 (0.1)
Sinusitis	0	1 (0.1)	1 (0.1)	0	0	0	1 (0.1)
Tinea faciei	2 (0.4)	0	2 (0.2)	0	0	0	2 (0.1)
Tuberculosis gastrointestinal	1 (0.2)	0	1 (0.1)	0	0	0	1 (0.1)
Upper respiratory tract infection	8 (1.4)	3 (0.4)	11 (0.9)	1 (1.0)	2 (1.4)	3 (1.2)	14 (0.9)
Vulvovaginitis trichomonal	1 (0.2)	0	1 (0.1)	0	0	0	1 (0.1)

Adverse events are sorted by descending overall system organ class frequency. All preferred terms for system organ classes experienced by ≥1% participants are displayed.
SARS2, SARS-CoV-2.

Table S10. Serious adverse events up till month 6 visit reported by preferred term in the Full Analysis Set.

n (%)	PLWH			HIV Negative			Total
	Study Group 1 (HIV+, Anti-S SARS2-)	Study Group 2 (HIV+, Anti-S SARS2+)	Total HIV+	Study Group 3 (HIV-, Anti-S SARS2-)	Study Group 4 (HIV-, Anti-S SARS2+)	Total HIV-	
	(N=3740)	(N=7941)	(N=11681)	(N=648)	(N=1673)	(N=2321)	(N=14002)
Totals							
Number of participants reporting serious AEs	86 (2.3) (1.79, 2.74)	124 (1.6) (1.31, 1.86)	210 (1.8) (1.55, 2.03)	5 (0.8) (0.33, 1.79)	32 (1.9) (1.36, 2.69)	37 (1.6) (1.16, 2.19)	247 (1.8) (1.54, 1.97)
Number of serious AEs	98	132	230	6	36	42	272
Number of serious AEs, related to study vaccination	0	1	1	0	0	0	1
Events by Preferred Term							
Pneumonia	9 (0.2) (0.13, 0.46)	6 (0.1) (0.03, 0.16)	15 (0.1) (0.08, 0.21)	0	3 (0.2) (0.06, 0.53)	3 (0.1) (0.04, 0.38)	18 (0.1) (0.08, 0.20)
Pulmonary tuberculosis	2 (0.1) (0.01, 0.19)	12 (0.2) (0.09, 0.26)	14 (0.1) (0.07, 0.20)	0	1 (0.1) (0.00, 0.34)	1 (<0.1) (0.00, 0.24)	15 (0.1) (0.06, 0.18)
Abnormal loss of weight	0	1 (<0.1) (0.00, 0.07)	1 (<0.1) (0.00, 0.05)	0	0	0	1 (<0.1) (0.00, 0.04)
Abnormal uterine bleeding	1 (<0.1) (0.00, 0.15)	2 (<0.1) (0.01, 0.09)	3 (<0.1) (0.01, 0.08)	0	1 (0.1) (0.00, 0.34)	1 (<0.1) (0.00, 0.24)	4 (<0.1) (0.01, 0.07)
Abortion incomplete	0	2 (<0.1) (0.01, 0.09)	2 (<0.1) (0.00, 0.06)	0	0	0	2 (<0.1) (0.00, 0.05)
Abortion spontaneous	0	4 (0.1) (0.02, 0.13)	4 (<0.1) (0.01, 0.09)	0	0	0	4 (<0.1) (0.01, 0.07)
Accident	0	1 (<0.1) (0.00, 0.07)	1 (<0.1) (0.00, 0.05)	0	0	0	1 (<0.1) (0.00, 0.04)
Acute kidney injury	2 (0.1) (0.01, 0.19)	2 (<0.1) (0.01, 0.09)	4 (<0.1) (0.01, 0.09)	0	0	0	4 (<0.1) (0.01, 0.07)
Acute lymphocytic leukaemia	1 (<0.1) (0.00, 0.15)	0	1 (<0.1) (0.00, 0.05)	0	0	0	1 (<0.1) (0.00, 0.04)
Acute myocardial infarction	1 (<0.1) (0.00, 0.15)	0	1 (<0.1) (0.00, 0.05)	0	0	0	1 (<0.1) (0.00, 0.04)
Alcohol poisoning	1 (<0.1) (0.00, 0.15)	0	1 (<0.1) (0.00, 0.05)	0	0	0	1 (<0.1) (0.00, 0.04)
Alcohol withdrawal syndrome	1 (<0.1) (0.00, 0.15)	1 (<0.1)	2 (<0.1)	0	0	0	2 (<0.1)

		(0.00, 0.07)	(0.00, 0.06)				(0.00, 0.05)
Anaemia	2 (0.1) (0.01, 0.19)	2 (<0.1) (0.01, 0.09)	4 (<0.1) (0.01, 0.09)	0	0	0	4 (<0.1) (0.01, 0.07)
Anal abscess	0	1 (<0.1) (0.00, 0.07)	1 (<0.1) (0.00, 0.05)	0	0	0	1 (<0.1) (0.00, 0.04)
Animal bite	1 (<0.1) (0.00, 0.15)	0	1 (<0.1) (0.00, 0.05)	0	0	0	1 (<0.1) (0.00, 0.04)
Ankle fracture	1 (<0.1) (0.00, 0.15)	0	1 (<0.1) (0.00, 0.05)	1 (0.2) (0.01, 0.87)	0	1 (<0.1) (0.00, 0.24)	2 (<0.1) (0.00, 0.05)
Appendicitis	0	1 (<0.1) (0.00, 0.07)	1 (<0.1) (0.00, 0.05)	0	0	0	1 (<0.1) (0.00, 0.04)
Arthritis bacterial	0	1 (<0.1) (0.00, 0.07)	1 (<0.1) (0.00, 0.05)	0	0	0	1 (<0.1) (0.00, 0.04)
Asthma	0	1 (<0.1) (0.00, 0.07)	1 (<0.1) (0.00, 0.05)	0	0	0	1 (<0.1) (0.00, 0.04)
Back pain	1 (<0.1) (0.00, 0.15)	0	1 (<0.1) (0.00, 0.05)	0	0	0	1 (<0.1) (0.00, 0.04)
Bicytopenia	1 (<0.1) (0.00, 0.15)	0	1 (<0.1) (0.00, 0.05)	0	0	0	1 (<0.1) (0.00, 0.04)
Bipolar I disorder	0	0	0	0	1 (0.1) (0.00, 0.34)	1 (<0.1) (0.00, 0.24)	1 (<0.1) (0.00, 0.04)
Bladder cyst	0	1 (<0.1) (0.00, 0.07)	1 (<0.1) (0.00, 0.05)	0	0	0	1 (<0.1) (0.00, 0.04)
Bone tuberculosis	1 (<0.1) (0.00, 0.15)	0	1 (<0.1) (0.00, 0.05)	0	0	0	1 (<0.1) (0.00, 0.04)
Brain oedema	0	0	0	1 (0.2) (0.01, 0.87)	0	1 (<0.1) (0.00, 0.24)	1 (<0.1) (0.00, 0.04)
Brain stem syndrome	1 (<0.1) (0.00, 0.15)	0	1 (<0.1) (0.00, 0.05)	0	0	0	1 (<0.1) (0.00, 0.04)
Bronchiectasis	1 (<0.1) (0.00, 0.15)	1 (<0.1) (0.00, 0.07)	2 (<0.1) (0.00, 0.06)	0	0	0	2 (<0.1) (0.00, 0.05)
Cardiac failure	0	1 (<0.1) (0.00, 0.07)	1 (<0.1) (0.00, 0.05)	0	0	0	1 (<0.1) (0.00, 0.04)
Cardiac failure acute	0	0	0	0	1 (0.1) (0.00, 0.34)	1 (<0.1) (0.00, 0.24)	1 (<0.1) (0.00, 0.04)
Cardiac failure	2 (0.1)	1 (<0.1)	3 (<0.1)	0	0	0	3 (<0.1)

congestive	(0.01, 0.19)	(0.00, 0.07)	(0.01, 0.08)				(0.01, 0.06)
Cellulitis	1 (<0.1) (0.00, 0.15)	0	1 (<0.1) (0.00, 0.05)	0	0	0	1 (<0.1) (0.00, 0.04)
Cerebrovascular accident	3 (0.1) (0.03, 0.24)	2 (<0.1) (0.01, 0.09)	5 (<0.1) (0.02, 0.10)	0	2 (0.1) (0.03, 0.43)	2 (0.1) (0.02, 0.31)	7 (<0.1) (0.02, 0.10)
Cervix carcinoma	2 (0.1) (0.01, 0.19)	2 (<0.1) (0.01, 0.09)	4 (<0.1) (0.01, 0.09)	0	0	0	4 (<0.1) (0.01, 0.07)
Cholecystitis	0	0	0	0	1 (0.1) (0.00, 0.34)	1 (<0.1) (0.00, 0.24)	1 (<0.1) (0.00, 0.04)
Cholecystitis acute	0	1 (<0.1) (0.00, 0.07)	1 (<0.1) (0.00, 0.05)	0	0	0	1 (<0.1) (0.00, 0.04)
Completed suicide	0	1 (<0.1) (0.00, 0.07)	1 (<0.1) (0.00, 0.05)	0	0	0	1 (<0.1) (0.00, 0.04)
Conversion disorder	1 (<0.1) (0.00, 0.15)	0	1 (<0.1) (0.00, 0.05)	0	0	0	1 (<0.1) (0.00, 0.04)
Cor pulmonale	0	0	0	0	1 (0.1) (0.00, 0.34)	1 (<0.1) (0.00, 0.24)	1 (<0.1) (0.00, 0.04)
Death	1 (<0.1) (0.00, 0.15)	2 (<0.1) (0.01, 0.09)	3 (<0.1) (0.01, 0.08)	0	0	0	3 (<0.1) (0.01, 0.06)
Deep vein thrombosis	0	2 (<0.1) (0.01, 0.09)	2 (<0.1) (0.00, 0.06)	0	0	0	2 (<0.1) (0.00, 0.05)
Device related infection	1 (<0.1) (0.00, 0.15)	0	1 (<0.1) (0.00, 0.05)	0	0	0	1 (<0.1) (0.00, 0.04)
Diabetic foot	0	1 (<0.1) (0.00, 0.07)	1 (<0.1) (0.00, 0.05)	0	1 (0.1) (0.00, 0.34)	1 (<0.1) (0.00, 0.24)	2 (<0.1) (0.00, 0.05)
Diabetic ketoacidosis	0	2 (<0.1) (0.01, 0.09)	2 (<0.1) (0.00, 0.06)	0	3 (0.2) (0.06, 0.53)	3 (0.1) (0.04, 0.38)	5 (<0.1) (0.02, 0.08)
Diarrhoea	2 (0.1) (0.01, 0.19)	2 (<0.1) (0.01, 0.09)	4 (<0.1) (0.01, 0.09)	0	0	0	4 (<0.1) (0.01, 0.07)
Disseminated tuberculosis	1 (<0.1) (0.00, 0.15)	2 (<0.1) (0.01, 0.09)	3 (<0.1) (0.01, 0.08)	0	0	0	3 (<0.1) (0.01, 0.06)
Drug-induced liver injury	0	1 (<0.1) (0.00, 0.07)	1 (<0.1) (0.00, 0.05)	0	0	0	1 (<0.1) (0.00, 0.04)
Dysentery	0	0	0	0	1 (0.1) (0.00, 0.34)	1 (<0.1) (0.00, 0.24)	1 (<0.1) (0.00, 0.04)
Ectopic pregnancy	0	1 (<0.1)	1 (<0.1)	0	0	0	1 (<0.1)

		(0.00, 0.07)	(0.00, 0.05)				(0.00, 0.04)
End stage renal disease	0	0	0	0	1 (0.1) (0.00, 0.34)	1 (<0.1) (0.00, 0.24)	1 (<0.1) (0.00, 0.04)
Epidural haemorrhage	0	0	0	0	1 (0.1) (0.00, 0.34)	1 (<0.1) (0.00, 0.24)	1 (<0.1) (0.00, 0.04)
Extrapulmonary tuberculosis	1 (<0.1) (0.00, 0.15)	3 (<0.1) (0.01, 0.11)	4 (<0.1) (0.01, 0.09)	0	0	0	4 (<0.1) (0.01, 0.07)
Eye infection syphilitic	0	1 (<0.1) (0.00, 0.07)	1 (<0.1) (0.00, 0.05)	0	0	0	1 (<0.1) (0.00, 0.04)
Femur fracture	0	0	0	0	1 (0.1) (0.00, 0.34)	1 (<0.1) (0.00, 0.24)	1 (<0.1) (0.00, 0.04)
Gallbladder cancer	0	1 (<0.1) (0.00, 0.07)	1 (<0.1) (0.00, 0.05)	0	0	0	1 (<0.1) (0.00, 0.04)
Gastric ulcer	0	1 (<0.1) (0.00, 0.07)	1 (<0.1) (0.00, 0.05)	0	0	0	1 (<0.1) (0.00, 0.04)
Gastritis	0	1 (<0.1) (0.00, 0.07)	1 (<0.1) (0.00, 0.05)	0	0	0	1 (<0.1) (0.00, 0.04)
Gastroenteritis	2 (0.1) (0.01, 0.19)	1 (<0.1) (0.00, 0.07)	3 (<0.1) (0.01, 0.08)	0	0	0	3 (<0.1) (0.01, 0.06)
Gun shot wound	5 (0.1) (0.06, 0.31)	1 (<0.1) (0.00, 0.07)	6 (0.1) (0.02, 0.11)	0	0	0	6 (<0.1) (0.02, 0.09)
HIV peripheral neuropathy	1 (<0.1) (0.00, 0.15)	1 (<0.1) (0.00, 0.07)	2 (<0.1) (0.00, 0.06)	0	1 (0.1) (0.00, 0.34)	1 (<0.1) (0.00, 0.24)	3 (<0.1) (0.01, 0.06)
HIV wasting syndrome	0	1 (<0.1) (0.00, 0.07)	1 (<0.1) (0.00, 0.05)	0	0	0	1 (<0.1) (0.00, 0.04)
Haemorrhage in pregnancy	1 (<0.1) (0.00, 0.15)	0	1 (<0.1) (0.00, 0.05)	0	0	0	1 (<0.1) (0.00, 0.04)
Haemorrhage intracranial	0	0	0	0	1 (0.1) (0.00, 0.34)	1 (<0.1) (0.00, 0.24)	1 (<0.1) (0.00, 0.04)
Haemorrhoids	0	0	0	0	1 (0.1) (0.00, 0.34)	1 (<0.1) (0.00, 0.24)	1 (<0.1) (0.00, 0.04)
Head injury	0	1 (<0.1) (0.00, 0.07)	1 (<0.1) (0.00, 0.05)	0	0	0	1 (<0.1) (0.00, 0.04)
Herpes zoster	0	3 (<0.1)	3 (<0.1)	0	0	0	3 (<0.1)

disseminated		(0.01, 0.11)	(0.01, 0.08)				(0.01, 0.06)
Human bite	0	1 (<0.1) (0.00, 0.07)	1 (<0.1) (0.00, 0.05)	0	0	0	1 (<0.1) (0.00, 0.04)
Humerus fracture	0	0	0	0	1 (0.1) (0.00, 0.34)	1 (<0.1) (0.00, 0.24)	1 (<0.1) (0.00, 0.04)
Hydrosalpinx	1 (<0.1) (0.00, 0.15)	2 (<0.1) (0.01, 0.09)	3 (<0.1) (0.01, 0.08)	0	0	0	3 (<0.1) (0.01, 0.06)
Hypertensive emergency	1 (<0.1) (0.00, 0.15)	0	1 (<0.1) (0.00, 0.05)	0	0	0	1 (<0.1) (0.00, 0.04)
Hypertensive urgency	0	0	0	0	2 (0.1) (0.03, 0.43)	2 (0.1) (0.02, 0.31)	2 (<0.1) (0.00, 0.05)
Hypoglycaemia	0	1 (<0.1) (0.00, 0.07)	1 (<0.1) (0.00, 0.05)	0	0	0	1 (<0.1) (0.00, 0.04)
Incarcerated inguinal hernia	0	2 (<0.1) (0.01, 0.09)	2 (<0.1) (0.00, 0.06)	0	0	0	2 (<0.1) (0.00, 0.05)
Infection	0	1 (<0.1) (0.00, 0.07)	1 (<0.1) (0.00, 0.05)	0	0	0	1 (<0.1) (0.00, 0.04)
Inguinal hernia strangulated	0	1 (<0.1) (0.00, 0.07)	1 (<0.1) (0.00, 0.05)	0	0	0	1 (<0.1) (0.00, 0.04)
Injury	1 (<0.1) (0.00, 0.15)	0	1 (<0.1) (0.00, 0.05)	0	0	0	1 (<0.1) (0.00, 0.04)
Intentional self-injury	1 (<0.1) (0.00, 0.15)	0	1 (<0.1) (0.00, 0.05)	0	1 (0.1) (0.00, 0.34)	1 (<0.1) (0.00, 0.24)	2 (<0.1) (0.00, 0.05)
Intestinal obstruction	0	3 (<0.1) (0.01, 0.11)	3 (<0.1) (0.01, 0.08)	1 (0.2) (0.01, 0.87)	0	1 (<0.1) (0.00, 0.24)	4 (<0.1) (0.01, 0.07)
Iron deficiency anaemia	1 (<0.1) (0.00, 0.15)	1 (<0.1) (0.00, 0.07)	2 (<0.1) (0.00, 0.06)	0	0	0	2 (<0.1) (0.00, 0.05)
Joint injury	1 (<0.1) (0.00, 0.15)	0	1 (<0.1) (0.00, 0.05)	0	0	0	1 (<0.1) (0.00, 0.04)
Lower limb fracture	1 (<0.1) (0.00, 0.15)	0	1 (<0.1) (0.00, 0.05)	0	0	0	1 (<0.1) (0.00, 0.04)
Lower respiratory tract infection	2 (0.1) (0.01, 0.19)	0	2 (<0.1) (0.00, 0.06)	0	0	0	2 (<0.1) (0.00, 0.05)

Major depression	1 (<0.1) (0.00, 0.15)	0	1 (<0.1) (0.00, 0.05)	0	0	0	1 (<0.1) (0.00, 0.04)
Malaria	1 (<0.1) (0.00, 0.15)	2 (<0.1) (0.01, 0.09)	3 (<0.1) (0.01, 0.08)	0	0	0	3 (<0.1) (0.01, 0.06)
Male genital tract tuberculosis	0	1 (<0.1) (0.00, 0.07)	1 (<0.1) (0.00, 0.05)	0	0	0	1 (<0.1) (0.00, 0.04)
Malignant neoplasm of unknown primary site	1 (<0.1) (0.00, 0.15)	0	1 (<0.1) (0.00, 0.05)	0	0	0	1 (<0.1) (0.00, 0.04)
Meningitis bacterial	0	1 (<0.1) (0.00, 0.07)	1 (<0.1) (0.00, 0.05)	0	0	0	1 (<0.1) (0.00, 0.04)
Meningitis cryptococcal	1 (<0.1) (0.00, 0.15)	1 (<0.1) (0.00, 0.07)	2 (<0.1) (0.00, 0.06)	0	0	0	2 (<0.1) (0.00, 0.05)
Meningitis tuberculous	0	1 (<0.1) (0.00, 0.07)	1 (<0.1) (0.00, 0.05)	0	0	0	1 (<0.1) (0.00, 0.04)
Meningoencephalitis bacterial	0	1 (<0.1) (0.00, 0.07)	1 (<0.1) (0.00, 0.05)	0	0	0	1 (<0.1) (0.00, 0.04)
Mixed anxiety and depressive disorder	0	1 (<0.1) (0.00, 0.07)	1 (<0.1) (0.00, 0.05)	0	0	0	1 (<0.1) (0.00, 0.04)
Necrotising fasciitis	0	0	0	1 (0.2) (0.01, 0.87)	0	1 (<0.1) (0.00, 0.24)	1 (<0.1) (0.00, 0.04)
Neuropathy peripheral	0	1 (<0.1) (0.00, 0.07)	1 (<0.1) (0.00, 0.05)	0	0	0	1 (<0.1) (0.00, 0.04)
Oesophageal carcinoma	1 (<0.1) (0.00, 0.15)	0	1 (<0.1) (0.00, 0.05)	0	0	0	1 (<0.1) (0.00, 0.04)
Oligohydramnios	0	0	0	0	1 (0.1) (0.00, 0.34)	1 (<0.1) (0.00, 0.24)	1 (<0.1) (0.00, 0.04)
Pancreatitis acute	0	2 (<0.1) (0.01, 0.09)	2 (<0.1) (0.00, 0.06)	0	0	0	2 (<0.1) (0.00, 0.05)
Patella fracture	0	1 (<0.1) (0.00, 0.07)	1 (<0.1) (0.00, 0.05)	0	0	0	1 (<0.1) (0.00, 0.04)
Pelvic fracture	0	1 (<0.1) (0.00, 0.07)	1 (<0.1) (0.00, 0.05)	0	0	0	1 (<0.1) (0.00, 0.04)
Pelvic inflammatory disease	0	1 (<0.1) (0.00, 0.07)	1 (<0.1) (0.00, 0.05)	0	0	0	1 (<0.1) (0.00, 0.04)

Peptic ulcer	1 (<0.1) (0.00, 0.15)	0	1 (<0.1) (0.00, 0.05)	0	0	0	1 (<0.1) (0.00, 0.04)
Perineal injury	1 (<0.1) (0.00, 0.15)	0	1 (<0.1) (0.00, 0.05)	0	0	0	1 (<0.1) (0.00, 0.04)
Physical assault	0	1 (<0.1) (0.00, 0.07)	1 (<0.1) (0.00, 0.05)	0	0	0	1 (<0.1) (0.00, 0.04)
Pneumocystis jirovecii pneumonia	0	1 (<0.1) (0.00, 0.07)	1 (<0.1) (0.00, 0.05)	0	0	0	1 (<0.1) (0.00, 0.04)
Pneumonia klebsiella	1 (<0.1) (0.00, 0.15)	0	1 (<0.1) (0.00, 0.05)	0	0	0	1 (<0.1) (0.00, 0.04)
Pneumothorax spontaneous	1 (<0.1) (0.00, 0.15)	0	1 (<0.1) (0.00, 0.05)	0	0	0	1 (<0.1) (0.00, 0.04)
Post procedural sepsis	1 (<0.1) (0.00, 0.15)	0	1 (<0.1) (0.00, 0.05)	0	0	0	1 (<0.1) (0.00, 0.04)
Post-traumatic epilepsy	0	1 (<0.1) (0.00, 0.07)	1 (<0.1) (0.00, 0.05)	0	0	0	1 (<0.1) (0.00, 0.04)
Pre-eclampsia	0	1 (<0.1) (0.00, 0.07)	1 (<0.1) (0.00, 0.05)	0	0	0	1 (<0.1) (0.00, 0.04)
Preterm premature rupture of membranes	1 (<0.1) (0.00, 0.15)	0	1 (<0.1) (0.00, 0.05)	0	0	0	1 (<0.1) (0.00, 0.04)
Psychotic disorder	1 (<0.1) (0.00, 0.15)	0	1 (<0.1) (0.00, 0.05)	0	0	0	1 (<0.1) (0.00, 0.04)
Pulmonary embolism	0	2 (<0.1) (0.01, 0.09)	2 (<0.1) (0.00, 0.06)	0	0	0	2 (<0.1) (0.00, 0.05)
Pyelonephritis	0	0	0	0	1 (0.1) (0.00, 0.34)	1 (<0.1) (0.00, 0.24)	1 (<0.1) (0.00, 0.04)
Rectal adenocarcinoma	0	0	0	0	1 (0.1) (0.00, 0.34)	1 (<0.1) (0.00, 0.24)	1 (<0.1) (0.00, 0.04)
Rectal cancer	1 (<0.1) (0.00, 0.15)	1 (<0.1) (0.00, 0.07)	2 (<0.1) (0.00, 0.06)	0	0	0	2 (<0.1) (0.00, 0.05)
Retained products of conception	0	1 (<0.1) (0.00, 0.07)	1 (<0.1) (0.00, 0.05)	0	0	0	1 (<0.1) (0.00, 0.04)
Road traffic accident	1 (<0.1) (0.00, 0.15)	0	1 (<0.1) (0.00, 0.05)	0	1 (0.1) (0.00, 0.34)	1 (<0.1) (0.00,	2 (<0.1) (0.00, 0.05)

						0.24)	
Ruptured ectopic pregnancy	1 (<0.1) (0.00, 0.15)	2 (<0.1) (0.01, 0.09)	3 (<0.1) (0.01, 0.08)	0	0	0	3 (<0.1) (0.01, 0.06)
Seizure	3 (0.1) (0.03, 0.24)	4 (0.1) (0.02, 0.13)	7 (0.1) (0.03, 0.12)	0	0	0	7 (<0.1) (0.02, 0.10)
Sepsis	1 (<0.1) (0.00, 0.15)	1 (<0.1) (0.00, 0.07)	2 (<0.1) (0.00, 0.06)	0	1 (0.1) (0.00, 0.34)	1 (<0.1) (0.00, 0.24)	3 (<0.1) (0.01, 0.06)
Skin injury	1 (<0.1) (0.00, 0.15)	0	1 (<0.1) (0.00, 0.05)	0	0	0	1 (<0.1) (0.00, 0.04)
Skin ulcer	0	1 (<0.1) (0.00, 0.07)	1 (<0.1) (0.00, 0.05)	0	0	0	1 (<0.1) (0.00, 0.04)
Small intestinal perforation	1 (<0.1) (0.00, 0.15)	0	1 (<0.1) (0.00, 0.05)	0	0	0	1 (<0.1) (0.00, 0.04)
Soft tissue injury	1 (<0.1) (0.00, 0.15)	0	1 (<0.1) (0.00, 0.05)	0	0	0	1 (<0.1) (0.00, 0.04)
Squamous cell carcinoma of the cervix	1 (<0.1) (0.00, 0.15)	0	1 (<0.1) (0.00, 0.05)	0	0	0	1 (<0.1) (0.00, 0.04)
Stab wound	0	2 (<0.1) (0.01, 0.09)	2 (<0.1) (0.00, 0.06)	0	2 (0.1) (0.03, 0.43)	2 (0.1) (0.02, 0.31)	4 (<0.1) (0.01, 0.07)
Status epilepticus	0	0	0	0	1 (0.1) (0.00, 0.34)	1 (<0.1) (0.00, 0.24)	1 (<0.1) (0.00, 0.04)
Stillbirth	0	1 (<0.1) (0.00, 0.07)	1 (<0.1) (0.00, 0.05)	0	0	0	1 (<0.1) (0.00, 0.04)
Subdural haematoma	0	1 (<0.1) (0.00, 0.07)	1 (<0.1) (0.00, 0.05)	0	0	0	1 (<0.1) (0.00, 0.04)
Substance-induced psychotic disorder	0	0	0	0	1 (0.1) (0.00, 0.34)	1 (<0.1) (0.00, 0.24)	1 (<0.1) (0.00, 0.04)
Syncope	1 (<0.1) (0.00, 0.15)	0	1 (<0.1) (0.00, 0.05)	0	0	0	1 (<0.1) (0.00, 0.04)
Syphilis	1 (<0.1) (0.00, 0.15)	0	1 (<0.1) (0.00, 0.05)	0	0	0	1 (<0.1) (0.00, 0.04)
Systemic lupus erythematosus	0	0	0	1 (0.2) (0.01, 0.87)	0	1 (<0.1) (0.00, 0.24)	1 (<0.1) (0.00, 0.04)
Thrombocytopenia	1 (<0.1) (0.00,	1 (<0.1) (0.00,	2 (<0.1) (0.00,	0	0	0	2 (<0.1) (0.00, 0.05)

	0.15)	0.07)	0.06)				
Thyroidectomy	0	1 (<0.1) (0.00, 0.07)	1 (<0.1) (0.00, 0.05)	0	0	0	1 (<0.1) (0.00, 0.04)
Tibia fracture	1 (<0.1) (0.00, 0.15)	0	1 (<0.1) (0.00, 0.05)	0	0	0	1 (<0.1) (0.00, 0.04)
Toxicity to various agents	0	0	0	0	1 (0.1) (0.00, 0.34)	1 (<0.1) (0.00, 0.24)	1 (<0.1) (0.00, 0.04)
Transient ischaemic attack	1 (<0.1) (0.00, 0.15)	1 (<0.1) (0.00, 0.07)	2 (<0.1) (0.00, 0.06)	0	0	0	2 (<0.1) (0.00, 0.05)
Tuberculosis gastrointestinal	1 (<0.1) (0.00, 0.15)	0	1 (<0.1) (0.00, 0.05)	0	0	0	1 (<0.1) (0.00, 0.04)
Tuberculous pleurisy	0	1 (<0.1) (0.00, 0.07)	1 (<0.1) (0.00, 0.05)	0	0	0	1 (<0.1) (0.00, 0.04)
Type IV hypersensitivity reaction	0	1 (<0.1) (0.00, 0.07)	1 (<0.1) (0.00, 0.05)	0	0	0	1 (<0.1) (0.00, 0.04)
Upper gastrointestinal haemorrhage	3 (0.1) (0.03, 0.24)	1 (<0.1) (0.00, 0.07)	4 (<0.1) (0.01, 0.09)	0	0	0	4 (<0.1) (0.01, 0.07)
Urinary retention	0	1 (<0.1) (0.00, 0.07)	1 (<0.1) (0.00, 0.05)	0	0	0	1 (<0.1) (0.00, 0.04)
Uterine leiomyoma	0	2 (<0.1) (0.01, 0.09)	2 (<0.1) (0.00, 0.06)	0	0	0	2 (<0.1) (0.00, 0.05)
Wrist fracture	0	0	0	1 (0.2) (0.01, 0.87)	0	1 (<0.1) (0.00, 0.24)	1 (<0.1) (0.00, 0.04)

Only serious adverse events (SAE) that occurred up till and including month 6 vaccination date or target month 6 date are shown and are sorted by descending overall preferred term frequency. An SAE was defined as: Any adverse event deemed serious by investigator or Sponsor based on the following criteria: death, a life-threatening adverse event, non-elective inpatient hospitalization, prolongation of existing non-elective hospitalization, persistent/significant incapacity, substantial disruption of the ability to conduct normal life functions, congenital anomaly/birth defect, or any other event considered medically important and thus serious even if it does not result in death, is not life-threatening, or does not require hospitalization. 95% CIs for adverse events are calculated using the score method of Agresti and Coull.
SARS2, SARS-CoV-2.

Table S11: Pregnancy Outcomes up until Month 6 Visit by Study Group, Full Analysis Set

Study Group	Ectopic pregnancy (N=2)	Full-term live birth (≥37 weeks) (N=87)	Premature live birth (<37 weeks) (N=10)	Spontaneous abortion (<20 weeks) (N=7)	Spontaneous fetal death and/or still birth (≥20 weeks) (N=3)	Therapeutic/elective abortion (N=10)	Congenital abnormality (N=0)
Study Group 1 (HIV+, Anti-S SARS2-) (N=3740)	2 (100.0%)	23 (26.4%)	2 (20.0%)	1 (14.3%)	0 (0.0%)	4 (40.0%)	0 (NA%)
Study Group 2 (HIV+, Anti-S SARS2+) (N=7941)	0 (0.0%)	53 (60.9%)	4 (40.0%)	5 (71.4%)	3 (100.0%)	6 (60.0%)	0 (NA%)
Study Group 3 (HIV-, Anti-S SARS2-) (N=648)	0 (0.0%)	3 (3.4%)	2 (20.0%)	0 (0.0%)	0 (0.0%)	0 (0.0%)	0 (NA%)
Study Group 4 (HIV-, Anti-S SARS2+) (N=1673)	0 (0.0%)	8 (9.2%)	2 (20.0%)	1 (14.3%)	0 (0.0%)	0 (0.0%)	0 (NA%)

There are 2587 participants assigned female sex at birth in Study Group 1, 6407 in Study Group 2, 208 in Study Group 3, and 909 in Study Group 4. Data on childbearing potential of these participants are not collected in this study. For participants reporting multiple pregnancy outcomes of the same type, the pregnancy outcome is counted only once.

Table S12. Number and incidence rates of Covid-19 endpoints based on the CDC case definition, including or excluding endpoints associated with NAAT results obtained at the month 1 vaccination visit, and the number and incidence rates of severe Covid-19 endpoints in the Full Analysis Set and the Per-Protocol cohort by analysis group.

	Full Analysis Set - N (rate per 100 person-years)			Per-Protocol cohort - N (rate per 100 person-years)		
Analysis Group	Covid-19 (CDC), including month 1 NAAT	Covid-19 (CDC), excluding month 1 NAAT	Severe Covid-19	Covid-19 (CDC), including month 1 NAAT	Covid-19 (CDC), excluding month 1 NAAT	Severe Covid-19
AG1 (PLWH, Vaccine Immunity) (HIV+, SARS2-, 2d)	119 (10.07)	76 (6.33)	5 (0.41)	68 (5.98)	68 (5.89)	3 (0.26)
AG2-1 (PLWH, Hybrid Immunity) (HIV+, SARS2+, 1d)	151 (3.92)	151 (3.92)	4 (0.10)	146 (3.86)	146 (3.86)	4 (0.11)
AG2-2 (HIV+, SARS2+, 2d)	39 (6.46)	28 (4.60)	2 (0.33)	20 (3.45)	21 (3.59)	1 (0.17)
AG3 (PLWoH, Vaccine Immunity) (HIV-, SARS2-, 2d)	14 (7.41)	10 (5.25)	2 (1.04)	8 (4.51)	8 (4.47)	2 (1.11)
AG4-1 (PLWoH, Hybrid Immunity) (HIV-, SARS2+, 1d)	28 (3.41)	28 (3.41)	2 (0.24)	28 (3.58)	28 (3.58)	2 (0.26)
AG4-2 (HIV-, SARS2+, 2d)	7 (5.56)	6 (4.75)	0 (0.00)	5 (4.23)	5 (4.22)	0 (0.00)
Total	358	299	15	275	276	12

NAAT, nucleic acid amplification test. PLWH, people living with HIV. PLWoH, people living without HIV. SARS2, SARS-CoV-2. 1d, one vaccine dose. 2d, two vaccine doses.

Table S13. Number and incidence rates of Covid-19 endpoints based on the COVE case definition, including or excluding endpoints associated with NAAT results obtained at the month 1 vaccination visit in the Full Analysis Set and the Per-Protocol cohort by analysis group.

	Full Analysis Set - N (rate per 100 person-years)		Per-Protocol cohort - N (rate per 100 person-years)	
Analysis Group	Covid-19 (COVE), including month 1 NAAT	Covid-19 (COVE), excluding month 1 NAAT	Covid-19 (COVE), including month 1 NAAT	Covid-19 (COVE), excluding month 1 NAAT
AG1 (PLWH, Vaccine Immunity) (HIV+, SARS2-, 2d)	30 (2.50)	30 (2.50)	22 (1.90)	22 (1.90)
AG2-1 (PLWH, Hybrid Immunity) (HIV+, SARS2+, 1d)	40 (1.04)	40 (1.04)	34 (0.90)	34 (0.90)
AG2-2 (HIV+, SARS2+, 2d)	14 (2.30)	14 (2.30)	10 (1.71)	10 (1.71)
AG3 (PLWoH, Vaccine Immunity) (HIV-, SARS2-, 2d)	4 (2.09)	4 (2.09)	3 (1.67)	3 (1.67)
AG4-1 (PLWoH, Hybrid Immunity) (HIV-, SARS2+, 1d)	5 (0.61)	5 (0.61)	5 (0.64)	5 (0.64)
AG4-2 (HIV-, SARS2+, 2d)	3 (2.37)	3 (2.37)	2 (1.69)	2 (1.69)
Total	96	96	76	76

NAAT, nucleic acid amplification test. PLWH, people living with HIV. PLWoH, people living without HIV. SARS2, SARS-CoV-2. 1d, one vaccine dose. 2d, two vaccine doses.

Table S14. Geographic distribution of identified subvariants of SARS-CoV-2 infections from nasal swabs collected at baseline and post-baseline visits throughout the study.

	B.1.1.529	BA.1.*	BA.2.*	BA.3.*	BA.4.*	BA.5.*	Lineage Unknown	Other	Recombinant	Total
Botswana	1 (14.3%)	1 (14.3%)	0 (0.0%)	0 (0.0%)	2 (28.6%)	1 (14.3%)	2 (28.6%)	0 (0.0%)	0 (0.0%)	7
Eswatini	2 (10.0%)	1 (5.0%)	6 (30.0%)	0 (0.0%)	2 (10.0%)	3 (15.0%)	4 (20.0%)	1 (5.0%)	1 (5.0%)	20
Kenya	0 (0.0%)	6 (7.7%)	24 (30.8%)	0 (0.0%)	11 (14.1%)	24 (30.8%)	10 (12.8%)	2 (2.6%)	1 (1.3%)	78
Malawi	0 (0.0%)	1 (2.0%)	19 (37.3%)	0 (0.0%)	8 (15.7%)	7 (13.7%)	7 (13.7%)	5 (9.8%)	4 (7.8%)	51
South Africa	30 (3.7%)	85 (10.4%)	235 (28.9%)	5 (0.6%)	94 (11.5%)	158 (19.4%)	128 (15.7%)	64 (7.9%)	15 (1.8%)	814
Uganda	0 (0.0%)	7 (10.1%)	15 (21.7%)	0 (0.0%)	16 (23.2%)	8 (11.6%)	10 (14.5%)	6 (8.7%)	7 (10.1%)	69
Zambia	0 (0.0%)	1 (1.4%)	9 (12.3%)	1 (1.4%)	19 (26.0%)	5 (6.8%)	12 (16.4%)	5 (6.8%)	21 (28.8%)	73
Total	33 (3.0%)	102 (9.2%)	308 (27.7%)	6 (0.5%)	152 (13.7%)	206 (18.5%)	173 (15.6%)	83 (7.5%)	49 (4.4%)	1112

Table S15. Number of baseline and post-baseline first occurrence of NAAT positive results, including or excluding those obtained at the month 1 vaccination visit, in the Full Analysis Set by the analysis group.

Analysis Group	Post-baseline NAAT +, including month 1 NAAT	Baseline and post-baseline NAAT+, including month 1 NAAT	Baseline and post-baseline NAAT +, excluding month 1 NAAT
AG1 (PLWH, Vaccine Immunity) (HIV+, SARS2-, 2d)	221	221	142
AG2-1 (PLWH, Hybrid Immunity) (HIV+, SARS2+, 1d)	261	601	601
AG2-2 (HIV+, SARS-CoV-2+, 2d)	51	334	317
AG3 (PLWoH, Vaccine Immunity) (HIV-, SARS2-, 2d)	27	27	16
AG4-1 (PLWoH, Hybrid Immunity) (HIV-, SARS2+, 1d)	56	132	132
AG4-2 (HIV-, SARS2+, 2d)	8	52	52
Total	624	1,367	1,260

NAAT, nucleic acid amplification test. PLWH, people living with HIV. PLWoH, people living without HIV. SARS2, SARS-CoV-2. 1d, one vaccine dose. 2d, two vaccine doses.

Supplemental Figures

Figure S1. Ubuntu CoVPN 3008 study flowcharts

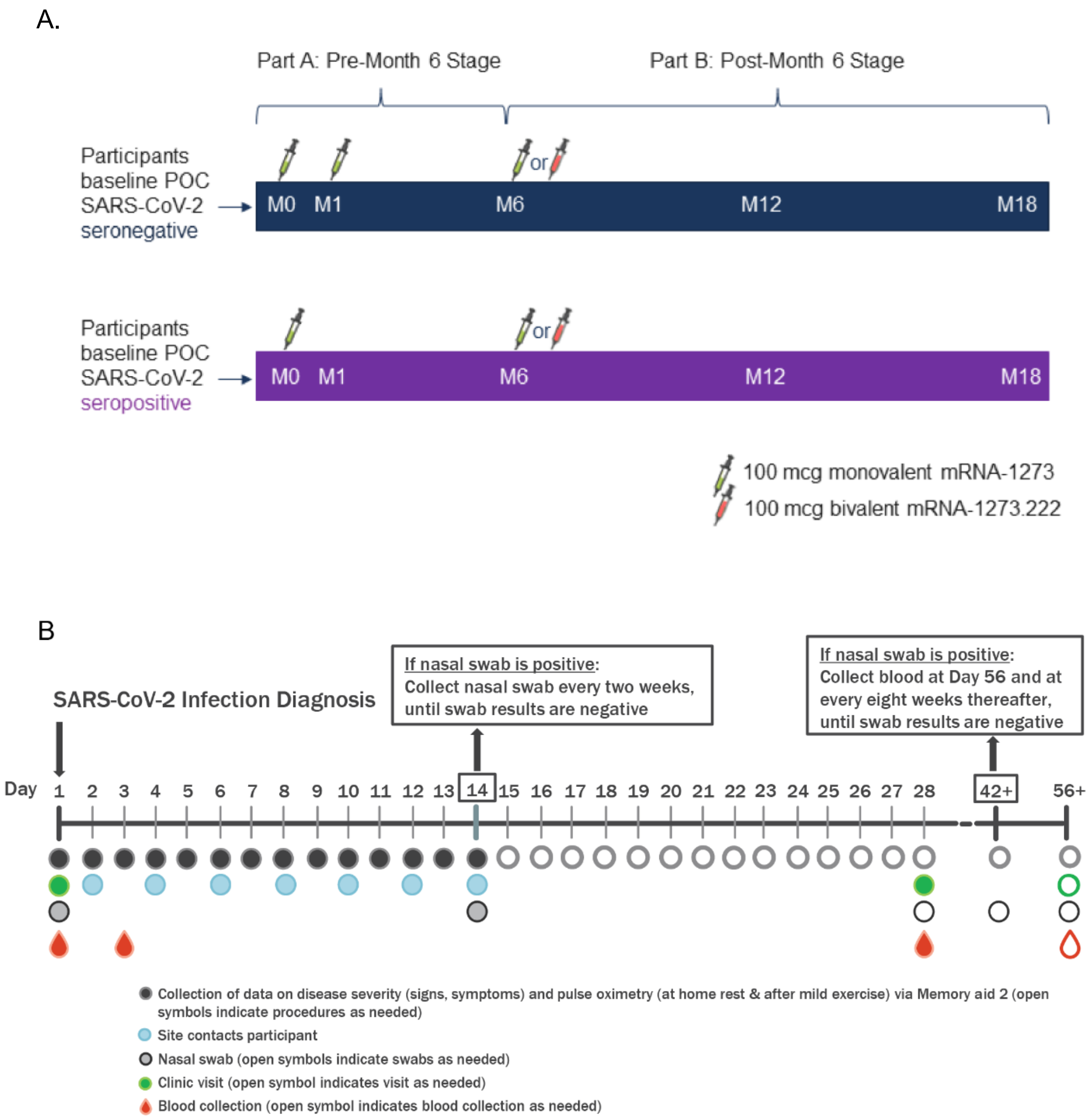

Figure S1. CoVPN 3008 (Ubuntu) study flowcharts. Panel A: The baseline anti-spike SARS-CoV-2 point-of-care serology (POC anti-S) result was used to assign the number of vaccinations participants would receive. Participants were assigned to receive one dose of mRNA-1273 at enrolment (M0) if their baseline POC anti-S was positive (hybrid immunity), or two doses of mRNA-1273 at M0 and month 1 (M1) if their baseline POC anti-S was negative (vaccine immunity). Note that baseline nasal swab SARS-CoV-2 nucleic acid amplification test (NAAT) and central lab serology test results were not generally available at the time of enrolment and did not influence group assignment but were later accounted for in the analyses. All participants were followed for 6 months after enrolment with periodic blood draws for immunogenicity characterization and nasal swabs for SARS-CoV-2 infection monitoring. **Panel B:** During the follow up, prespecified symptoms that met the criteria for suspicion of Covid-19 were solicited approximately every 2 weeks. Participants were also encouraged to report symptoms at any time. The presence of symptoms triggered a site visit to collect two nasal swabs within 72 hours for local

SARS-CoV-2 NAAT and, if positive, for quantification and whole genome sequencing at a central laboratory. Participants who tested positive by NAAT attended additional visits where symptoms, serum/peripheral blood mononuclear cells, and nasal swabs were collected.

Figure S2. Time to early termination by analysis group.

A

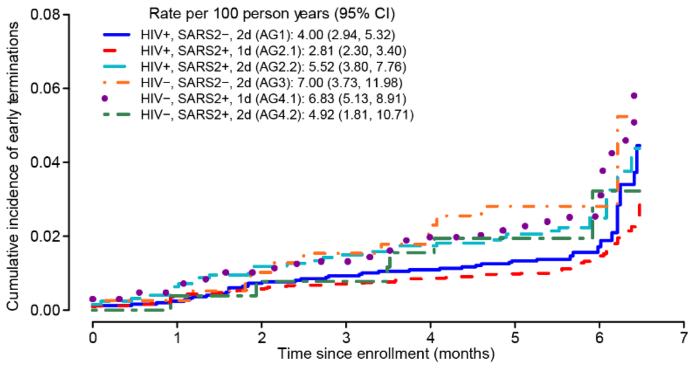

	0	1	2	3	4	5	6	7
**No. at Risk**								
HIV+, SARS2−, 2d (AG1)	2476	2470	2458	2453	2449	2438	583	0
HIV+, SARS2+, 1d (AG2.1)	7941	7910	7895	7885	7872	7841	1815	0
HIV+, SARS2+, 2d (AG2.2)	1264	1256	1249	1245	1242	1235	281	0
HIV−, SARS2−, 2d (AG3)	391	390	387	385	382	380	110	0
HIV−, SARS2+, 1d (AG4.1)	1672	1661	1655	1648	1637	1625	436	0
HIV−, SARS2+, 2d (AG4.2)	257	256	255	255	253	251	61	0
**Cumulative Early Termination**								
HIV+, SARS2−, 2d (AG1)	3	6	18	23	27	33	37	47
HIV+, SARS2+, 1d (AG2.1)	8	31	46	56	68	78	94	110
HIV+, SARS2+, 2d (AG2.2)	2	8	15	19	22	26	29	33
HIV−, SARS2−, 2d (AG3)	1	1	4	6	9	11	11	13
HIV−, SARS2+, 1d (AG4.1)	5	11	17	22	33	38	44	54
HIV−, SARS2+, 2d (AG4.2)	0	1	2	2	4	5	6	6

B

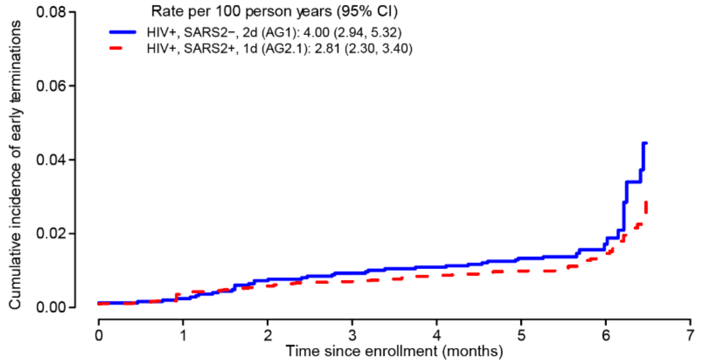

	0	1	2	3	4	5	6	7
**No. at Risk**								
HIV+, SARS2−, 2d (AG1)	2476	2470	2458	2453	2449	2438	583	0
HIV+, SARS2+, 1d (AG2.1)	7941	7910	7895	7885	7872	7841	1815	0
**Cumulative Early Termination**								
HIV+, SARS2−, 2d (AG1)	3	6	18	23	27	33	37	47
HIV+, SARS2+, 1d (AG2.1)	8	31	46	56	68	78	94	110

C

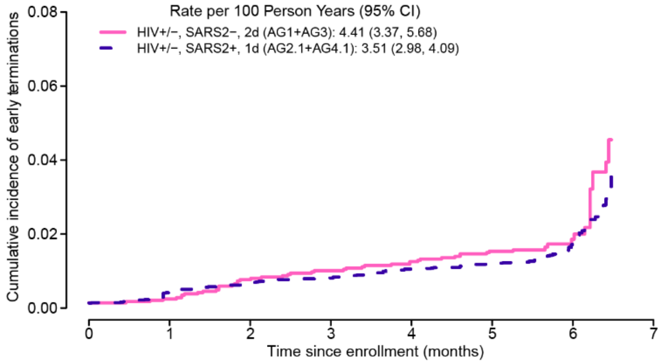

	0	1	2	3	4	5	6	7
**No. at Risk**								
HIV+/−, SARS2−, 2d (AG1+AG3)	2867	2860	2845	2838	2831	2818	693	0
HIV+/−, SARS2+, 1d (AG2.1+AG4.1)	9613	9571	9550	9533	9509	9466	2251	0
**Cumulative Early Termination**								
HIV+/−, SARS2−, 2d (AG1+AG3)	4	7	22	29	36	44	48	60
HIV+/−, SARS2+, 1d (AG2.1+AG4.1)	13	42	63	78	101	116	138	164

Figure S2. Time to early termination by analysis group. Panel A: Cumulative incidence of early termination for the two primary comparison analysis groups in people living with HIV (PLWH): vaccine

immunity (HIV+, SARS2-, 2d) and hybrid immunity (HIV+, SARS2+, 1d). **Panel B**: Cumulative incidence of early termination for the two secondary comparison analysis groups in PLWH and people living without HIV pooled: vaccine immunity (HIV+/-, SARS2-, 2d) and hybrid immunity (HIV+/-, SARS2+, 1d). **Panel C**: Cumulative incidence of early termination for each analysis group. Early termination in Part A is defined if a participant did not attend the month 6 visit and the early termination date on the termination form was before the upper visit window of the intended month 6 vaccination visit (i.e., Day 169 + 28 days).
SARS2, SARS-CoV-2. 1d, one vaccine dose. 2d, two vaccine doses.

Figure S3. Solicited local and systemic adverse events.

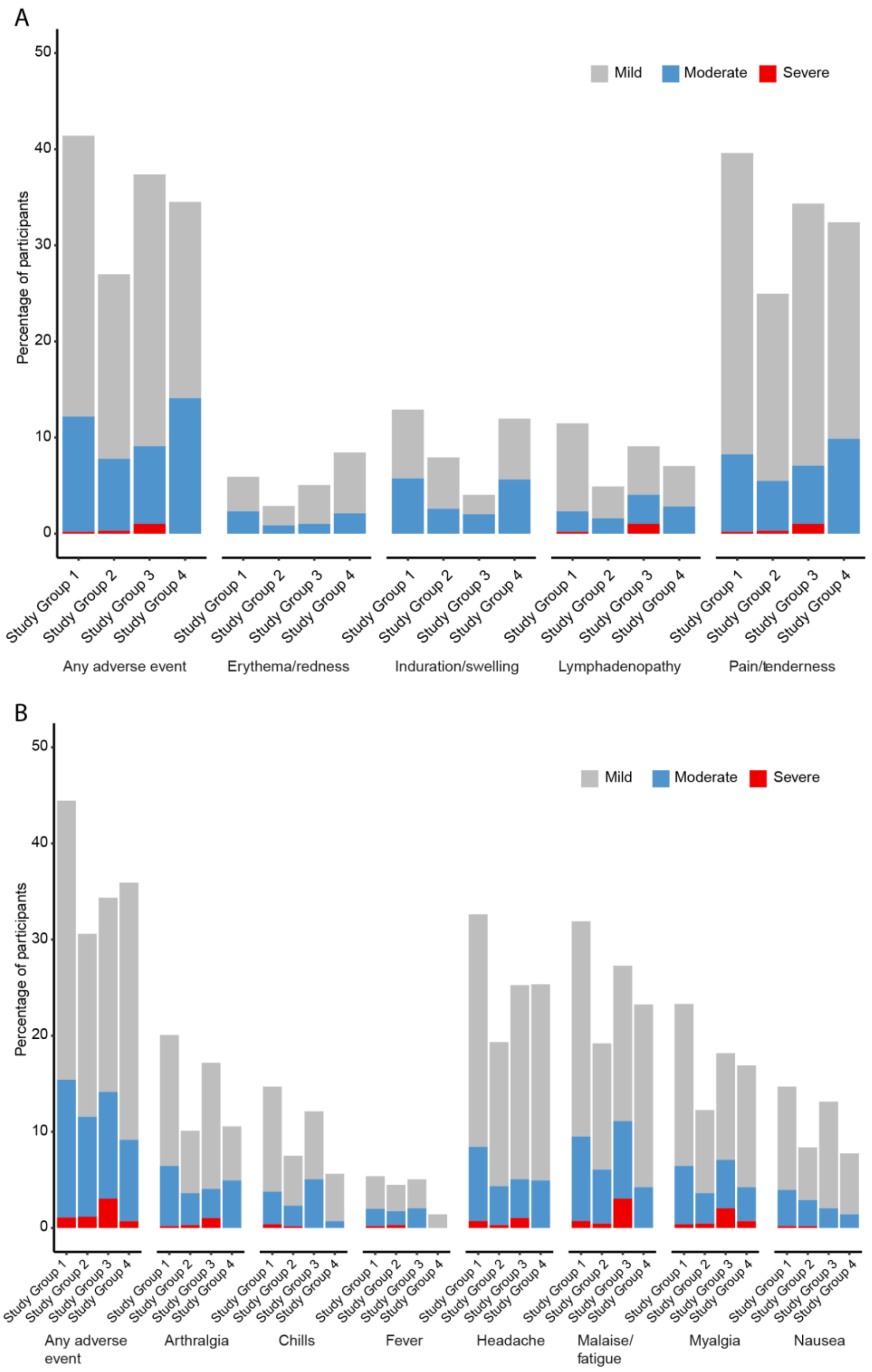

Figure S3. Solicited local and systemic adverse events. Shown is the percentage of participants in the Safety Subset who had a solicited local (**Panel A**) or systemic (**Panel B**) adverse event within 7 days after injection 1 or injection 2 of the mRNA-1273 vaccine.

Figure S4. Hybrid and vaccine immunity cumulative incidence of severe Covid-19 among people living with HIV in the Full Analysis Set.

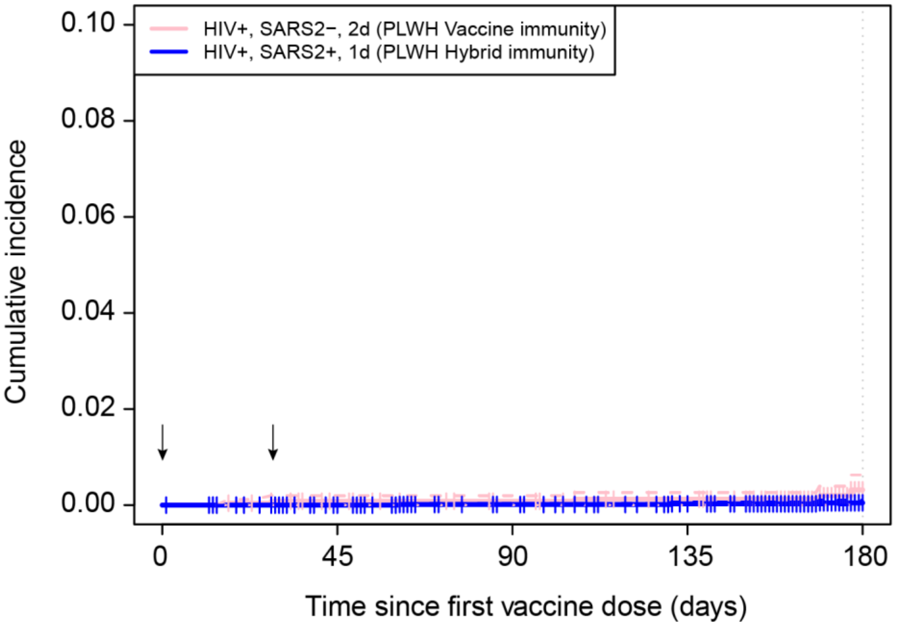

No. at Risk					
HIV+, SARS2−, 2d	2473	2463	2451	2444	714
HIV+, SARS2+, 1d	7933	7903	7883	7863	2185
**Total Events**					
HIV+, SARS2−, 2d	0	2	2	3	5
HIV+, SARS2+, 1d	0	0	1	2	3

Figure S4. Hybrid and vaccine immunity cumulative incidence of severe Covid-19 among people living with HIV in the Full Analysis Set. Shown is the cumulative incidence of severe Covid-19 events among people living with HIV (PLWH) in the Full Analysis Set (FAS) for the vaccine immunity group (orange) and the hybrid immunity group (blue), counting events starting 1 day after the first vaccination in FAS. To ensure stability of estimated standard error, pointwise and simultaneous intervals are reported starting 14 days after the first vaccination. The vaccine immunity group represents participants who were overall SARS-CoV-2 negative and received 2 doses of mRNA-1273 at enrolment and month 1. The hybrid immunity group represents participants who were overall SARS-CoV-2 positive and received 1 dose of mRNA-1273 at enrolment. Arrows along the x-axis indicate enrolment and month 1 vaccination visits, and the tick marks in the curves indicate censored data.
SARS2, SARS-CoV-2. 1d, one vaccine dose. 2d, two vaccine doses.

Figure S5. Number of SARS-CoV-2 nucleic acid amplification tests performed over study time.

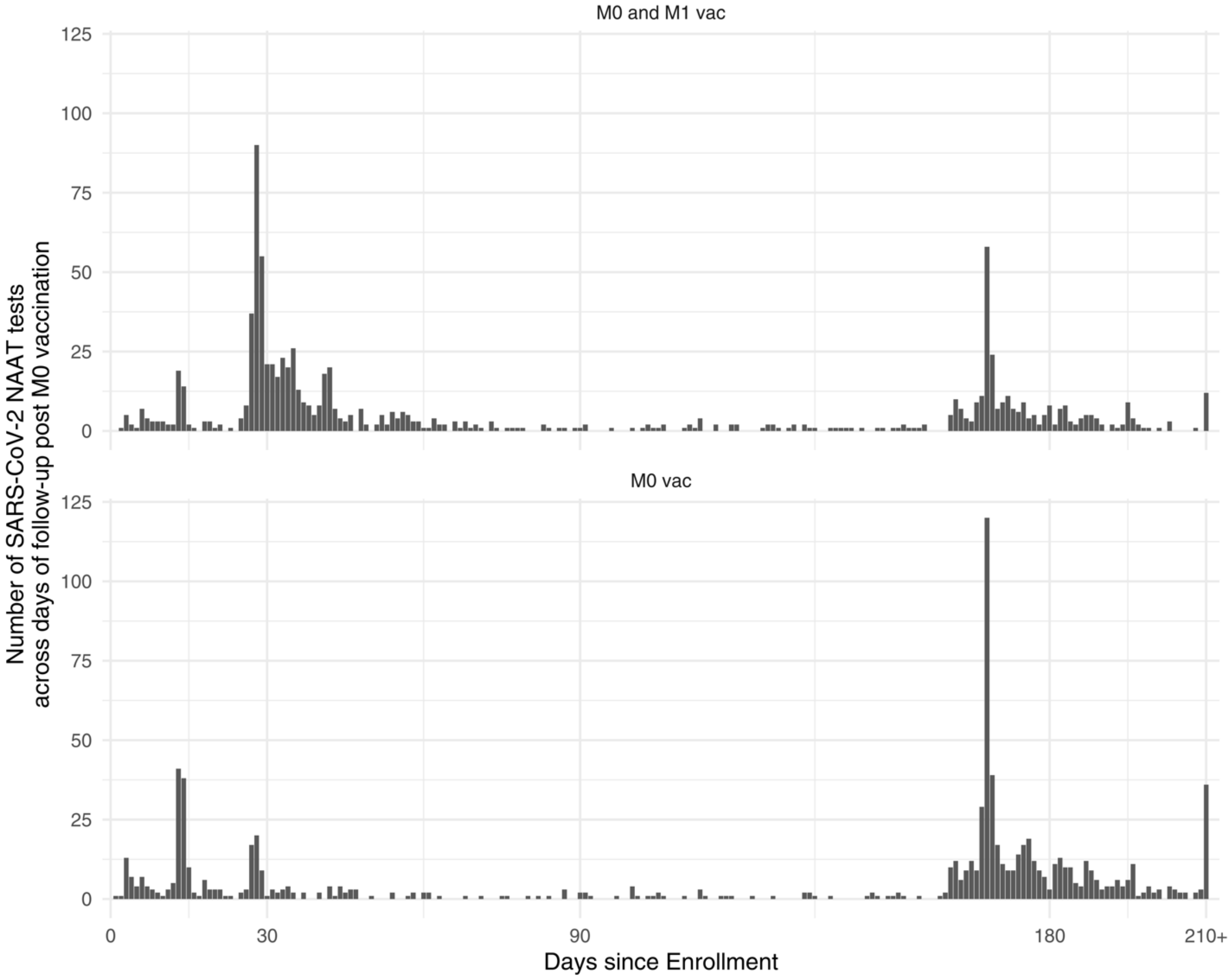

Figure S5. Number of SARS-CoV-2 nucleic acid amplification tests performed over study time. Shown are the number of positive SARS-CoV-2 nucleic acid amplification tests (NAATs), as scheduled at each vaccination or triggered by symptoms, performed on each study day post enrolment (not including enrolment or the first vaccination visit) for participants who received both enrolment (M0) and month 1 (M1) vaccinations (upper Panel) and participants who received only the enrolment vaccination (bottom panel) before month 6. This figure excludes positive NAAT results for those performed at the enrolment vaccination visits.

Figure S6. Association of hybrid versus vaccine immunity with SARS-CoV-2 nucleic acid amplification test positivity in the Full Analysis Set.

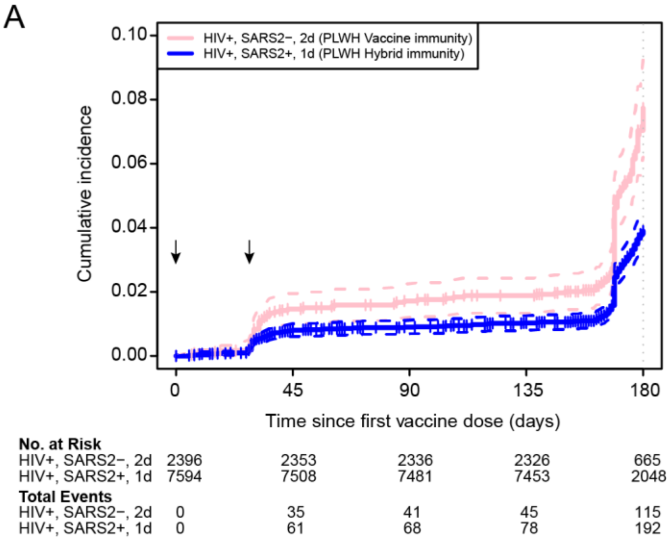

B

Endpoint Type	Analysis Group	No. of Endpoints	No. of Person-Years	Rate per 100 Person-Years
NAAT+	HIV+, SARS2-, 2d	220	1201	18.32
	HIV+, SARS2+, 1d	598	3857	15.50
	HIV+, SARS2+, 2d	333	610	54.59
	HIV-, SARS2-, 2d	27	191	14.14
	HIV-, SARS2+, 1d	131	823	15.92
	HIV-, SARS2+, 2d	52	126	41.27

Figure S6. Association of hybrid versus vaccine immunity with SARS-CoV-2 nucleic acid amplification test positivity in the Full Analysis Set. Shown is the cumulative incidence of post-baseline nucleic acid amplification test (NAAT) positive events (**Panel A**) with 95% confidence intervals based on assessment starting 1 day after the first vaccination in the Full Analysis Set (FAS) of persons living with HIV (PLWH). Arrows in both panels indicate enrolment and month 1, and the tick marks indicate censored data. Vaccine immunity group: overall SARS-CoV-2 negative and received mRNA-1273 at enrolment and month 1 (PLWH AG1); hybrid immunity group: overall SARS-CoV-2 positive and received mRNA-1273 at enrolment (PLWH AG2-1). **Panel B** shows the number and incidence rates of post-baseline NAAT positive events by the six analysis groups.
SARS2, SARS-CoV-2. 1d, one vaccine dose. 2d, two vaccine doses. AG, analysis group.

Figure S7. Association of hybrid versus vaccine immunity with Covid-19 (COVE) in the Full Analysis Set.

A

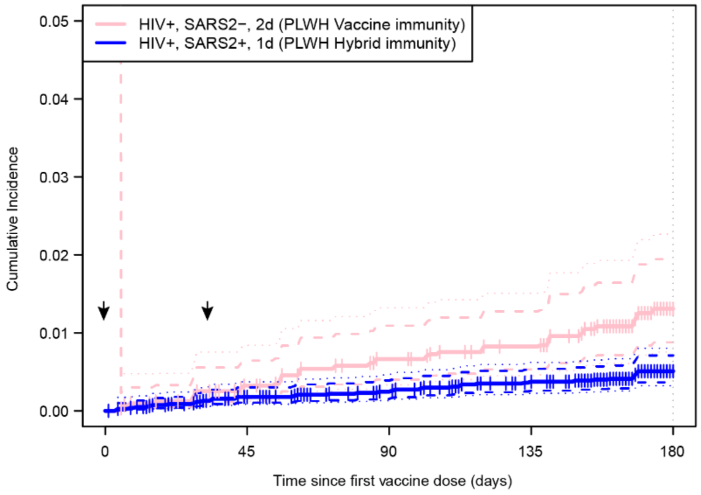

**No. at Risk**					
HIV+, SARS2−, 2d	2473	2456	2436	2425	704
HIV+, SARS2+, 1d	7933	7889	7865	7838	2172
**Total Events**					
HIV+, SARS2−, 2d	0	9	17	22	30
HIV+, SARS2+, 1d	0	14	19	29	37

B

	Endpoint Type	Comparison	No. of Endpoints	No. of Person-Years	Rate per 100 Person-Years	Hazard Ratio (95% CI)	P-value
B1	Covid-19 (COVE)	HIV+, SARS2-, 2d	30	1201	2.50		
		HIV+, SARS2+, 1d	40	3857	1.04	0.48 [0.29, 0.78]	0.003
B2	Covid-19 (COVE)	HIV+/-, SARS2-, 2d	34	1392	2.44		
		HIV+/-, SARS2+, 1d	45	4679	0.96	0.43 [0.27, 0.68]	< .001
B3	Covid-19 (COVE)	HIV-, SARS2-, 2d	4	191	2.09		
		HIV-, SARS2+, 1d	5	823	0.61	0.29 [0.08, 1.09]	0.067
	Covid-19 (COVE)	HIV+, SARS2-, 2d	30	1201	2.50		
		HIV-, SARS2-, 2d	4	191	2.09	0.62 [0.20, 1.88]	0.396
	Covid-19 (COVE)	HIV+, SARS2+, 1d	40	3857	1.04		
		HIV-, SARS2+, 1d	5	823	0.61	1.28 [0.49, 3.36]	0.621
	Covid-19 (COVE)	HIV+, SARS2+, 1d	40	3857	1.04		
		HIV+, SARS2+, 2d	14	610	2.30	0.53 [0.28, 0.98]	0.042
	Covid-19 (COVE)	HIV-, SARS2+, 1d	5	823	0.61		
		HIV-, SARS2+, 2d	3	126	2.37	0.30 [0.07, 1.31]	0.11
	Covid-19 (COVE)	HIV+, SARS2-, 2d	30	1201	2.50		
		HIV+, SARS2+, 2d	14	610	2.30	0.88 [0.46, 1.67]	0.698
	Covid-19 (COVE)	HIV-, SARS2-, 2d	4	191	2.09		
		HIV-, SARS2+, 2d	3	126	2.37	1.09 [0.24, 4.91]	0.915

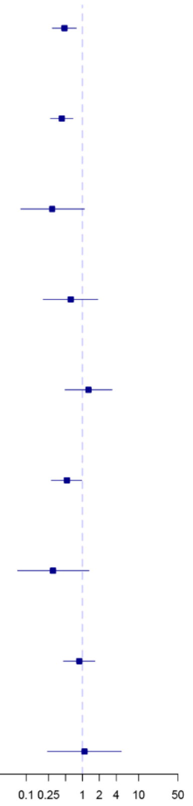

Figure S7. Association of hybrid versus vaccine immunity with Covid-19 (COVE) in the Full Analysis Set. Shown is the cumulative incidence of Covid-19 based on the COVE case definition (**Panel A**) with 95% confidence intervals based on assessment starting 1 day after the first vaccination in the Full Analysis Set (FAS) of persons living with HIV (PLWH). Arrows in both panels indicate enrolment and month 1, and the tick marks indicate censored data. Vaccine immunity group: overall SARS-CoV-2 negative and received mRNA-1273 at enrolment and month 1 (PLWH AG1); hybrid immunity group: overall SARS-CoV-2 positive and received mRNA-1273 at enrolment (PLWH AG2-1). **Panel B:** The forest plot shows hazard ratios of endpoints based on the COVE case definition for comparing FAS analysis groups with 95% confidence intervals estimated with the use of a Cox proportional hazards model with adjustment for baseline potential confounding variables (see Methods). The primary analysis (marked B1) compares PLWH and SARS-CoV-2- and 2 doses vs. PLWH and SARS-CoV-2+ and 1 dose (AG1 vs. AG2-1); the secondary analysis (marked B2) compares SARS-CoV-2- and 2 doses vs. SARS-CoV-2+ and 1 dose (AG1+AG3 pooled vs. AG2-2+AG4-2 pooled). The exploratory analyses (marked B3) compare seven other pairs of analysis groups. Incidence was defined as the number of events divided by the number of participants at risk and was adjusted by person-years.
SARS2, SARS-CoV-2. 1d, one vaccine dose. 2d, two vaccine doses. AG, analysis group.

Figure S8. Association of hybrid versus vaccine immunity with Covid-19 (COVE) in the Per-Protocol cohort.

A

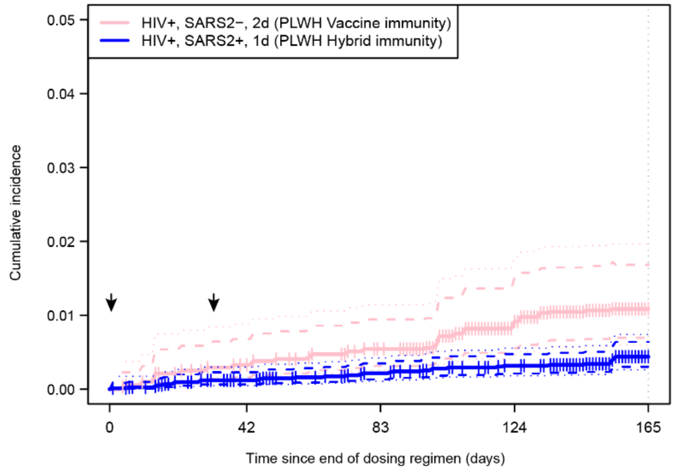

**No. at Risk**					
HIV+, SARS2−, 2d	2384	2370	2354	1680	116
HIV+, SARS2+, 1d	7824	7783	7763	7739	2159
**Total Events**					
HIV+, SARS2−, 2d	0	8	14	20	22
HIV+, SARS2+, 1d	1	9	16	24	31

B

	Endpoint Type	Comparison	No. of Endpoints	No. of Person-Years	Rate per 100 Person-Years	Hazard Ratio (95% CI)	P-value
B1	Covid-19 (COVE)	HIV+, SARS2-, 2d	22	1155	1.90		
		HIV+, SARS2+, 1d	34	3785	0.90	0.40 [0.23, 0.69]	0.001
B2	Covid-19 (COVE)	HIV+/-, SARS2-, 2d	25	1335	1.87		
		HIV+/-, SARS2+, 1d	39	4568	0.85	0.36 [0.21, 0.60]	< .001
B3	Covid-19 (COVE)	HIV-, SARS2-, 2d	3	179	1.67		
		HIV-, SARS2+, 1d	5	783	0.64	0.33 [0.08, 1.39]	0.13
	Covid-19 (COVE)	HIV+, SARS2-, 2d	22	1155	1.90		
		HIV-, SARS2-, 2d	3	179	1.67	0.48 [0.13, 1.72]	0.26
	Covid-19 (COVE)	HIV+, SARS2+, 1d	34	3785	0.90		
		HIV-, SARS2+, 1d	5	783	0.64	0.97 [0.36, 2.60]	0.953
	Covid-19 (COVE)	HIV+, SARS2+, 1d	34	3785	0.90		
		HIV+, SARS2+, 2d	10	585	1.71	0.47 [0.23, 0.96]	0.037
	Covid-19 (COVE)	HIV-, SARS2+, 1d	5	783	0.64		
		HIV-, SARS2+, 2d	2	119	1.69	0.41 [0.08, 2.23]	0.303
	Covid-19 (COVE)	HIV+, SARS2-, 2d	22	1155	1.90		
		HIV+, SARS2+, 2d	10	585	1.71	0.78 [0.37, 1.67]	0.523
	Covid-19 (COVE)	HIV-, SARS2-, 2d	3	179	1.67		
		HIV-, SARS2+, 2d	2	119	1.69	0.88 [0.14, 5.50]	0.891

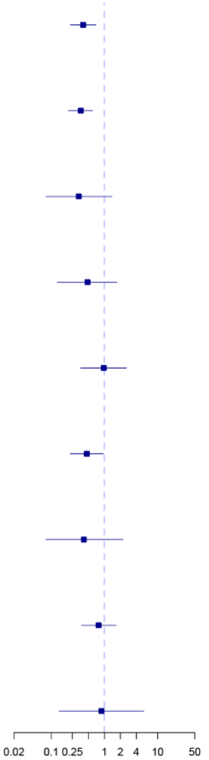

Figure S8. Association of hybrid versus vaccine immunity with Covid-19 (COVE) in the Per-Protocol cohort. This figure repeats the analysis of Figure S7 in the Per-Protocol (PP) cohort. Shown is the cumulative incidence of Covid-19 based on the COVE case definition (**Panel A**) with 95% confidence intervals based on assessment starting 14 days after the first or second vaccination in the PP set of persons living with HIV (PLWH). Arrows in both panels indicate enrolment and month 1, and the tick marks indicate censored data. Vaccine immunity group: overall SARS-CoV-2 negative and received mRNA-1273 at enrolment and month 1 (PLWH AG1); hybrid immunity group: overall SARS-CoV-2 positive and received mRNA-1273 at enrolment (PLWH AG2-1). **Panel B:** The forest plot shows hazard ratios of endpoints based on the COVE case definition for comparing PP analysis groups with 95% confidence intervals estimated with the use of a Cox proportional hazards model with adjustment for baseline potential confounding variables (see Methods). The primary analysis (marked B1) compares PLWH and SARS-CoV-2- and 2 doses vs. PLWH and SARS-CoV-2+ and 1 dose (AG1 vs. AG2-1); the secondary analysis (marked B2) compares SARS-CoV-2- and 2 doses vs. SARS-CoV-2+ and 1 dose (AG1+AG3 pooled vs. AG2-2+AG4-2 pooled). The exploratory analyses (marked B3) compare seven other pairs of analysis groups. Incidence was defined as the number of events divided by the number of participants at risk and was adjusted by person-years.
SARS2, SARS-CoV-2. 1d, one vaccine dose. 2d, two vaccine doses. AG, analysis group.

Figure S9. Association of hybrid versus vaccine immunity with Covid-19 (CDC) based on cumulative incidence analysis in the Full Analysis Set and the Per-Protocol cohort.

A

	Endpoint Type	Comparison	No. of Endpoints	No. of Person-Years	Rate per 100 Person-Years	Risk Ratio (95% CI)	P-value
A1	Covid-19 (CDC)	HIV+, SARS2-, 2d	76	1200	6.33		
		HIV+, SARS2+, 1d	151	3856	3.92	0.60 [0.41, 0.87]	0.008
A2	Covid-19 (CDC)	HIV+/-, SARS2-, 2d	86	1391	6.18		
		HIV+/-, SARS2+, 1d	179	4678	3.83	0.61 [0.43, 0.87]	0.007
A3	Covid-19 (CDC)	HIV-, SARS2-, 2d	10	191	5.25		
		HIV-, SARS2+, 1d	28	822	3.41	0.75 [0.31, 1.82]	0.526
	Covid-19 (CDC)	HIV+, SARS2-, 2d	119	1182	10.07		
		HIV-, SARS2-, 2d	14	189	7.41	1.70 [0.92, 3.15]	0.090
	Covid-19 (CDC)	HIV+, SARS2+, 1d	151	3856	3.92		
		HIV-, SARS2+, 1d	28	822	3.41	1.47 [0.83, 2.61]	0.183
	Covid-19 (CDC)	HIV+, SARS2+, 1d	151	3856	3.92		
		HIV+, SARS2+, 2d	28	609	4.60	0.80 [0.48, 1.32]	0.381
	Covid-19 (CDC)	HIV-, SARS2+, 1d	28	822	3.41		
		HIV-, SARS2+, 2d	6	126	4.75	0.74 [0.26, 2.11]	0.572
	Covid-19 (CDC)	HIV+, SARS2-, 2d	119	1182	10.07		
		HIV+, SARS2+, 2d	39	604	6.46	0.66 [0.44, 0.99]	0.045
	Covid-19 (CDC)	HIV-, SARS2-, 2d	14	189	7.41		
		HIV-, SARS2+, 2d	7	126	5.56	0.87 [0.32, 2.39]	0.786

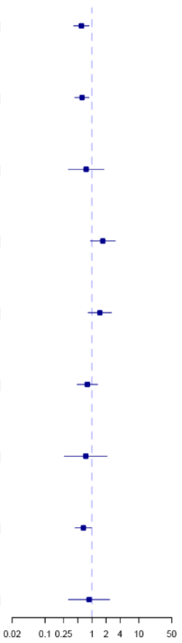

B

	Endpoint Type	Comparison	No. of Endpoints	No. of Person-Years	Rate per 100 Person-Years	Risk Ratio (95% CI)	P-value
B1	Covid-19 (CDC)	HIV+, SARS2-, 2d	68	1155	5.89		
		HIV+, SARS2+, 1d	146	3784	3.86	0.39 [0.21, 0.72]	0.002
B2	Covid-19 (CDC)	HIV+/-, SARS2-, 2d	76	1334	5.70		
		HIV+/-, SARS2+, 1d	174	4567	3.81	0.23 [0.08, 0.66]	0.006
B3	Covid-19 (CDC)	HIV-, SARS2-, 2d	8	179	4.47		
		HIV-, SARS2+, 1d	28	782	3.58	0.30 [0.14, 0.66]	0.003
	Covid-19 (CDC)	HIV+, SARS2-, 2d	68	1137	5.98		
		HIV-, SARS2-, 2d	8	177	4.51	2.05 [0.81, 5.21]	0.129
	Covid-19 (CDC)	HIV+, SARS2+, 1d	146	3784	3.86		
		HIV-, SARS2+, 1d	28	782	3.58	1.37 [0.76, 2.44]	0.292
	Covid-19 (CDC)	HIV+, SARS2+, 1d	146	3784	3.86		
		HIV+, SARS2+, 2d	21	584	3.59	1.25 [0.47, 3.28]	0.657
	Covid-19 (CDC)	HIV-, SARS2+, 1d	28	782	3.58		
		HIV-, SARS2+, 2d	5	119	4.22	0.49 [0.18, 1.35]	0.167
	Covid-19 (CDC)	HIV+, SARS2-, 2d	68	1137	5.98		
		HIV+, SARS2+, 2d	20	580	3.45	0.31 [0.16, 0.60]	<.001
	Covid-19 (CDC)	HIV-, SARS2-, 2d	8	177	4.51		
		HIV-, SARS2+, 2d	5	118	4.23	0.47 [0.08, 2.87]	0.416

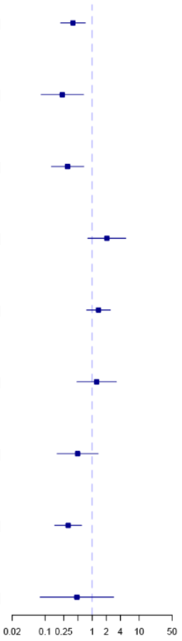

Figure S9. Association of hybrid versus vaccine immunity with Covid-19 (CDC) based on cumulative incidence analysis in the Full Analysis Set and the Per-Protocol cohort. The **Panel A** forest plot shows ratios of cumulative incidence of Covid-19 based on the CDC case definition by 6 months for comparing Full Analysis Set analysis groups with 95% confidence intervals with adjustment for baseline potential confounding variables (see Methods). The primary analysis (marked A1) compares PLWH and SARS-CoV-2- and 2 doses vs. PLWH and SARS-CoV-2+ and 1 dose (AG1 vs. AG2-1); the secondary analysis (marked A2) compares SARS-CoV-2- and 2 doses vs. SARS-CoV-2+ and 1 dose (AG1+AG3 pooled vs. AG2-2+AG4-2 pooled). The exploratory analyses (marked A3) compare seven other pairs of analysis groups. Incidence was defined as the number of events divided by the number of participants at risk and was adjusted by person-years. **Panel B:** The forest plot repeats the primary (B1), secondary (B2) and exploratory analyses (B3) in the Per-Protocol cohort.
SARS2, SARS-CoV-2. 1d, one vaccine dose. 2d, two vaccine doses. AG, analysis group.

Figure S10. Association of hybrid versus vaccine immunity with Covid-19 (COVE) based on cumulative incidence analysis in the Full Analysis Set and the Per-Protocol cohort.

A

	Endpoint Type	Comparison	No. of Endpoints	No. of Person-Years	Rate per 100 Person-Years	Risk Ratio (95% CI)	P-value
A1	Covid-19 (COVE)	HIV+, SARS2-, 2d	30	1201	2.50		
		HIV+, SARS2+, 1d	40	3857	1.04	0.39 [0.23, 0.65]	<.001
A2	Covid-19 (COVE)	SARS2-, 2d	34	1392	2.44		
		SARS2+, 1d	45	4679	0.96	0.39 [0.23, 0.64]	<.001
A3	Covid-19 (COVE)	HIV-, SARS2-, 2d	4	191	2.09		
		HIV-, SARS2+, 1d	5	823	0.61	0.40 [0.10, 1.63]	0.200
	Covid-19 (COVE)	HIV+, SARS2-, 2d	30	1201	2.50		
		HIV-, SARS2-, 2d	4	191	2.09	1.27 [0.44, 3.64]	0.656
	Covid-19 (COVE)	HIV+, SARS2+, 1d	40	3857	1.04		
		HIV-, SARS2+, 1d	5	823	0.61	1.36 [0.50, 3.72]	0.547
	Covid-19 (COVE)	HIV+, SARS2+, 1d	40	3857	1.04		
		HIV+, SARS2+, 2d	14	610	2.30	0.45 [0.24, 0.84]	0.012
	Covid-19 (COVE)	HIV-, SARS2+, 1d	5	823	0.61		
		HIV-, SARS2+, 2d	3	126	2.37	0.36 [0.08, 1.63]	0.183
	Covid-19 (COVE)	HIV+, SARS2-, 2d	30	1201	2.50		
		HIV+, SARS2+, 2d	14	610	2.30	0.87 [0.46, 1.65]	0.669
	Covid-19 (COVE)	HIV-, SARS2-, 2d	4	191	2.09		
		HIV-, SARS2+, 2d	3	126	2.37	1.11 [0.25, 4.96]	0.887

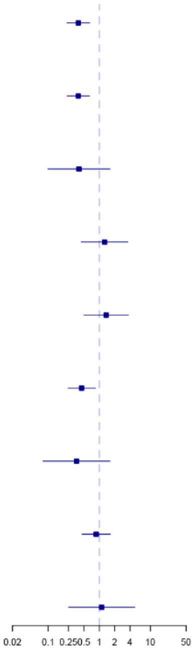

B

	Endpoint Type	Comparison	No. of Endpoints	No. of Person-Years	Rate per 100 Person-Years	Risk Ratio (95% CI)	P-value
B1	Covid-19 (COVE)	HIV+, SARS2-, 2d	22	1155	1.90		
		HIV+, SARS2+, 1d	34	3785	0.90	0.41 [0.23, 0.73]	0.002
B2	Covid-19 (COVE)	SARS2-, 2d	25	1335	1.87		
		SARS2+, 1d	39	4568	0.85	0.38 [0.22, 0.66]	<.001
B3	Covid-19 (COVE)	HIV-, SARS2-, 2d	3	179	1.67		
		HIV-, SARS2+, 1d	5	783	0.64	0.46 [0.10, 2.03]	0.302
	Covid-19 (COVE)	HIV+, SARS2-, 2d	22	1155	1.90		
		HIV-, SARS2-, 2d	3	179	1.67	1.29 [0.38, 4.32]	0.681
	Covid-19 (COVE)	HIV+, SARS2+, 1d	34	3785	0.90		
		HIV-, SARS2+, 1d	5	783	0.64	1.11 [0.40, 3.09]	0.844
	Covid-19 (COVE)	HIV+, SARS2+, 1d	34	3785	0.90		
		HIV+, SARS2+, 2d	10	585	1.71	0.48 [0.24, 0.96]	0.038
	Covid-19 (COVE)	HIV-, SARS2+, 1d	5	783	0.64		
		HIV-, SARS2+, 2d	2	119	1.69	0.45 [0.09, 2.24]	0.331
	Covid-19 (COVE)	HIV+, SARS2-, 2d	22	1155	1.90		
		HIV+, SARS2+, 2d	10	585	1.71	0.79 [0.37, 1.69]	0.539
	Covid-19 (COVE)	HIV-, SARS2-, 2d	3	179	1.67		
		HIV-, SARS2+, 2d	2	119	1.69	1.04 [0.17, 6.21]	0.967

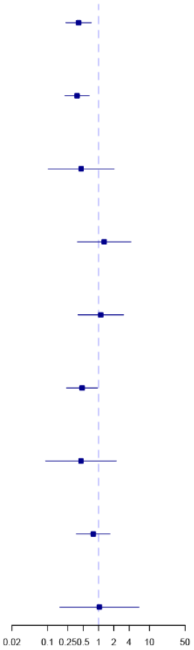

Figure S10. Association of hybrid versus vaccine immunity with Covid-19 (COVE) based on cumulative incidence analysis in the Full Analysis Set and the Per-Protocol cohort. The **Panel A** forest plot shows ratios of cumulative incidence of Covid-19 by 6 months for comparing Full Analysis Set analysis groups with 95% confidence intervals with adjustment for baseline potential confounding variables (see Methods). The primary analysis (marked A1) compares PLWH and SARS-CoV-2- and 2 doses vs. PLWH and SARS-CoV-2+ and 1 dose (AG1 vs. AG2-1); the secondary analysis (marked A2) compares SARS-CoV-2- and 2 doses vs. SARS-CoV-2+ and 1 dose (AG1+AG3 pooled vs. AG2-2+AG4-2 pooled). The exploratory analyses (marked A3) compare seven other pairs of analysis groups. Incidence was defined as the number of events divided by the number of participants at risk and was adjusted by person-years. **Panel B:** The forest plot repeats the primary (B1), secondary (B2) and exploratory analyses (B3) in the Per-Protocol cohort.
SARS2, SARS-CoV-2. 1d, one vaccine dose. 2d, two vaccine doses. AG, analysis group.

Figure S11. Association of hybrid versus vaccine immunity with Covid-19 (CDC) over time based on cumulative incidence analysis in the Full Analysis Set and the Per-Protocol cohort.

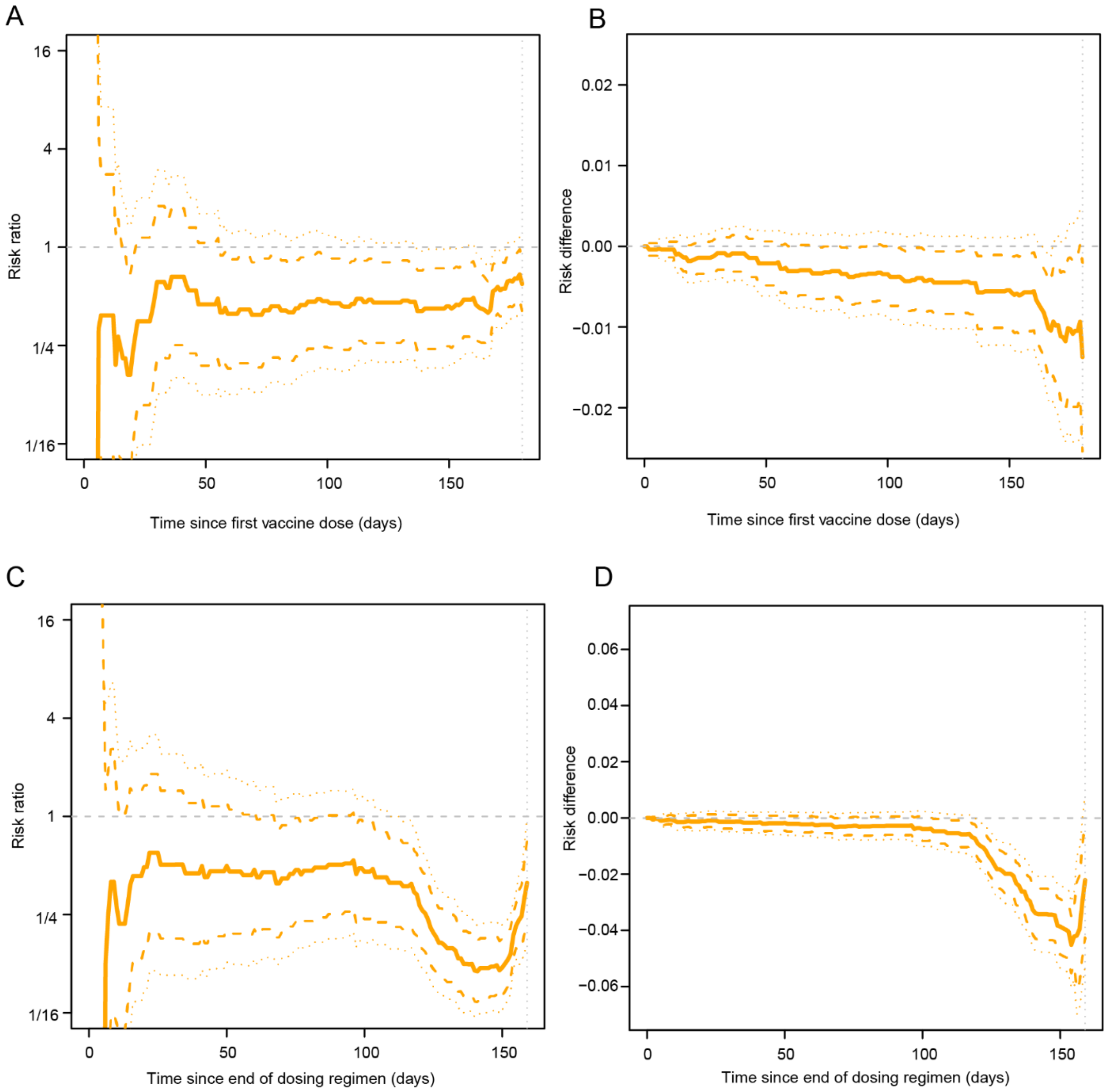

Figure S11. Association of hybrid versus vaccine immunity with Covid-19 (CDC) over time based on cumulative incidence analysis in the Full Analysis Set and the Per-Protocol cohort. Shown is the ratio (hybrid versus vaccine immunity, **Panel A**) or additive difference (hybrid minus vaccine immunity, **Panel B**) of cumulative incidence of Covid-19 (CDC case definition) over time starting post dose 1 in the Full Analysis Set in persons living with HIV (PLWH). **Panels C and D** show the results for the Per-Protocol set for Covid-19 starting 14 days after the first or second vaccination. Vaccine immunity group: overall SARS-CoV-2 negative and received mRNA-1273 at enrolment and month 1 (PLWH AG1); hybrid immunity group: overall SARS-CoV-2 positive and received mRNA-1273 at enrolment (PLWH AG2-1).
AG, analysis group.

Figure S12. Association of hybrid versus vaccine immunity with Covid-19 (COVE) over time based on cumulative incidence analysis in the Full Analysis Set and the Per-Protocol cohort.

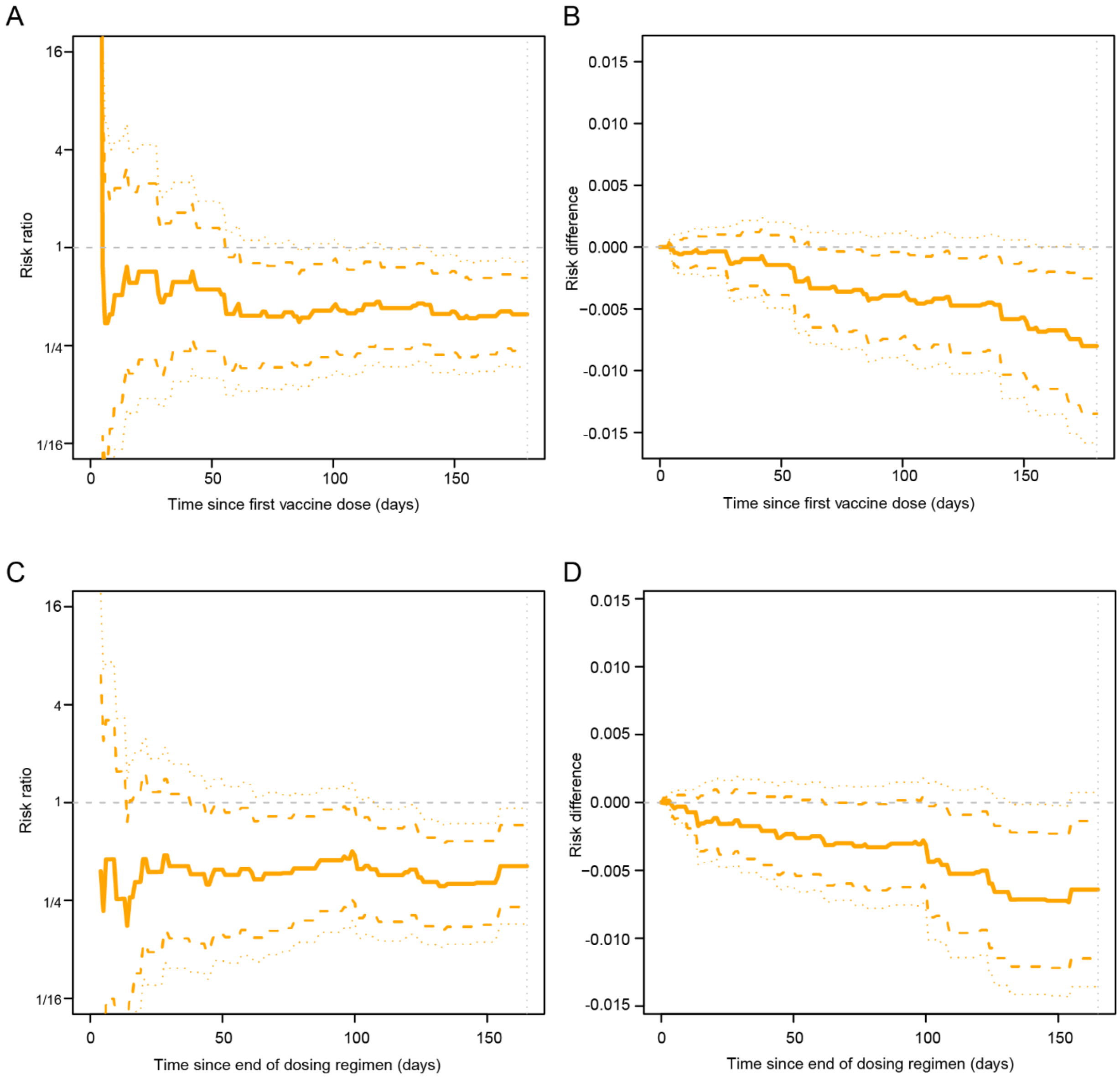

Figure S12. Association of hybrid versus vaccine immunity with Covid-19 (COVE) over time based on cumulative incidence analysis in the Full Analysis Set and the Per-Protocol cohort. Shown is the ratio (hybrid versus vaccine immunity, **Panel A**) or additive difference (hybrid minus vaccine immunity, **Panel B**) of cumulative incidence of Covid-19 (COVE) over time starting post dose 1 in the Full Analysis Set in persons living with HIV (PLWH). **Panels C and D** show the results for the Per-Protocol set for Covid-19 starting 14 days after the first or second vaccination. Vaccine immunity group: overall SARS-CoV-2 negative and received mRNA-1273 at enrolment and month 1 (PLWH AG1); hybrid immunity group: overall SARS-CoV-2 positive and received mRNA-1273 at enrolment (PLWH AG2-1).
AG, analysis group.

Figure S13. Sensitivity analysis of Cox proportional hazards results with alternative covariate adjustment strategies.

	Endpoint Type	Comparison	No. of Endpoints	No. of Person-Years	Rate per 100 Person-Years	Hazard Ratio (95% CI)	P-value
A	Covid-19 (COVE)	HIV+, SARS2-, 2d	30	1201	2.50		
		HIV+, SARS2+, 1d	40	3857	1.04	0.47 [0.29, 0.77]	0.003
	Covid-19 (CDC)	HIV+, SARS2-, 2d	76	1200	6.33		
		HIV+, SARS2+, 1d	151	3856	3.92	0.59 [0.44, 0.79]	< .001
B	Covid-19 (COVE)	SARS2-, 2d	34	1392	2.44		
		SARS2+, 1d	45	4679	0.96	0.43 [0.27, 0.67]	< .001
	Covid-19 (CDC)	SARS2-, 2d	86	1391	6.18		
		SARS2+, 1d	179	4678	3.83	0.56 [0.43, 0.73]	< .001
C	Covid-19 (COVE)	HIV+, SARS2-, 2d	22	1155	1.90		
		HIV+, SARS2+, 1d	34	3785	0.90	0.40 [0.23, 0.70]	0.001
	Covid-19 (CDC)	HIV+, SARS2-, 2d	68	1155	5.89		
		HIV+, SARS2+, 1d	146	3784	3.86	0.55 [0.41, 0.75]	< .001
D	Covid-19 (COVE)	SARS2-, 2d	25	1335	1.87		
		SARS2+, 1d	39	4568	0.85	0.35 [0.21, 0.59]	< .001
	Covid-19 (CDC)	SARS2-, 2d	76	1334	5.70		
		SARS2+, 1d	174	4567	3.81	0.53 [0.40, 0.70]	< .001

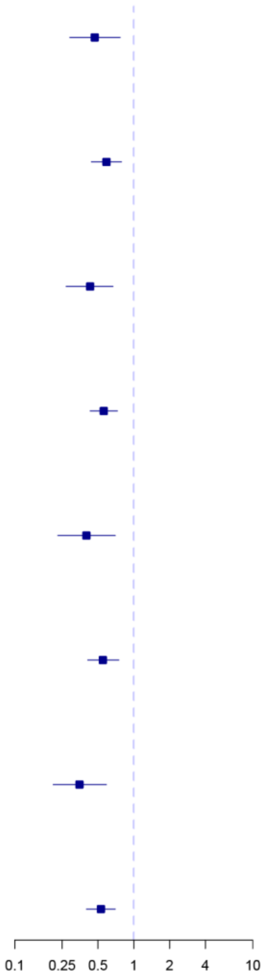

Figure S13. Sensitivity analysis of Cox proportional hazards results with alternative covariate adjustment strategies. This figure repeats the primary comparison analysis (AG1 vs. AG2-1) for Covid-19 (COVE) and for Covid-19 (CDC) in the Full Analysis Set (FAS) (**marked A**) and the Per-Protocol (PP) cohort (**marked C**) and the secondary comparison (AG1+AG3 pooled vs. AG2-2+AG4-2 pooled) in the FAS (**marked B**) and the PP cohort (**marked D**) based on a Cox proportional hazards model but with a different covariate adjustment strategy (see Methods).
SARS2, SARS-CoV-2. 1d, one vaccine dose. 2d, two vaccine doses. AG, analysis group.

Figure S14. Sensitivity analysis of cumulative incidence results with alternative covariate adjustment strategies.

	Endpoint Type	Comparison	No. of Endpoints	No. of Person-Years	Rate per 100 Person-Years	Risk Ratio (95% CI)	P-value
A	Covid-19 (COVE)	HIV+, SARS2-, 2d	30	1201	2.50		
		HIV+, SARS2+, 1d	40	3857	1.04	0.35 [0.20, 0.61]	<.001
	Covid-19 (CDC)	HIV+, SARS2-, 2d	76	1200	6.33		
		HIV+, SARS2+, 1d	151	3856	3.92	0.53 [0.36, 0.78]	0.001
B	Covid-19 (COVE)	SARS2-, 2d	34	1392	2.44		
		SARS2+, 1d	45	4679	0.96	0.31 [0.17, 0.57]	<.001
	Covid-19 (CDC)	SARS2-, 2d	86	1391	6.18		
		SARS2+, 1d	179	4678	3.83	0.52 [0.37, 0.75]	<.001
C	Covid-19 (COVE)	HIV+, SARS2-, 2d	22	1155	1.90		
		HIV+, SARS2+, 1d	34	3785	0.90	0.35 [0.18, 0.68]	0.002
	Covid-19 (CDC)	HIV+, SARS2-, 2d	68	1155	5.89		
		HIV+, SARS2+, 1d	146	3784	3.86	0.26 [0.16, 0.41]	<.001
D	Covid-19 (COVE)	SARS2-, 2d	25	1335	1.87		
		SARS2+, 1d	39	4568	0.85	0.35 [0.18, 0.66]	0.001
	Covid-19 (CDC)	SARS2-, 2d	76	1334	5.70		
		SARS2+, 1d	174	4567	3.81	0.24 [0.15, 0.39]	<.001

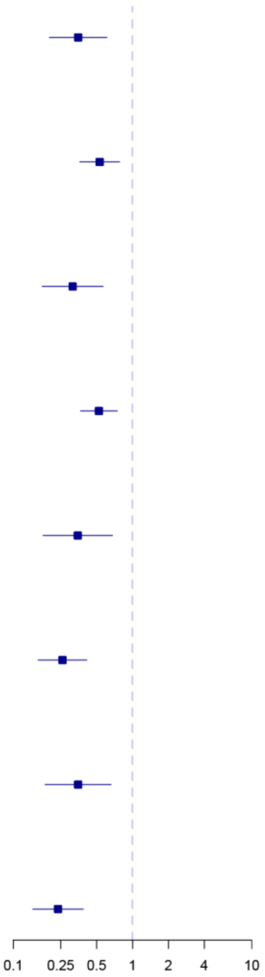

Figure S14. Sensitivity analysis of cumulative incidence results with alternative covariate adjustment strategies. This figure repeats the primary comparison analysis (AG1 vs. AG2-1) for Covid-19 (COVE) and for Covid-19 (CDC) in the Full Analysis Set (FAS) (**marked A**) and the Per-Protocol (PP) cohort (**marked C**) and the secondary comparison (AG1+AG3 pooled vs. AG2-2+AG4-2 pooled) in the FAS (**marked B**) and the PP cohort (**marked D**) based on a cumulative incidence approach but with a different covariate adjustment strategy (see Methods).

SARS2, SARS-CoV-2. 1d, one vaccine dose. 2d, two vaccine doses. AG, analysis group.

Figure S15. Association of hybrid versus vaccine immunity with the Covid-19 (CDC) over time based on cumulative incidence analysis and the secondary comparison pooling over people living with HIV and people living without HIV in the Full Analysis Set and the Per-Protocol cohort.

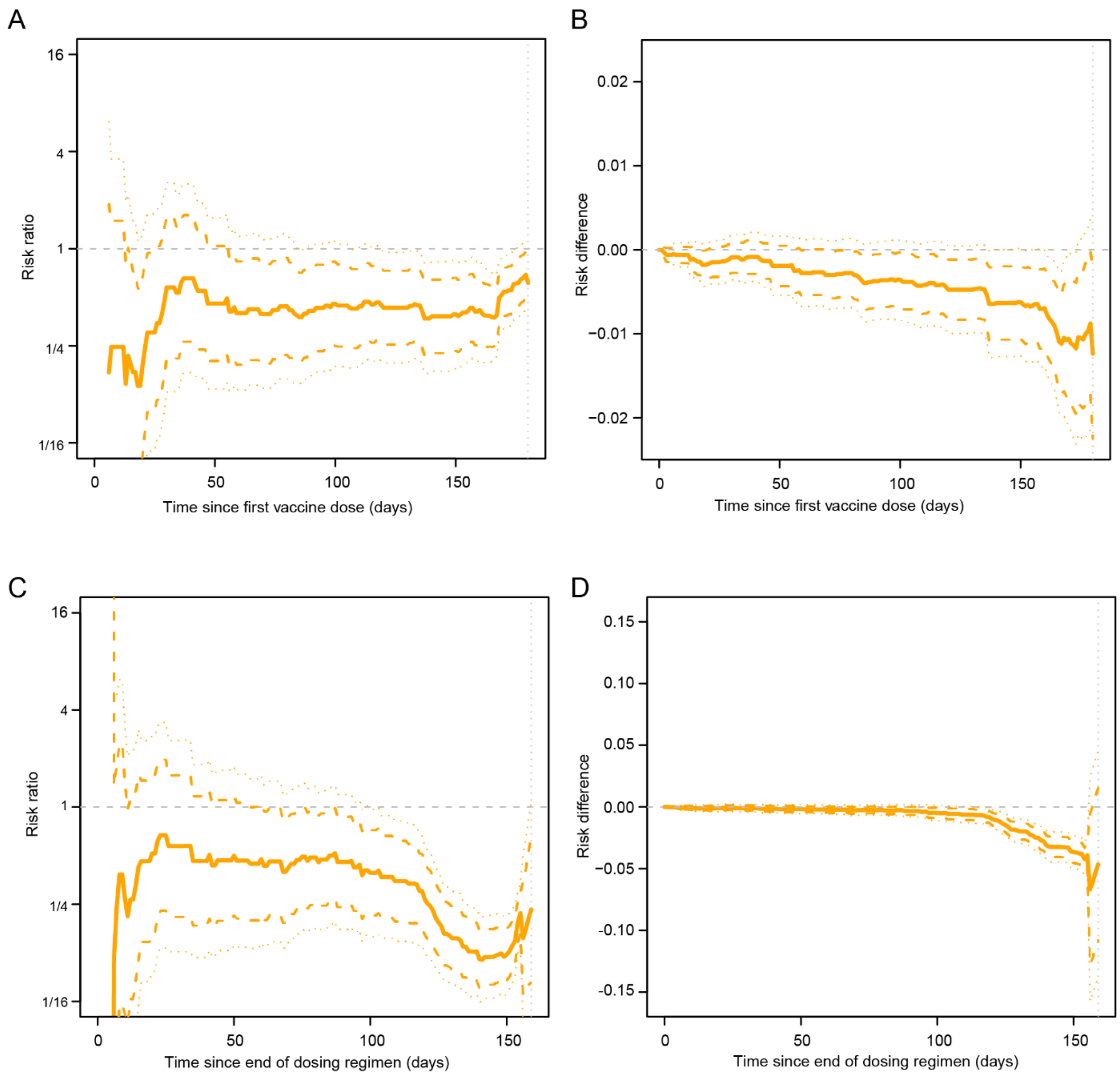

Figure S15. Association of hybrid versus vaccine immunity with the Covid-19 (CDC) over time based on cumulative incidence analysis and the secondary comparison pooling over people living with HIV and people living without HIV in the Full Analysis Set and the Per-Protocol cohort. Shown is the ratio (hybrid versus vaccine immunity, **Panel A**) or additive difference (hybrid minus vaccine immunity, **Panel B**) of cumulative incidence of Covid-19 based on the CDC case definition over time starting post dose 1 in Full Analysis Set for the secondary comparison. **Panels C and D** show the results in the Per-Protocol cohort for Covid-19 starting 14 days after the first or second vaccination. Arrows in both panels indicate enrolment and month 1, and the tick marks indicate censored data. Vaccine immunity group: overall SARS-CoV-2 negative and received mRNA-1273 at enrolment and month 1 (AG1+AG3 pooled); hybrid immunity group: overall SARS-CoV-2 positive and received mRNA-1273 at enrolment (AG2-2+AG4-2 pooled).
AG, analysis group.

Figure S16. Hybrid and vaccine immunity cumulative incidence of severe Covid-19 for the secondary comparison pooling over people living with HIV and people living without HIV in the Full Analysis Set and the Per-Protocol cohort.

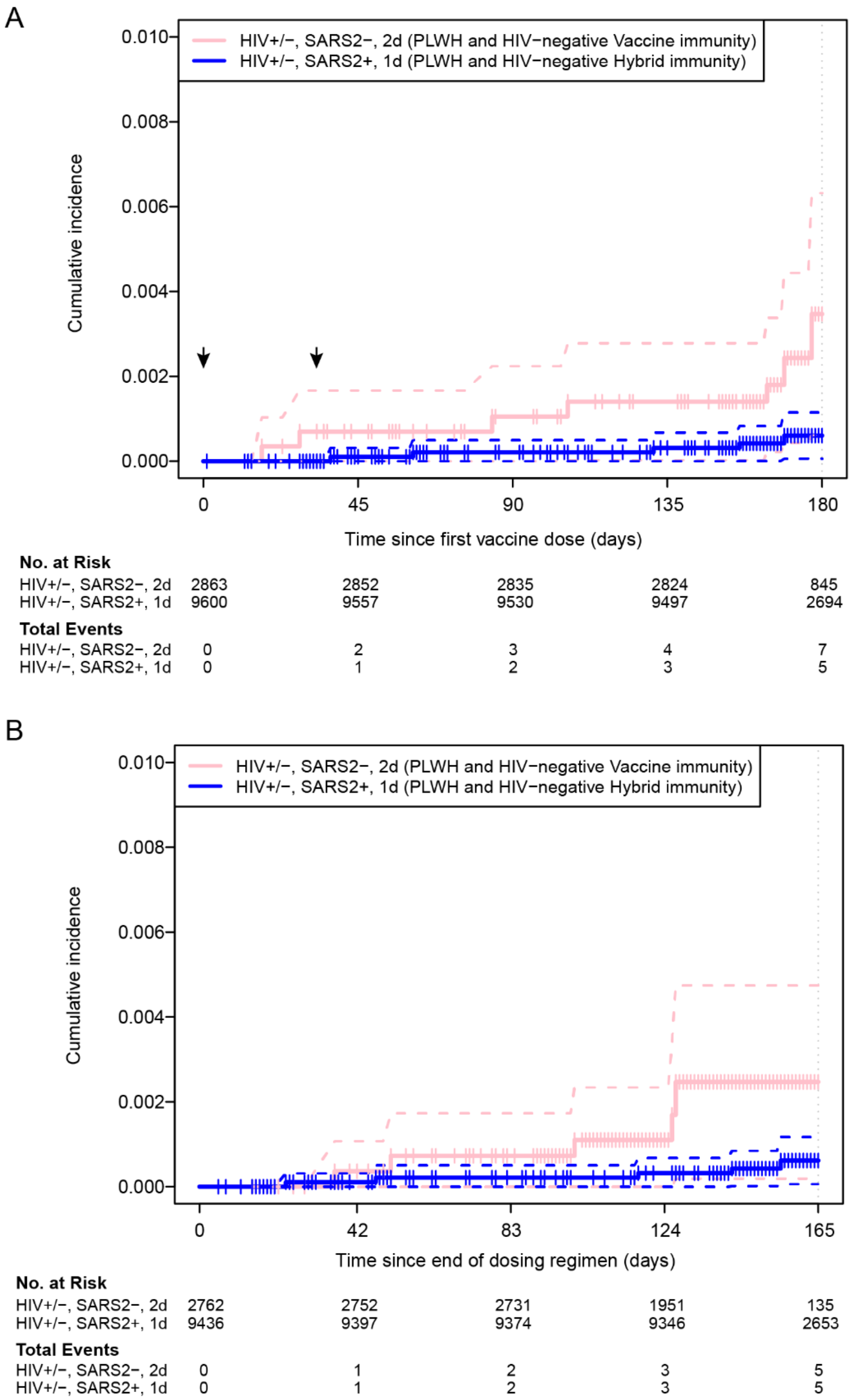

Figure S16. Hybrid and vaccine immunity cumulative incidence of severe Covid-19 for the secondary comparison pooling over people living with HIV and people living without HIV in the Full Analysis Set and the Per-Protocol cohort. Shown is the cumulative incidence of adjudicated severe Covid-19 with pointwise 95% confidence intervals based on the Full Analysis Set starting 1 day after first

vaccination (**Panel A**) and based on the Per-Protocol cohort starting 14 days after the first or second vaccination (**Panel B**), pooling over people living with HIV (PLWH) and people living without HIV. Arrows in both panels indicate enrolment and month 1, and the tick marks indicate censored data. Vaccine immunity group: overall SARS-CoV-2 negative and received mRNA-1273 at enrolment and month 1 (AG1+AG3 pooled); hybrid immunity group: overall SARS-CoV-2 positive and received mRNA-1273 at enrolment (AG2-2+AG4-2 pooled).
SARS2, SARS-CoV-2. 1d, one vaccine dose. 2d, two vaccine doses. AG, analysis group.

Figure S17. Hybrid and vaccine immunity cumulative incidence of Covid-19 (COVE) for the secondary comparison pooling over people living with HIV and people living without HIV.

A

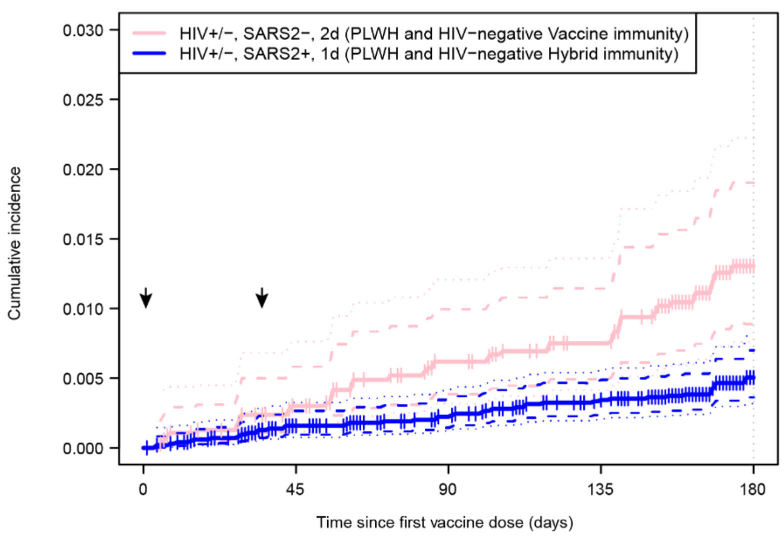

No. at Risk					
HIV+/−, SARS2−, 2d	2863	2844	2819	2804	833
HIV+/−, SARS2+, 1d	9600	9543	9511	9470	2679
**Total Events**					
HIV+/−, SARS2−, 2d	0	10	19	24	34
HIV+/−, SARS2+, 1d	0	15	21	32	42

B

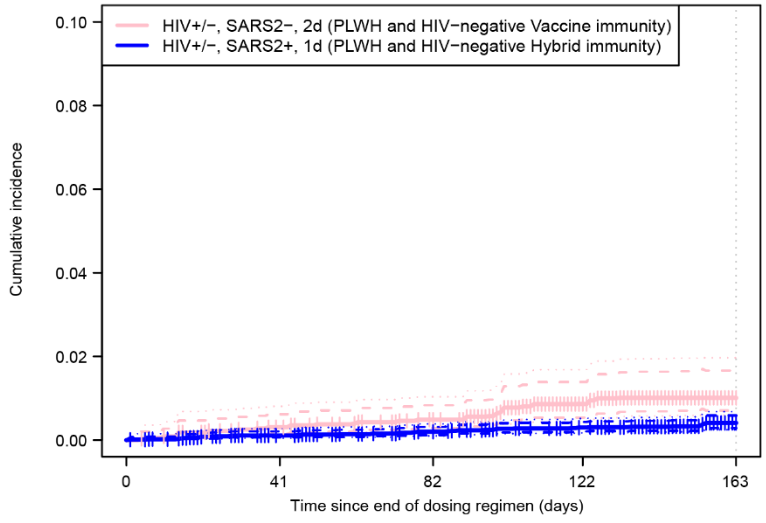

No. at Risk					
HIV+/−, SARS2−, 2d	2755	2737	2714	2063	145
HIV+/−, SARS2+, 1d	9431	9383	9355	9318	2912
**Total Events**					
HIV+/−, SARS2−, 2d	0	9	15	22	25
HIV+/−, SARS2+, 1d	1	10	18	27	35

Figure S17. Hybrid and vaccine immunity cumulative incidence of Covid-19 (COVE) for the secondary comparison pooling over people living with HIV and people living without HIV. Shown is the cumulative incidence of Covid-19 (COVE) with pointwise and simultaneous 95% confidence intervals

based on the Full Analysis Set starting 1 day after first vaccination (**Panel A**) and based on the Per-Protocol cohort starting 14 days after the first or second vaccination (**Panel B**), pooling over people living with HIV and people living without HIV. Arrows in both panels indicate enrolment and month 1, and the tick marks indicate censored data. Vaccine immunity group: overall SARS-CoV-2 negative and received mRNA-1273 at enrolment and month 1 (AG1+AG3 pooled); hybrid immunity group: overall SARS-CoV-2 positive and received mRNA-1273 at enrolment (AG2-2).
SARS2, SARS-CoV-2. 1d, one vaccine dose. 2d, two vaccine doses. AG, analysis group.

Figure S18. Association of hybrid versus vaccine immunity with Covid-19 (COVE) over time based on cumulative incidence analysis and the secondary comparison pooling over people living with HIV and people living without HIV in the Full Analysis Set and the Per-Protocol cohort.

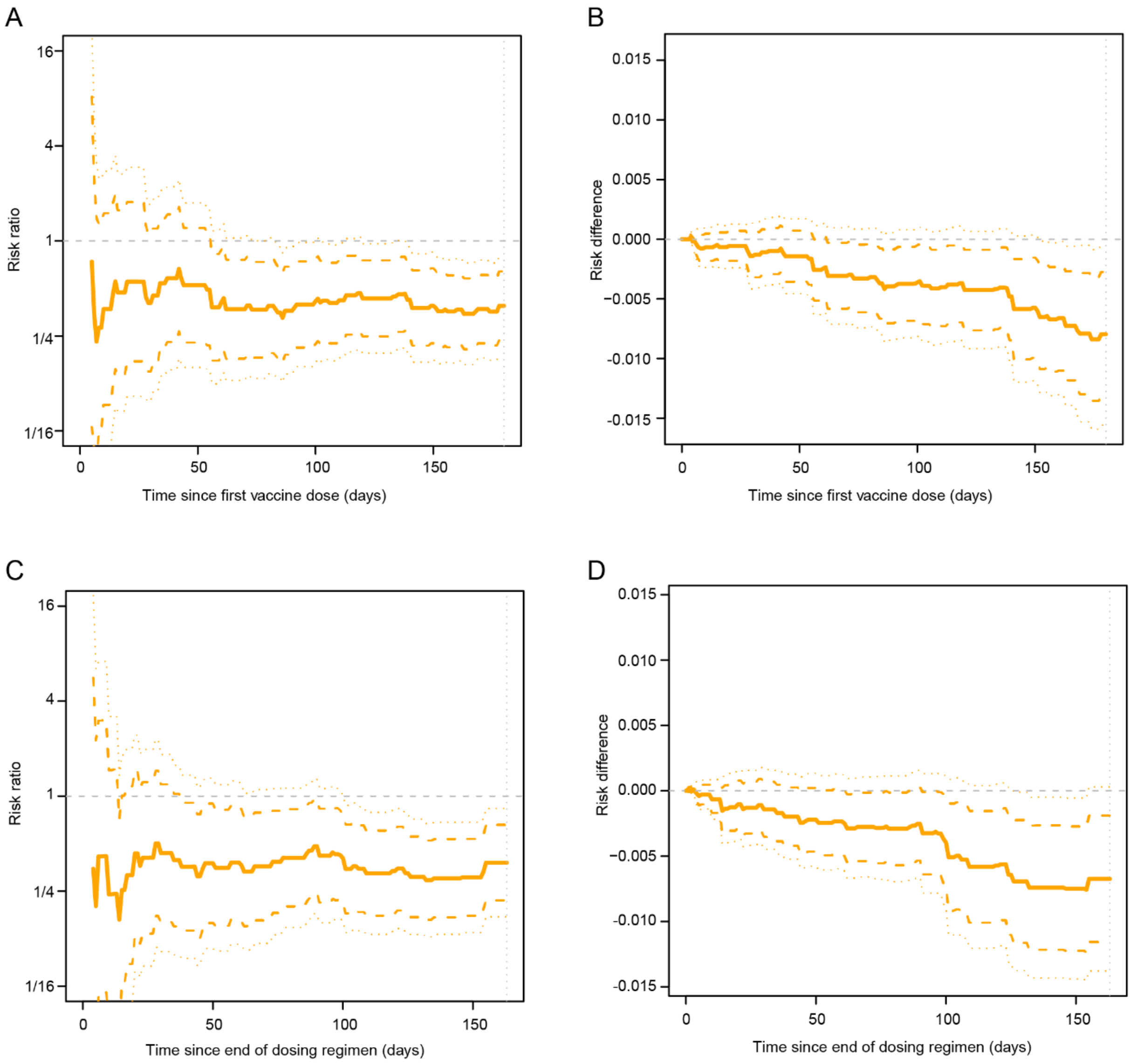

Figure S18. Association of hybrid versus vaccine immunity with Covid-19 (COVE) over time based on cumulative incidence analysis and the secondary comparison pooling over people living with HIV and people living without HIV in the Full Analysis Set and the Per-Protocol cohort. Shown is the ratio (hybrid versus vaccine immunity, **Panel A**) or additive difference (hybrid minus vaccine immunity, **Panel B**) of cumulative incidence of Covid-19 (COVE) over time starting post dose 1 in the Full Analysis Set for the secondary comparison. **Panels C and D** show the results for the Per-Protocol cohort for Covid-19 starting 14 days after the first or second vaccination. Vaccine immunity group: overall SARS-CoV-2 negative and received mRNA-1273 at enrolment and month 1 (AG1+AG3 pooled); hybrid immunity group: overall SARS-CoV-2 positive and received mRNA-1273 at enrolment (AG2-2+AG4-2 pooled).
AG, analysis group.

Figure S19. Seven exploratory comparisons of the risk of Covid-19 (CDC) based on the Cox models in the Full Analysis Set and the Per-Protocol cohort.

A

Endpoint Type	Comparison	No. of Endpoints	No. of Person-Years	Rate per 100 Person-Years	Hazard Ratio (95% CI)	P-value
Covid-19 (CDC)	HIV-, SARS2-, 2d	10	191	5.25		
	HIV-, SARS2+, 1d	28	822	3.41	0.49 [0.23, 1.05]	0.066
Covid-19 (CDC)	HIV+, SARS2-, 2d	119	1182	10.07		
	HIV-, SARS2-, 2d	14	189	7.41	0.94 [0.52, 1.70]	0.848
Covid-19 (CDC)	HIV+, SARS2+, 1d	151	3856	3.92		
	HIV-, SARS2+, 1d	28	822	3.41	0.97 [0.63, 1.50]	0.903
Covid-19 (CDC)	HIV+, SARS2+, 1d	151	3856	3.92		
	HIV+, SARS2+, 2d	28	609	4.60	0.73 [0.48, 1.10]	0.13
Covid-19 (CDC)	HIV-, SARS2+, 1d	28	822	3.41		
	HIV-, SARS2+, 2d	6	126	4.75	0.53 [0.21, 1.34]	0.179
Covid-19 (CDC)	HIV+, SARS2-, 2d	119	1182	10.07		
	HIV+, SARS2+, 2d	39	604	6.46	0.60 [0.42, 0.87]	0.007
Covid-19 (CDC)	HIV-, SARS2-, 2d	14	189	7.41		
	HIV-, SARS2+, 2d	7	126	5.56	0.76 [0.30, 1.94]	0.566

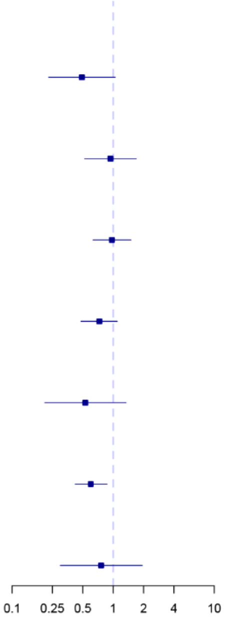

B

Endpoint Type	Comparison	No. of Endpoints	No. of Person-Years	Rate per 100 Person-Years	Hazard Ratio (95% CI)	P-value
Covid-19 (CDC)	HIV-, SARS2-, 2d	8	179	4.47		
	HIV-, SARS2+, 1d	28	782	3.58	0.55 [0.24, 1.25]	0.156
Covid-19 (CDC)	HIV+, SARS2-, 2d	68	1137	5.98		
	HIV-, SARS2-, 2d	8	177	4.51	0.89 [0.40, 1.94]	0.762
Covid-19 (CDC)	HIV+, SARS2+, 1d	146	3784	3.86		
	HIV-, SARS2+, 1d	28	782	3.58	0.88 [0.57, 1.36]	0.572
Covid-19 (CDC)	HIV+, SARS2+, 1d	146	3784	3.86		
	HIV+, SARS2+, 2d	21	584	3.59	0.79 [0.50, 1.26]	0.325
Covid-19 (CDC)	HIV-, SARS2+, 1d	28	782	3.58		
	HIV-, SARS2+, 2d	5	119	4.22	0.60 [0.23, 1.62]	0.316
Covid-19 (CDC)	HIV+, SARS2-, 2d	68	1137	5.98		
	HIV+, SARS2+, 2d	20	580	3.45	0.59 [0.36, 0.98]	0.04
Covid-19 (CDC)	HIV-, SARS2-, 2d	8	177	4.51		
	HIV-, SARS2+, 2d	5	118	4.23	1.07 [0.33, 3.47]	0.915

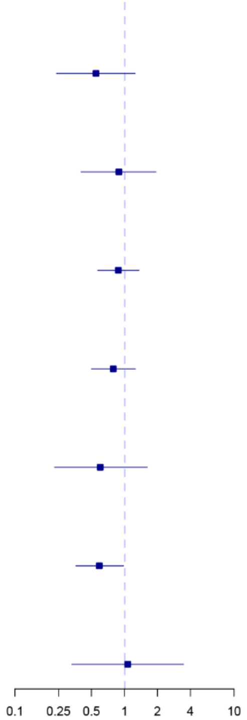

Figure S19. Seven exploratory comparisons of the risk of Covid-19 (CDC) based on the Cox models in the Full Analysis Set and the Per-Protocol cohort. The forest plot shows hazard ratios of Covid-19 based on the CDC case definition for comparing different groups in Full Analysis Set (**Panel A**) and in Per-Protocol cohort (**Panel B**). The hazard ratios with 95% confidence intervals were estimated using Cox proportional hazards models with adjustment for baseline potential confounding variables (see Methods). Incidence was defined as the number of events divided by the number of participants at risk and was adjusted by person-years.
SARS2, SARS-CoV-2. 1d, one vaccine dose. 2d, two vaccine doses.

Figure S20. Comparison of cumulative incidence of Covid-19 (CDC) between exploratory comparison groups HIV-, SARS-CoV-2-, 2 doses versus HIV+, SARS-CoV-2+, 1 dose.

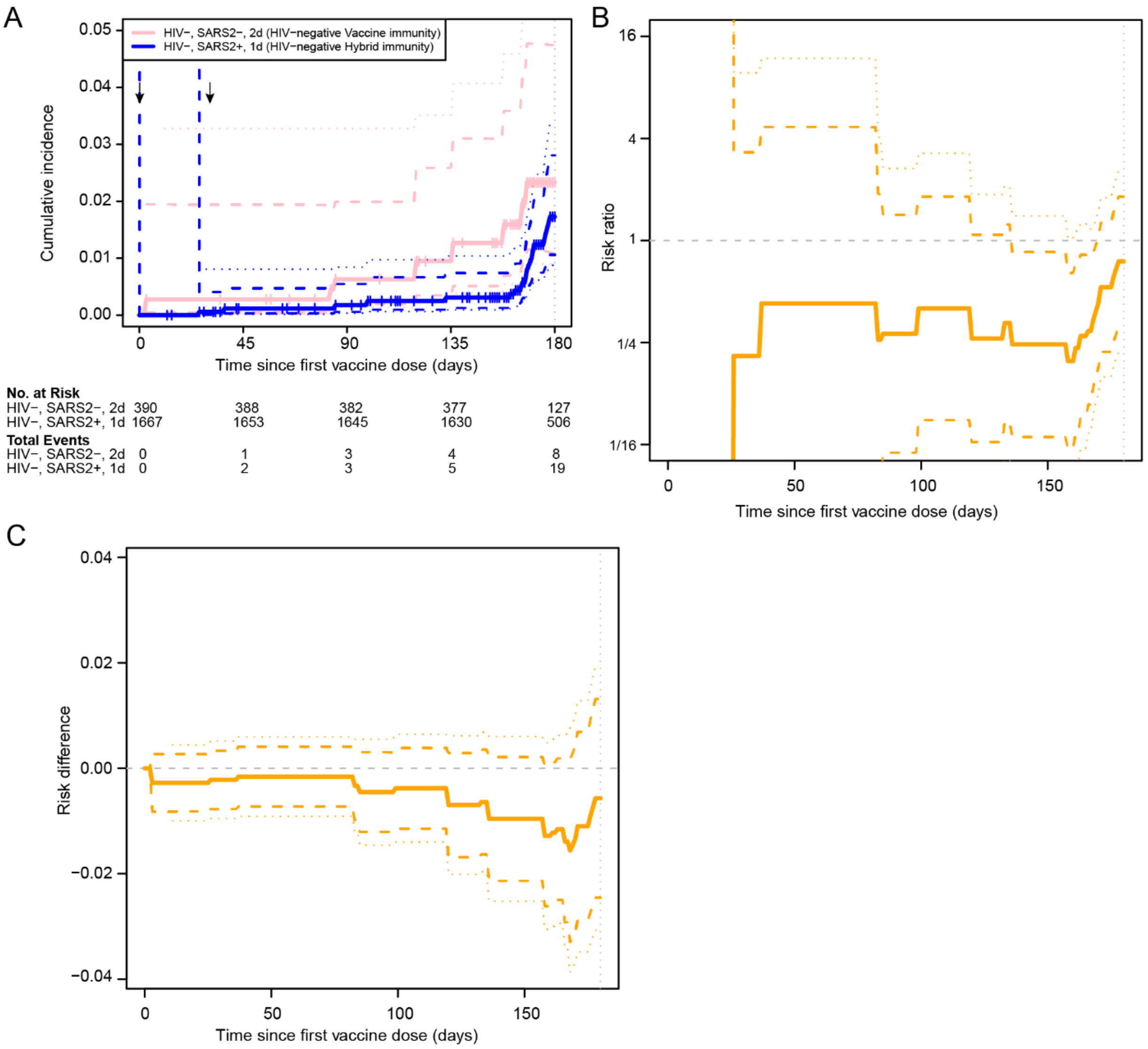

Figure S20. **Comparison of cumulative incidence of Covid-19 (CDC) between exploratory comparison groups HIV-, SARS-CoV-2-, 2 doses versus HIV-, SARS-CoV-2+, 1 dose.** Shown is the cumulative incidence of Covid-19 (CDC) with pointwise and simultaneous 95% confidence intervals based on the Full Analysis Set starting 1 day after first vaccination (**Panel A**) for HIV-, SARS-CoV-2-, 2 doses (AG-3) vs. HIV-, SARS-CoV-2+, 1 dose (AG4-1). **Panel B** shows the ratio (AG-3 vs. AG4-1) and **Panel C** the additive difference (AG-3 minus AG4-1) of cumulative incidence of Covid-19 (CDC) over time. Arrows in panels indicate enrolment and month 1, and the tick marks indicate censored data. Note, panel A is a duplicate of Figure 2D.
SARS2, SARS-CoV-2. 1d, one vaccine dose. 2d, two vaccine doses. AG, analysis group.

Figure S21. Comparison of cumulative incidence of Covid-19 (CDC) between exploratory comparison groups HIV+, SARS-CoV-2-, 2 doses versus HIV-, SARS-CoV-2-, 2 doses.

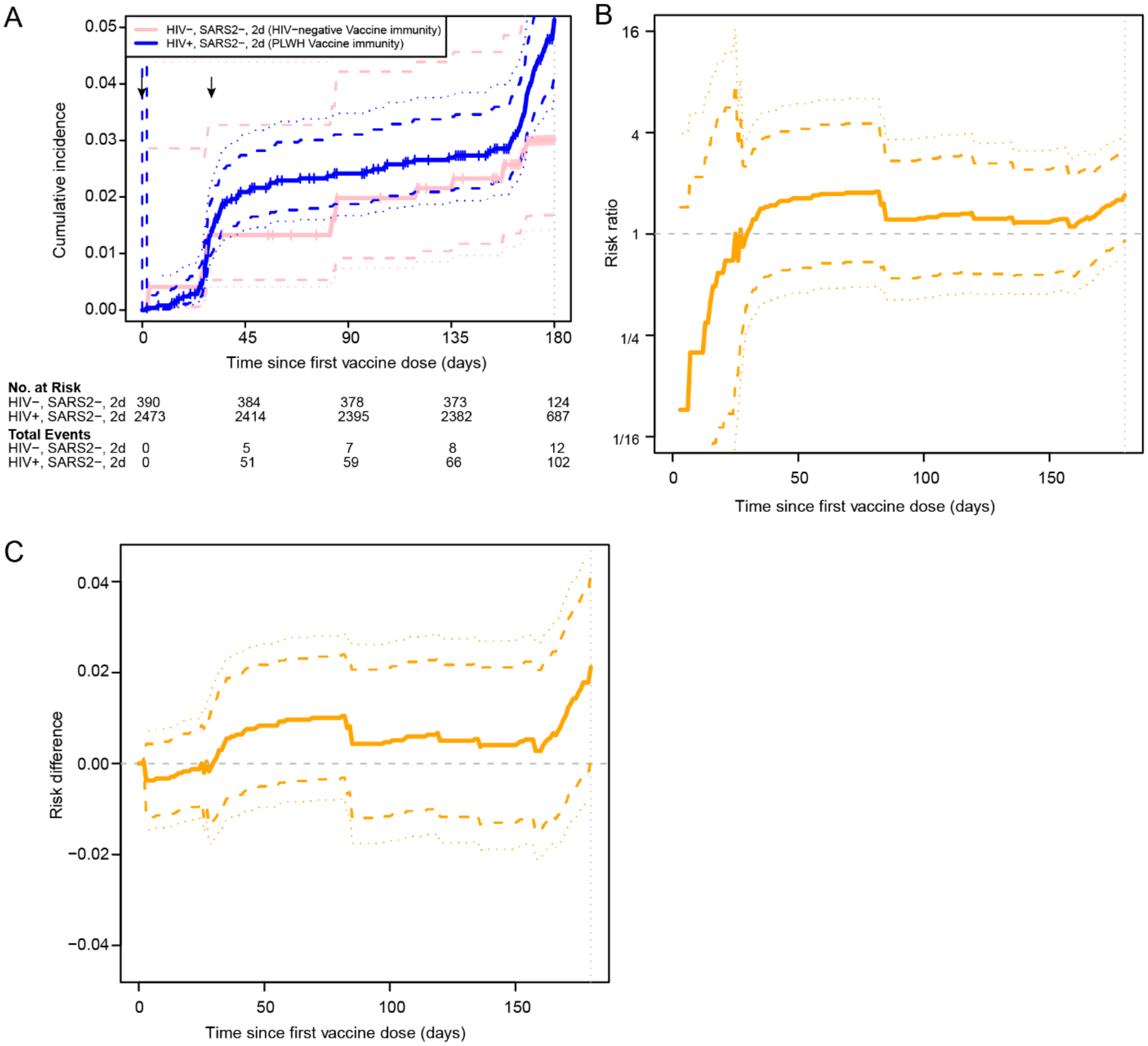

Figure S21. Comparison of cumulative incidence of Covid-19 (CDC) between exploratory comparison groups HIV+, SARS-CoV-2-, 2 doses versus HIV-, SARS-CoV-2-, 2 doses. Shown is the cumulative incidence of Covid-19 (CDC) with pointwise and simultaneous 95% confidence intervals based on the Full Analysis Set starting 1 day after first vaccination (**Panel A**) for HIV+, SARS-CoV-2-, 2 doses (AG-1) vs. HIV-, SARS-CoV-2-, 2 doses (AG3). **Panel B** shows the ratio (AG-1 vs. AG3) and **Panel C** the additive difference (AG-1 minus AG3) of cumulative incidence of Covid-19 (CDC) over time. Arrows in panels indicate enrolment and month 1, and the tick marks indicate censored data. SARS2, SARS-CoV-2. 1d, one vaccine dose. 2d, two vaccine doses. AG, analysis group.

Figure S22. Comparison of cumulative incidence of Covid-19 (CDC) between exploratory comparison groups HIV+, SARS-CoV-2+, 1 dose versus HIV-, SARS-CoV-2+, 1 dose.

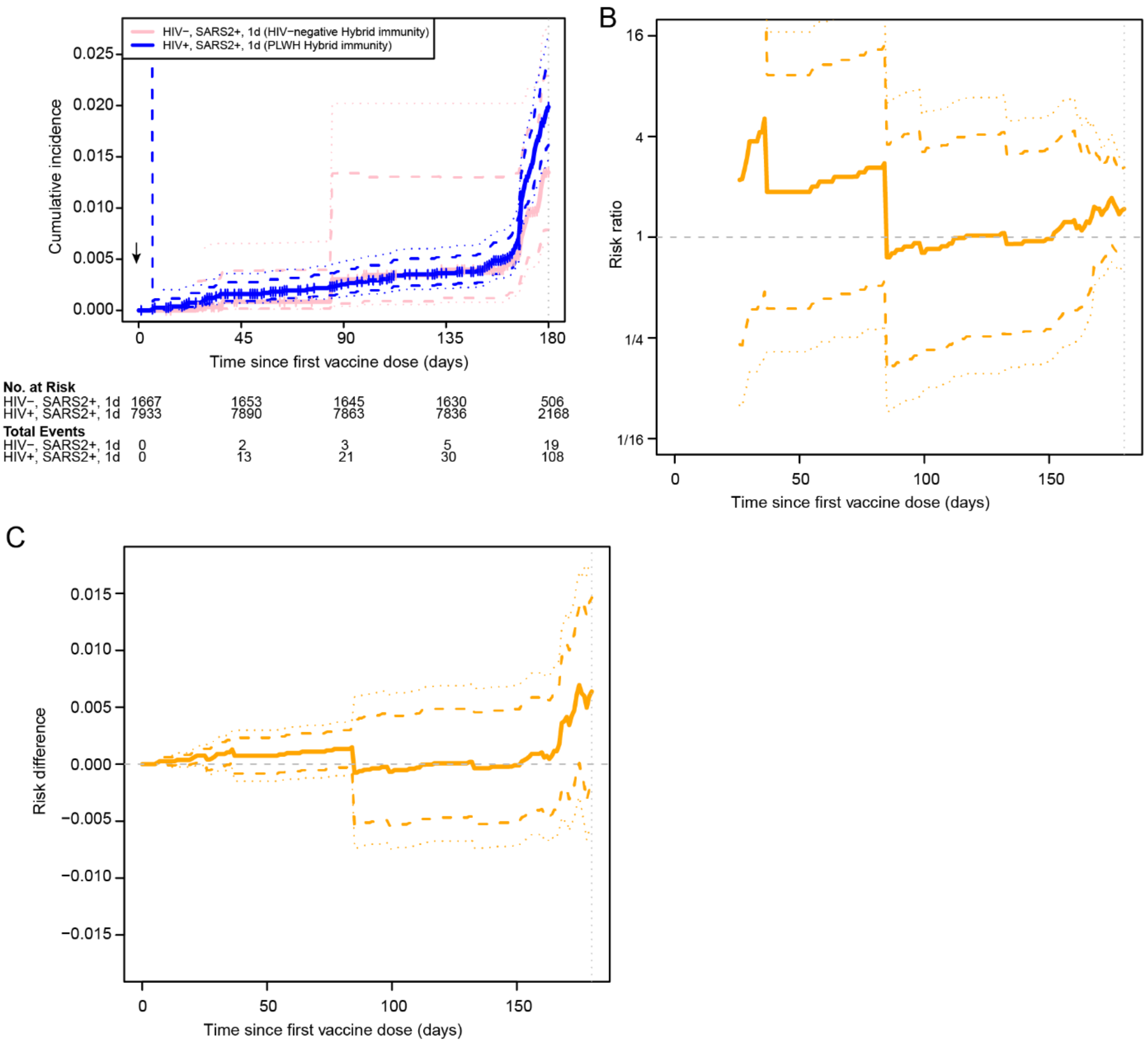

Figure S22. **Comparison of cumulative incidence of Covid-19 (CDC) between exploratory comparison groups HIV+, SARS-CoV-2+, 1 dose versus HIV-, SARS-CoV-2+, 1 dose.** Shown is the cumulative incidence of Covid-19 (CDC) with pointwise and simultaneous 95% confidence intervals based on the Full Analysis Set starting 1 day after first vaccination (**Panel A**) for HIV+, SARS-CoV-2+, 1 dose (AG2-1) vs. HIV-, SARS-CoV-2+, 1 dose (AG4-1). **Panel B** shows the ratio (AG2-1 vs. AG4-1) and **Panel C** the additive difference (AG2-1 minus AG4-1) of cumulative incidence of Covid-19 (CDC) over time. Arrow in panels indicates enrolment, and the tick marks indicate censored data. SARS2, SARS-CoV-2. 1d, one vaccine dose. 2d, two vaccine doses. AG, analysis group.

Figure S23. Comparison of cumulative incidence of Covid-19 (CDC) between exploratory comparison groups HIV+, SARS-CoV-2+, 1 dose versus HIV+, SARS-CoV-2+, 2 doses.

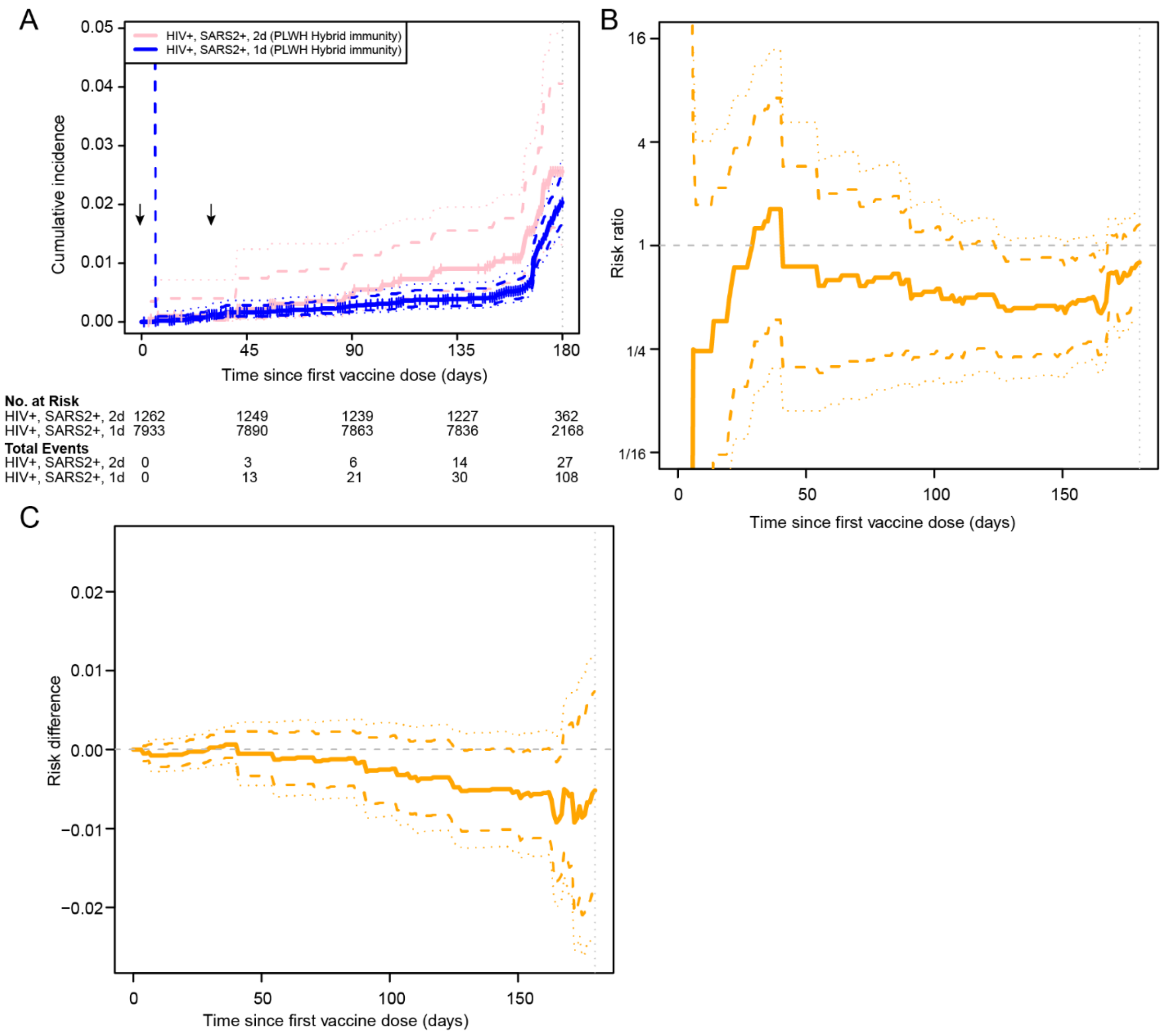

Figure S23. **Comparison of cumulative incidence of Covid-19 (CDC) between exploratory comparison groups HIV+, SARS-CoV-2+, 1 dose versus HIV+, SARS-CoV-2+, 2 doses.** Shown is the cumulative incidence of Covid-19 (CDC) with pointwise and simultaneous 95% confidence intervals based on the Full Analysis Set starting 1 day after first vaccination (**Panel A**) for HIV+ (PLWH), SARS-CoV-2+, 1 dose (AG2-1) vs. PLWH, SARS-CoV-2+, 2 doses (AG2-2). **Panel B** shows the ratio (AG2-1 vs. AG2-2) and **Panel C** the additive difference (AG2-1 minus AG2-2) of cumulative incidence of Covid-19 (CDC) over time. Arrows in panels indicate enrolment and month 1, and the tick marks indicate censored data.

SARS2, SARS-CoV-2. 1d, one vaccine dose. 2d, two vaccine doses. AG, analysis group.

Figure S24. Comparison of cumulative incidence of Covid-19 (CDC) between exploratory comparison groups HIV-, SARS-CoV-2+, 1 dose versus HIV-, SARS-CoV-2+, 2 doses.

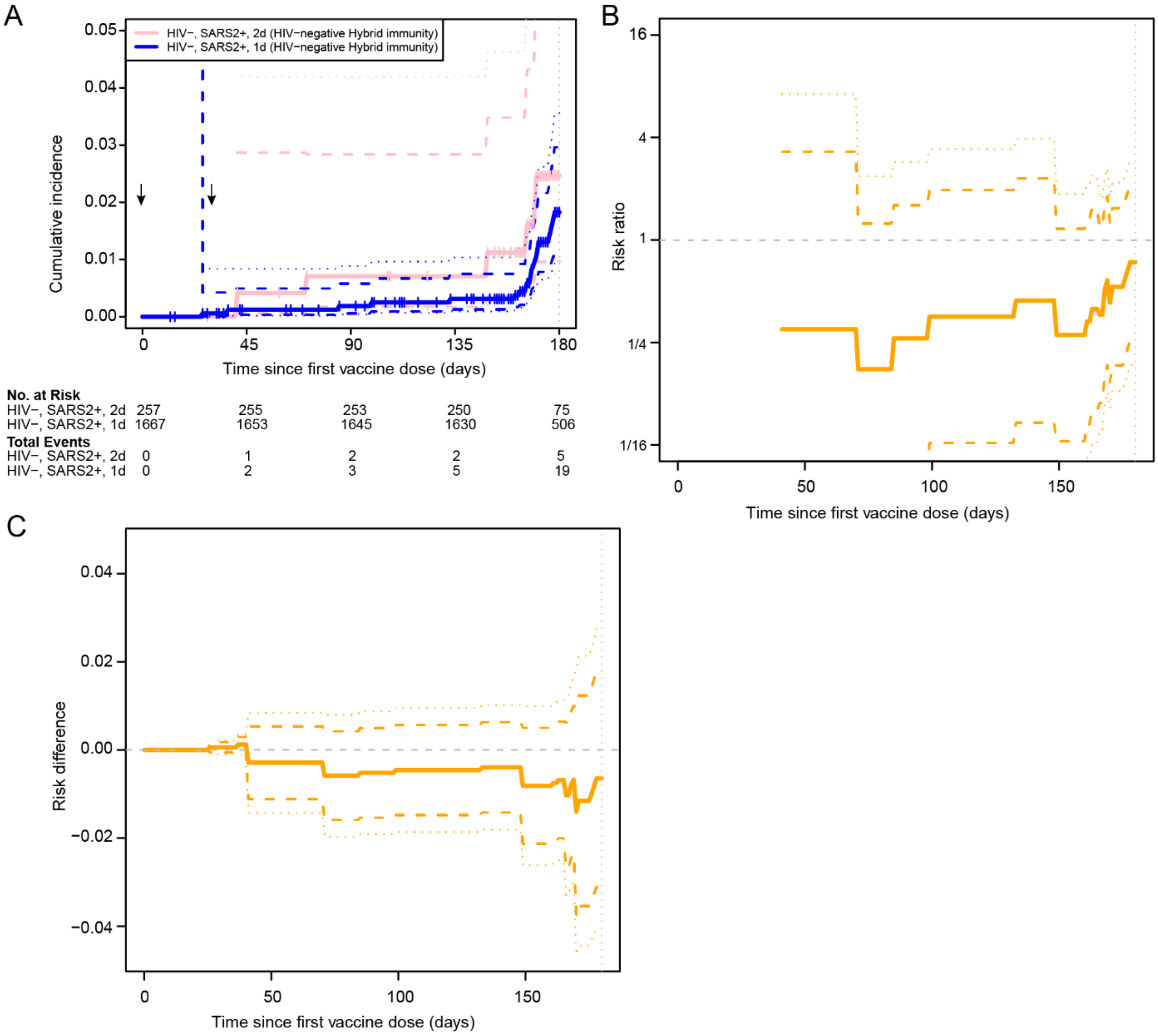

Figure S24. Comparison of cumulative incidence of Covid-19 (CDC) between exploratory comparison groups HIV-, SARS-CoV-2+, 1 dose versus HIV-, SARS-CoV-2+, 2 doses. Shown is the cumulative incidence of Covid-19 (CDC) with pointwise and simultaneous 95% confidence intervals based on the Full Analysis Set starting 1 day after first vaccination (**Panel A**) for HIV-, SARS-CoV-2+, 1 dose (AG4-1) vs. HIV-, SARS-CoV-2+, 2 doses (AG4-2). **Panel B** shows the ratio (AG4-1 vs. AG4-2) and **Panel C** the additive difference (AG4-1 minus AG4-2) of cumulative incidence of Covid-19 (CDC) over time. Arrows in panels indicate enrolment and month 1, and the tick marks indicate censored data. SARS2, SARS-CoV-2. 1d, one vaccine dose. 2d, two vaccine doses. AG, analysis group.

Figure S25. Comparison of cumulative incidence of Covid-19 (CDC) between exploratory comparison groups HIV+, SARS-CoV-2-, 2 doses versus HIV+, SARS-CoV-2+, 2 doses.

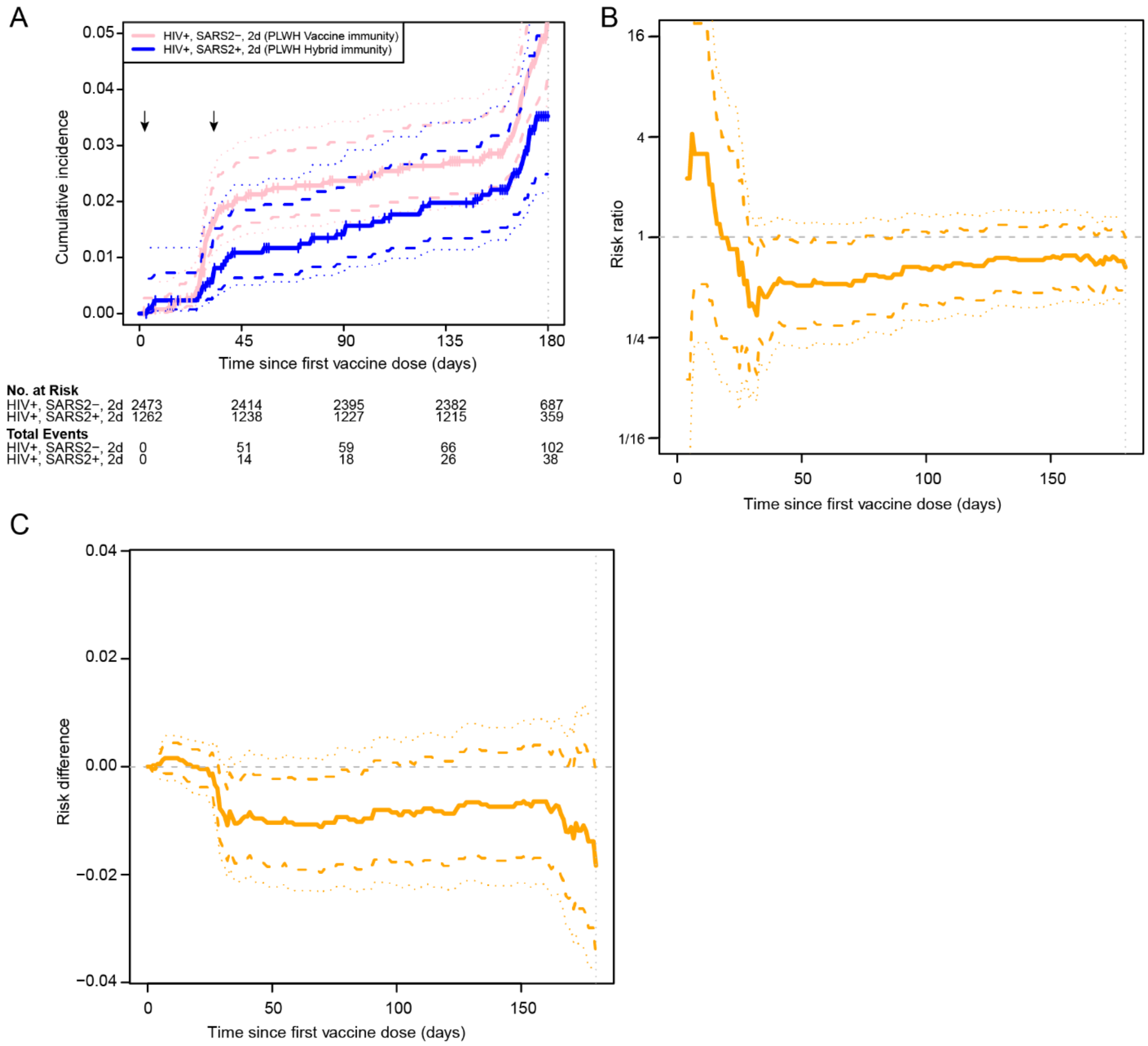

Figure S25. **Comparison of cumulative incidence of Covid-19 (CDC) between exploratory comparison groups HIV+, SARS-CoV-2-, 2 doses versus HIV+, SARS-CoV-2+, 2 doses.** Shown is the cumulative incidence of Covid-19 (CDC) with pointwise and simultaneous 95% confidence intervals based on the Full Analysis Set starting 1 day after first vaccination (**Panel A**) for HIV+, SARS-CoV-2-, 2 doses (AG-1) vs. HIV+, SARS-CoV-2+, 2 doses (AG2-2). **Panel B** shows the ratio (AG-1 vs. AG2-2) and **Panel C** the additive difference (AG-1 minus AG2-2) of cumulative incidence of Covid-19 (CDC) over time. Arrows in panels indicate enrolment and month 1, and the tick marks indicate censored data. SARS2, SARS-CoV-2. 1d, one vaccine dose. 2d, two vaccine doses. AG, analysis group.

Figure S26. Comparison of cumulative incidence of Covid-19 (CDC) between exploratory comparison groups HIV-, SARS-CoV-2-, 2 doses versus HIV-, SARS-CoV-2+, 2 doses.

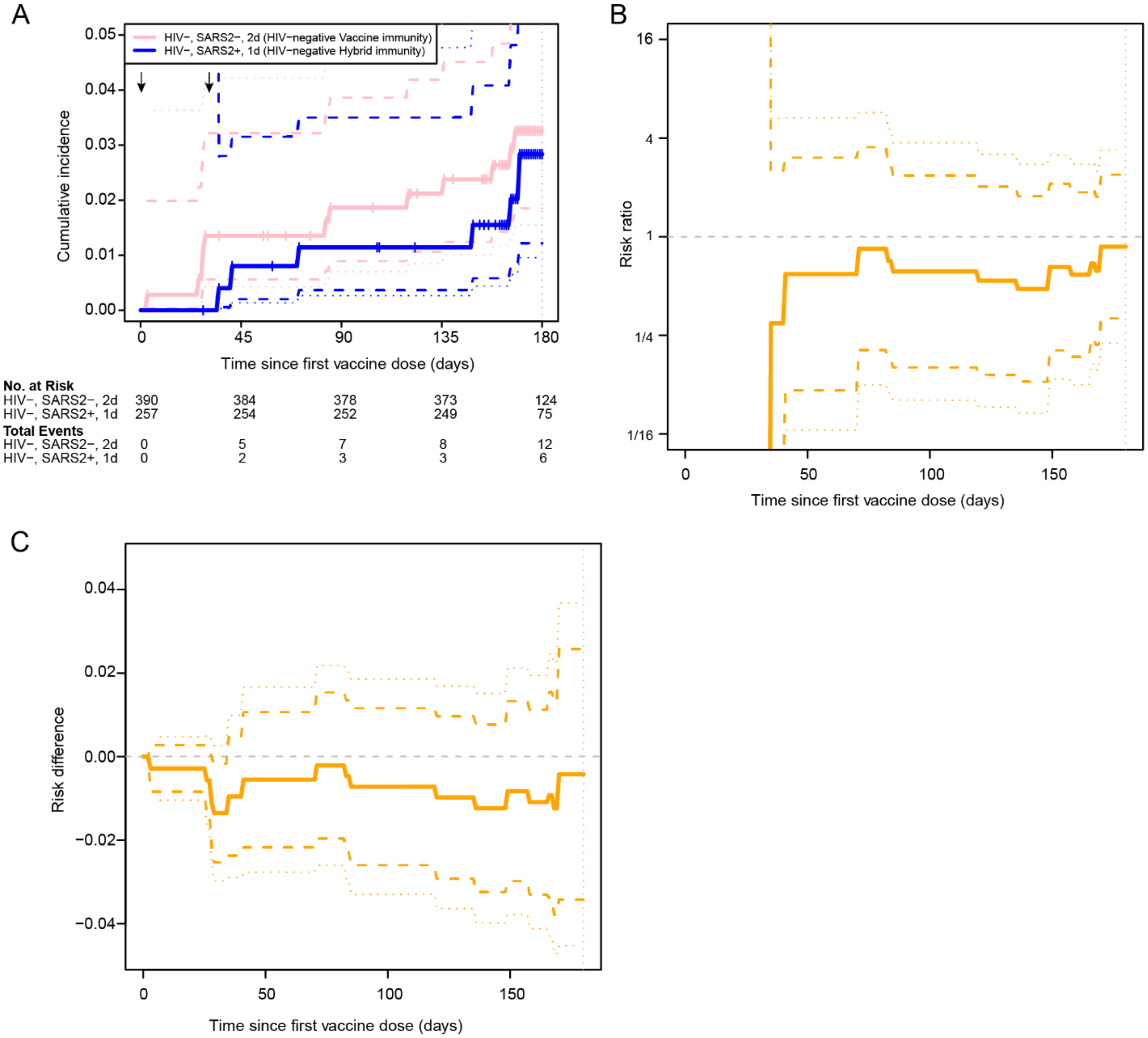

Figure S26. **Comparison of cumulative incidence of Covid-19 (CDC) between exploratory comparison groups HIV-, SARS-CoV-2-, 2 doses versus HIV-, SARS-CoV-2+, 2 doses.** Shown is the cumulative incidence of Covid-19 (CDC) with pointwise and simultaneous 95% confidence intervals based on the Full Analysis Set starting 1 day after first vaccination (**Panel A**) for HIV-, SARS-CoV-2-, 2 doses (AG-3) vs. HIV-, SARS-CoV-2+, 2 doses (AG4-2). **Panel B** shows the ratio (AG-3 vs. AG4-2) and **Panel C** the additive difference (AG-3 minus AG4-2) of cumulative incidence of Covid-19 (CDC) over time. Arrows in panels indicate enrolment and month 1, and the tick marks indicate censored data. SARS2, SARS-CoV-2. 1d, one vaccine dose. 2d, two vaccine doses. AG, analysis group.

Figure S27. Comparison of cumulative incidence of Covid-19 (COVE) between exploratory comparison groups HIV-, SARS-CoV-2-, 2 doses versus HIV-, SARS-CoV-2+, 1 dose.

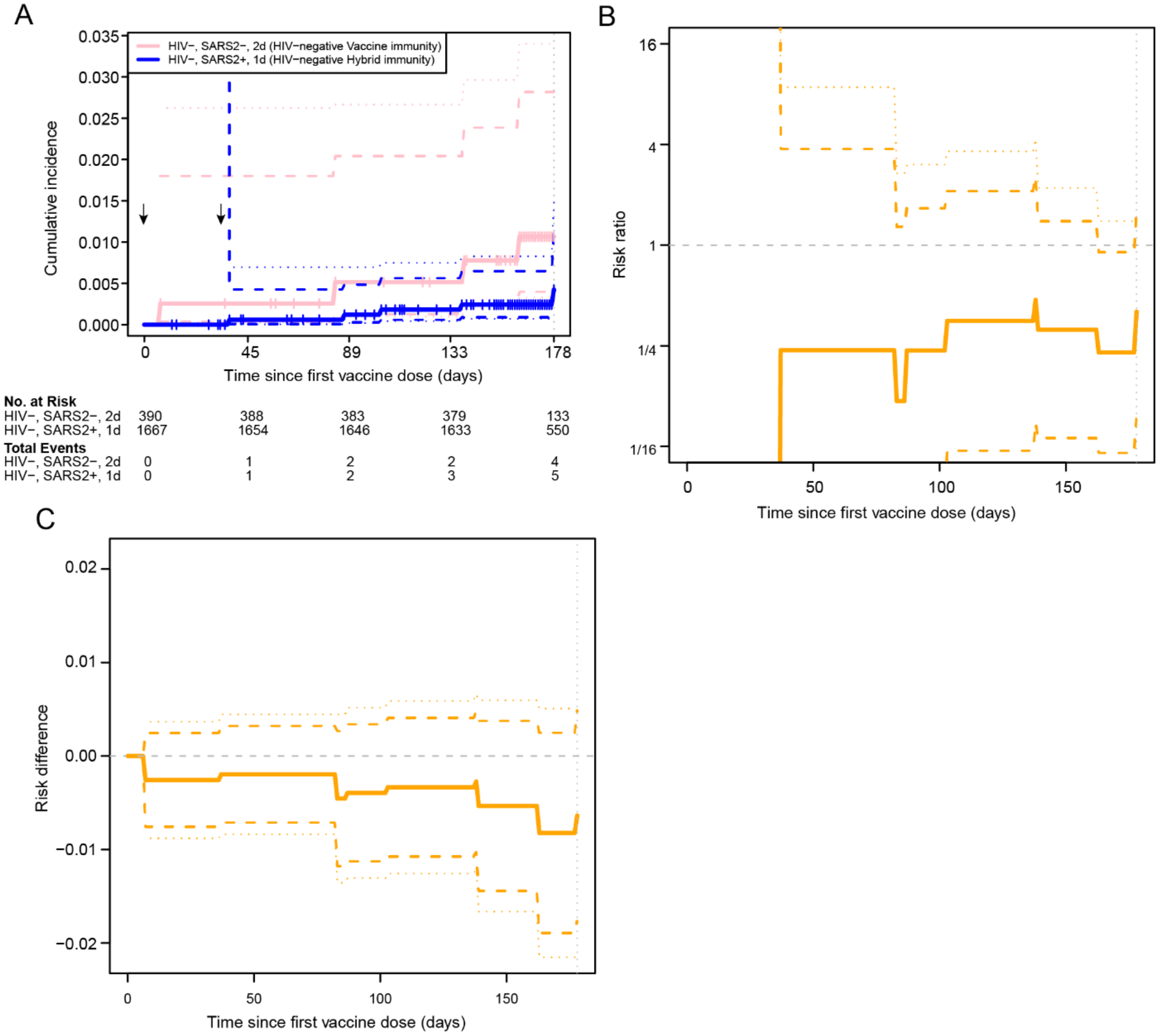

Figure S27. **Comparison of cumulative incidence of Covid-19 (COVE) between exploratory comparison groups HIV-, SARS-CoV-2-, 2 doses versus HIV-, SARS-CoV-2+, 1 dose.** Shown is the cumulative incidence of Covid-19 (COVE) with pointwise and simultaneous 95% confidence intervals based on the Full Analysis Set starting 1 day after first vaccination (**Panel A**) for HIV-, SARS-CoV-2-, 2 doses (AG-3) vs. HIV-, SARS-CoV-2+, 1 dose (AG4-1). **Panel B** shows the ratio (AG-3 vs. AG4-1) and **Panel C** the additive difference (AG-3 minus AG4-1) of cumulative incidence of Covid-19 (COVE) over time. Arrows in panels indicate enrolment and month 1, and the tick marks indicate censored data. SARS2, SARS-CoV-2. 1d, one vaccine dose. 2d, two vaccine doses. AG, analysis group.

Figure S28. Comparison of cumulative incidence of Covid-19 (COVE) between exploratory comparison groups HIV+, SARS-CoV-2-, 2 doses versus HIV-, SARS-CoV-2-, 2 doses.

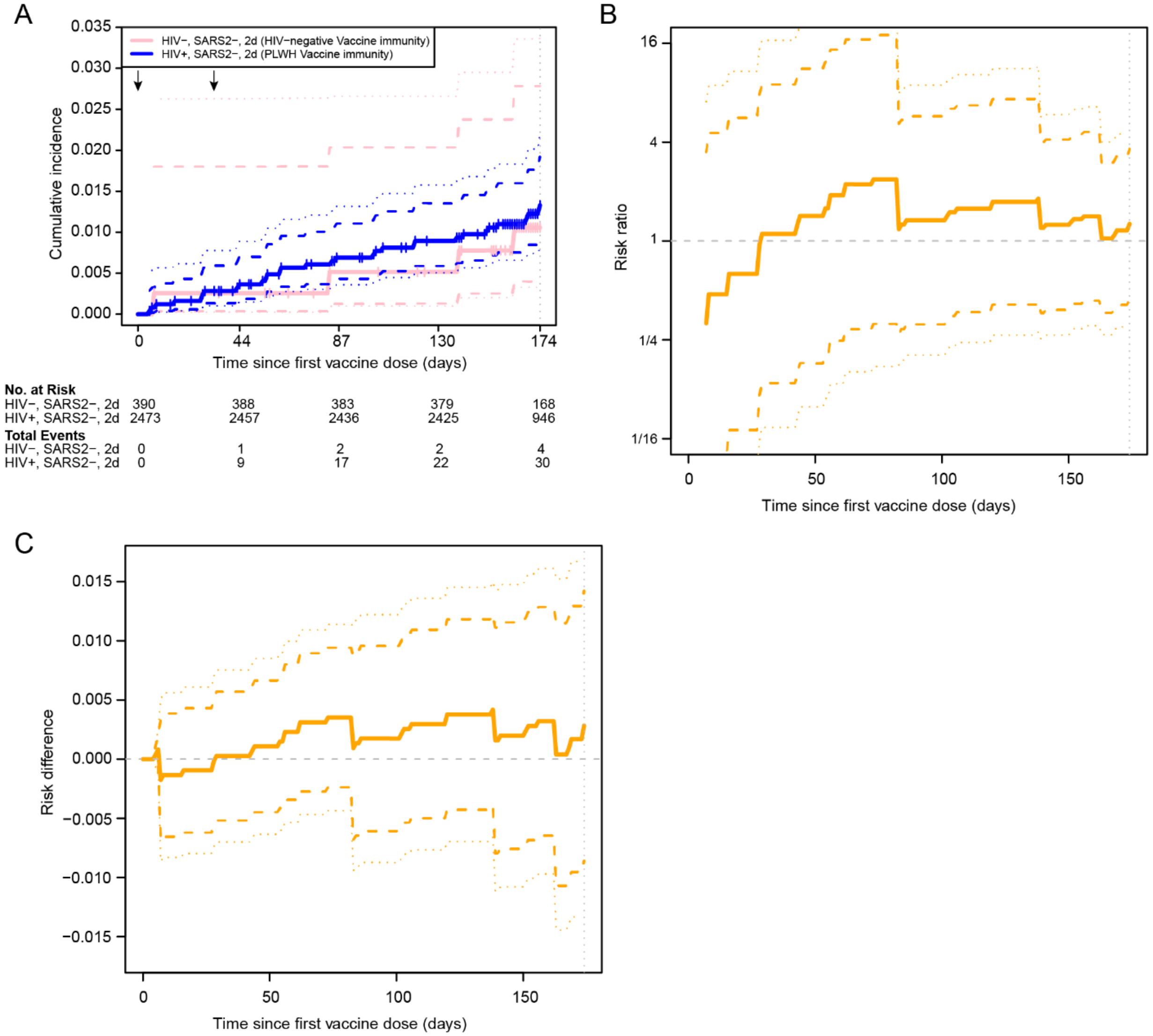

Figure S28. Comparison of cumulative incidence of Covid-19 (COVE) between exploratory comparison groups HIV+, SARS-CoV-2-, 2 doses versus HIV-, SARS-CoV-2-, 2 doses. Shown is the cumulative incidence of Covid-19 (COVE) with pointwise and simultaneous 95% confidence intervals based on the Full Analysis Set (FAS) starting 1 day after first vaccination (**Panel A**) for HIV+, SARS-CoV-2-, 2 doses (AG-1) vs. HIV-, SARS-CoV-2-, 2 doses (AG3). **Panel B** shows the ratio (AG-1 vs. AG3) and **Panel C** the additive difference (AG-1 minus AG3) of cumulative incidence of Covid-19 (COVE) over time. Arrows in panels indicate enrolment and month 1, and the tick marks indicate censored data.

SARS2, SARS-CoV-2. 1d, one vaccine dose. 2d, two vaccine doses. AG, analysis group.

Figure S29. Comparison of cumulative incidence of Covid-19 (COVE) between exploratory comparison groups HIV+, SARS-CoV-2+, 1 dose versus HIV-, SARS-CoV-2+, 1 dose.

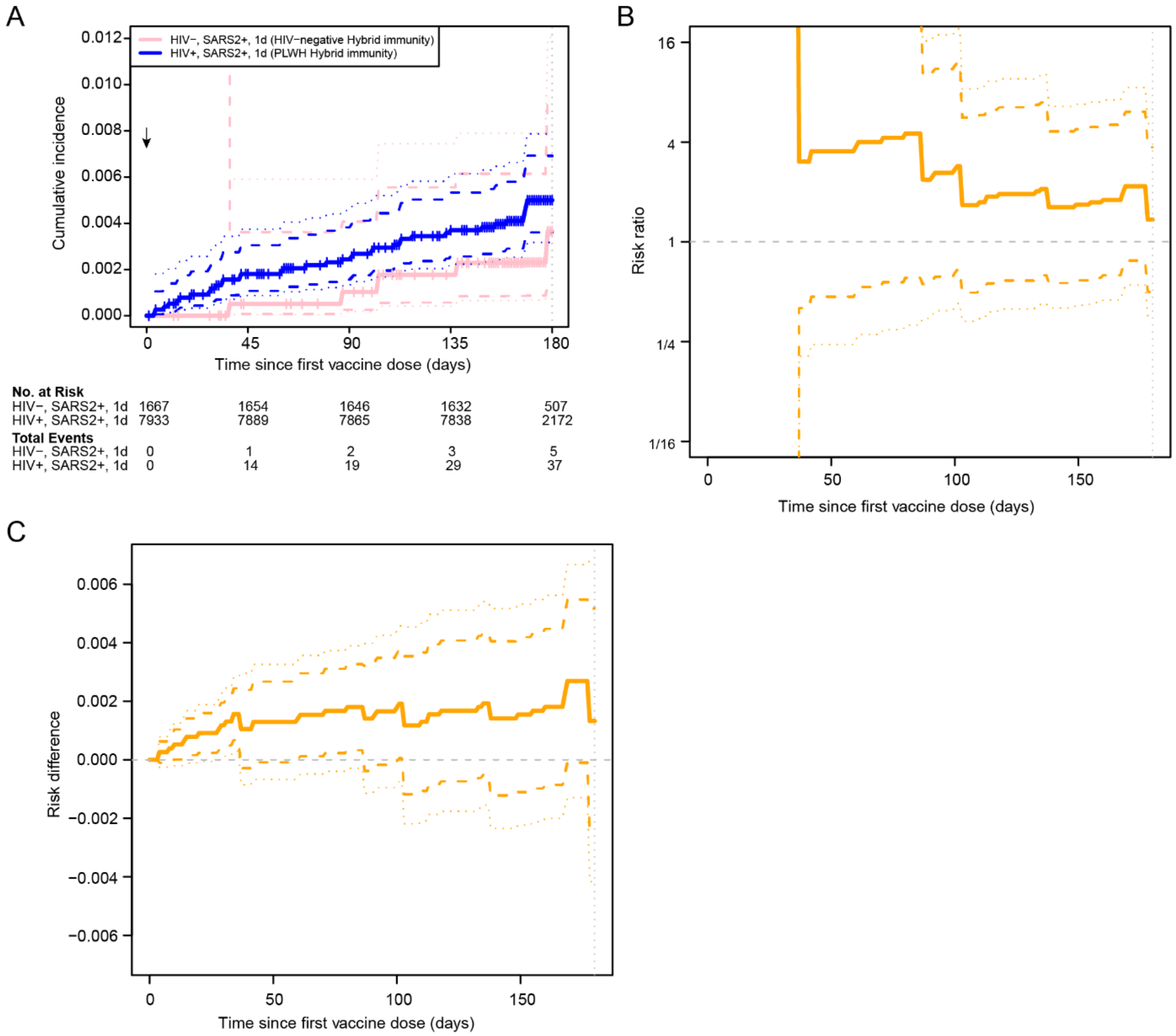

Figure S29. **Comparison of cumulative incidence of Covid-19 (COVE) between exploratory comparison groups HIV+, SARS-CoV-2+, 1 dose versus HIV-, SARS-CoV-2+, 1 dose.** Shown is the cumulative incidence of Covid-19 (COVE) with pointwise and simultaneous 95% confidence intervals based on the Full Analysis Set starting 1 day after first vaccination (**Panel A**) for HIV+, SARS-CoV-2+, 1 dose (AG2-1) vs. HIV-, SARS-CoV-2+, 1 dose (AG4-1). **Panel B** shows the ratio (AG2-1 vs. AG4-1) and **Panel C** the additive difference (AG2-1 minus AG4-1) of cumulative incidence of Covid-19 (COVE) over time. Arrow in panels indicates enrolment, and the tick marks indicate censored data.
SARS2, SARS-CoV-2. 1d, one vaccine dose. 2d, two vaccine doses. AG, analysis group.

Figure S30. Comparison of cumulative incidence of Covid-19 (COVE) between exploratory comparison groups HIV+, SARS-CoV-2+, 1 dose versus HIV+, SARS-CoV-2+, 2 doses.

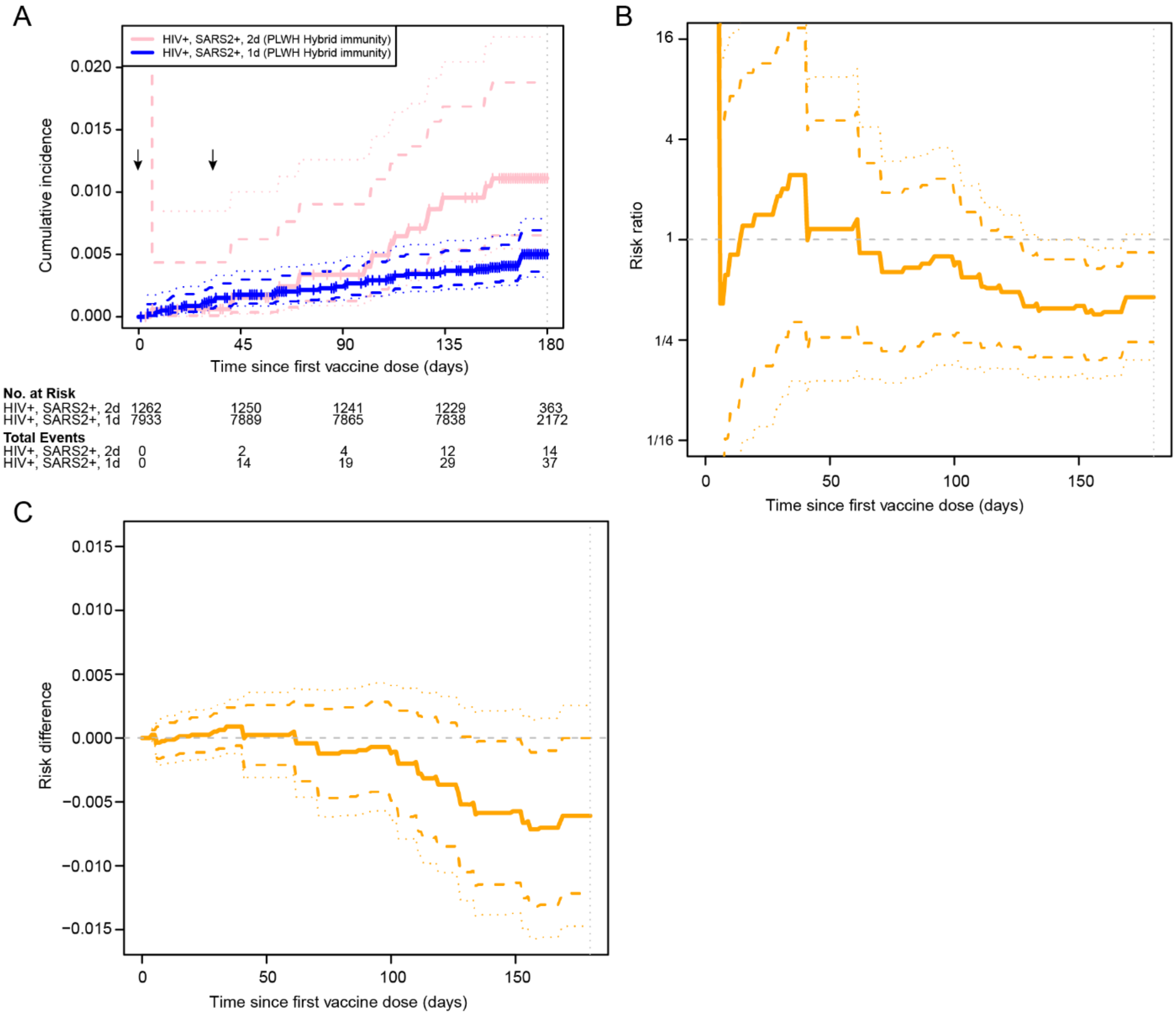

Figure S30. **Comparison of cumulative incidence of Covid-19 (COVE) between exploratory comparison groups HIV+, SARS-CoV-2+, 1 dose versus HIV+, SARS-CoV-2+, 2 doses.** Shown is the cumulative incidence of Covid-19 (COVE) with pointwise and simultaneous 95% confidence intervals based on the Full Analysis Set starting 1 day after first vaccination (**Panel A**) for HIV+, SARS-CoV-2+, 1 dose (AG2-1) vs. HIV+, SARS-CoV-2+, 2 doses (AG2-2). **Panel B** shows the ratio (AG2-1 vs. AG2-2) and **Panel C** the additive difference (AG2-1 minus AG2-2) of cumulative incidence of Covid-19 (COVE) over time. Arrows in panels indicate enrolment and month 1, and the tick marks indicate censored data. SARS2, SARS-CoV-2. 1d, one vaccine dose. 2d, two vaccine doses. AG, analysis group.

Figure S31. Comparison of cumulative incidence of Covid-19 (COVE) between exploratory comparison groups HIV-, SARS-CoV-2+, 1 dose versus HIV-, SARS-CoV-2+, 2 doses.

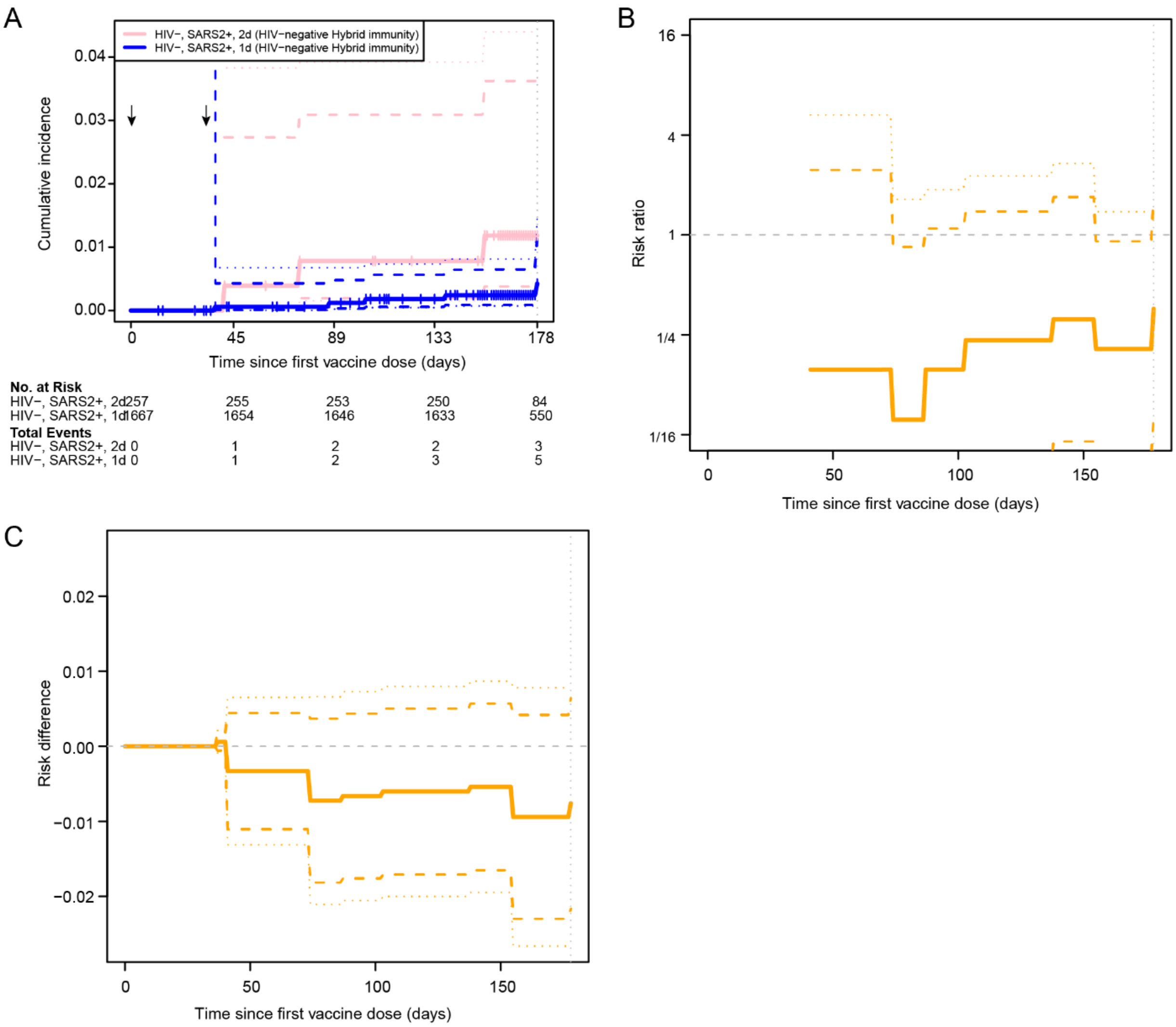

Figure S31. **Comparison of cumulative incidence of Covid-19 (COVE) between exploratory comparison groups HIV-, SARS-CoV-2+, 1 dose versus HIV-, SARS-CoV-2+, 2 doses.** Shown is the cumulative incidence of Covid-19 (COVE) with pointwise and simultaneous 95% confidence intervals based on the Full Analysis Set starting 1 day after first vaccination (**Panel A**) for HIV-, SARS-CoV-2+, 1 dose (AG4-1) vs. HIV-, SARS-CoV-2+, 2 doses (AG4-2). **Panel B** shows the ratio (AG4-1 vs. AG4-2) and **Panel C** the additive difference (AG4-1 minus AG4-2) of cumulative incidence of Covid-19 (COVE) over time. Arrows in panels indicate enrolment and month 1, and the tick marks indicate censored data. SARS2, SARS-CoV-2. 1d, one vaccine dose. 2d, two vaccine doses. AG, analysis group.

Figure S32. Comparison of cumulative incidence of Covid-19 (COVE) between exploratory comparison groups HIV+, SARS-CoV-2-, 2 doses versus HIV+, SARS-CoV-2+, 2 doses.

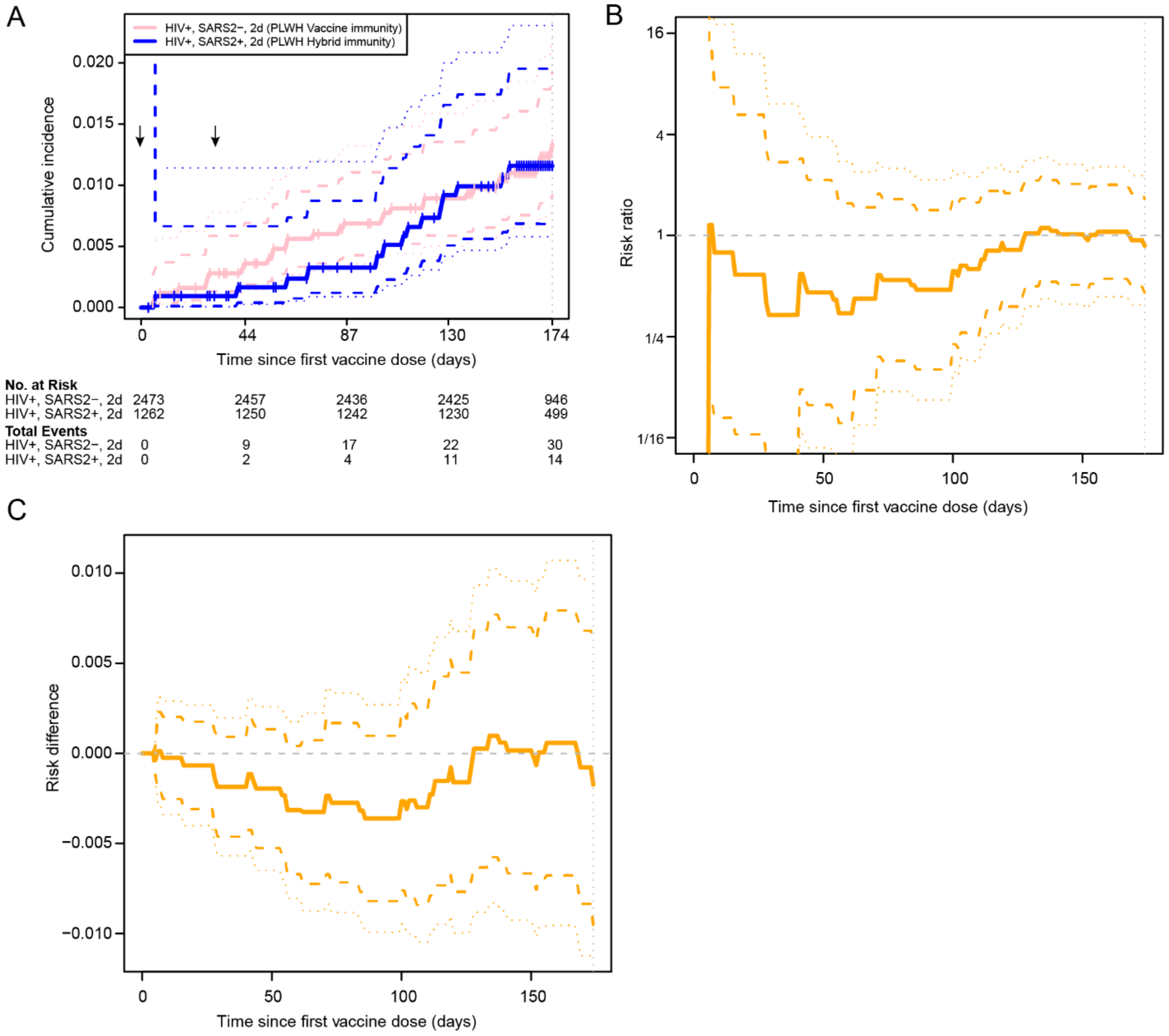

Figure S32. **Comparison of cumulative incidence of Covid-19 (COVE) between exploratory comparison groups HIV+, SARS-CoV-2-, 2 doses versus HIV+, SARS-CoV-2+, 2 doses.** Shown is the cumulative incidence of Covid-19 (COVE) with pointwise and simultaneous 95% confidence intervals based on the Full Analysis Set starting 1 day after first vaccination (**Panel A**) for HIV+, SARS-CoV-2-, 2 doses (AG-1) vs. HIV+, SARS-CoV-2+, 2 doses (AG2-2). **Panel B** shows the ratio (AG-1 vs. AG2-2) and **Panel C** the additive difference (AG-1 minus AG2-2) of cumulative incidence of Covid-19 (COVE) over time. Arrows in panels indicate enrolment and month 1, and the tick marks indicate censored data. SARS2, SARS-CoV-2. 1d, one vaccine dose. 2d, two vaccine doses. AG, analysis group.

Figure S33. Comparison of cumulative incidence of Covid-19 (COVE) between exploratory comparison groups HIV-, SARS-CoV-2-, 2 doses versus HIV-, SARS-CoV-2+, 2 doses.

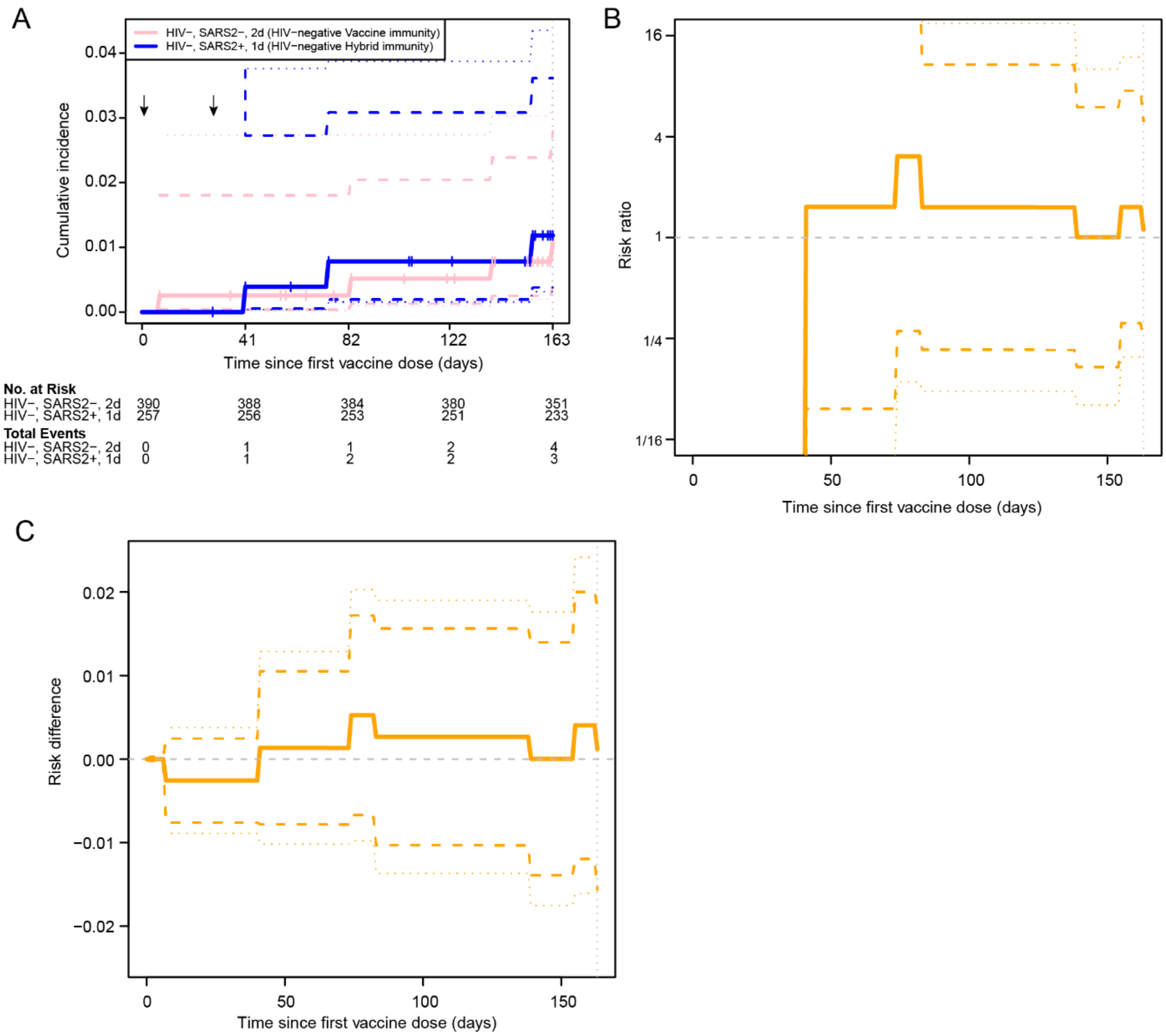

Figure S33. **Comparison of cumulative incidence of Covid-19 (COVE) between exploratory comparison groups HIV-, SARS-CoV-2-, 2 doses versus HIV-, SARS-CoV-2+, 2 doses.** Shown is the cumulative incidence of Covid-19 (COVE) with pointwise and simultaneous 95% confidence intervals based on the Full Analysis Set starting 1 day after first vaccination (**Panel A**) for HIV-, SARS-CoV-2-, 2 doses (AG-3) vs. HIV-, SARS-CoV-2+, 2 doses (AG4-2). **Panel B** shows the ratio (AG-3 vs. AG4-2) and **Panel C** the additive difference (AG-3 minus AG4-2) of cumulative incidence of Covid-19 (COVE) over time. Arrows in panels indicate enrolment and month 1, and the tick marks indicate censored data. SARS2, SARS-CoV-2. 1d, one vaccine dose. 2d, two vaccine doses. AG, analysis group
.

Figure S34. Association of CD4 count (< vs. ≥ 350 cells/μl) and HIV viremia (< vs. ≥ 50 copies/ml) with risk of Covid-19 in people living with HIV, within strata defined by hybrid and vaccine immunity.

Analysis Group	Comparison	No. of Endpoints	No. of Person-Years	Rate per 100 Person-Years	Hazard Ratio (95% CI)	P-value
HIV+, SARS2-, 2d	CD4 count ≥350 cells/µl	88	884	9.95		
	CD4 count <350 cells/µl	31	298	10.40	1.20 [0.78, 1.83]	0.403
HIV+, SARS2-, 2d	HIV viraemia (≥50 copies/ml)	27	387	6.98		
	HIV viral load suppressed (<50 copies/ml)	92	796	11.56	1.38 [0.89, 2.14]	0.144
HIV+, SARS2+, 1d	CD4 count ≥350 cells/µl	124	3320	3.73		
	CD4 count <350 cells/µl	27	536	5.04	1.43 [0.93, 2.19]	0.107
HIV+, SARS2+, 1d	HIV viraemia (≥50 copies/ml)	47	859	5.47		
	HIV viral load suppressed (<50 copies/ml)	104	2997	3.47	0.56 [0.39, 0.79]	0.001

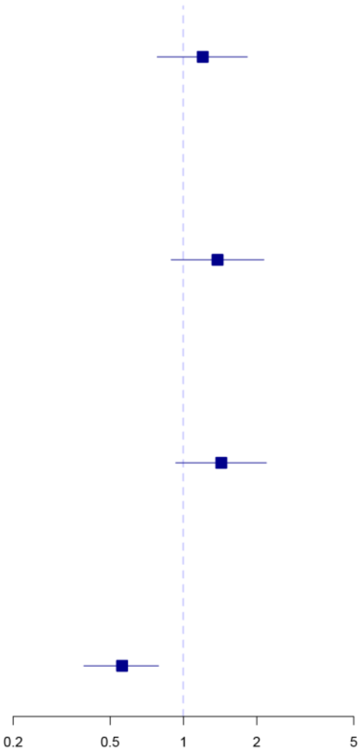

Figure S34. Association of CD4 count (< vs. ≥ 350 cells/µl) and HIV viremia (< vs. ≥ 50 copies/ml) with risk of Covid-19 in people living with HIV, within strata defined by hybrid and vaccine immunity. Forest-plot shows hazard ratios of CDC case definition Covid-19 among PLWH with CD4 counts <350 cells/µl compared to ≥350 cells/µl, and among PLWH with HIV viremia compared to those with viral suppression in the FAS within each hybrid versus vaccine immunity stratum.
SARS2, SARS-CoV-2. 1d, one vaccine dose. 2d, two vaccine doses.

Figure S35. Lineages of SARS-CoV-2 associated with diagnosis of Covid-19 (CDC) by analysis group in the Full Analysis Set.

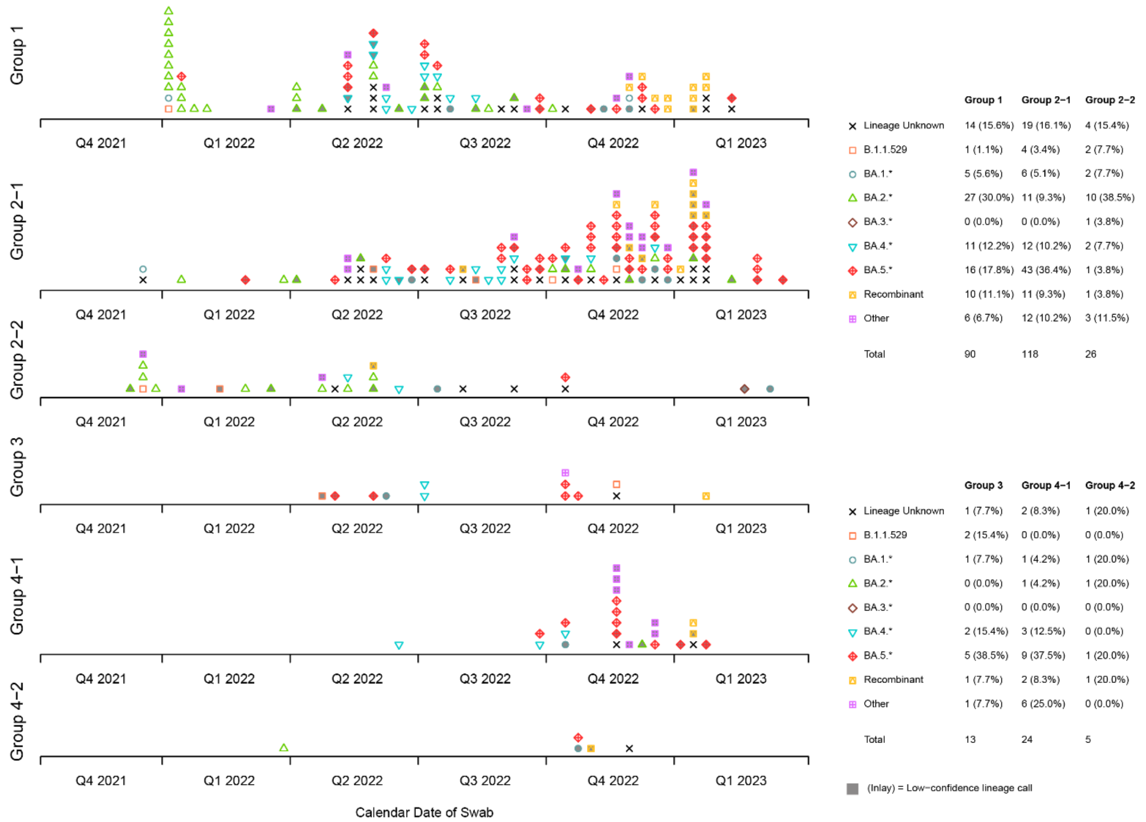

Figure S35. Lineages of SARS-CoV-2 associated with diagnosis of Covid-19 (CDC) by analysis group in the Full Analysis Set. Shown are numbers of Covid-19 cases by SARS-CoV-2 lineage through 6 months of follow-up starting after first dose for the six analysis groups (AGs). AG1 represents people living with HIV (PLWH), overall SARS-CoV-2 status negative, and assigned 2 doses (PLWH, vaccine immunity). AG2-1 represents PLWH, overall SARS-CoV-2 status positive (POC anti-S positive), and assigned 1 dose (PLWH, hybrid immunity). AG2-2 represents PLWH, overall SARS-CoV-2 status positive (POC anti-S negative but anti-NP or NAAT positive), and assigned 2 doses. AG3 represents HIV-negative (PLWoH), SARS-CoV-2 status negative, and assigned 2 doses (PLWoH, vaccine immunity). AG4-1 represents PLWoH, overall SARS-CoV-2 status positive (POC anti-S positive), and assigned 1 dose (PLWoH, hybrid immunity). AG4-2 represents PLWoH, overall SARS-CoV-2 status positive (POC anti-S negative but anti-NP or NAAT positive), and assigned 2 doses. The viral lineage is illustrated by the color and plot character. Lineage typing was performed with both PANGOLIN and NextClade, and the call with the highest confidence was selected (see Methods). Due to insufficient viral material, many sequences were either (i) unable to be obtained (indicated by "Lineage Unknown"); (ii) exhibited such a degree of missingness that they were incapable of being lineage typed (also indicated by "Lineage Unknown"), or (iii) obtained a lineage from at least one platform, but failed QC on both platforms. These latter sequences are regarded as having low-confidence lineage calls and are indicated in this figure by having their plot character inlaid with gray. Most sequences were lineage-typed within Omicron, so these sequences are grouped in the figure by the basal Omicron lineage (B.1.1.529), the five major Omicron sub-lineages (BA.1 through BA.5; e.g., BE.7 is classified as "BA.5.*") or recombinant status (e.g., XBB). Non-Omicron lineages, almost exclusively occurring among the low-confidence lineage calls, are classified as "Other".

Q, three-month quarter: Q1 (January-March), Q2 (April-June), Q3 (July-September), and Q4 (October-December).

Figure S36. Lineages of SARS-CoV-2 associated with diagnosis of Covid-19 (COVE) by analysis group in the Full Analysis Set.

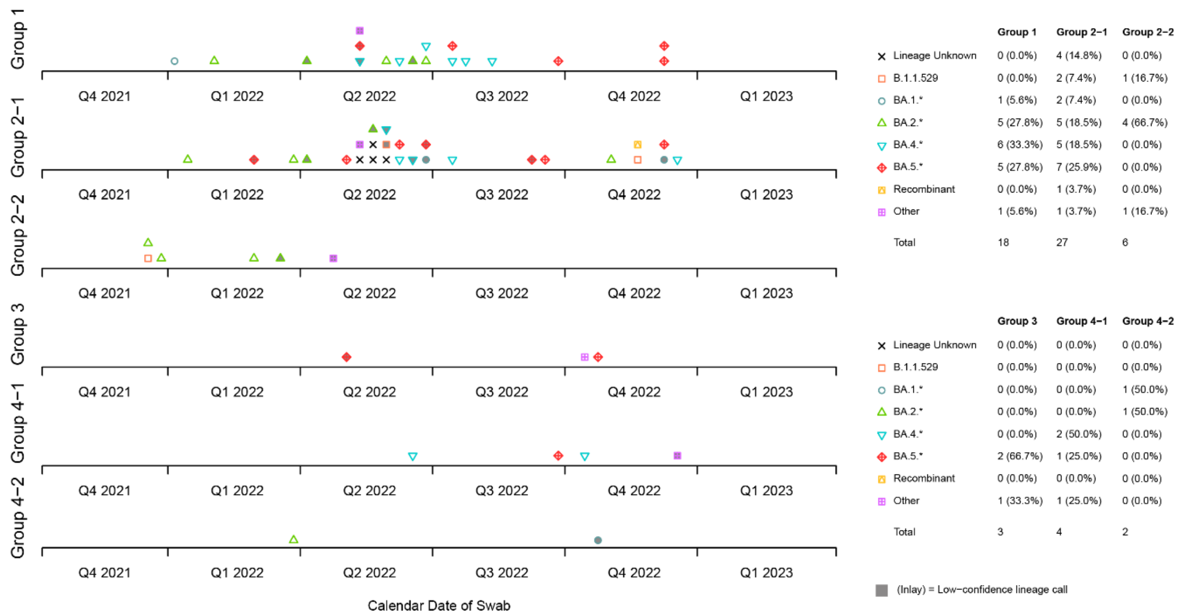

Figure S36. Lineages of SARS-CoV-2 associated with diagnosis of Covid-19 (COVE) by analysis group in the Full Analysis Set. Shown are numbers of Covid-19 cases by SARS-CoV-2 lineage through 6 months of follow-up starting after the first dose for the six analysis groups (AGs). AG1 represents people living with HIV (PLWH), overall SARS-CoV-2 status negative, and assigned 2 doses (PLWH, vaccine immunity). AG2-1 represents PLWH, overall SARS-CoV-2 status positive (POC anti-S positive), and assigned 1 dose (PLWH, hybrid immunity). AG2-2 represents PLWH, overall SARS-CoV-2 status positive (POC anti-S negative but anti-NP or NAAT positive), and assigned 2 doses. AG3 represents people living without HIV (PLWoH), SARS-CoV-2 status negative, and assigned 2 doses (PLWoH, vaccine immunity). AG4-1 represents PLWoH, overall SARS-CoV-2 status positive (POC anti-S positive), and assigned 1 dose (PLWoH, hybrid immunity). AG4-2 represents PLWoH, overall SARS-CoV-2 status positive (POC anti-S negative but anti-NP or NAAT positive), and assigned 2 doses. The viral lineage is illustrated by the color and plot character. Lineage typing was performed with both PANGOLIN and NextClade, and the call with the highest confidence was selected (see Methods). Due to insufficient viral material, many sequences were either (i) unable to be obtained (indicated by "Lineage Unknown"); (ii) exhibited such a degree of missingness that they were incapable of being lineage typed (also indicated by "Lineage Unknown"), or (iii) obtained a lineage from at least one platform, but failed QC on both platforms. These latter sequences are regarded as having low-confidence lineage calls and are indicated in this figure by having their plot character inlaid with gray. Most sequences were lineage-typed within Omicron, so these sequences are grouped in the figure by the basal Omicron lineage (B.1.1.529), the five major Omicron sub-lineages (BA.1 through BA.5; e.g., BE.7 is classified as "BA.5.*") or recombinant status (e.g., XBB). Non-Omicron lineages, almost exclusively occurring among the low-confidence lineage calls, are classified as "Other".
Q, three-month quarter: Q1 (January-March), Q2 (April-June), Q3 (July-September), and Q4 (October-December).

Figure S37. Lineages of all baseline and post-baseline positive nucleic acid amplification tests by analysis group in the Full Analysis Set.

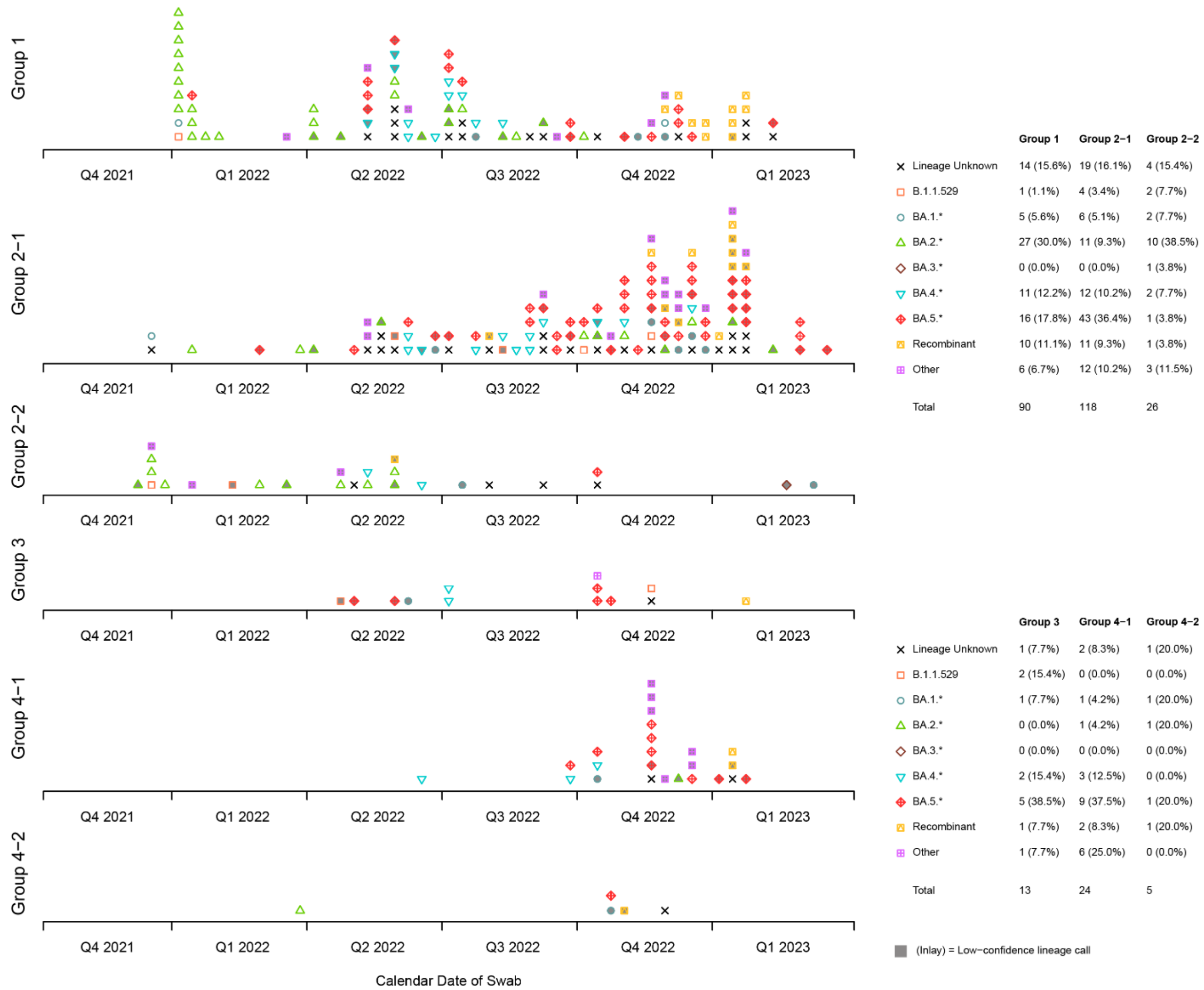

Figure S37. Lineages of all baseline and post-baseline positive nucleic acid amplification tests by analysis group in the Full Analysis Set. There are six analysis groups (AGs): AG1 represents people living with HIV (PLWH), overall SARS-CoV-2 status negative, and assigned 2 doses (PLWH, vaccine immunity). AG2-1 represents PLWH, overall SARS-CoV-2 status positive (POC anti-S positive), and assigned 1 dose (PLWH, hybrid immunity). AG2-2 represents PLWH, overall SARS-CoV-2 status positive (POC anti-S negative but anti-NP or NAAT positive), and assigned 2 doses. AG3 represents people living without HIV (PLWoH), SARS-CoV-2 status negative, and assigned 2 doses (PLWoH, vaccine immunity). AG4-1 represents PLWoH, overall SARS-CoV-2 status positive (POC anti-S positive), and assigned 1 dose (PLWoH, hybrid immunity). AG4-2 represents PLWoH, overall SARS-CoV-2 status positive (POC anti-S negative but anti-NP or NAAT positive), and assigned 2 doses. The viral lineage is illustrated by the color and plot character. Lineage typing was performed with both PANGOLIN and NextClade, and the call with the highest confidence was selected (see Methods). Due to insufficient viral material, many sequences were either (i) unable to be obtained (indicated by "Lineage Unknown"); (ii) exhibited such a degree of missingness that they were incapable of being lineage typed (also indicated by "Lineage Unknown"), or (iii) obtained a lineage from at least one platform, but failed QC on both platforms. These latter sequences are regarded as having low-confidence lineage calls and are

indicated in this figure by having their plot character inlaid with gray. Most sequences were lineage-typed within Omicron, so these sequences are grouped in the figure by the basal Omicron lineage (B.1.1.529), the five major Omicron sub-lineages (BA.1 through BA.5; e.g., BE.7 is classified as "BA.5.*") or recombinant status (e.g., XBB). Non-Omicron lineages, almost exclusively occurring among the low-confidence lineage calls, are classified as "Other."
Q, three-month quarter: Q1 (January-March), Q2 (April-June), Q3 (July-September), and Q4 (October-December).

References

1. Division of AIDS. Table for Grading the Severity of Adult and Pediatric Adverse Events, Corrected Version 2.1. Published July 2017. Accessed December 20, 2023. https://rsc.niaid.nih.gov/sites/default/files/daidsgradingcorrectedv21.pdf.

2. Rambaut A, Holmes EC, O'Toole Á, Hill V, McCrone JT, Ruis C, du Plessis L & Pybus OG (2020) *Nature Microbiology* DOI:10.1038/s41564-020-0770-5.

3. Aksamentov et al., (2021). Nextclade: clade assignment, mutation calling and quality control for viral genomes. Journal of Open Source Software, 6(67), 3773, https://doi.org/10.21105/joss.03773.

4. Nextstrain. Nextclade Pango Algorithm. Nextstrain Docs. Accessed December 20, 2023. https://docs.nextstrain.org/projects/nextclade/en/stable/user/algorithm/nextclade-pango.html.
